# Waning effectiveness of BNT162b2 and ChAdOx1 covid-19 vaccines over six months since second dose: OpenSAFELY cohort study using linked electronic health records

**DOI:** 10.1136/bmj-2022-071249

**Published:** 2022-07-20

**Authors:** Elsie M F Horne, William J Hulme, Ruth H Keogh, Tom M Palmer, Elizabeth J Williamson, Edward P K Parker, Amelia Green, Venexia Walker, Alex J Walker, Helen Curtis, Louis Fisher, Brian MacKenna, Richard Croker, Lisa Hopcroft, Robin Y Park, Jon Massey, Jessica Morley, Amir Mehrkar, Sebastian Bacon, David Evans, Peter Inglesby, Caroline E Morton, George Hickman, Simon Davy, Tom Ward, Iain Dillingham, Ben Goldacre, Miguel A Hernán, Jonathan A C Sterne

**Affiliations:** 1 Population Health Sciences, University of Bristol, Bristol, UK; 2 NIHR Bristol Biomedical Research Centre, Bristol, UK; 3 The DataLab, Nuffield Department of Primary Care Health Sciences, University of Oxford, Oxford, UK; 4 London School of Hygiene and Tropical Medicine, London, UK; 5 MRC Integrative Epidemiology Unit, University of Bristol, Bristol, UK; 6 CAUSALab, Harvard T.H. Chan School of Public Health, Boston, MA, USA; 7 Departments of Epidemiology and Biostatistics, Harvard T.H. Chan School of Public Health, Boston, USA; 8 Health Data Research UK South-West, Bristol, UK; * Contributed equally

## Abstract

**Objective:**

To estimate waning of covid-19 vaccine effectiveness over six months after second dose.

**Design:**

Cohort study, approved by NHS England.

**Setting:**

Linked primary care, hospital, and covid-19 records within the OpenSAFELY-TPP database.

**Participants:**

Adults without previous SARS-CoV-2 infection were eligible, excluding care home residents and healthcare professionals.

**Exposures:**

People who had received two doses of BNT162b2 or ChAdOx1 (administered during the national vaccine rollout) were compared with unvaccinated people during six consecutive comparison periods, each of four weeks.

**Main outcome measures:**

Adjusted hazard ratios for covid-19 related hospital admission, covid-19 related death, positive SARS-CoV-2 test, and non-covid-19 related death comparing vaccinated with unvaccinated people. Waning vaccine effectiveness was quantified as ratios of adjusted hazard ratios per four week period, separately for subgroups aged ≥65 years, 18-64 years and clinically vulnerable, 40-64 years, and 18-39 years.

**Results:**

1 951 866 and 3 219 349 eligible adults received two doses of BNT162b2 and ChAdOx1, respectively, and 2 422 980 remained unvaccinated. Waning of vaccine effectiveness was estimated to be similar across outcomes and vaccine brands. In the ≥65 years subgroup, ratios of adjusted hazard ratios for covid-19 related hospital admission, covid-19 related death, and positive SARS-CoV-2 test ranged from 1.19 (95% confidence interval 1.14 to 1.24)to 1.34 (1.09 to 1.64) per four weeks. Despite waning vaccine effectiveness, rates of covid-19 related hospital admission and death were substantially lower among vaccinated than unvaccinated adults up to 26 weeks after the second dose, with estimated vaccine effectiveness ≥80% for BNT162b2, and ≥75% for ChAdOx1. By weeks 23-26, rates of positive SARS-CoV-2 test in vaccinated people were similar to or higher than in unvaccinated people (adjusted hazard ratios up to 1.72 (1.11 to 2.68) for BNT162b2 and 1.86 (1.79 to 1.93) for ChAdOx1).

**Conclusions:**

The rate at which estimated vaccine effectiveness waned was consistent for covid-19 related hospital admission, covid-19 related death, and positive SARS-CoV-2 test and was similar across subgroups defined by age and clinical vulnerability. If sustained to outcomes of infection with the omicron variant and to booster vaccination, these findings will facilitate scheduling of booster vaccination.

## Introduction

The effectiveness of covid-19 vaccines, first shown in randomised trials,^1^ ^2^ has been confirmed with longer follow-up in observational studies.^3^ ^4^ ^5^ However, neutralising antibody titres decrease with time since vaccination,^6^ ^7^ ^8^ and vaccine effectiveness against infection wanes over time.^5^ ^9^ ^10^ ^11^ ^12^ ^13^ ^14^ ^15^ ^16^ The extent of waning of vaccine effectiveness against severe covid-19 is less clear: studies have found no evidence of waning,^9^ ^10^ ^17^ modest waning,^5^ ^11^ or substantial waning.^12^ Clarification of rates of waning effectiveness is needed to determine the frequency with which booster doses are needed and whether booster vaccination should be targeted at groups defined by age, clinical vulnerability, or brand of primary vaccine.

Examination of waning of covid-19 vaccine effectiveness is difficult. The success of vaccine rollouts in many countries means that only a small and selected proportion of the population remains unvaccinated and uninfected. Continuing uptake of vaccination, as well as ongoing infection with SARS-CoV-2, further depletes this group over time. Vaccines were offered in priority order determined by age and clinical vulnerability, so that the longest follow-up is in people at the highest risk of severe covid-19. Rapid changes in rates of SARS-CoV-2 infection over time, related to pandemic control measures and introduction of new variants, make accounting for the calendar date on which events occurred essential. Many studies of waning covid-19 vaccine effectiveness have used “test negative case-control” designs, restricted to people tested for infection with SARS-CoV-2 and comparing those testing positive (cases) and negative (controls)^5^ ^9^ ^17^ ^18^ or reported indirect evidence such as changing rates of covid-19 with time since vaccination.^11^ ^12^ The extent to which test negative case-control designs control bias due to confounding, or are biased because of the restriction to people who were tested, remains unclear.^19^ ^20^

We did a cohort study within the OpenSAFELY-TPP database (https://opensafely.org), which includes detailed linked data on 24 million people (approximately 44% of the English population) registered with an English general practice using The Phoenix Partnership (TPP) SystmOne electronic health record software. We compared rates of covid-19 related hospital admission, covid-19 related and non-covid-19 related mortality, and infection with SARS-CoV-2, between adults fully vaccinated with the Pfizer-BioNTech BNT162b2 mRNA vaccine (BNT162b2) or the Oxford-AstraZeneca ChAdOx1 nCoV-19 AZD1222 (ChAdOx1) vaccine and those who were unvaccinated.

## Methods

### Data source

OpenSAFELY-TPP includes detailed pseudonymised primary care data linked (via National Health Service (NHS) number) with accident and emergency attendance, inpatient hospital spell records (NHS Digital’s Hospital Episode Statistics dataset), national SARS-CoV-2 testing records (Second Generation Surveillance System; SGSS), and national death registry records. Vaccination status (National Immunisation Management System; NIMS) is available in the primary care record. Healthcare worker status (recorded for vaccine recipients at the time of vaccination) is provided by NHS Digital’s covid-19 data store.

### Study design

This study was approved by NHS England. People were eligible for inclusion if they were aged ≥18 years on 1 July 2021 and had been registered with a primary care doctor for at least one year before eligibility for their first vaccine dose (the “eligibility date,” supplementary table S2). People were excluded if they were aged >120 years on the date at which they became eligible for vaccination; their sex, geographical region, ethnicity, or English Index of Multiple Deprivation were unknown; or they were resident in a care home or medically housebound at six weeks after their eligibility date. Full details are in supplementary figure S1.

We defined three groups who received two doses of BNT162b2, received two doses of ChAdOx1, or were unvaccinated. Eligibility for the vaccinated groups was restricted to people who received their second vaccine dose during a four week “second vaccination period” within analysis strata defined by UK Joint Committee on Vaccination and Immunisation (JCVI) priority groups (supplementary table S1), eligibility date (for priority groups within which eligibility was based on age; supplementary table S2), and English NHS region (defined using individuals’ primary care practice address).

We defined the second vaccination period as the four week period during which the greatest number of people in the stratum received their second dose. We excluded people from the vaccine groups if they received their first dose before their eligibility date, had an interval between first and second dose of less than six or more than 14 weeks, or were flagged as a healthcare worker on their vaccination record. We assigned people to the unvaccinated group if they had received no covid-19 vaccine at the start of the second vaccination period for their analysis stratum. We excluded people from any group if they had evidence of previous SARS-CoV-2 infection by the start of their second vaccination period (either a positive SARS-CoV-2 test in SGSS or probable covid-19 coded in primary care records), had ever been recorded as being resident in a care home, or had evidence of having started an end-of-life care pathway.

**Fig 1:**
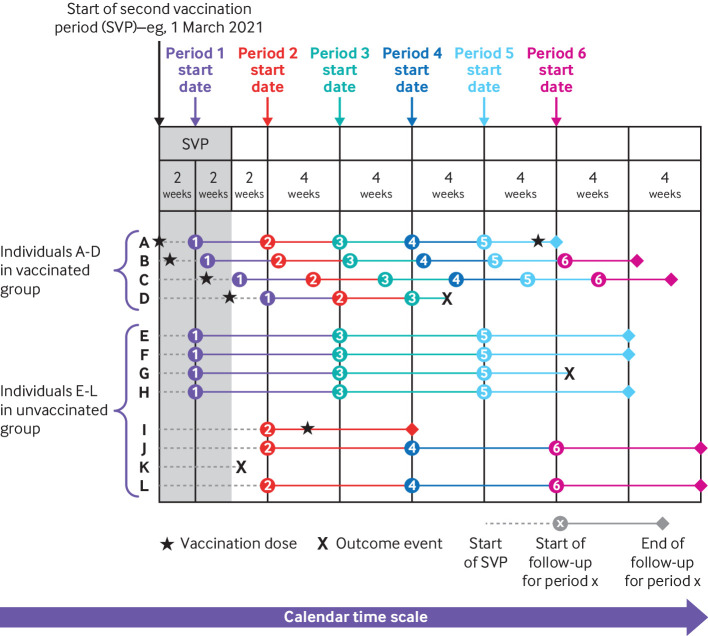
Illustrative example showing definition of four week comparison periods, within strata defined by Joint Committee on Vaccination and Immunisation group, eligibility date, and region. Horizontal lines represent follow-up time for four vaccinated and eight unvaccinated people, and colours of lines correspond to six comparison periods. Individuals A and D received their second vaccinations on first and last day of second vaccination period (SVP), respectively

Figure 1 depicts the study design. The analysis timescale was calendar time, which ensured that vaccinated and unvaccinated people were compared on the calendar day on which each outcome event occurred. We split follow-up time for fully vaccinated people into six consecutive four week “comparison periods,” starting two weeks after receipt of the second dose. Because each second vaccination period was four weeks long and each vaccinated person was followed up for four weeks per comparison period, vaccinated people were followed during eight calendar weeks in each comparison period. Vaccinated people entered and finished follow-up on the calendar dates corresponding to the start and end of their comparison period.

We followed up unvaccinated people for the full eight calendar weeks that spanned the comparison periods for vaccinated people. To avoid overlap in follow-up of unvaccinated people between comparison periods (see lines corresponding to unvaccinated individuals E-L in figure 1), follow-up time for unvaccinated people was assigned at random to start either two or six weeks after the start of the second vaccination period and was split into the three consecutive eight week calendar periods during which vaccinated people were followed in each comparison period. Unvaccinated people assigned to start at two weeks were followed during comparison periods 1, 3, and 5, and those assigned to start at six weeks were followed during comparison periods 2, 4, and 6.

### Outcomes

The outcomes were covid-19 related hospital admission (identified using Hospital Episode Statistics inpatient hospital records), covid-19 related death, positive SARS-CoV-2 test, and non-covid-19 related death. We also investigated test seeking behaviour by comparing rates of testing for SARS-CoV-2 between the vaccine groups. Covid-19 and non-covid-19 related deaths (death certificates with and without a covid-19 code) were based on death registry data from the Office for National Statistics. We identified SARS-CoV-2 tests by using SGSS records, on the basis of swab date. Both polymerase chain reaction and lateral flow tests were included, without differentiation between symptomatic and asymptomatic infection. We defined all outcomes by the date of their first occurrence during the comparison follow-up period. Where no positive SARS-CoV-2 test was present, but a record of covid-19 related hospital admission and/or death was recorded, we imputed the date of positive SARS-CoV-2 test as the date of covid-19 related hospital admission or death.

### Potential confounding factors

Models were adjusted for the following potential confounders: age, sex (male or female), English Index of Multiple Deprivation (grouped by fifths), ethnicity (black, mixed, South Asian, white, other, as per the UK census), body mass index, learning disability, serious mental illness, number of comorbid conditions in different organ systems (supplementary table S3), current pregnancy, number of SARS-CoV-2 tests between 18 May 2020 (when widespread testing became available) and eligibility for first vaccine dose, and receipt of one or more flu vaccines in the five years before the start of the second vaccination period.

### Missing data

After exclusion of participants with missing sex, geographical region, ethnicity, or English Index of Multiple Deprivation, no values were missing in the remaining variables, as they were each defined by the presence or absence of clinical codes or events in the electronic health record.

### Statistical analysis

For each comparison period, we estimated hazard ratios comparing BNT162b2 recipients versus unvaccinated people, ChAdOx1 recipients versus unvaccinated people, and BNT162b2 versus ChAdOx1 recipients. We did not estimate hazard ratios for comparison periods with fewer than three events in either group. For each person, follow-up ended at the earliest of the outcome of interest, deregistration from the primary care practice, death, or 15 December 2021. However, for SARS-CoV-2 test, follow-up ended at the “outcome of interest” only when the test result was positive. Fully vaccinated people who received a booster dose, and unvaccinated people who received a first dose were excluded from subsequent comparison periods, but follow-up within comparison periods was not censored after these events.

To estimate hazard ratios, we fitted Cox regression models with baseline hazards stratified by JCVI group, eligibility date, and region used to define the second vaccination periods, and with the covariates described above. To avoid problems with model convergence, we excluded binary covariates from the model if any cell of the table defined by cross tabulating the covariate with vaccine group and comparison period contained fewer than three events. For categorical covariates with more than two levels, levels were merged until either all levels had more than three events or only one level existed, in which case the variable was excluded. We carried out this process independently for each outcome. We modelled age within strata as linear, with quadratic terms additionally included for strata with age range >5 years.

We used meta-regression to quantify waning vaccine effectiveness as ratios of hazard ratios per comparison period. The ratios of hazard ratios can be interpreted as follows: if the hazard ratio in the three to six week comparison period is 0.5 and the ratio of hazard ratios is 1.2, then the hazard ratios will be 0.6 (=0.5×1.2), 0.72 (=0.5×1.2^2^), 0.86 (=0.5×1.2^3^), 1.04 (=0.5×1.2^4^), and 1.24 (0.5×1.2^5^) in the 7-10, 11-14, 15-18, 19-22, and 23-26 week comparison periods respectively.

We did all analyses independently in four vaccine priority subgroups: aged ≥65 years and in JCVI groups 2-5, aged 18-64 years and clinically vulnerable (JCVI groups 4 or 6), aged 40-64 years (JCVI groups 7-10; most people in this subgroup received ChAdOx1), and aged 18-39 years (JCVI groups 11-12; this subgroup received only BNT162b2). The ≥65 subgroup included participants who were clinically vulnerable, whereas the 40-64 and 18-39 subgroups did not. We fitted additional models separately in men and women to investigate effect modification by sex and in 65-74 and ≥75 years subgroups to investigate effect modification by age in older adults.

### Reporting

This study followed STROBE-RECORD reporting guidelines. All frequencies presented have been rounded up to the nearest 7 to mitigate the risk of disclosure.

### Patient and public involvement

Public contributors were not involved in setting the research question or the outcome measures, nor in developing plans for design or implementation of the study. No public contributors were asked to advise on interpretation or writing up of results. Covid-19 vaccination is offered to the whole population, including the researchers involved in the study. The main barriers to involving external public contributors were the rapidly evolving nature of the pandemic, which means that evidence quickly becomes outdated, and the urgent need to conduct and disseminate the research. OpenSAFELY invites any patient or member of the public to make contact with the project via https://opensafely.org/.

## Results

### Study population

Of 13 841 107 people satisfying initial eligibility criteria (supplementary figure S2), 5 222 812 received second doses of BNT162b2 or ChAdOx1 during the second vaccination period for their stratum (supplementary figures S3-18) and 2 575 111 were unvaccinated at the start of their second vaccination period. Of these, 1 951 866, 3 219 349, and 2 422 980 were eligible for inclusion in the first comparison period for BNT162b2, ChAdOx1, and unvaccinated groups, respectively. Table 1 and supplementary table S4 show summary statistics for these three groups by subgroup. Compared with vaccinated people, unvaccinated people were less likely to be white, to live in a more affluent area, to have had a flu vaccine in the previous five years, or to have tested for SARS-CoV-2 before their eligibility date. The distribution of other characteristics between vaccine groups differed according to subgroup. For example, in the ≥65 subgroup, those vaccinated with BNT162b2 were older than those vaccinated with ChAdOx1 (75 (interquartile range 72-80) years) versus 72 (70-75) years, whereas the converse was true in the 40-64 subgroup (44 (41-50) years versus 55 (50-59) years).

**Table 1:** Summary statistics for selected characteristics across subgroups and vaccination groups. Values are numbers (percentages) unless stated otherwise

Characteristic	≥65 years				18-64 years, clinically vulnerable				40-64 years*				18-39 years*	
	BNT162b2 (n=830 662)	ChAdOx1 (n=1 096 872)	Unvaccinated (n=144 774)		BNT162b2 (n=368 732)	ChAdOx1 (n=649 075)	Unvaccinated (n=300 881)		BNT162b2 (n=63 021)	ChAdOx1 (n=1 473 402)	Unvaccinated (n=625 226)		BNT162b2 (n=689 451)	Unvaccinated (n=1 352 099)
Median (IQR) age, years	75 (72-80)	72 (70-75)	72 (68-78)		54 (45-60)	52 (42-59)	40 (32-52)		44 (41-50)	55 (50-59)	48 (42-54)		30 (24-35)	30 (25-34)
Female sex	440 195 (53)	568 498 (52)	77 553 (54)		179 165 (49)	329 749 (51)	154 693 (51)		31 976 (51)	692 202 (47)	257 824 (41)		336 770 (49)	607 138 (45)
IMD†:														
1	99 680 (12)	139 160 (13)	35 021 (24)		81 578 (22)	146 188 (23)	113 050 (38)		9275 (15)	191 310 (13)	176 484 (28)		113 421 (16)	427 392 (32)
2	138 250 (17)	186 025 (17)	32 326 (22)		75 943 (21)	137 543 (21)	72 828 (24)		11 312 (18)	256 102 (17)	148 106 (24)		140 581 (20)	337 365 (25)
3	187 467 (23)	249 767 (23)	30 730 (21)		79 009 (21)	136 934 (21)	53 228 (18)		13 545 (21)	327 215 (22)	128 051 (20)		155 085 (22)	266 525 (20)
4	200 088 (24)	259 350 (24)	26 376 (18)		70 644 (19)	120 386 (19)	37 254 (12)		14 448 (23)	345 324 (23)	101 745 (16)		145 488 (21)	193 256 (14)
5	205 191 (25)	262 584 (24)	20 328 (14)		61 565 (17)	108 038 (17)	24 542 (8)		14 448 (23)	353 465 (24)	70 847 (11)		134 897 (20)	127 561 (9)
Ethnicity:														
White	801 836 (97)	1 058 288 (96)	113 988 (79)		335 153 (91)	574 959 (89)	217 014 (72)		53 522 (85)	1 362 669 (92)	460 131 (74)		595 714 (86)	970 130 (72)
Black	3997 (0)	4921 (0)	8316 (6)		5327 (1)	12 173 (2)	24 073 (8)		1162 (2)	18 179 (1)	42 273 (7)		8974 (1)	69 216 (5)
South Asian	17 528 (2)	23 534 (2)	14 259 (10)		20 636 (6)	46 067 (7)	39 536 (13)		5523 (9)	57 393 (4)	70 875 (11)		54 971 (8)	168 441 (12)
Mixed	2478 (0)	3283 (0)	2604 (2)		3451 (1)	6909 (1)	9380 (3)		903 (1)	12 180 (1)	17 332 (3)		11 592 (2)	44 674 (3)
Other	4837 (1)	6860 (1)	5614 (4)		4172 (1)	8974 (1)	10 892 (4)		1925 (3)	22 995 (2)	34 622 (6)		18 221 (3)	99 659 (7)
Body mass index:														
<30	631 918 (76)	812 364 (74)	117 229 (81)		199 948 (54)	371 931 (57)	203 644 (68)		53 788 (85)	1 222 242 (83)	556 997 (89)		618 226 (90)	1 256 815 (93)
≥40	18 956 (2)	30 303 (3)	3206 (2)		59 941 (16)	100 324 (15)	40 047 (13)		140 (0)	2828 (0)	952 (0)		1722 (0)	2415 (0)
30-34.9	135 135 (16)	187 236 (17)	17 829 (12)		70 469 (19)	115 241 (18)	38 122 (13)		6741 (11)	184 093 (12)	50 001 (8)		48 650 (7)	66 864 (5)
35-39.9	44 667 (5)	66 976 (6)	6517 (5)		38 388 (10)	61 593 (9)	19 082 (6)		2359 (4)	64 246 (4)	17 283 (3)		20 860 (3)	26 012 (2)
Morbidity count:														
0	342 160 (41)	530 530 (48)	79 520 (55)		59 465 (16)	121 310 (19)	78 120 (26)		62 601 (99)	1 464 155 (99)	621 607 (99)		687 477 (100)	1 344 497 (99)
1	273 301 (33)	344 561 (31)	39 900 (28)		237 048 (64)	417 823 (64)	192 528 (64)		413 (1)	9093 (1)	3542 (1)		1960 (0)	7518 (1)
≥2	215 201 (26)	221 781 (20)	25 361 (18)		72 226 (20)	109 949 (17)	30 240 (10)		14 (0)	161 (0)	84 (0)		21 (0)	84 (0)
No of SARS-CoV-2 tests‡:														
0	704 123 (85)	934 269 (85)	130 760 (90)		258 853 (70)	447 643 (69)	229 250 (76)		44 709 (71)	1 063 391 (72)	534 002 (85)		370 146 (54)	1 017 667 (75)
1	83 468 (10)	109 606 (10)	8274 (6)		68 971 (19)	124 649 (19)	41 923 (14)		11 207 (18)	253 652 (17)	54 978 (9)		145 691 (21)	177 401 (13)
2	24 038 (3)	29 715 (3)	2632 (2)		22 925 (6)	42 336 (7)	14 455 (5)		3493 (6)	73 920 (5)	16 618 (3)		63 686 (9)	71 057 (5)
≥3	19 040 (2)	23 289 (2)	3115 (2)		17 997 (5)	34 461 (5)	15 267 (5)		3619 (6)	82 446 (6)	19 628 (3)		109 942 (16)	85 981 (6)
Flu vaccine	761 551 (92)	982 961 (90)	54 768 (38)		280 364 (76)	456 519 (70)	88 172 (29)		14 994 (24)	588 805 (40)	34 153 (5)		104 531 (15)	107 240 (8)

IMD=Index of Multiple Deprivation; IQR=interquartile range.

* Not clinically vulnerable.

† 1=most deprived.

‡ Number of tests in between 18 May 2020 (when widespread testing became available) and first eligibility date for subgroup.

### Attrition due to subsequent vaccination

The cumulative incidence of first vaccine dose by week 23 in previously unvaccinated people was 16%, 28%, 13%, and 14% in the ≥65, 18-64 and clinically vulnerable, 40-64, and 18-39 subgroups respectively (supplementary figure S19). The UK vaccination programme initially offered third doses only after six months (23-26 weeks) since the second dose^21^; however, among the 18-39 subgroup, 35% and 26% of those in the BNT162b2 and ChAdOx1 groups, respectively, had received a third dose by week 20, because the required time since second dose was reduced to three months in early December 2021 owing to concerns about the omicron variant.^22^ ^23^

### Distribution of follow-up time

Supplementary figures S20-23 show the distribution of follow-up by calendar time in each comparison period, for each subgroup, relative to dates during which different variants were dominant. Follow-up in the ≥65 subgroup began on 15 March 2021 (when the alpha variant was dominant) and ended on 30 November 2021. Follow-up for the 18-64 and clinically vulnerable, 40-64, and 18-39 subgroups began on 21 April, 18 May, and 23 July 2021, respectively: the delta variant was dominant in England by 1 June 2021. The latest follow-up was on 15 December 2021; 54% of specimens sampled in England on this date had S-gene target failure, which suggests that the omicron variant became dominant in England by this date.^24^

### Waning vaccine effectiveness

Unadjusted and adjusted hazard ratios are shown in supplementary tables S6 and S7, respectively, and compared in supplementary figures S24-29, with adjusted hazard ratios for each covariate shown in supplementary tables S9-50. For the models comparing people fully vaccinated with BNT162b2 and ChAdOx1 with unvaccinated people, the unadjusted and adjusted hazard ratios were generally similar. Where they differed, patterns were outcome and subgroup specific. Unadjusted and adjusted hazard ratios were similar for comparisons of BNT162b2 with ChAdOx1.

**Fig 2:**
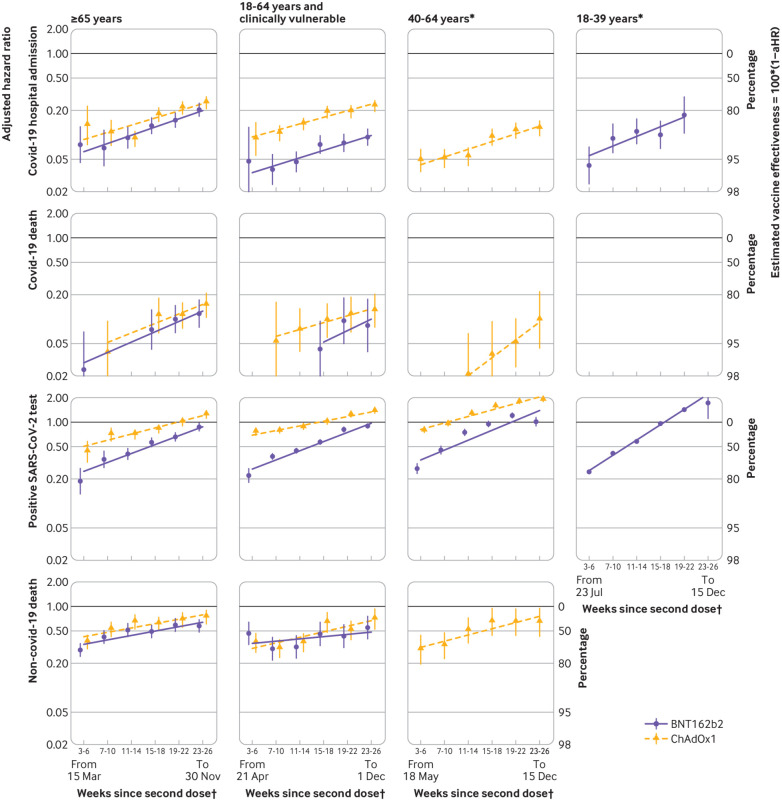
Adjusted hazard ratios (aHRs) comparing BNT162b2 and ChAdOx1 recipients with unvaccinated people in each comparison period. Estimates for BNT162b2 in 40-64 age group are omitted for all outcomes except positive SARS-CoV-2 test owing to low event counts. Slopes of lines are ratios of hazard ratios across comparison periods, fitted using meta-regression. Y axis is on log scale. *Not clinically vulnerable. †Dates (all in 2021) represent earliest and latest dates of follow-up within subgroup

Figure 2 shows adjusted hazard ratios (with 95% confidence intervals) for BNT162b2 or ChAdOx1 vaccination versus no vaccination across the six comparison periods. The slopes of the lines correspond to ratios of adjusted hazard ratios per comparison period (also shown in supplementary table S8). The missing adjusted hazard ratios could not be estimated because too few events occurred in one or both groups (supplementary table S5).

#### Covid-19 related hospital admissions

We observed 2100, 5369, and 9443 covid-19 related hospital admissions in BNT162b2 recipients, ChAdOx1 recipients, and unvaccinated people, respectively (supplementary table S5). Estimated adjusted hazard ratios comparing BNT162b2 and ChAdOx1 recipients with unvaccinated people in the ≥65 subgroup were 0.08 (95% confidence interval 0.05 to 0.13) and 0.13 (0.08 to 0.23), respectively, during weeks three to six after the second dose, waning to 0.20 (0.17 to 0.25) and 0.25 (0.21 to 0.29), respectively, during weeks 23-26 (fig 2). The ratios of adjusted hazard ratios per period were similar for BNT162b2 and ChAdOx1: 1.26 (1.17 to 1.36) and 1.23 (1.07 to 1.40), respectively. Estimated adjusted hazard ratios comparing BNT162b2 and ChAdOx1 recipients with unvaccinated people in the 18-64 and clinically vulnerable subgroup were 0.05 (0.02 to 0.12) and 0.09 (0.06 to 0.14), respectively, during weeks three to six, waning to 0.09 (0.07 to 0.12) and 0.23 (0.19 to 0.26), respectively, by weeks 23-26. The ratios of adjusted hazard ratios per period were 1.23 (1.13 to 1.35) and 1.20 (1.14 to 1.27) for BNT162b2 and ChAdOx1, respectively. Estimated adjusted hazard ratios comparing ChAdOx1 recipients with unvaccinated people in the 40-64 subgroup waned from 0.05 (0.04 to 0.07) during weeks three to six to 0.12 (0.10 to 0.15) during weeks 23-26 (ratio of adjusted hazard ratios 1.24 (1.16 to 1.33) per period). Adjusted hazard ratios for BNT162b2 could not be estimated in the 40-64 subgroup because too few covid-19 related hospital admissions occurred. Estimated adjusted hazard ratios comparing BNT162b2 recipients with unvaccinated people in the 18-39 subgroup waned from 0.04 (0.02 to 0.07) during weeks three to six to 0.18 (0.10 to 0.29) during weeks 19-22 (ratio of adjusted hazard ratios 1.31 (1.10 to 1.57) per period).

#### Covid-19 related deaths

We observed 329, 595, and 889 covid-19 related deaths in BNT162b2 recipients, ChAdOx1 recipients, and unvaccinated people, respectively (supplementary table S5). In the ≥65 subgroup, estimated adjusted hazard ratios comparing BNT162b2 recipients with unvaccinated people were 0.02 (0.01 to 0.07) during weeks three to six after the second dose and those comparing ChAdOx1 recipients with unvaccinated people were 0.04 (0.02 to 0.09) during weeks seven to 10 after the second dose. These estimates waned to 0.12 (0.08 to 0.17) and 0.15 (0.11 to 0.21) for BNT162b2 and ChAdOx1, respectively, by weeks 23-26 (fig 2). The ratios of adjusted hazard ratios per period were 1.34 (1.09 to 1.64) and 1.31 (1.07 to 1.61) for BNT162b2 and ChAdOx1, respectively. In the 18-64 and clinically vulnerable subgroup, estimated adjusted hazard ratios comparing BNT162b2 recipients with unvaccinated people were 0.04 (0.02 to 0.09) during weeks 15-18 after the second dose and those comparing ChAdOx1 recipients with unvaccinated people were 0.05 (0.02 to 0.16) during weeks seven to 10 after the second dose. These estimates waned to 0.08 (0.04 to 0.18) and 0.13 (0.08 to 0.20) for BNT162b2 and ChAdOx1, respectively, by weeks 23-26. The ratios of adjusted hazard ratios per period were 1.38 (0.77 to 2.47) and 1.21 (0.99 to 1.49) for BNT162b2 and ChAdOx1, respectively. Estimated adjusted hazard ratios comparing ChAdOx1 recipients with unvaccinated people in the 40-64 subgroup waned from 0.02 (0.01 to 0.07) during weeks 11-14 to 0.10 (0.04 to 0.22) during weeks 23-26 (ratio of adjusted hazard ratios 1.66 (1.08 to 2.55) per period). Too few covid-19 related deaths occurred in the 18-39 subgroup to allow estimation of hazard ratios.

#### Positive SARS-CoV-2 tests

We observed 62 363, 186 137, and 118 216 positive SARS-CoV-2 tests in BNT162b2 recipients, ChAdOx1 recipients, and unvaccinated people, respectively (supplementary table S5). For BNT162b2 recipients compared with unvaccinated people, estimated adjusted hazard ratios across subgroups ranged from 0.19 (0.13 to 0.27) to 0.27 (0.23 to 0.31) during weeks three to six and from 0.87 (0.78-0.97) to 1.72 (1.11 to 2.68) during weeks 23-26 (fig 2; supplementary table S7). For ChAdOx1 recipients compared with unvaccinated people, estimated adjusted hazard ratios ranged across subgroups from 0.43 (0.32 to 0.58) to 0.78 (0.75 to 0.81) and waned to 1.23 (1.11 to 1.36), 1.35 (1.29 to 1.41), and 1.86 (1.79 to 1.93) in the ≥65, 18-64 and clinically vulnerable, and 40-64 subgroups, respectively. For BNT162b2, ratios of hazard ratios were 1.29 (1.23 to 1.35), 1.30 (1.23 to 1.36), 1.32 (1.17 to 1.49), and 1.54 (1.50 to 1.59) in the ≥65, 18-64 and clinically vulnerable, 40-64, and 18-39 subgroups, respectively (supplementary table S8). For ChAdOx1, ratios of hazard ratios were 1.19 (1.14 to 1.24), 1.14 (1.11 to 1.17), and 1.20 (1.16 to 1.25) in the ≥65, 18-64 and clinically vulnerable, and 40-64 subgroups, respectively.

#### Non-covid-19 related deaths

We observed 8463, 9135, and 3031 non-covid-19 related deaths in BNT162b2 recipients, ChAdOx1 recipients, and unvaccinated people, respectively (supplementary table S5). Across subgroups, estimated adjusted hazard ratios during weeks three to six ranged from 0.29 (0.24 to 0.35) to 0.47 (0.34 to 0.64) for BNT162b2 and from 0.29 (0.20 to 0.44) to 0.36 (0.30 to 0.44) for ChAdOx1 (fig 2; supplementary table S7). By weeks 23-26, these ranged from 0.55 (0.40 to 0.76) to 0.58 (0.48 to 0.69) for BNT162b2 and from 0.64 (0.43 to 0.95) to 0.74 (0.61 to 0.90) for ChAdOx1. Rates of waning were lower than for the other outcomes (maximum ratio of adjusted hazard ratios 1.19 (1.09 to 1.31); supplementary table S8).

#### SARS-CoV-2 tests

We observed 1 406 181, 2 907 849, and 674 534 SARS-CoV-2 tests in BNT162b2 recipients, ChAdOx1 recipients, and unvaccinated people, respectively (only the first test in each comparison period counted; supplementary table S5). Across subgroups, rates of testing during weeks three to six were higher in vaccinated people than in unvaccinated people; adjusted hazard ratios ranged from 1.55 (1.50 to 1.61) to 2.09 (2.03 to 2.15) for BNT162b2 and from 2.01 (1.95 to 2.08) to 2.79 (2.75 to 2.83) for ChAdOx1 (supplementary figure S30; supplementary tables S7 and S8). The discrepancy in testing behaviour between the vaccinated and unvaccinated people increased slightly by weeks 23-26; adjusted hazard ratios ranged from 2.06 (2.00 to 2.13) to 3.06 (2.66 to 3.53) for BNT162b2 and from 2.31 (2.27 to 2.36) to 3.70 (3.64 to 3.76) for ChAdOx1.

#### Comparative effectiveness

**Fig 3:**
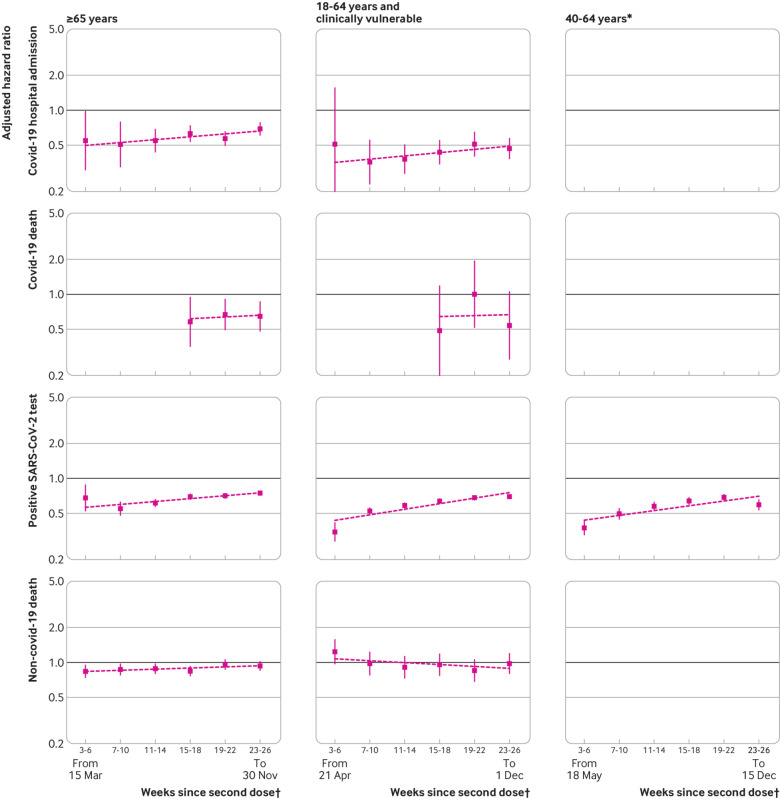
Adjusted hazard ratios comparing BNT162b2 with ChAdOx1. Hazard ratios <1 favour BNT162b2. Slopes of lines correspond to ratios of hazard ratios across comparison periods, estimated using meta-regression. Estimates in 40-64 years age group are omitted for all outcomes except positive SARS-CoV-2 test owing to low event counts. Y axis is on log scale. *Not clinically vulnerable. †Dates (all in 2021) represent earliest and latest dates of follow-up within subgroup

Estimated adjusted hazard ratios comparing BNT162b2 with ChAdOx1 recipients consistently favoured BNT162b2 (fig 3; supplementary table S7; ratios of adjusted hazard ratios in supplementary table S8). In the ≥65 and 18-64 and clinically vulnerable subgroups, estimated adjusted hazard ratios were 0.51 (0.33 to 0.79) and 0.36 (0.23 to 0.55), respectively, for covid-19 related hospital admission during weeks seven to 10; 0.58 (0.36 to 0.94) and 0.49 (0.20 to 1.18), respectively, for covid-19 related death during weeks 15-18; and 0.68 (0.52 to 0.88) and 0.35 (0.29 to 0.41), respectively, for positive SARS-CoV-2 test during weeks three to six. Because the rate of waning was slightly higher for BNT162b2 than ChAdOx1, the adjusted hazard ratios for both the ≥65 and 18-64 and clinically vulnerable subgroups were attenuated by weeks 23-26, but they still favoured BNT162b2 over ChAdOx1 (0.69 (0.61 to 0.78) and 0.47 (0.38 to 0.57), respectively, for covid-19 related hospital admission; 0.65 (0.48 to 0.86) and 0.54 (0.28 to 1.05), respectively, for covid-19 related death; and 0.75 (0.72 to 0.78) and 0.70 (0.67 to 0.72), respectively, for positive SARS-CoV-2 test).

#### Effect modification by sex and by age in older adults

We observed no consistent differences between vaccine effectiveness in men and women. For ChAdOx1, adjusted hazard ratios seemed to be broadly similar in men and women. For BNT162b2, vaccine effectiveness against covid-19 related hospital admission seemed to be somewhat greater in women than in men in the ≥65 subgroup but greater in men than in women in the 18-39 subgroup (supplementary tables S51-58; supplementary figures S32-35).

For both BNT162b2 and ChAdOx1 recipients, estimated vaccine effectiveness against covid-19 related hospital admission and covid-19 related death was greater in those aged 65-74 years than in those ≥75 years during weeks three to 14, and then broadly similar in these two age groups during weeks 15-26. Estimated vaccine effectiveness against a positive SARS-CoV-2 test was generally lower in people aged 65-74 years than ≥75 years (supplementary tables S59-62; supplementary figures S36-39).

## Discussion

This cohort study estimated the effectiveness and comparative effectiveness of the BNT162b2 and ChAdOx1 covid-19 vaccines during six consecutive periods, each of four weeks’ duration, starting two weeks after receipt of second dose. Rates of covid-19 related hospital admission and covid-19 related death were consistently and substantially lower among fully vaccinated people compared with those who remained unvaccinated, up to 26 weeks after the second vaccination, and consistently lower among people fully vaccinated with BNT162b2 than with ChAdOx1. However, by 23-26 weeks, rates of positive SARS-CoV-2 test (ascertained through freely available national routine testing) in fully vaccinated people were similar to or higher than those in unvaccinated people. Rates of non-covid-19 related death were consistently lower among fully vaccinated than unvaccinated people.

When quantified as ratios of adjusted hazard ratios, estimated waning of vaccine effectiveness was strikingly similar across risk groups, except that waning was fastest in the 18-39 year subgroup (those at lowest risk of severe covid-19, all vaccinated with BNT162b2). In those subgroups in which the two vaccines could be compared, estimated vaccine effectiveness was consistently greater for BNT162b2 than for ChAdOx1, but waning was somewhat faster for BNT162b2 than ChAdOx1, so that the two brands’ comparative effectiveness became more similar over time. Estimated adjusted hazard ratios for covid-19 related hospital admission and covid-19 related death remained ≤0.20 (≥80% vaccine effectiveness) for BNT162b2 and ≤0.25 (≥75% vaccine effectiveness) for ChAdOx1 during weeks 23-26 after the second vaccination.

### Findings in context

A systematic review and meta-regression of the duration of effectiveness of covid-19 vaccines included 18 studies, most of which evaluated BNT162b2 or Moderna’s mRNA-1273 vaccine.^13^ Estimates of vaccine effectiveness and the duration of vaccine effectiveness varied substantially between studies. Only two studies found minimal vaccine effectiveness against SARS-CoV-2 infection by six months, and none found decreases in vaccine effectiveness against positive SARS-CoV-2 test as large as those reported in our study.^9^ ^14^ The meta-regression estimated an average decrease in vaccine effectiveness from one to six months after full vaccination across studies of 9.5 (95% confidence interval 5.7 to 14.6) percentage points for severe covid-19 (hospital admission or death due to covid-19), and of 20.7 (10.2 to 36.6) percentage points for SARS-CoV-2 infection. The corresponding reductions in the ≥65 subgroup in our study were: covid-19 related hospital admission 12.8 (7.41 to 18.2) for BNT162b2 and 11.7 (3.44 to 20.0) for ChAdOx1; positive SARS-CoV-2 test 68.2 (56.4 to 80.0) for BNT162b2 and 79.6 (62.0 to 97.2) for ChAdOx1. However, the ratios of adjusted hazard ratios in the ≥65 subgroup in our study were similar for covid-19 related hospital admission (1.26 (1.17 to 1.36) for BNT162b2 and 1.23 (1.07 to 1.40) for ChAdOx1) and for positive SARS-CoV-2 test (1.29 (1.23 to 1.35) for BNT162b2 and 1.19 (1.14 to 1.24) for ChAdOx1). Thus, the metric used to quantify waning vaccine effectiveness can lead to strikingly different conclusions about its magnitude. The review concluded that the decline in vaccine effectiveness against severe covid-19 was less than for SARS-CoV-2 infection and symptomatic disease. However, by quantifying waning in terms of ratios of hazard ratios, we found that rates of waning were similar for these two outcomes (supplementary table S8).

As in our study, Andrews and colleagues analysed NHS England electronic health records data to investigate the duration of protection by covid-19 vaccines against symptomatic and severe covid-19.^5^ They used a test negative case-control design, which aims to reduce confounding due to health seeking behaviour by considering only people who were tested for SARS-CoV-2 infection.^25^ However, this restriction to the subpopulation of people who are tested if they have symptoms has potential biases. For example, if older people were more likely to seek testing when they had symptoms then vaccine effectiveness in the tested subpopulation may differ from that in the general population.^20^ The restriction to those tested can also induce so-called “collider bias,” because associations between causes of being tested are distorted in the tested subpopulation.^26^

Comparisons between this study and that of Andrews and colleagues are restricted to the ≥65 and 40-64 subgroups, as these are identically defined in the two studies, and to the 15-18 weeks comparison period in this study and the 15-19 weeks since second dose in the other study, which were the most similar across the two studies. Compared with Andrews and colleagues, we consistently estimated lower effectiveness for both vaccines against covid-19 related hospital admission (differences ranged from 1.3 to 6.4 percentage points). Andrews and colleagues concluded (by contrast with our study) that waning was greater for ChAdOx1 than BNT162b2 and greater among older adults and those in a clinical risk group. Whereas those authors found continuing vaccine effectiveness against symptomatic covid-19 up to 26 weeks after receipt of the second vaccine dose, we found rates of positive SARS-CoV-2 test to be similar to or higher than those in unvaccinated participants by that time.

Most of the follow-up in our study was while the delta variant was dominant in England. Previous studies found vaccine effectiveness to be lower for the delta variant than the alpha variant,^3^ ^18^ ^27^ and lower for the omicron variant than the delta variant.^28^ ^29^ Consistent with the findings of this study, infection in vaccinated people has been widespread since the omicron variant became dominant; an estimated 71% of people in England had been infected with SARS-CoV-2 by 22 April 2022.^30^ It will be important to extend the analyses reported in this study to examine longer term effectiveness of two dose vaccination, and waning effectiveness of booster vaccination, against the omicron variant.

### Strengths and limitations of this study

Our study is based on whole population data analysed within the OpenSAFELY Trusted Research Environment, which has stringent disclosure controls to protect patients’ privacy. The large study size and large numbers of outcome events led to precise estimates of vaccine effectiveness according to brand of vaccine and time since second vaccine dose. We accounted for risk dependent vaccine allocation by separating the cohort into subgroups based on JCVI group,^31^ and by doing analyses within strata defined by JCVI group, eligibility date for primary vaccination, and geographical region. We fully accounted for rapid changes in incidence of covid-19 with calendar time, because the risk sets underpinning the Cox models compared people being followed on the calendar day when outcome events occurred. Our analyses also excluded people with a pre-vaccine rollout record of SARS-CoV-2 infection and accounted for censoring due to occurrence of outcome events and attenuation of comparison groups because of receipt of first vaccine dose by unvaccinated people and third dose by fully vaccinated people. The OpenSAFELY platform contains an unprecedented scale of NHS records that are refreshed every week, and the methods used for data storage and management facilitate rapid re-execution of all curation and analysis code. This will enable us to regularly reassess waning vaccine effectiveness over extended time intervals, the impact of new SARS-CoV-2 variants, and the effects of further doses of covid-19 vaccines.

Our study has several limitations. Firstly, as in any observational study, our estimates could be affected by confounding by unmeasured factors. The detailed linked data analysed permitted adjustment for a wide range of potential confounding factors, but we may not have been able to control completely for the associations of being vaccinated with health seeking behaviours and being less socioeconomically deprived. Secondly, patients registered with a primary care practice who have moved or emigrated (or whose death was not recorded)^32^ may contribute person time but not events. Because the BNT162b2 and ChAdOx1 groups are defined by recent vaccination, these “ghost” patients are more likely to be present in the unvaccinated group, leading to bias in estimates of waning. Also, healthcare workers could be identified and excluded from the vaccinated groups, because this information was recorded at the time of vaccination, but not from the unvaccinated group. This limitation should not affect results for the ≥65 subgroup, most of whom are retired, or comparisons between BNT162b2 and ChAdOx1. Thirdly, consistent with an Australian survey,^33^ we found that unvaccinated people had tested less frequently than vaccinated people during the pre-vaccine rollout period when widespread testing was available (table 1) and were considerably less likely to be tested during follow-up (supplementary table S7). Fourthly, differential depletion of susceptible people in the unvaccinated groups over time may lead to attenuation of hazard ratios even when true vaccine effectiveness does not change. However, such bias is likely to be minimal when vaccine effectiveness is high.^34^ Fifthly, we excluded people vaccinated outside defined “second vaccination periods” and estimated hazard ratios within four week periods subsequent to second vaccination. Modelling non-linear interactions of vaccine effectiveness with time since vaccination is a potentially more powerful approach that could avoid excluding so many people but would rely on more complex modelling assumptions than those underpinning our analyses.

### Conclusions

When quantified as ratios of hazard ratios, the rate at which estimated vaccine effectiveness waned was strikingly consistent (we saw little variation around the fitted rates of waning shown in figure 2) and (by contrast with other studies) similar across subgroups defined by age and clinical vulnerability. If sustained to outcomes of infection with the omicron variant and to booster vaccination, these findings will facilitate scheduling of booster vaccination doses. By 26 weeks after the second dose, rates of positive SARS-CoV-2 test in fully vaccinated people were similar to or higher than those in unvaccinated people, implying that vaccination has only transient effects on transmissibility of SARS-CoV-2 and emphasising the desirability of development of new vaccines that inhibit transmission.^35^ These findings may result partly from greater social mixing by vaccinated than unvaccinated people based on their substantially reduced risk of severe covid-19. The reduction and ultimate removal of restrictions on social mixing is a crucial benefit of covid-19 vaccination. Protection against covid-19 related hospital admission and death was substantial up to 26 weeks after the second vaccination, even in older and clinically vulnerable people. Finally, cessation of freely available population based testing programmes is likely to limit applications of the test negative case-control study design, which has to date provided rapid estimates of vaccine effectiveness. By contrast, cohort approaches based on detailed linked electronic health record data, such as were used in this study, will remain feasible for severe covid-19 outcomes.

## What is already known on this topic

A recent systematic review estimated that vaccine effectiveness against severe covid-19 decreased by 10 (95% confidence interval 6.1 to 15.4) percentage points from one to six months after full vaccination
Estimated vaccine effectiveness against SARS-CoV-2 infection decreased by 21 (13.9 to 29.8) percentage points over the same period
However, differences in study design and substantial differences between findings from different studies limited the conclusions that could be made

## What this study adds

Waning vaccine effectiveness quantified as ratios of hazard ratios per four week period were strikingly consistent for covid-19 related hospital admission, covid-19 related death, and positive SARS-CoV-2 test
Strong protection against covid-19 related hospital admission and death persisted up to 26 weeks after second vaccine dose
This cohort study design is an alternative to the test negative case-control design, which has become less feasible with the cessation of freely available population based testing programmes

## Data Availability

Data management and analyses were conducted in Python version 3.8.10 and R version 4.0.2. All data were linked, stored, and analysed securely within the OpenSAFELY platform (https://opensafely.org/). Data include pseudonymised data such as coded diagnoses, medications, and physiological parameters. No free text data are included. All code is shared openly for review and re-use under MIT open license (https://github.com/opensafely/covid-ve-change-over-time). Detailed pseudonymised patient data are potentially re-identifiable and therefore not shared. Codelists are available at https://www.opencodelists.org/.

## Web extra

Extra material supplied by authors

Web appendix: Supplementary materials

## Notes

Contributors: JACS and MAH conceived the study. JACS, MAH, EMFH, WJH, RK, TP, and EJW designed the study. EMFH, WJH, SB, ID, and BG contributed to data curation. EMFH, WJH, and JACS did the statistical analysis. JACS and BG acquired funding for the study. WJH, JM, AM, and BG contributed to project administration. SB and ID contributed resources for the study. SB, DE, PI, CEM, GH, SD, TW, and ID created and maintained software. JACS, WJH, JM, AM, and BG supervised the study. AM and BG were responsible for work relating to information governance. EMFH and JACS wrote the first draft of the manuscript. All authors contributed to reviewing and editing of the manuscript. All authors had final responsibility for the decision to submit for publication. JACS is the guarantor. The corresponding author attests that all listed authors meet authorship criteria and that no others meeting the criteria have been omitted.

Information governance: Patient data have been pseudonymised for analysis and linkage using industry standard cryptographic hashing techniques; all pseudonymised datasets transmitted for linkage onto OpenSAFELY are encrypted; access to the platform is via a virtual private network connection, restricted to a small group of researchers; the researchers hold contracts with NHS England and access the platform only to initiate database queries and statistical models; all database activity is logged; only aggregate statistical outputs leave the platform environment following best practice for anonymisation of results such as statistical disclosure control for low cell counts.^37^ The OpenSAFELY research platform adheres to the obligations of the UK General Data Protection Regulation and the Data Protection Act 2018. In March 2020 the Secretary of State for Health and Social Care used powers under the UK Health Service (Control of Patient Information) Regulations 2002 to require organisations to process confidential patient information for the purposes of protecting public health, providing healthcare services to the public, and monitoring and managing the covid-19 outbreak and incidents of exposure; this sets aside the requirement for patient consent.^38^ Taken together, these provide the legal basis to link patient datasets on the OpenSAFELY platform. Primary care practices, from which the primary care data are obtained, are required to share relevant health information to support the public health response to the pandemic and have been informed of the OpenSAFELY analytics platform.

Dissemination to participants and related patient and public communities: We plan to present our findings at national and international conferences. The results of the research will be disseminated to study participants (the whole population) through press releases, lay summaries on study websites, and discussion with groups of patients and the public advising the covid-19 National Core Studies. We have developed a publicly available website (https://opensafely.org/) through which we invite any patient or member of the public to contact us regarding this study or the broader OpenSAFELY project.

Provenance and peer review: Not commissioned; externally peer reviewed.
