# Supplementary material for: Waning effectiveness of BNT162b2 and ChAdOx1 covid-19 vaccines over six months since second dose: OpenSAFELY cohort study using linked electronic health records
Source: BMJ. 2022 Jul 20;378:e071249. doi: 10.1136/bmj-2022-071249 (PMC10441183; doi:10.1136/bmj-2022-071249)
Supplement: Supplementary file 1 — Web appendix: Supplementary materials [file hore071249.ww1.pdf]

Contents

List of Tables 1

List of Figures 1

Supplementary Methods 2

Supplementary Results 8

Study population . . . 8

Attrition due to subsequent vaccination . . . 18

Distribution of follow-up time . . . 19

Waning vaccine effectiveness in risk-based subgroups . . . 22

Waning vaccine effectiveness in risk- and sex-based subgroups . . . 78

Waning vaccine effectiveness in older adults . . . 91

List of Tables

1 Vaccination phases and priority groups for primary vaccination advised by the Joint Committee on Vaccination and Immunisation . . . 2

2 Eligibility dates for first vaccine dose . . . 2

3 Variable definitions . . . 4

4 Additional characteristics summarised across subgroups. . . . 17

5 Event counts / person-years. . . . 23

6 Unadjusted hazard ratios for effect of vaccination. . . . 24

7 Adjusted hazard ratios for effect of vaccination. . . . 25

8 Per-comparison-period ratio of adjusted hazard ratios for effect of vaccination. . . . 26

9 Covariate hazard ratios for COVID-19 hospitalisation in the BNT162b2 vs unvaccinated comparison and the 65+ years subgroup. . . . 35

10 Covariate hazard ratios for COVID-19 death in the BNT162b2 vs unvaccinated comparison and the 65+ years subgroup. 36

11 Covariate hazard ratios for Positive SARS-CoV-2 test in the BNT162b2 vs unvaccinated comparison and the 65+ years subgroup. . . . 37

12 Covariate hazard ratios for Non-COVID-19 death in the BNT162b2 vs unvaccinated comparison and the 65+ years subgroup. . . . 38

13 Covariate hazard ratios for Any SARS-CoV-2 test in the BNT162b2 vs unvaccinated comparison and the 65+ years subgroup. . . . 39

14 Covariate hazard ratios for COVID-19 hospitalisation in the ChAdOx1 vs unvaccinated comparison and the 65+ years subgroup. . . . 40

15 Covariate hazard ratios for COVID-19 death in the ChAdOx1 vs unvaccinated comparison and the 65+ years subgroup. 41

16 Covariate hazard ratios for Positive SARS-CoV-2 test in the ChAdOx1 vs unvaccinated comparison and the 65+ years subgroup. . . . 42

17 Covariate hazard ratios for Non-COVID-19 death in the ChAdOx1 vs unvaccinated comparison and the 65+ years subgroup. . . . 43

18 Covariate hazard ratios for Any SARS-CoV-2 test in the ChAdOx1 vs unvaccinated comparison and the 65+ years subgroup. . . . 44

19 Covariate hazard ratios for COVID-19 hospitalisation in the BNT162b2 vs ChAdOx1 comparison and the 65+ years subgroup. . . . 45
20 Covariate hazard ratios for COVID-19 death in the BNT162b2 vs ChAdOx1 comparison and the 65+ years subgroup. 46
21 Covariate hazard ratios for Positive SARS-CoV-2 test in the BNT162b2 vs ChAdOx1 comparison and the 65+ years subgroup. . . . 47
22 Covariate hazard ratios for Non-COVID-19 death in the BNT162b2 vs ChAdOx1 comparison and the 65+ years subgroup. . . . 48
23 Covariate hazard ratios for Any SARS-CoV-2 test in the BNT162b2 vs ChAdOx1 comparison and the 65+ years subgroup. . . . 49
24 Covariate hazard ratios for COVID-19 hospitalisation in the BNT162b2 vs unvaccinated comparison and the 18-64 years and clinically vulnerable subgroup. . . . 50
25 Covariate hazard ratios for COVID-19 death in the BNT162b2 vs unvaccinated comparison and the 18-64 years and clinically vulnerable subgroup. . . . 51
26 Covariate hazard ratios for Positive SARS-CoV-2 test in the BNT162b2 vs unvaccinated comparison and the 18-64 years and clinically vulnerable subgroup. . . . 52
27 Covariate hazard ratios for Non-COVID-19 death in the BNT162b2 vs unvaccinated comparison and the 18-64 years and clinically vulnerable subgroup. . . . 53
28 Covariate hazard ratios for Any SARS-CoV-2 test in the BNT162b2 vs unvaccinated comparison and the 18-64 years and clinically vulnerable subgroup. . . . 54
29 Covariate hazard ratios for COVID-19 hospitalisation in the ChAdOx1 vs unvaccinated comparison and the 18-64 years and clinically vulnerable subgroup. . . . 55
30 Covariate hazard ratios for COVID-19 death in the ChAdOx1 vs unvaccinated comparison and the 18-64 years and clinically vulnerable subgroup. . . . 56
31 Covariate hazard ratios for Positive SARS-CoV-2 test in the ChAdOx1 vs unvaccinated comparison and the 18-64 years and clinically vulnerable subgroup. . . . 57
32 Covariate hazard ratios for Non-COVID-19 death in the ChAdOx1 vs unvaccinated comparison and the 18-64 years and clinically vulnerable subgroup. . . . 58
33 Covariate hazard ratios for Any SARS-CoV-2 test in the ChAdOx1 vs unvaccinated comparison and the 18-64 years and clinically vulnerable subgroup. . . . 59
34 Covariate hazard ratios for COVID-19 hospitalisation in the BNT162b2 vs ChAdOx1 comparison and the 18-64 years and clinically vulnerable subgroup. . . . 60
35 Covariate hazard ratios for COVID-19 death in the BNT162b2 vs ChAdOx1 comparison and the 18-64 years and clinically vulnerable subgroup. . . . 61
36 Covariate hazard ratios for Positive SARS-CoV-2 test in the BNT162b2 vs ChAdOx1 comparison and the 18-64 years and clinically vulnerable subgroup. . . . 62
37 Covariate hazard ratios for Non-COVID-19 death in the BNT162b2 vs ChAdOx1 comparison and the 18-64 years and clinically vulnerable subgroup. . . . 63
38 Covariate hazard ratios for Any SARS-CoV-2 test in the BNT162b2 vs ChAdOx1 comparison and the 18-64 years and clinically vulnerable subgroup. . . . 64
39 Covariate hazard ratios for Positive SARS-CoV-2 test in the BNT162b2 vs unvaccinated comparison and the 40-64 years subgroup. . . . 65
40 Covariate hazard ratios for Any SARS-CoV-2 test in the BNT162b2 vs unvaccinated comparison and the 40-64 years subgroup. . . . 66
41 Covariate hazard ratios for COVID-19 hospitalisation in the ChAdOx1 vs unvaccinated comparison and the 40-64 years subgroup. . . . 67
42 Covariate hazard ratios for COVID-19 death in the ChAdOx1 vs unvaccinated comparison and the 40-64 years subgroup. 68
43 Covariate hazard ratios for Positive SARS-CoV-2 test in the ChAdOx1 vs unvaccinated comparison and the 40-64 years subgroup. . . . 69

44 Covariate hazard ratios for Non-COVID-19 death in the ChAdOx1 vs unvaccinated comparison and the 40-64 years subgroup. . . . 70
45 Covariate hazard ratios for Any SARS-CoV-2 test in the ChAdOx1 vs unvaccinated comparison and the 40-64 years subgroup. . . . 71
46 Covariate hazard ratios for Positive SARS-CoV-2 test in the BNT162b2 vs ChAdOx1 comparison and the 40-64 years subgroup. . . . 72
47 Covariate hazard ratios for Any SARS-CoV-2 test in the BNT162b2 vs ChAdOx1 comparison and the 40-64 years subgroup. . . . 73
48 Covariate hazard ratios for COVID-19 hospitalisation in the BNT162b2 vs unvaccinated comparison and the 18-39 years subgroup. . . . 74
49 Covariate hazard ratios for Positive SARS-CoV-2 test in the BNT162b2 vs unvaccinated comparison and the 18-39 years subgroup. . . . 76
50 Covariate hazard ratios for Any SARS-CoV-2 test in the BNT162b2 vs unvaccinated comparison and the 18-39 years subgroup. . . . 77
51 Event counts / person-years (females only). . . . 79
52 Event counts / person-years (males only). . . . 80
53 Unadjusted hazard ratios for effect of vaccination (female-only model). . . . 81
54 Unadjusted hazard ratios for effect of vaccination (male-only model). . . . 82
55 Adjusted hazard ratios for effect of vaccination (female-only model). . . . 83
56 Adjusted hazard ratios for effect of vaccination (male-only model). . . . 84
57 Per-comparison-period ratio of adjusted hazard ratios for effect of vaccination (female-only model). . . . 85
58 Per-comparison-period ratio of adjusted hazard ratios for effect of vaccination (male-only model). . . . 86
59 Event counts / person-years (older adults). . . . 91
60 Unadjusted hazard ratios for effect of vaccination (older adults). . . . 92
61 Adjusted hazard ratios for effect of vaccination (older adults). . . . 93
62 Per-comparison-period ratio of adjusted hazard ratios for effect of vaccination (older adults). . . . 93

List of Figures

1 Study eligibility criteria. . . . 7
2 Flow of individuals into study 8
3 Second vaccination period for JCVI group 2 9
4 Second vaccination period for JCVI group 3 9
5 Second vaccination period for JCVI group 4 10
6 Second vaccination period for JCVI group 4 10
7 Second vaccination period for JCVI group 5 11
8 Second vaccination period for JCVI group 6 11
9 Second vaccination period for JCVI group 7 and aged 64 years 12
10 Second vaccination period for JCVI group 7 and aged 60-63 years 12
11 Second vaccination period for JCVI group 8 13
12 Second vaccination period for JCVI group 9 13
13 Second vaccination period for JCVI group 10 and aged 45-49 years 14
14 Second vaccination period for JCVI group 10 and aged 40-44 years 14

15 Second vaccination period for JCVI group 11 and aged 36-39 years 15
16 Second vaccination period for JCVI group 11 and aged 30-35 years 15
17 Second vaccination period for JCVI group 12 and aged 25-29 years 16
18 Second vaccination period for JCVI group 12 and aged 18-24 years 16
19 Cumulative incidence of subsequent vaccination. *And not clinically vulnerable. 18
20 Distribution of follow-up time across the six comparison periods in the 65+ years subgroup. Comparison periods (numeric labels on right-hand side of plots) correspond to the following weeks since second vaccine dose: 1 = 3-6 weeks; 2 = 7-10 weeks; 3: 11-14 weeks; 4: 15-18 weeks; 5: 19-22 weeks; 6 = 23-26 weeks. 19
21 Distribution of follow-up time across the six comparison periods in the 18-64 years and clinically vulnerable subgroup. Comparison periods (numeric labels on right-hand side of plots) correspond to the following weeks since second vaccine dose: 1 = 3-6 weeks; 2 = 7-10 weeks; 3: 11-14 weeks; 4: 15-18 weeks; 5: 19-22 weeks; 6 = 23-26 weeks. . 20
22 Distribution of follow-up time across the six comparison periods in the 40-64 years subgroup. Comparison periods (numeric labels on right-hand side of plots) correspond to the following weeks since second vaccine dose: 1 = 3-6 weeks; 2 = 7-10 weeks; 3: 11-14 weeks; 4: 15-18 weeks; 5: 19-22 weeks; 6 = 23-26 weeks. 21
23 Distribution of follow-up time across the six comparison periods in the 18-39 years subgroup. Comparison periods (numeric labels on right-hand side of plots) correspond to the following weeks since second vaccine dose: 1 = 3-6 weeks; 2 = 7-10 weeks; 3: 11-14 weeks; 4: 15-18 weeks; 5: 19-22 weeks; 6 = 23-26 weeks. 22
24 Unadjusted and adjusted hazard ratios for BNT162b2 vs unvaccinated 27
25 Unadjusted and adjusted hazard ratios for ChAdOx1 vs unvaccinated 28
26 Unadjusted and adjusted hazard ratios for BNT162b2 vs ChAdOx1 29
27 Unadjusted and adjusted hazard ratios for any SARS-CoV-2 test for BNT162b2 vs unvaccinated 30
28 Unadjusted and adjusted hazard ratios for any SARS-CoV-2 test for ChAdOx1 vs unvaccinated 31
29 Unadjusted and adjusted hazard ratios for any SARS-CoV-2 test for BNT162b2 vs ChAdOx1 32
30 Adjusted hazard ratios for any SARS-CoV-2 test for BNT162b2 and ChAdOx1 vs unvaccinated 33
31 Adjusted hazard ratios for any SARS-CoV-2 test for BNT162b2 vs ChAdOx1 34
32 Adjusted hazard ratios for BNT162b2 vs unvaccinated from the female- and male-only models. 87
33 Adjusted hazard ratios for ChAdOx1 vs unvaccinated from the female- and male-only models. 88
34 Adjusted hazard ratios for any SARS-CoV-2 test for BNT162b2 vs unvaccinated from the female- and male-only models. 89
35 Adjusted hazard ratios for any SARS-CoV-2 test for ChAdOx1 vs unvaccinated from the female- and male-only models. 90
36 Adjusted hazard ratios for BNT162b2 vs unvaccinated from the 65-74 years and 75+ years models. 94
37 Adjusted hazard ratios for ChAdOx1 vs unvaccinated from the 65-74 years and 75+ years models. 95
38 Adjusted hazard ratios for any SARS-CoV-2 test for BNT162b2 vs unvaccinated from the 65-74 years and 75+ years models. 96
39 Adjusted hazard ratios for any SARS-CoV-2 test for ChAdOx1 vs unvaccinated from the 65-74 years and 75+ years models. 96

Supplementary Methods

Supplementary Table 1: Vaccination phases and priority groups for primary vaccination advised by the Joint Committee on Vaccination and Immunisation

Vaccination phase	Priority group	Risk group
1	1	Residents in a care home for older adults
		Staff in a care home for older adults
	2	All those 80 years of age and over
		Frontline health and social care workers
	3	All those 75 years of age and over
	4a	All those 70 years of age and over
	4b	Individuals aged 16 to 69 in a high risk group[a]
	5	All those 65 years of age and over
	6	Individuals aged 16 to 65 years in an at-risk group[a]
	7	All those 60 years of age and over
	8	All those 55 years of age and over
	9	All those 50 years of age and over
2	10	All those 40 years of age and over
	11	All those 30 years of age and over
	12	All those 18 years of age and over

[a] See COVID-19: the green book, chapter 14a for definitions of the high-risk and at-risk groups.

Supplementary Table 2: Eligibility dates for first vaccine dose

Grouped eligibility date	Exact eligibility date	JCVI groups	Age range	Reference
2020-12-08	2020-12-08	1, 2	-	source
2021-01-18	2021-01-18	3, 4a, 4b	-	source
2021-02-15	2021-02-15	5, 6	-	source
2021-02-22	2021-02-22	7	64	source
2021-03-01	2021-03-01	7	60-63	source
2021-03-08	2021-03-08	8	56-59	source
	2021-03-09	8	55	source
2021-03-19	2021-03-19	9	50-54	source
2021-04-13	2021-04-13	10	45-49	source
2021-04-26	2021-04-26	10	44	source
	2021-04-27	10	42-43	source
	2021-04-30	10	40-41	source
2021-05-13	2021-05-13	11	38-39	source
	2021-05-19	11	36-37	source
2021-05-21	2021-05-21	11	34-35	source
	2021-05-25	11	32-33	source
	2021-05-26	11	30-31	source
2021-06-08	2021-06-08	12	25-29	source
2021-06-15	2021-06-15	12	23-24	source
	2021-06-16	12	21-22	source
	2021-06-18	12	18-20	source

JCVI: Joint Committee on Vaccination and Immunisation

Supplementary Table 3: Variable definitions

Name	Description	Encoding	Date defined
**Exclusion criteria**			
Evidence of prior COVID-19 infection	Date of code corresponding to COVID-19 hospitalisation, positive SARS-CoV-2 test, or probable COVID-19 code in GP record.	Date	First occurence
End-of-life care pathway initiated	Date of code corresponding to end-of-life care or midazolam injection used in end-of-life care in patient's record.	Date	First occurence prior to SVP
Resident in care home	Date of code corresponding to care home in patient's record.	Date	First occurence prior to SVP
Medically housebound	Date of most recent code corresponding to medically housebound patient's record, not superceded by a code indicationg that the patient is no longer housebound.	Date	First occurence prior to SVP
**JCVI group definitions**			
Age	-	Numeric	31 March 2021 for individuals eligible in phase 1 and 1 July 2021 for individuals eligible in phase 2
High risk	A code in the primary care record indicating that the patient was at high risk of developing complications from COVID-19, not superseded by a code indicating that they were at low or moderate risk.	0; 1.	18 January 2021 (eligibility date for high risk individuals).
At-risk	A code corresponding to one or more of the following: severe obesity (BMI 40+ kg/m^2); learning disability including Down's syndrome; serious mental illness; chronic heart disease; chronic respiratory disease; chronic liver disease; chronic kidney disease; chronic neurological disease; diabetes; immunosuppressed (diagnosis of permenant immunosuppression or asplenia, or currently taking immunosuppresant medication).	0; 1.	15 February 2021 (eligibility date for at-rosk individuals).
**Strata**			
JCVI group	Priority groups 2-9 are as defined by the JCVI expert advisory group, and we further define groups 10, 11 and 12 are defined as those aged 40-49, 30-39 and 18-29 years, respectively, who were not assigned to JCVI groups 4 (high risk) or 6 (at-risk).	2-12	-
Eligibility date	Date on which JCVI group (or age range within group) became eligible for 1st dose of COVID-19 vaccination.	Date	-
Region	NHS region based on practice address.	East of England; Midlands; London; North East; Yorkshire; North West; South East; South West.	Eligibility date + 6 weeks.
**Potential confounders (demographic)**			
Sex	-	M; F.	-
Age	Age in whole years.	Numeric[a]	Start of SVP
Ethnicity	From primary care records or SUS if missing from primary care.	Black; Mixed; South Asian; White; Other.	Eligibility date + 6 weeks

Supplementary Table 3: Variable definitions *(continued)*

Name	Description	Encoding	Date defined
Index of Multiple Deprivation (IMD)	IMD quintile based on patient address. The IMD is a measure of socioeconomic deprivation based on lower-layer super output area (LSOA; a small geographical area defined by the Office of National Statistics). For more information see the English IMD 2019: technical report	1; 2; 3; 4; 5.	Eligibility date + 6 weeks
**Potential confounders (clinical)**			
Body Mass Index (BMI)	Based on numeric BMI data from primary care records.	Under 30kg/m^2 or not recorded; 30-34.9; 35-39.9; 40+ kg/m^2.	Start of SVP
Learning disability including Down's syndrome.	Any code in primary care record before date defined.	0; 1.	Start of SVP
Serious mental illness.	Any code in primary care record before date defined.	0; 1.	Start of SVP
Multimorbidity	Number of the following comorbid conditions in different organ systems: Chronic heart disease; Chronic respiratory disease; Chronic liver disease; Chronic kidney disease; Diabetes; Chronic neurological disease; Immunosuppressed (diagnosis of permenant immunosuppression or asplenia, or currently taking immunosuppresant medication)	0; 1; 2+	Start of SVP
Influenza vaccination	Any record in past 5 years.	0; 1.	Start of SVP
Pregnancy	Pregnancy recorded in 36 weeks prior to date and no delivery code recorded more recently that pregnancy code.	0; 1.	Start of SVP
Number of SARS-CoV-2 tests.	SARS-CoV-2 tests were identified using SGSS records and based on swab date. Both polymerase chain reaction (PCR) and lateral flow tests will be included, without differentiation between symptomatic and asymptomatic infection.	Integer	Between 18 May 2020 (when widespread testing became available in England) and the earliest date of eligibility for first dose in the subgroup.

JCVI: Joint Committee on Vaccination and Immunisation; SVP: second vaccination period.
[a] Age was modelled separately within each strata. Age within strata was modelled as linear, with quadratic terms additionally included for strata with age range >5 years.

Box A: Initial eligibility criteria.
Inclusion criteria:
- Registered at a GP surgery (using TPP's SystemOne clinical information system) for at least one year prior to the date at which they became eligible for vaccination.
- Aged 18 years or over on 1 July 2021

Exclusion criteria:
- Aged over 120 on 1 July 2021
- Sex unknown
- Geographical region, ethnicity or deprivation unknown on eligibility date + 6 weeks.
- Evidence of SARS-CoV-2 infection recorded on or before eligibility date + 6 weeks.
- Recorded as being in care home or medically housebound on or before eligibility date + 6 weeks

Box B: Eligibility criteria for identification of second vaccination period (SVP).
Inclusion criteria:
- Received second dose of BNT162b2 or ChAdOx1 between eligibility date + 6 weeks and eligibility date + 16 weeks (first and second doses must be same brand).

Exclusion criteria:
- First dose received before eligibility date.
- Interval of less than 6 or more than 14 weeks between first and second doses.
- Flagged as healthcare worker on vaccination record.

Box C: Inclusion criteria for vaccinated group:
- Second dose of BNT162b2 or ChAdOx1 received during the SVP.

Box D: Inclusion criteria for unvaccinated group:
- No doses of vaccine received before the start of the SVP.

Box E: Exclusion criteria for both groups:
- Evidence of SARS-CoV-2 infection recorded on or before start of SVP.
- Recorded as being in care home on or before start of the SVP.
- End-of-life care pathway initiated on or before start of the SVP.

Box F: Individual *i* excluded from comparison period *k* with outcome *z* if any of the following events have occurred on or before the start of their follow-up for comparison period *k* (start_fu_{ki}):
- Third dose of vaccine received for individuals in the vaccinated group, or first dose of vaccine received for individuals in the unvaccinated group.
- Outcome *z*.*
- Death of any cause.
- De-registration from all practices using using TPP's SystemOne clinical information system.

Supplementary Figure 1: Study eligibility criteria.

Supplementary Results

Study population

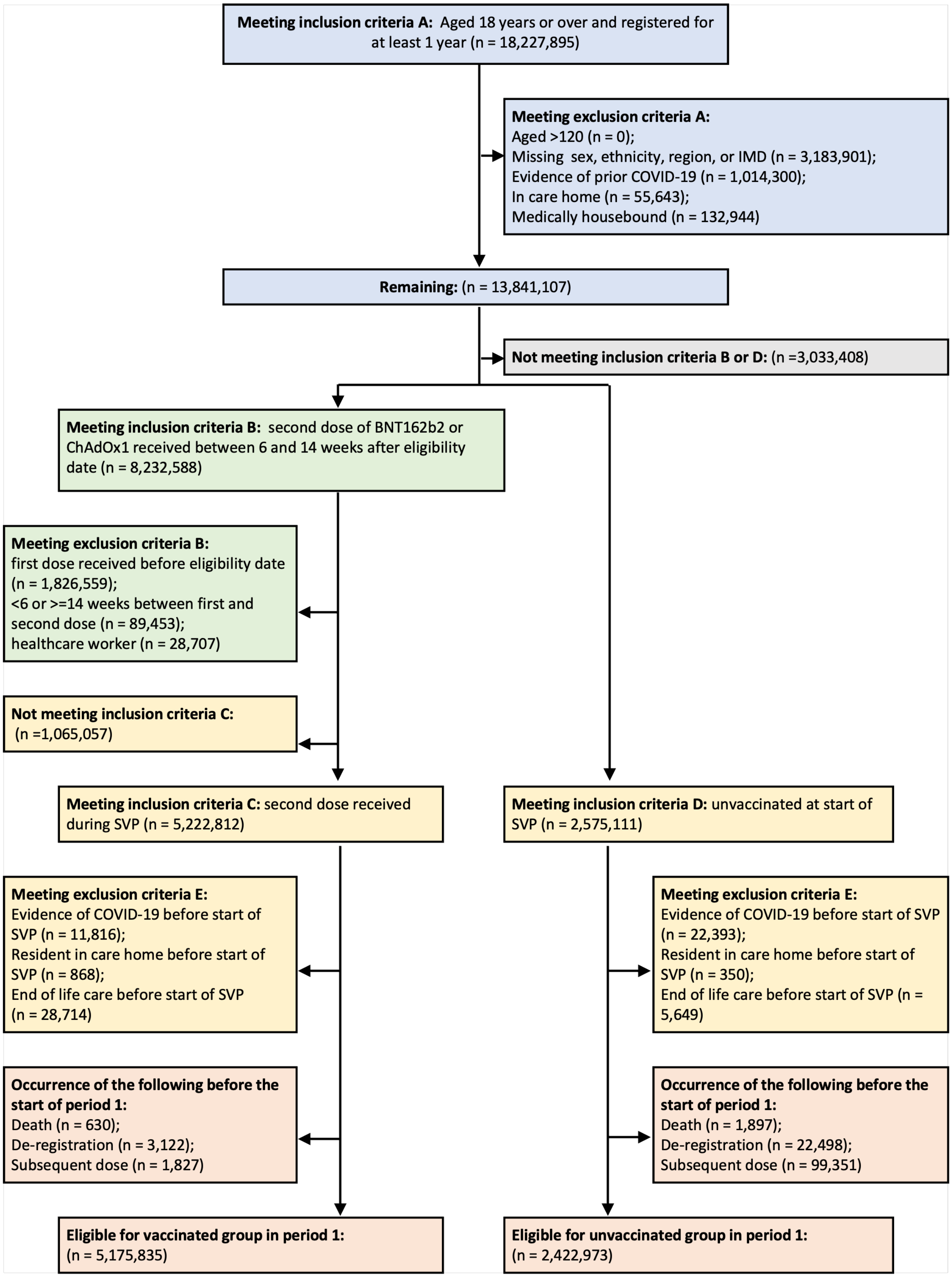

Supplementary Figure 2: Flow of individuals into study

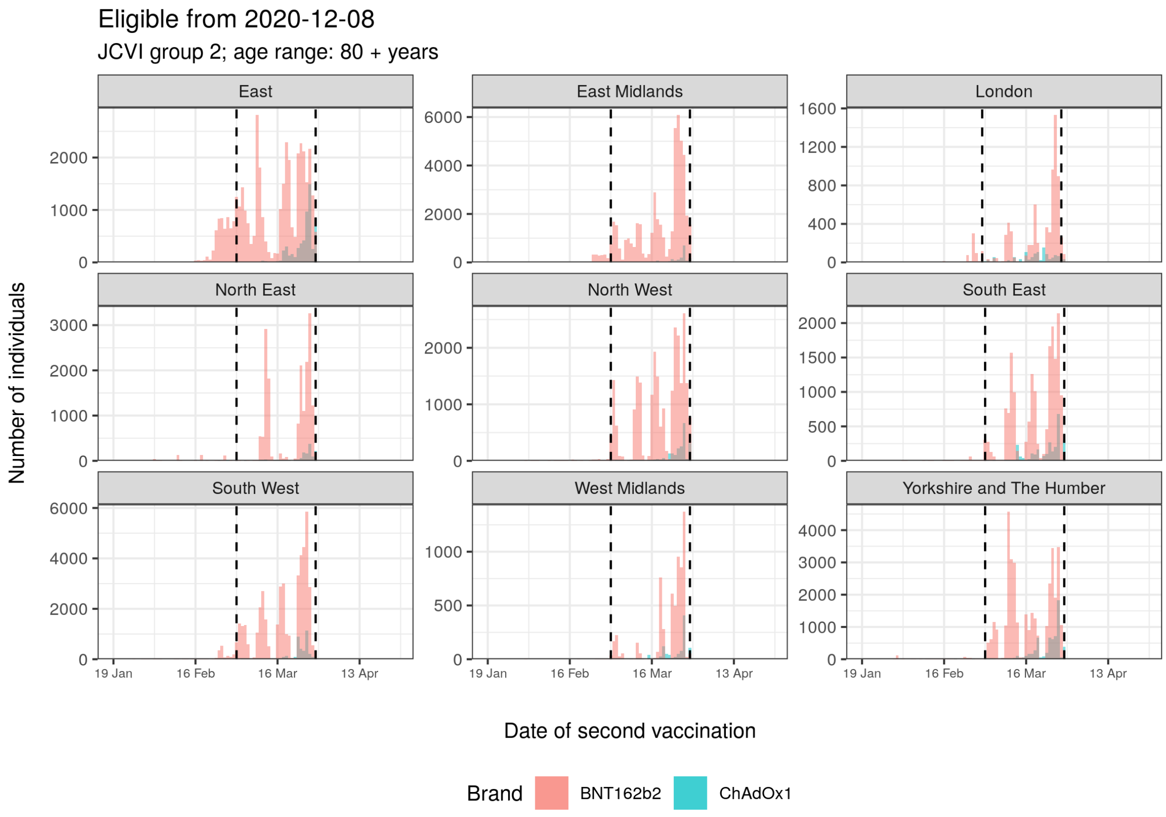

Supplementary Figure 3: Second vaccination period for JCVI group 2

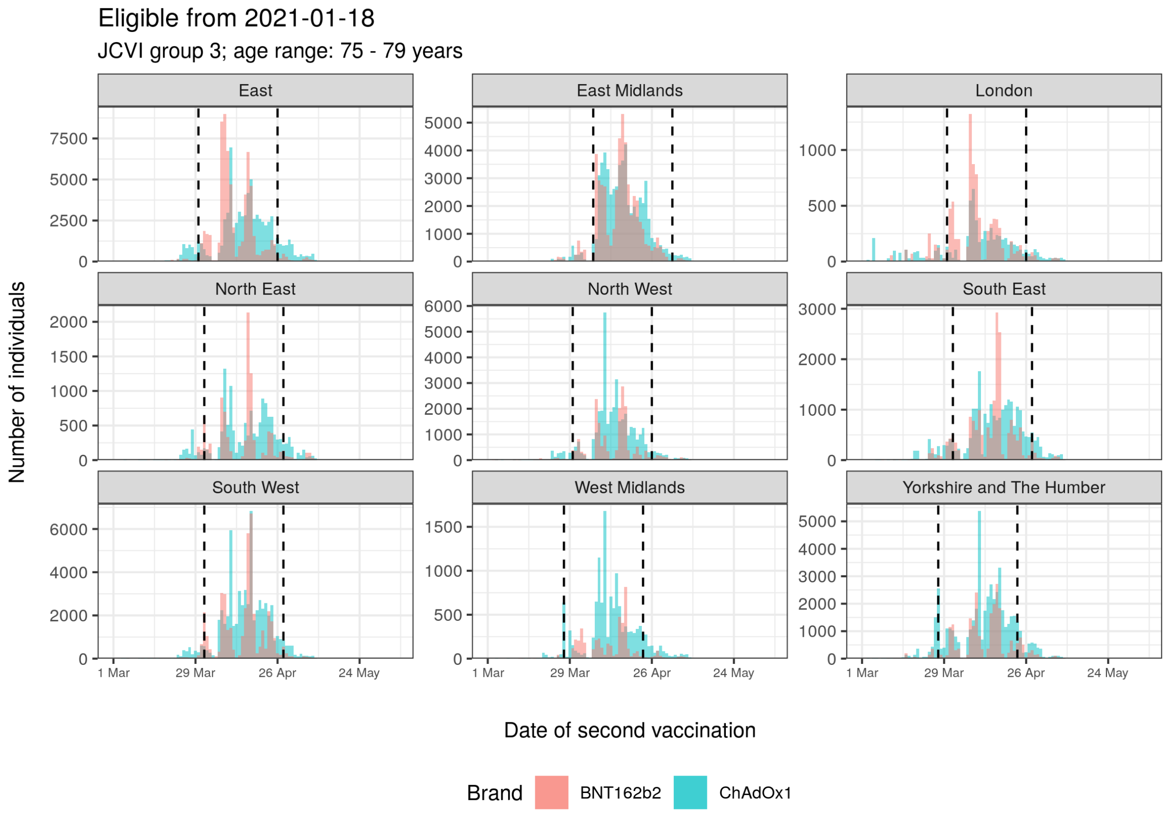

Supplementary Figure 4: Second vaccination period for JCVI group 3

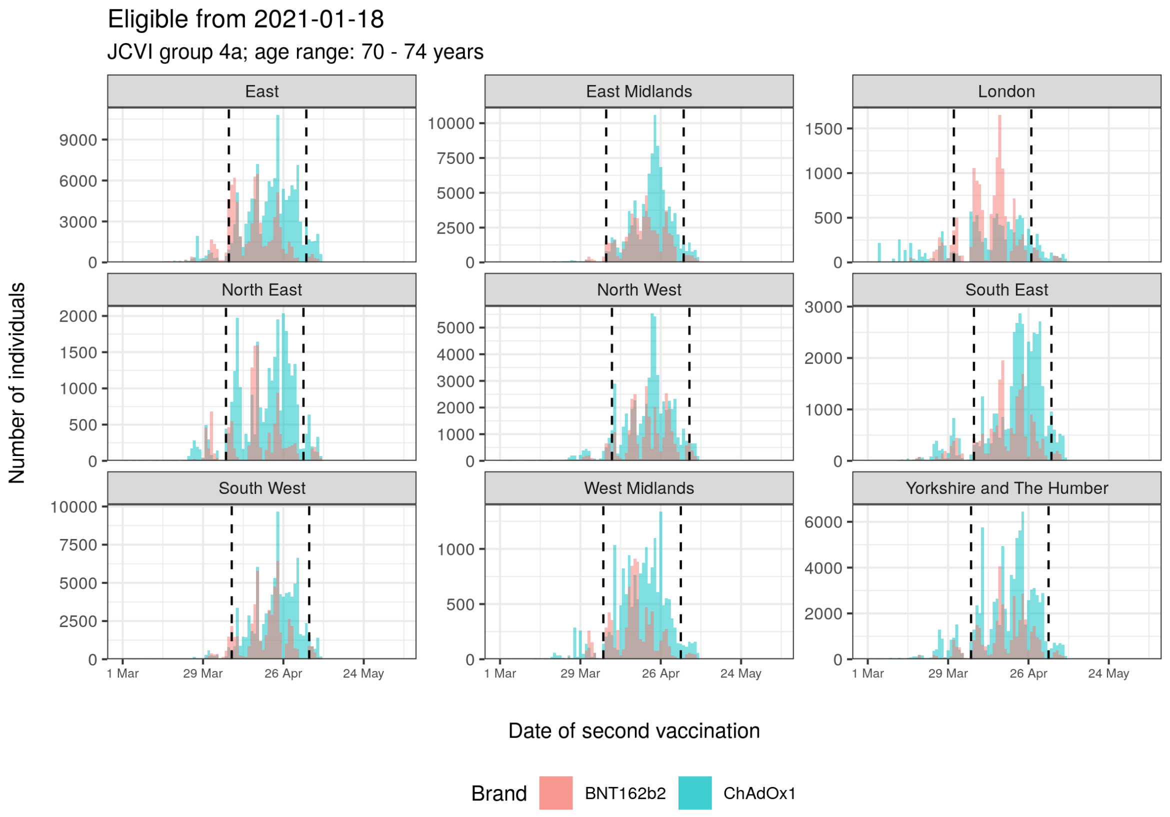

Supplementary Figure 5: Second vaccination period for JCVI group 4

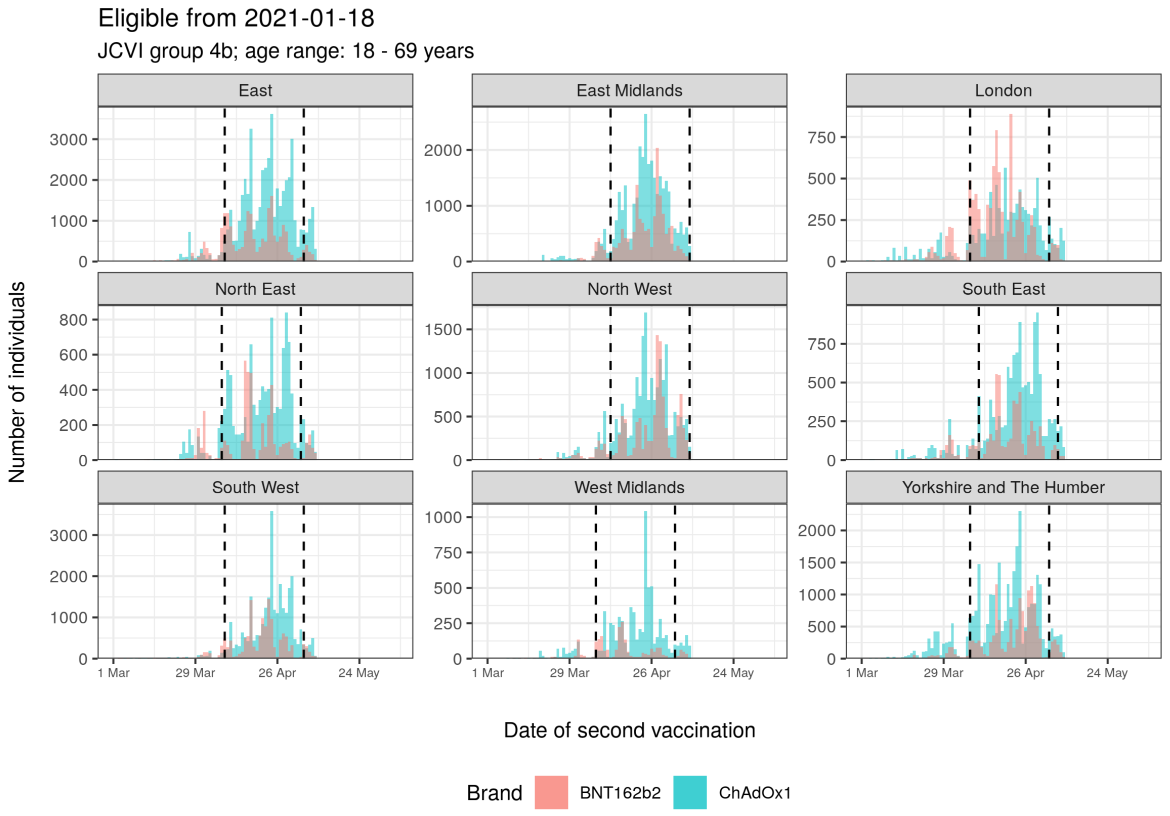

Supplementary Figure 6: Second vaccination period for JCVI group 4

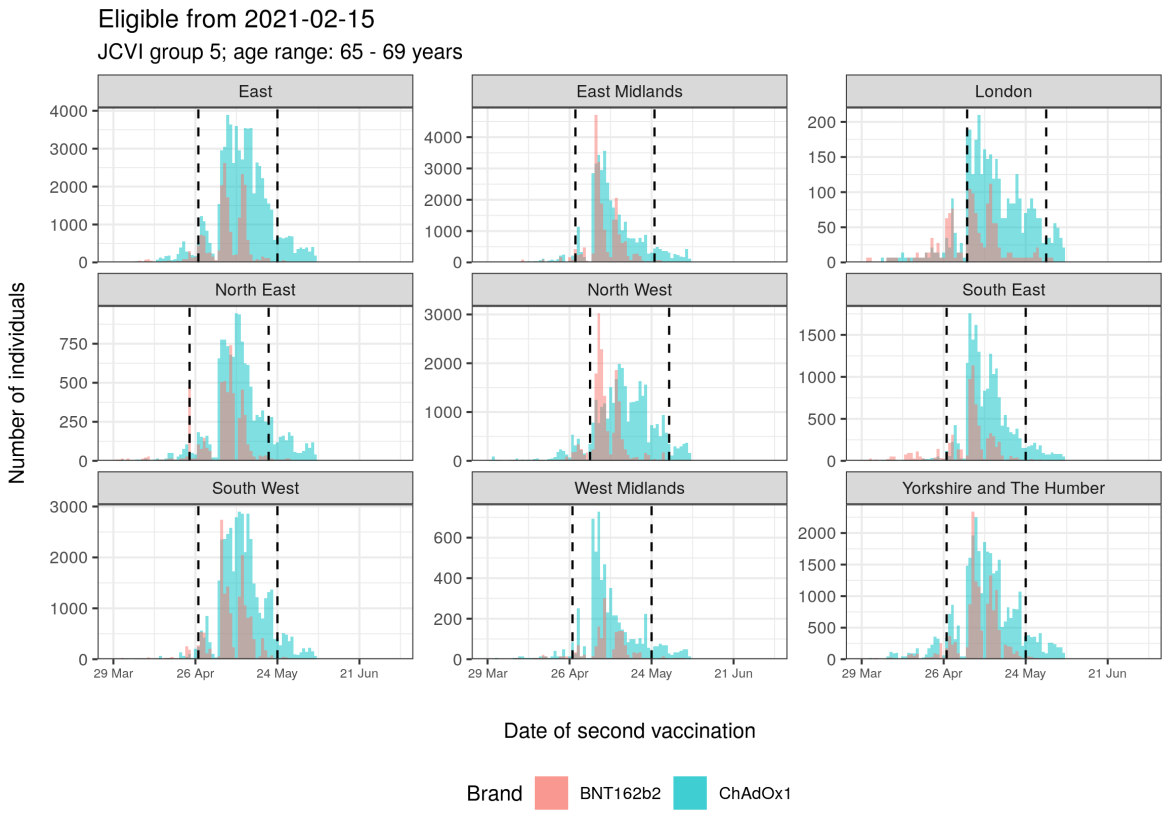

Supplementary Figure 7: Second vaccination period for JCVI group 5

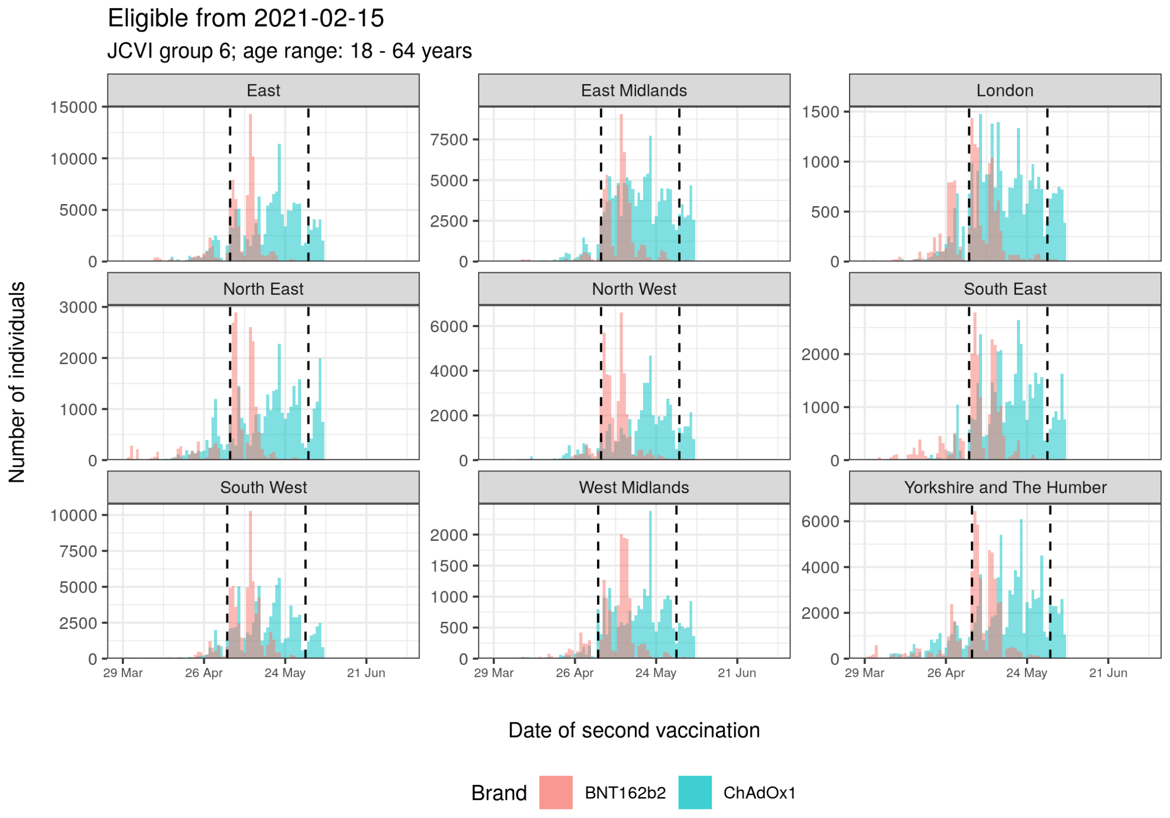

Supplementary Figure 8: Second vaccination period for JCVI group 6

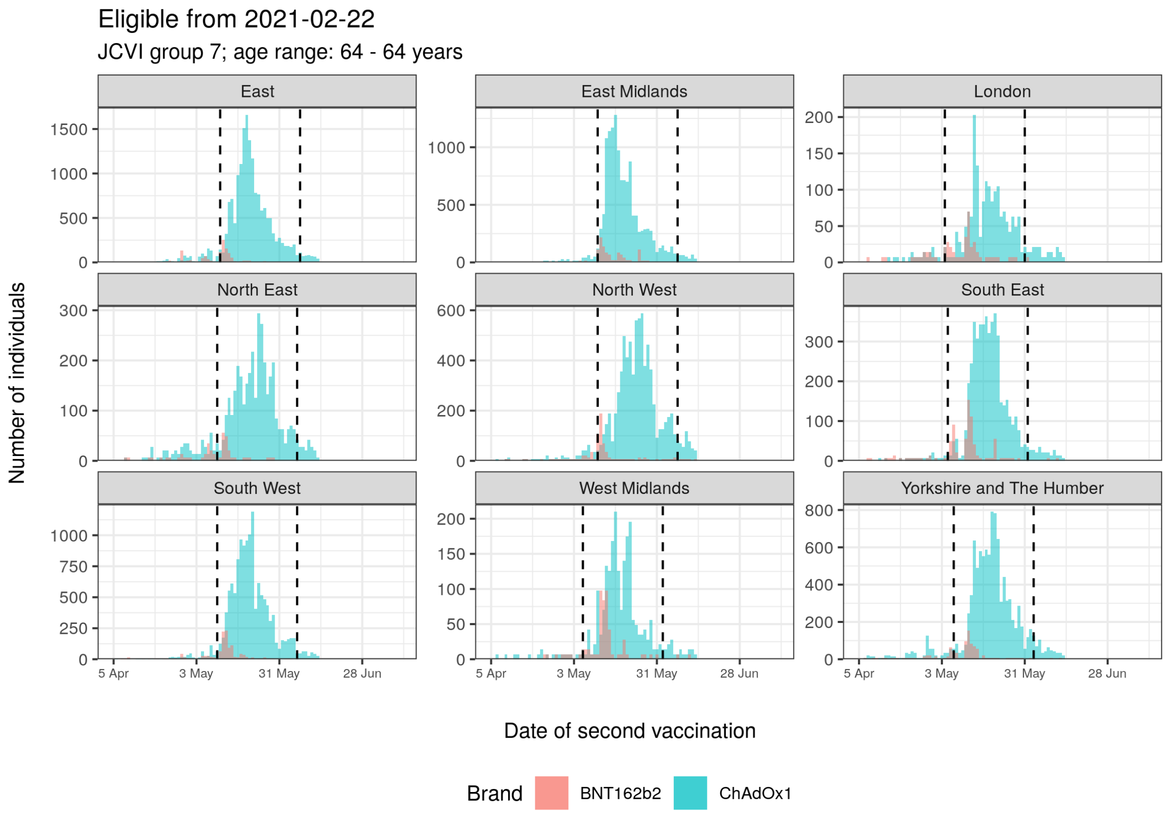

Supplementary Figure 9: Second vaccination period for JCVI group 7 and aged 64 years

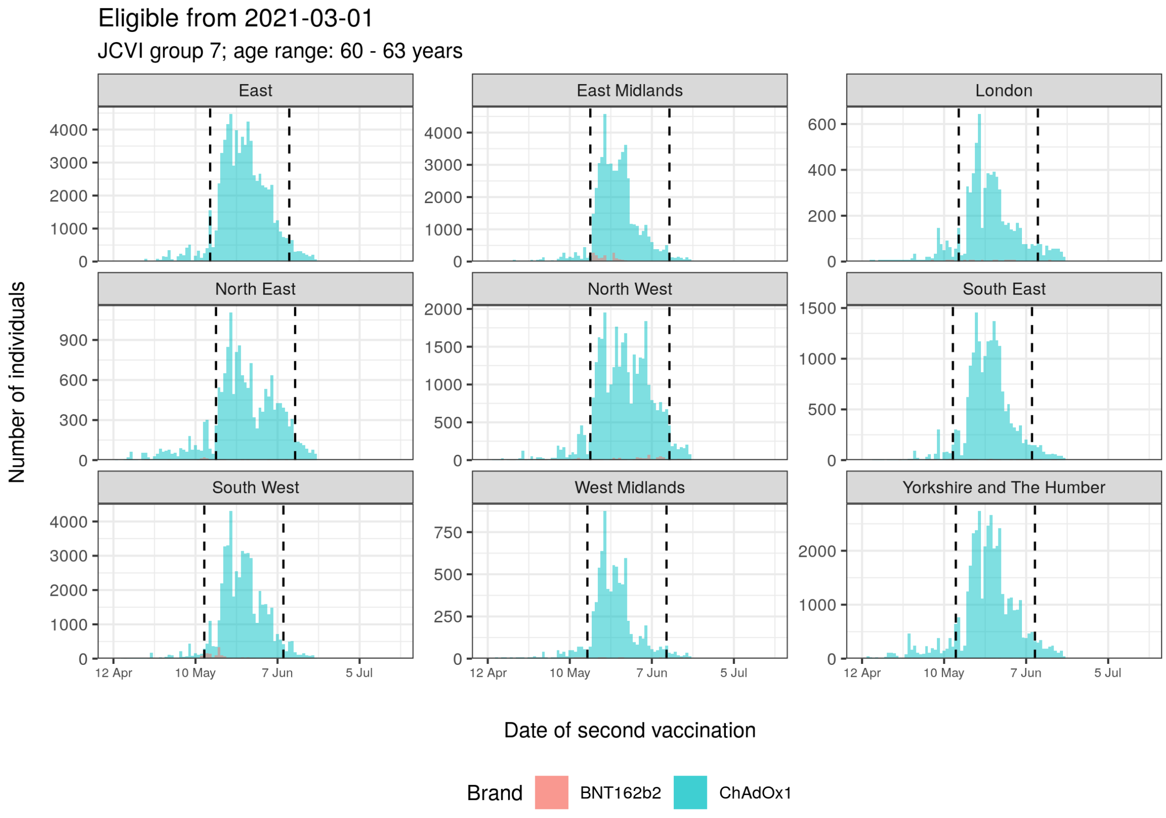

Supplementary Figure 10: Second vaccination period for JCVI group 7 and aged 60-63 years

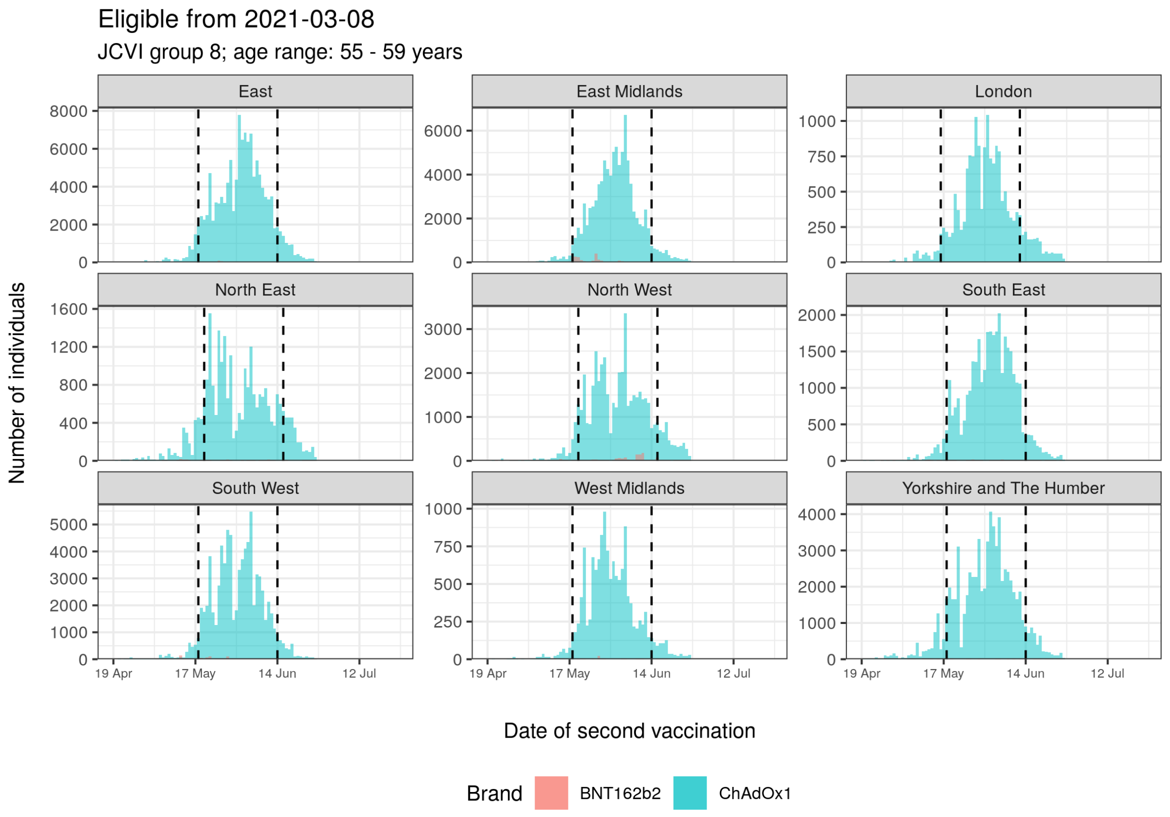

Supplementary Figure 11: Second vaccination period for JCVI group 8

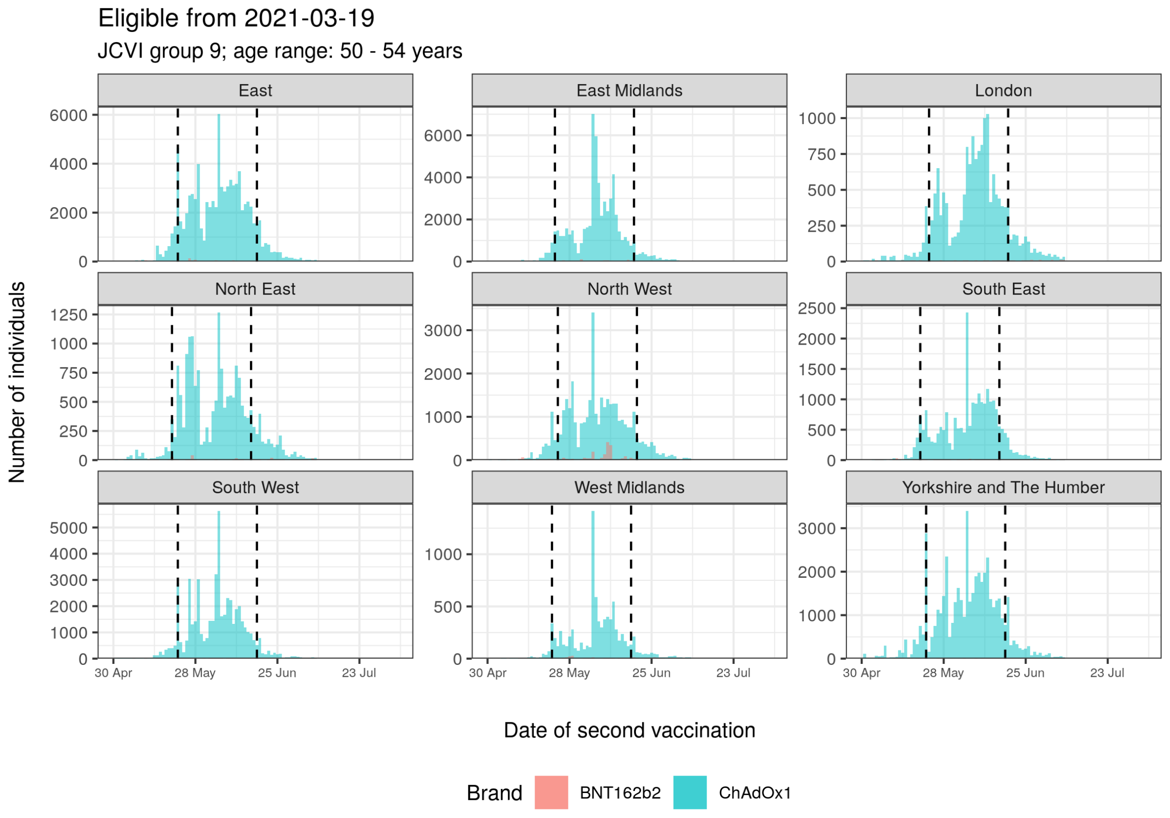

Supplementary Figure 12: Second vaccination period for JCVI group 9

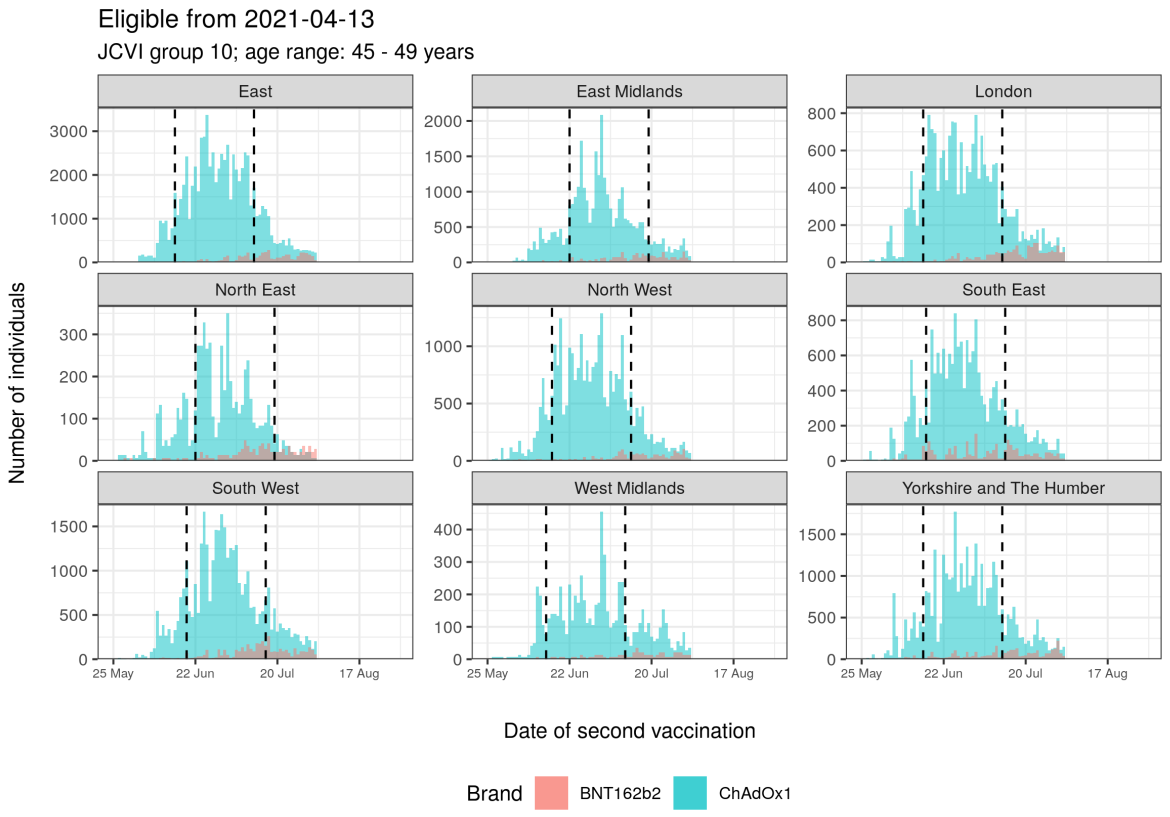

Supplementary Figure 13: Second vaccination period for JCVI group 10 and aged 45-49 years

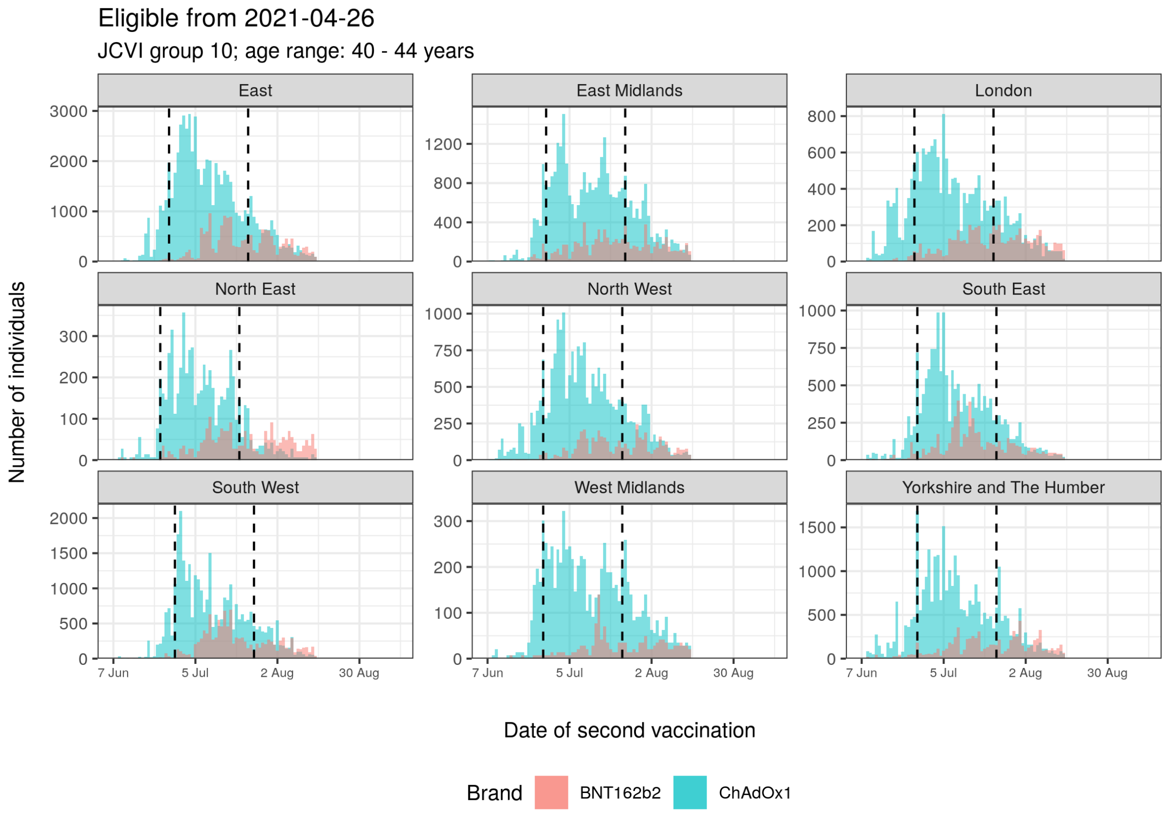

Supplementary Figure 14: Second vaccination period for JCVI group 10 and aged 40-44 years

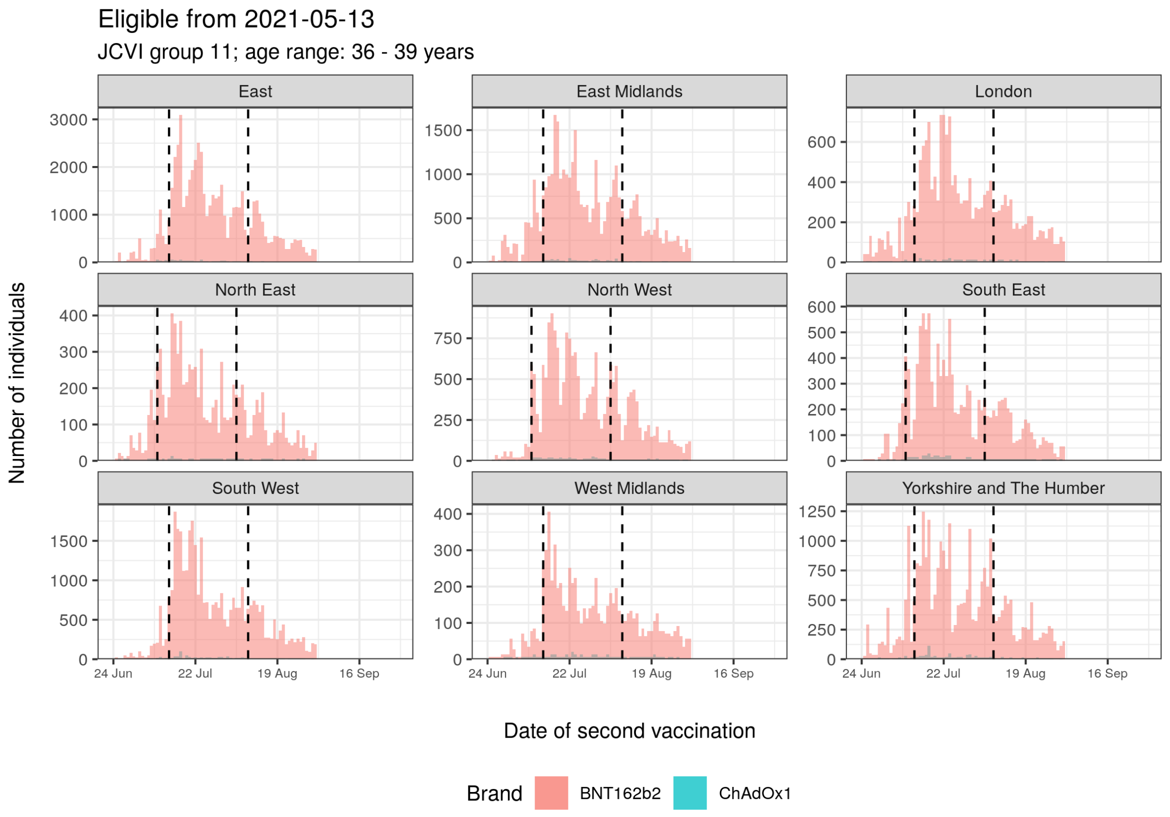

Supplementary Figure 15: Second vaccination period for JCVI group 11 and aged 36-39 years

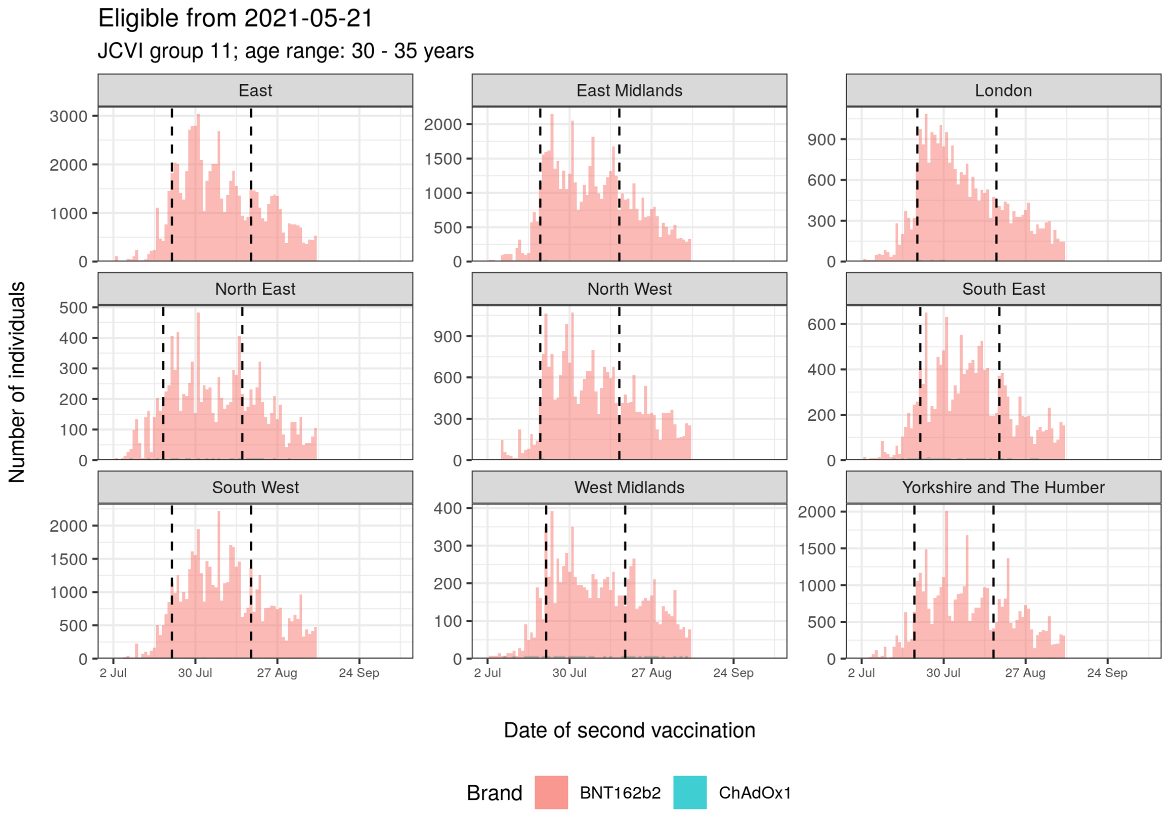

Supplementary Figure 16: Second vaccination period for JCVI group 11 and aged 30-35 years

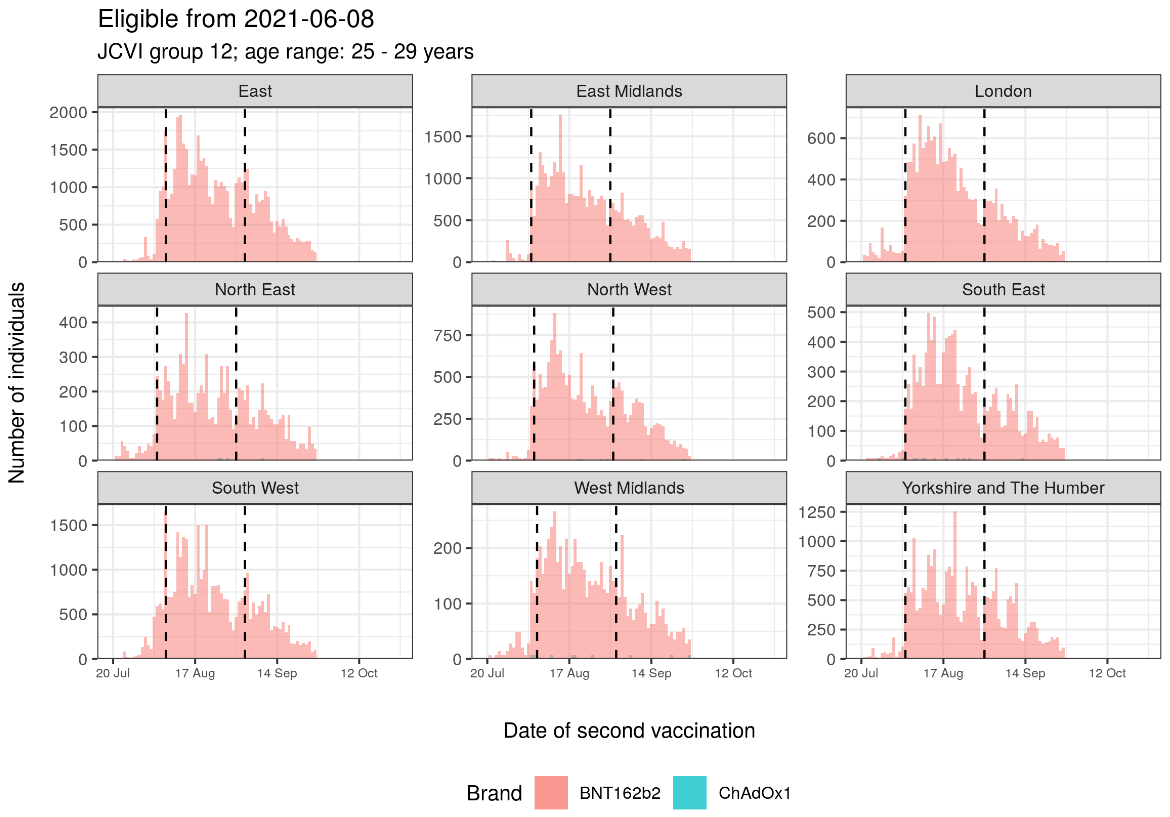

Supplementary Figure 17: Second vaccination period for JCVI group 12 and aged 25-29 years

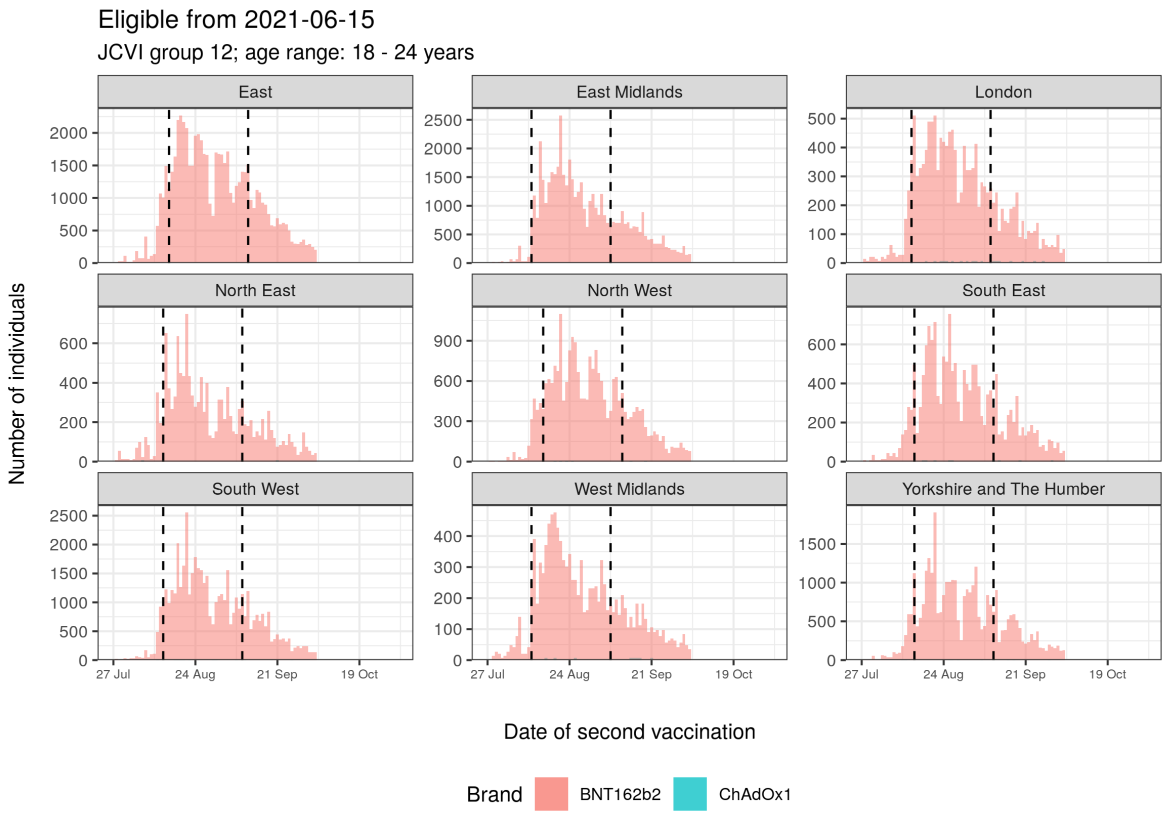

Supplementary Figure 18: Second vaccination period for JCVI group 12 and aged 18-24 years

Supplementary Table 4: Additional characteristics summarised across subgroups.

		65+ years			18-64 years and clinically vulnerable			40-64 years[a]			18-39 years[a]	
**Characteristic**	**Characteristic**	**BNT162b2**	**ChAdOx1**	**Unvaccinated**	**BNT162b2**	**ChAdOx1**	**Unvaccinated**	**BNT162b2**	**ChAdOx1**	**Unvaccinated**	**BNT162b2**	**Unvaccinated**
N		830,662	1,096,872	144,774	368,732	649,075	300,881	63,021	1,473,402	625,226	689,451	1,352,099
Region	East	182,441 (22%)	248,297 (23%)	32,102 (22%)	79,870 (22%)	150,577 (23%)	61,488 (20%)	13,545 (21%)	377,615 (26%)	139,706 (22%)	165,361 (24%)	300,195 (22%)
	East Midlands	169,330 (20%)	192,297 (18%)	21,903 (15%)	63,406 (17%)	133,644 (21%)	51,184 (17%)	11,809 (19%)	249,235 (17%)	95,851 (15%)	119,343 (17%)	221,284 (16%)
	London	28,147 (3%)	18,963 (2%)	20,356 (14%)	14,518 (4%)	28,203 (4%)	34,545 (11%)	3,633 (6%)	63,714 (4%)	105,518 (17%)	53,270 (8%)	194,313 (14%)
	North East	40,446 (5%)	52,752 (5%)	5,866 (4%)	18,795 (5%)	32,424 (5%)	16,114 (5%)	1,757 (3%)	63,378 (4%)	25,242 (4%)	27,048 (4%)	62,153 (5%)
	North West	84,385 (10%)	122,262 (11%)	9,625 (7%)	45,262 (12%)	59,115 (9%)	23,716 (8%)	6,489 (10%)	149,520 (10%)	40,264 (6%)	60,599 (9%)	90,174 (7%)
	South East	56,105 (7%)	78,918 (7%)	9,695 (7%)	20,314 (6%)	42,910 (7%)	15,771 (5%)	5,824 (9%)	102,249 (7%)	37,513 (6%)	38,479 (6%)	78,414 (6%)
	South West	149,415 (18%)	194,306 (18%)	17,955 (12%)	62,265 (17%)	89,467 (14%)	28,917 (10%)	12,831 (20%)	233,709 (16%)	61,663 (10%)	119,392 (17%)	125,447 (9%)
	West Midlands	19,579 (2%)	35,014 (3%)	7,966 (6%)	13,916 (4%)	26,201 (4%)	19,012 (6%)	1,337 (2%)	39,711 (3%)	31,444 (5%)	23,345 (3%)	76,685 (6%)
	Yorkshire and The Humber	100,842 (12%)	154,084 (14%)	19,313 (13%)	50,414 (14%)	86,555 (13%)	50,155 (17%)	5,817 (9%)	194,299 (13%)	88,046 (14%)	82,635 (12%)	203,455 (15%)
JCVI group	02	225,106 (27%)	23,268 (2%)	30,436 (21%)	-	-	-	-	-	-	-	-
	03	222,292 (27%)	292,005 (27%)	22,449 (16%)	-	-	-	-	-	-	-	-
	04a	271,439 (33%)	514,983 (47%)	38,696 (27%)	-	-	-	-	-	-	-	-
	04b	19,355 (2%)	38,892 (4%)	3,199 (2%)	61,264 (17%)	130,347 (20%)	29,834 (10%)	-	-	-	-	-
	05	92,484 (11%)	227,731 (21%)	50,008 (35%)	-	-	-	-	-	-	-	-
	06	-	-	-	307,468 (83%)	518,735 (80%)	271,054 (90%)	-	-	-	-	-
	07	-	-	-	-	-	-	8,708 (14%)	346,850 (24%)	52,892 (8%)	-	-
	08	-	-	-	-	-	-	4,186 (7%)	455,196 (31%)	84,497 (14%)	-	-
	09	-	-	-	-	-	-	3,339 (5%)	303,793 (21%)	113,652 (18%)	-	-
	10	-	-	-	-	-	-	46,802 (74%)	367,570 (25%)	374,192 (60%)	-	-
	11	-	-	-	-	-	-	-	-	-	358,211 (52%)	674,485 (50%)
	12	-	-	-	-	-	-	-	-	-	331,247 (48%)	677,614 (50%)
	Chronic heart disease	253,715 (31%)	272,706 (25%)	29,883 (21%)	87,850 (24%)	145,915 (22%)	52,955 (18%)	98 (0%)	2,135 (0%)	658 (0%)	217 (0%)	602 (0%)
	Chronic kidney disease	141,484 (17%)	122,283 (11%)	15,071 (10%)	21,637 (6%)	33,005 (5%)	9,240 (3%)	21 (0%)	378 (0%)	84 (0%)	28 (0%)	63 (0%)
	Chronic liver disease	23,723 (3%)	34,286 (3%)	4,025 (3%)	28,651 (8%)	61,922 (10%)	34,524 (11%)	70 (0%)	1,050 (0%)	420 (0%)	301 (0%)	469 (0%)
	Chronic neurological disease	88,788 (11%)	95,193 (9%)	11,669 (8%)	58,072 (16%)	102,151 (16%)	47,719 (16%)	28 (0%)	581 (0%)	203 (0%)	154 (0%)	371 (0%)
Evidence of	Chronic respiratory disease	88,256 (11%)	108,073 (10%)	10,808 (7%)	33,397 (9%)	59,941 (9%)	19,446 (6%)	84 (0%)	1,652 (0%)	1,169 (0%)	455 (0%)	3,423 (0%)
	Diabetes	149,646 (18%)	183,148 (17%)	24,178 (17%)	125,699 (34%)	184,933 (28%)	69,433 (23%)	126 (0%)	3,045 (0%)	987 (0%)	672 (0%)	2,373 (0%)
	Immunosupp-ression	44,303 (5%)	55,286 (5%)	4,326 (3%)	42,231 (11%)	75,117 (12%)	24,822 (8%)	28 (0%)	588 (0%)	196 (0%)	196 (0%)	399 (0%)
	Learning disability	847 (0%)	1,470 (0%)	322 (0%)	11,585 (3%)	22,645 (3%)	9,912 (3%)	7 (0%)	21 (0%)	7 (0%)	56 (0%)	112 (0%)
	Serious mental illness	6,818 (1%)	10,745 (1%)	3,297 (2%)	17,066 (5%)	31,948 (5%)	32,333 (11%)	84 (0%)	2,324 (0%)	2,492 (0%)	266 (0%)	2,079 (0%)
Pregnancy	yes	-	-	-	742 (0%)	1,890 (0%)	10,717 (4%)	322 (1%)	301 (0%)	1,820 (0%)	8,456 (1%)	44,583 (3%)

[a] And not clinically vulnerable

Attrition due to subsequent vaccination

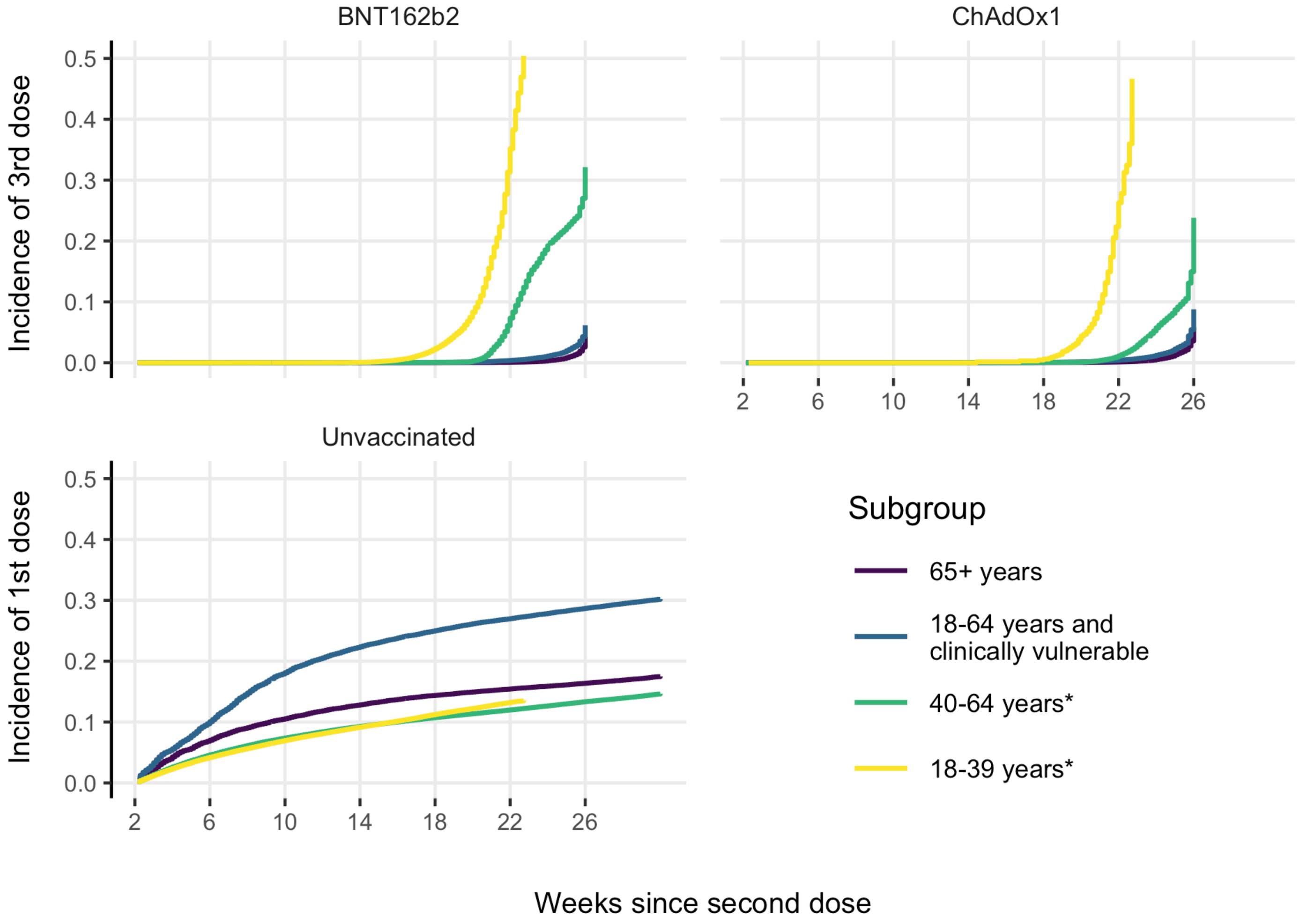

Supplementary Figure 19: Cumulative incidence of subsequent vaccination. *And not clinically vulnerable.

Distribution of follow-up time

The distribution of follow-up time in relation to virus variants is given in Supplementary Figures 20 to 23. We used 1 June 2021 as the approximate date at which the Delta variant became dominant in England (BMJ 2021;373:n1445), 1 December 2021 as the date at which the first cases of Omicron were detected, and 15 December 2021 as the date at which Omicron became dominant (Omicron daily overview: 17 December 2021).

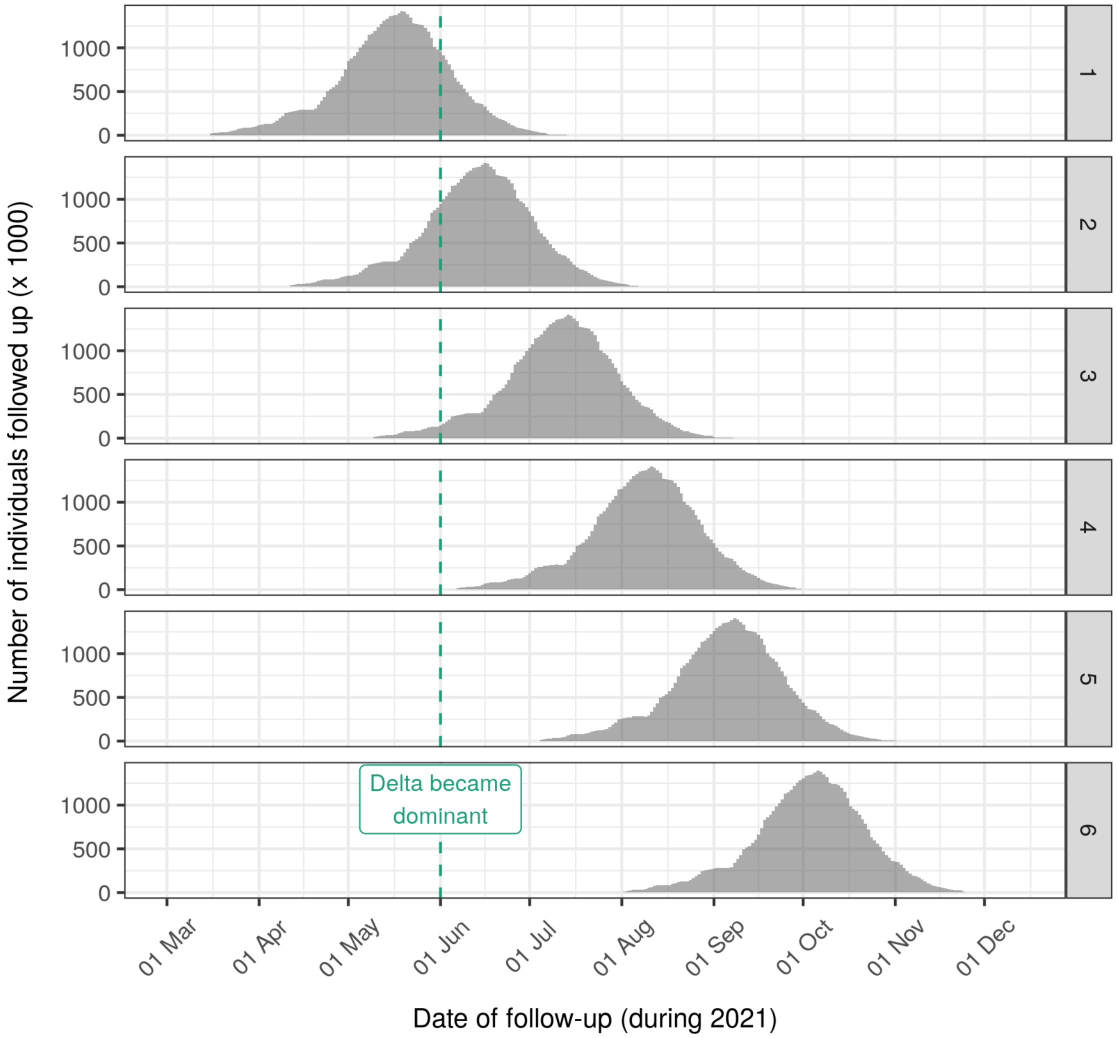

Supplementary Figure 20: Distribution of follow-up time across the six comparison periods in the 65+ years subgroup. Comparison periods (numeric labels on right-hand side of plots) correspond to the following weeks since second vaccine dose: 1 = 3-6 weeks; 2 = 7-10 weeks; 3: 11-14 weeks; 4: 15-18 weeks; 5: 19-22 weeks; 6 = 23-26 weeks.

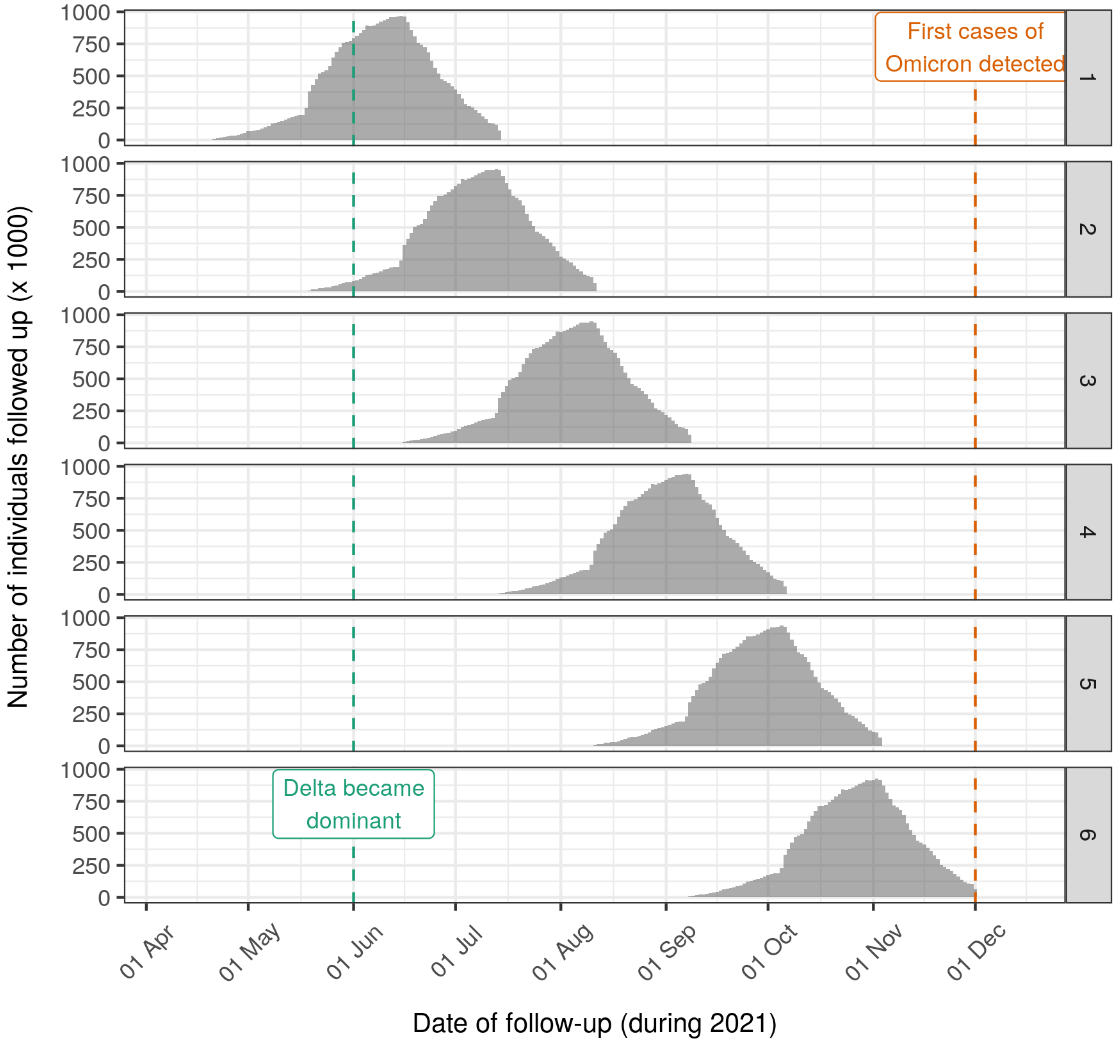

Supplementary Figure 21: Distribution of follow-up time across the six comparison periods in the 18-64 years and clinically vulnerable subgroup. Comparison periods (numeric labels on right-hand side of plots) correspond to the following weeks since second vaccine dose: 1 = 3-6 weeks; 2 = 7-10 weeks; 3: 11-14 weeks; 4: 15-18 weeks; 5: 19-22 weeks; 6 = 23-26 weeks.

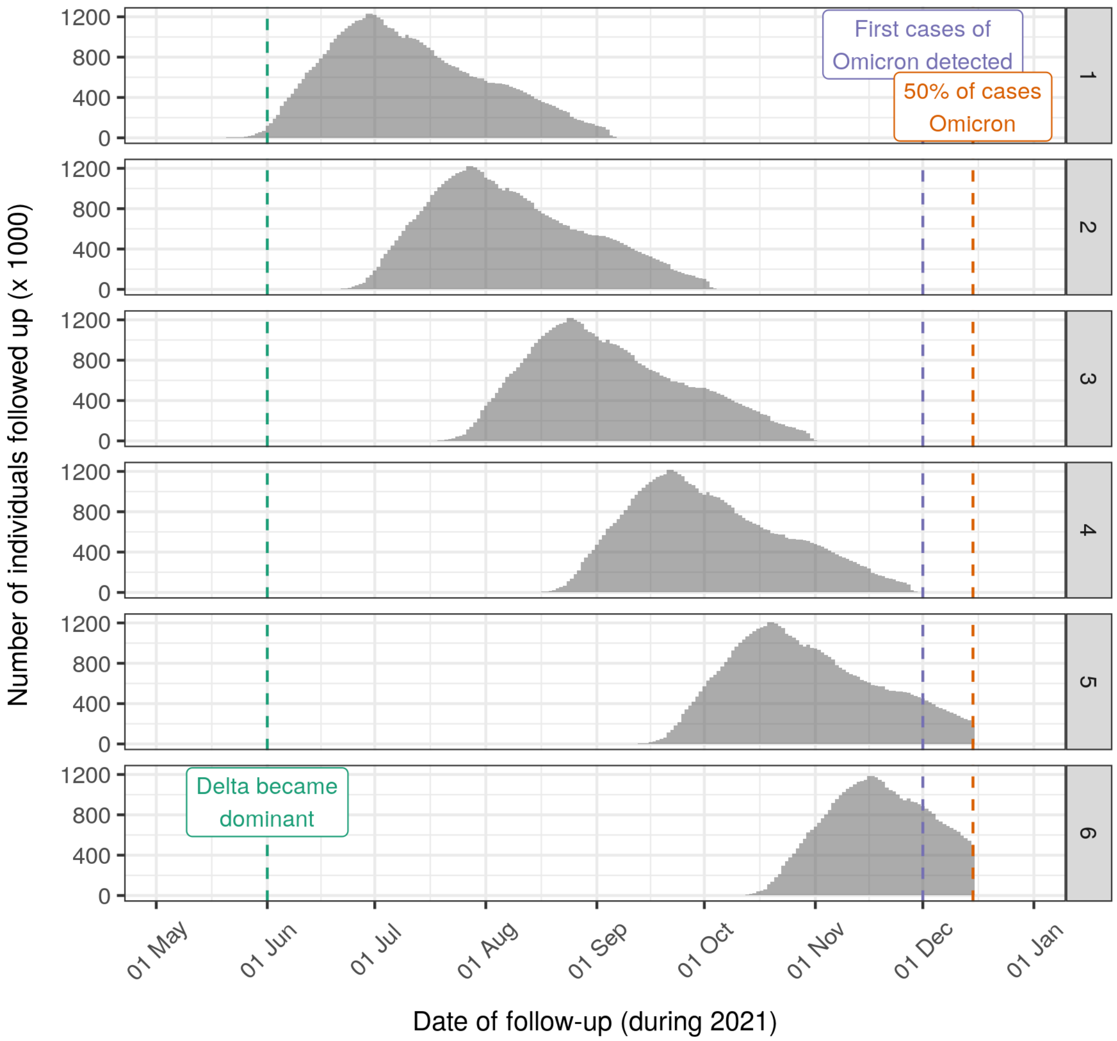

Supplementary Figure 22: Distribution of follow-up time across the six comparison periods in the 40-64 years subgroup. Comparison periods (numeric labels on right-hand side of plots) correspond to the following weeks since second vaccine dose: 1 = 3-6 weeks; 2 = 7-10 weeks; 3: 11-14 weeks; 4: 15-18 weeks; 5: 19-22 weeks; 6 = 23-26 weeks.

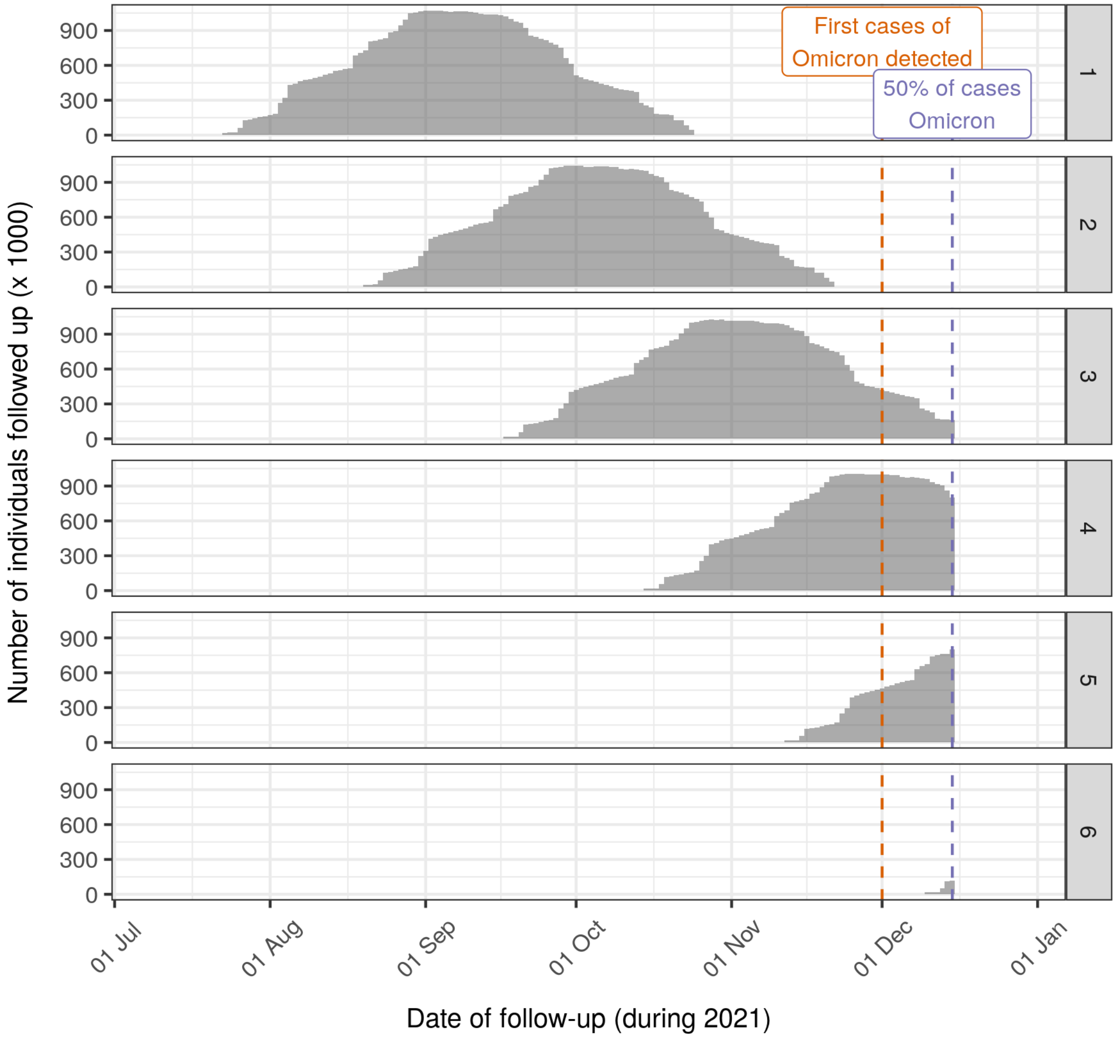

Supplementary Figure 23: Distribution of follow-up time across the six comparison periods in the 18-39 years subgroup. Comparison periods (numeric labels on right-hand side of plots) correspond to the following weeks since second vaccine dose: 1 = 3-6 weeks; 2 = 7-10 weeks; 3: 11-14 weeks; 4: 15-18 weeks; 5: 19-22 weeks; 6 = 23-26 weeks.

Waning vaccine effectiveness in risk-based subgroups

This subsection provides additional results for the waning of vaccine effectiveness across the four risk-based subgroups. Supplementary Table 5 gives the event counts / person-years for each subgroup, outcome and comparison period; Supplementary Tables 6 and 7 give the unadjusted and adjusted hazard ratios (respectively) for each subgroup, outcome and comparison period; Supplementary Table 8 gives the per-comparison-period ratio of adjusted hazard ratios. The unadjusted and adjusted hazard ratios are also plotted in Supplementary Figures 24, 25 and 26 for BNT162b2 vs unvaccinated, ChAdOx1 vs unvaccinated and BNT162b2 vs ChAdOx1 respectively. The unadjusted and adjusted hazard ratios with any SARS-CoV-2 test as the outcome are plotted in Supplementary Figures 27, 28 and Supplementary Figure 29 for BNT162b2 vs unvaccinated, ChAdOx1 vs unvaccinated and BNT162b2 vs ChAdOx1 respectively. The adjusted hazard ratios and lines corresponding to the ratio of hazard ratios with any SARS-CoV-2 test as the outcome are plotted in Supplementary Figure 30 for BNT162b2 vs unvaccinated and ChAdOx1 vs unvaccinated, and Supplementary Figure 31 for BNT162b2 vs ChAdOx1. The hazard ratios corresponding to the covariates in the adjusted models are given in Supplementary Tables 9 to 50.

Supplementary Table 5: Event counts / person-years.

		65+ years			18-64 years and clinically vulnerable			40-64 years[a]			18-39 years[a]	
**Outcome**	**Weeks since 2nd dose**	**BNT162b2**	**ChAdOx1**	**Unvaccinated**	**BNT162b2**	**ChAdOx1**	**Unvaccinated**	**BNT162b2**	**ChAdOx1**	**Unvaccinated**	**BNT162b2**	**Unvaccinated**
COVID-19 hospitalisation	3-6	28 / 63,672	42 / 84,088	77 / 10,935	7 / 28,269	28 / 49,762	168 / 22,966	7 / 4,832	63 / 112,968	532 / 47,788	21 / 52,714	749 / 103,074
	7-10	35 / 63,747	91 / 83,948	112 / 10,191	28 / 28,228	161 / 49,676	462 / 20,541	7 / 4,826	112 / 112,827	588 / 45,100	28 / 52,381	553 / 98,086
	11-14	119 / 63,610	336 / 83,784	196 / 9,625	63 / 28,185	301 / 49,578	602 / 18,578	7 / 4,818	119 / 112,690	560 / 43,740	42 / 52,103	539 / 93,860
	15-18	301 / 63,449	595 / 83,591	315 / 9,358	105 / 28,134	399 / 49,465	504 / 17,460	7 / 4,811	175 / 112,546	504 / 42,456	35 / 41,531	413 / 66,955
	19-22	413 / 63,274	756 / 83,382	322 / 9,028	105 / 28,074	385 / 49,334	427 / 16,784	7 / 4,597	210 / 111,778	504 / 38,859	21 / 12,835	147 / 21,915
	23-26	574 / 63,047	833 / 83,089	336 / 8,884	133 / 27,922	504 / 49,034	448 / 16,222	7 / 1,794	259 / 94,855	378 / 25,431	0 / 50	7 / 554
	Total	1,470 / 380,799	2,653 / 501,882	1,358 / 58,021	441 / 168,812	1,778 / 296,849	2,611 / 112,551	42 / 25,678	938 / 657,664	3,066 / 243,374	147 / 211,614	2,408 / 384,444
COVID-19 death	3-6	7 / 63,675	7 / 84,090	28 / 10,943	0 / 28,270	7 / 49,763	14 / 22,976	0 / 4,832	7 / 112,973	21 / 47,825	0 / 52,716	14 / 103,176
	7-10	7 / 63,752	7 / 83,955	28 / 10,201	0 / 28,230	7 / 49,683	28 / 20,572	0 / 4,826	7 / 112,838	28 / 45,178	0 / 52,385	14 / 98,232
	11-14	14 / 63,619	35 / 83,805	49 / 9,643	7 / 28,191	21 / 49,602	63 / 18,644	0 / 4,819	7 / 112,710	35 / 43,857	0 / 52,109	7 / 94,034
	15-18	42 / 63,471	84 / 83,642	63 / 9,391	14 / 28,146	28 / 49,514	70 / 17,564	0 / 4,812	7 / 112,576	28 / 42,603	0 / 41,538	7 / 67,111
	19-22	98 / 63,318	133 / 83,479	98 / 9,078	14 / 28,092	35 / 49,411	70 / 16,911	0 / 4,598	14 / 111,822	42 / 39,023	0 / 12,838	7 / 21,974
	23-26	112 / 63,120	126 / 83,232	98 / 8,950	14 / 27,948	42 / 49,140	49 / 16,374	0 / 1,795	21 / 94,906	28 / 25,559	0 / 50	0 / 556
	Total	280 / 380,955	392 / 502,203	364 / 58,206	49 / 168,877	140 / 297,113	294 / 113,041	0 / 25,682	63 / 657,825	182 / 244,045	0 / 211,636	49 / 385,083
Positive SARS-CoV-2 test	3-6	105 / 63,663	252 / 84,070	133 / 10,930	147 / 28,259	1,400 / 49,706	1,561 / 22,900	224 / 4,805	8,890 / 112,412	5,880 / 47,310	2,688 / 52,260	16,331 / 100,695
	7-10	322 / 63,727	1,197 / 83,891	231 / 10,181	1,414 / 28,175	5,481 / 49,371	3,318 / 20,317	385 / 4,776	13,405 / 111,385	5,894 / 44,260	4,053 / 51,679	13,783 / 94,796
	11-14	1,078 / 63,545	3,374 / 83,573	420 / 9,603	2,366 / 27,982	7,679 / 48,783	3,878 / 18,141	700 / 4,731	17,402 / 110,123	5,670 / 42,524	7,217 / 50,995	16,142 / 89,674
	15-18	2,275 / 63,277	5,411 / 83,082	581 / 9,318	3,101 / 27,727	8,295 / 48,068	3,045 / 16,833	1,001 / 4,656	20,356 / 108,520	5,747 / 40,920	11,669 / 40,081	13,475 / 63,383
	19-22	3,073 / 62,920	6,433 / 82,462	574 / 8,974	3,332 / 27,437	9,800 / 47,311	2,688 / 16,014	1,358 / 4,371	28,231 / 106,037	5,845 / 37,144	6,188 / 12,089	5,075 / 20,555
	23-26	4,123 / 62,468	8,799 / 81,683	609 / 8,810	5,103 / 26,972	12,726 / 46,179	2,954 / 15,309	406 / 1,715	27,006 / 88,990	4,228 / 24,279	35 / 45	154 / 517
	Total	10,976 / 379,600	25,466 / 498,761	2,548 / 57,816	15,463 / 166,552	45,381 / 289,418	17,444 / 109,514	4,074 / 25,054	115,290 / 637,467	33,264 / 236,437	31,850 / 207,149	64,960 / 369,620
Non-COVID-19 death	3-6	847 / 63,675	812 / 84,090	357 / 10,943	133 / 28,270	189 / 49,763	147 / 22,976	7 / 4,832	91 / 112,973	91 / 47,825	7 / 52,716	21 / 103,176
	7-10	1,092 / 63,752	966 / 83,955	287 / 10,201	133 / 28,230	245 / 49,683	161 / 20,572	7 / 4,826	112 / 112,838	84 / 45,178	7 / 52,385	21 / 98,232
	11-14	1,281 / 63,619	1,148 / 83,805	266 / 9,643	140 / 28,191	280 / 49,602	140 / 18,644	7 / 4,819	126 / 112,710	77 / 43,857	7 / 52,109	28 / 94,034
	15-18	1,330 / 63,471	1,246 / 83,642	294 / 9,391	154 / 28,146	301 / 49,514	112 / 17,564	7 / 4,812	161 / 112,576	70 / 42,603	7 / 41,538	14 / 67,111
	19-22	1,393 / 63,318	1,183 / 83,479	259 / 9,078	140 / 28,092	294 / 49,411	119 / 16,911	14 / 4,598	161 / 111,822	70 / 39,023	0 / 12,838	7 / 21,974
	23-26	1,561 / 63,120	1,358 / 83,232	259 / 8,950	182 / 27,948	308 / 49,140	98 / 16,374	7 / 1,795	154 / 94,906	49 / 25,559	0 / 50	0 / 556
	Total	7,504 / 380,955	6,713 / 502,203	1,722 / 58,206	882 / 168,877	1,617 / 297,113	777 / 113,041	49 / 25,682	805 / 657,825	441 / 244,045	28 / 211,636	91 / 385,083
Any SARS-CoV-2 test	3-6	65,310 / 60,849	99,890 / 79,562	5,936 / 10,430	47,677 / 25,944	91,133 / 45,389	19,929 / 21,301	9,135 / 4,392	241,339 / 101,040	34,993 / 44,712	116,935 / 47,419	99,813 / 93,454
	7-10	73,171 / 60,489	111,041 / 78,930	5,698 / 9,710	59,220 / 25,486	108,122 / 44,434	20,461 / 18,800	10,591 / 4,319	249,354 / 99,916	34,342 / 41,729	117,355 / 46,646	84,399 / 88,423
	11-14	80,465 / 59,981	120,351 / 78,150	5,621 / 9,149	56,518 / 25,344	101,612 / 44,324	19,572 / 16,747	11,102 / 4,249	250,229 / 99,375	33,040 / 40,057	114,527 / 46,275	77,672 / 84,452
	15-18	80,192 / 59,747	118,475 / 77,906	5,761 / 8,871	57,750 / 25,281	107,254 / 43,558	17,808 / 15,533	11,466 / 4,167	273,966 / 96,413	30,947 / 38,621	113,869 / 36,314	60,823 / 60,574
	19-22	85,946 / 59,284	131,397 / 76,887	5,579 / 8,535	63,609 / 24,634	114,597 / 42,433	16,716 / 14,734	12,243 / 3,899	281,904 / 94,053	28,770 / 35,238	53,984 / 10,881	23,877 / 20,007
	23-26	94,416 / 58,489	143,213 / 75,680	5,614 / 8,364	64,771 / 24,155	113,400 / 41,419	15,316 / 14,189	5,516 / 1,551	250,572 / 79,312	20,951 / 23,245	413 / 44	896 / 515
	Total	479,500 / 358,839	724,367 / 467,115	34,209 / 55,059	349,545 / 150,844	636,118 / 261,557	109,802 / 101,304	60,053 / 22,577	1,547,364 / 570,109	183,043 / 223,602	517,083 / 187,579	347,480 / 347,425

[a] And not clinically vulnerable

Supplementary Table 6: Unadjusted hazard ratios for effect of vaccination.

		65+ years			18-64 years and clinically vulnerable			40-64 years[a]			18-39 years[a]
**Outcome**	**Weeks since 2nd dose**	**BNT162b2 vs unvaccinated**	**ChAdOx1 vs unvaccinated**	**BNT162b2 vs ChAdOx1**	**BNT162b2 vs unvaccinated**	**ChAdOx1 vs unvaccinated**	**BNT162b2 vs ChAdOx1**	**BNT162b2 vs unvaccinated**	**ChAdOx1 vs unvaccinated**	**BNT162b2 vs ChAdOx1**	**BNT162b2 vs unvaccinated**
COVID-19 hospitalisation	3-6	0.07 (0.04-0.11)	0.13 (0.08-0.20)	0.54 (0.30-0.96)	0.05 (0.02-0.12)	0.10 (0.06-0.16)	0.51 (0.17-1.54)	-	0.06 (0.04-0.08)	-	0.04 (0.02-0.06)
	7-10	0.07 (0.04-0.10)	0.10 (0.07-0.13)	0.51 (0.33-0.80)	0.05 (0.04-0.08)	0.14 (0.11-0.17)	0.38 (0.24-0.58)	-	0.06 (0.05-0.07)	-	0.09 (0.06-0.13)
	11-14	0.10 (0.08-0.13)	0.15 (0.12-0.19)	0.55 (0.44-0.69)	0.06 (0.05-0.08)	0.17 (0.15-0.20)	0.39 (0.29-0.51)	-	0.06 (0.04-0.07)	-	0.12 (0.08-0.16)
	15-18	0.13 (0.11-0.15)	0.18 (0.15-0.21)	0.62 (0.53-0.73)	0.10 (0.08-0.13)	0.24 (0.21-0.28)	0.45 (0.35-0.56)	-	0.12 (0.10-0.14)	-	0.11 (0.07-0.16)
	19-22	0.15 (0.13-0.18)	0.23 (0.20-0.27)	0.57 (0.49-0.65)	0.12 (0.10-0.16)	0.27 (0.23-0.32)	0.53 (0.42-0.67)	-	0.12 (0.10-0.15)	-	0.20 (0.12-0.32)
	23-26	0.22 (0.19-0.26)	0.28 (0.24-0.33)	0.69 (0.61-0.79)	0.16 (0.13-0.19)	0.33 (0.28-0.37)	0.49 (0.40-0.60)	-	0.16 (0.13-0.19)	-	-
COVID-19 death	3-6	0.02 (0.01-0.07)	-	-	-	-	-	-	-	-	-
	7-10	-	0.03 (0.01-0.09)	-	-	0.08 (0.03-0.25)	-	-	-	-	-
	11-14	0.04 (0.02-0.08)	0.07 (0.04-0.11)	0.57 (0.29-1.14)	-	0.12 (0.07-0.20)	-	-	0.02 (0.01-0.07)	-	-
	15-18	0.09 (0.06-0.14)	0.13 (0.08-0.19)	0.58 (0.35-0.94)	0.07 (0.03-0.14)	0.15 (0.09-0.23)	0.51 (0.21-1.23)	-	0.04 (0.02-0.10)	-	-
	19-22	0.11 (0.08-0.15)	0.12 (0.09-0.17)	0.67 (0.50-0.91)	0.16 (0.09-0.29)	0.18 (0.11-0.28)	0.99 (0.51-1.90)	-	0.05 (0.03-0.10)	-	-
	23-26	0.13 (0.10-0.18)	0.19 (0.13-0.26)	0.64 (0.48-0.86)	0.19 (0.09-0.38)	0.27 (0.17-0.42)	0.59 (0.30-1.14)	-	0.10 (0.05-0.21)	-	-
Positive SARS-CoV-2 test	3-6	0.20 (0.15-0.28)	0.51 (0.39-0.67)	0.67 (0.52-0.87)	0.17 (0.14-0.21)	0.61 (0.56-0.66)	0.33 (0.27-0.39)	0.33 (0.29-0.38)	0.91 (0.88-0.95)	0.37 (0.32-0.42)	0.31 (0.30-0.32)
	7-10	0.40 (0.32-0.48)	0.76 (0.65-0.90)	0.55 (0.48-0.62)	0.31 (0.29-0.34)	0.65 (0.62-0.68)	0.50 (0.47-0.53)	0.56 (0.50-0.63)	1.06 (1.02-1.10)	0.49 (0.44-0.54)	0.53 (0.51-0.55)
	11-14	0.49 (0.43-0.56)	0.82 (0.73-0.93)	0.61 (0.57-0.66)	0.38 (0.36-0.40)	0.73 (0.70-0.77)	0.56 (0.54-0.59)	0.94 (0.86-1.02)	1.46 (1.41-1.51)	0.57 (0.53-0.61)	0.75 (0.73-0.77)
	15-18	0.64 (0.57-0.71)	0.95 (0.86-1.04)	0.70 (0.66-0.73)	0.54 (0.51-0.57)	0.93 (0.89-0.98)	0.62 (0.59-0.65)	1.24 (1.15-1.33)	1.86 (1.80-1.92)	0.63 (0.59-0.67)	1.26 (1.23-1.29)
	19-22	0.75 (0.68-0.84)	1.18 (1.07-1.30)	0.71 (0.68-0.74)	0.80 (0.75-0.85)	1.24 (1.19-1.30)	0.66 (0.63-0.69)	1.54 (1.44-1.65)	2.14 (2.07-2.21)	0.68 (0.64-0.72)	1.91 (1.84-1.99)
	23-26	1.08 (0.97-1.19)	1.59 (1.45-1.75)	0.75 (0.72-0.78)	0.93 (0.88-0.98)	1.41 (1.35-1.47)	0.68 (0.66-0.70)	1.36 (1.21-1.52)	2.27 (2.19-2.36)	0.58 (0.53-0.64)	2.63 (1.75-3.97)
Non-COVID-19 death	3-6	0.33 (0.28-0.38)	0.46 (0.39-0.55)	0.80 (0.71-0.91)	0.68 (0.52-0.90)	0.52 (0.41-0.66)	1.30 (1.03-1.65)	-	0.29 (0.20-0.41)	-	-
	7-10	0.46 (0.40-0.53)	0.59 (0.49-0.72)	0.84 (0.75-0.94)	0.51 (0.39-0.67)	0.49 (0.40-0.61)	1.03 (0.82-1.29)	-	0.29 (0.21-0.41)	-	-
	11-14	0.55 (0.47-0.64)	0.69 (0.58-0.82)	0.86 (0.78-0.94)	0.56 (0.43-0.73)	0.57 (0.46-0.71)	0.95 (0.77-1.18)	-	0.49 (0.36-0.68)	-	-
	15-18	0.52 (0.45-0.60)	0.68 (0.58-0.81)	0.82 (0.74-0.90)	0.79 (0.60-1.05)	0.87 (0.68-1.11)	1.00 (0.81-1.24)	-	0.61 (0.42-0.87)	-	-
	19-22	0.62 (0.53-0.72)	0.70 (0.58-0.85)	0.93 (0.85-1.03)	0.68 (0.51-0.90)	0.75 (0.59-0.96)	0.90 (0.72-1.11)	-	0.55 (0.39-0.79)	-	-
	23-26	0.62 (0.53-0.71)	0.75 (0.63-0.89)	0.90 (0.83-0.99)	0.96 (0.73-1.28)	1.02 (0.78-1.32)	1.04 (0.85-1.26)	-	0.56 (0.40-0.80)	-	-
Any SARS-CoV-2 test	3-6	2.14 (2.08-2.20)	2.84 (2.76-2.93)	0.86 (0.85-0.87)	2.24 (2.20-2.28)	2.50 (2.46-2.55)	0.77 (0.76-0.78)	2.91 (2.83-2.99)	3.75 (3.70-3.79)	0.92 (0.90-0.94)	2.35 (2.33-2.37)
	7-10	2.44 (2.37-2.52)	2.98 (2.89-3.07)	0.86 (0.85-0.87)	2.09 (2.05-2.13)	2.34 (2.31-2.38)	0.78 (0.78-0.79)	2.96 (2.89-3.04)	3.52 (3.47-3.56)	0.89 (0.87-0.91)	2.73 (2.70-2.75)
	11-14	2.48 (2.40-2.55)	2.96 (2.87-3.05)	0.87 (0.86-0.88)	1.99 (1.96-2.03)	2.20 (2.17-2.24)	0.82 (0.81-0.83)	3.32 (3.24-3.41)	3.68 (3.63-3.73)	0.91 (0.89-0.93)	2.81 (2.78-2.84)
	15-18	2.42 (2.35-2.50)	2.90 (2.82-2.99)	0.86 (0.86-0.87)	1.97 (1.93-2.00)	2.25 (2.22-2.29)	0.82 (0.81-0.83)	3.62 (3.53-3.71)	4.09 (4.04-4.14)	0.92 (0.90-0.94)	3.22 (3.19-3.26)
	19-22	2.56 (2.48-2.64)	3.12 (3.02-3.21)	0.87 (0.86-0.88)	2.25 (2.20-2.29)	2.53 (2.49-2.58)	0.82 (0.81-0.83)	3.81 (3.72-3.91)	4.34 (4.28-4.40)	0.91 (0.90-0.93)	4.53 (4.46-4.61)
	23-26	2.77 (2.68-2.85)	3.31 (3.21-3.41)	0.88 (0.87-0.89)	2.44 (2.39-2.49)	2.72 (2.67-2.77)	0.82 (0.82-0.83)	4.33 (4.18-4.49)	4.96 (4.88-5.04)	0.84 (0.82-0.86)	5.97 (5.25-6.77)

[a] And not clinically vulnerable

Supplementary Table 7: Adjusted hazard ratios for effect of vaccination.

		65+ years			18-64 years and clinically vulnerable			40-64 years[a]			18-39 years[a]
**Outcome**	**Weeks since 2nd dose**	**BNT162b2 vs unvaccinated**	**ChAdOx1 vs unvaccinated**	**BNT162b2 vs ChAdOx1**	**BNT162b2 vs unvaccinated**	**ChAdOx1 vs unvaccinated**	**BNT162b2 vs ChAdOx1**	**BNT162b2 vs unvaccinated**	**ChAdOx1 vs unvaccinated**	**BNT162b2 vs ChAdOx1**	**BNT162b2 vs unvaccinated**
COVID-19 hospitalisation	3-6	0.08 (0.05-0.13)	0.13 (0.08-0.23)	0.55 (0.31-0.98)	0.05 (0.02-0.12)	0.09 (0.06-0.14)	0.51 (0.17-1.56)	-	0.05 (0.04-0.07)	-	0.04 (0.02-0.07)
	7-10	0.07 (0.04-0.11)	0.11 (0.07-0.15)	0.51 (0.33-0.79)	0.04 (0.02-0.06)	0.10 (0.08-0.13)	0.36 (0.23-0.55)	-	0.05 (0.04-0.07)	-	0.09 (0.06-0.14)
	11-14	0.09 (0.07-0.12)	0.09 (0.07-0.11)	0.55 (0.44-0.68)	0.05 (0.04-0.06)	0.14 (0.12-0.16)	0.38 (0.29-0.50)	-	0.05 (0.04-0.07)	-	0.11 (0.08-0.16)
	15-18	0.13 (0.10-0.16)	0.18 (0.15-0.22)	0.63 (0.54-0.74)	0.08 (0.06-0.10)	0.19 (0.16-0.22)	0.44 (0.35-0.55)	-	0.09 (0.07-0.12)	-	0.10 (0.07-0.15)
	19-22	0.15 (0.12-0.19)	0.21 (0.18-0.26)	0.57 (0.50-0.65)	0.08 (0.06-0.10)	0.19 (0.16-0.23)	0.51 (0.40-0.65)	-	0.11 (0.09-0.14)	-	0.18 (0.10-0.29)
	23-26	0.20 (0.17-0.25)	0.25 (0.21-0.29)	0.69 (0.61-0.78)	0.09 (0.07-0.12)	0.23 (0.19-0.26)	0.47 (0.38-0.57)	-	0.12 (0.10-0.15)	-	-
COVID-19 death	3-6	0.02 (0.01-0.07)	-	-	-	-	-	-	-	-	-
	7-10	-	0.04 (0.02-0.09)	-	-	0.05 (0.02-0.16)	-	-	-	-	-
	11-14	-	-	-	-	0.07 (0.04-0.13)	-	-	0.02 (0.01-0.07)	-	-
	15-18	0.07 (0.04-0.13)	0.11 (0.07-0.18)	0.58 (0.36-0.94)	0.04 (0.02-0.09)	0.10 (0.06-0.15)	0.49 (0.20-1.18)	-	0.04 (0.01-0.09)	-	-
	19-22	0.10 (0.07-0.15)	0.11 (0.08-0.16)	0.67 (0.49-0.90)	0.10 (0.05-0.18)	0.11 (0.07-0.19)	1.00 (0.52-1.93)	-	0.05 (0.03-0.10)	-	-
	23-26	0.12 (0.08-0.17)	0.15 (0.11-0.21)	0.65 (0.48-0.86)	0.08 (0.04-0.18)	0.13 (0.08-0.20)	0.54 (0.28-1.05)	-	0.10 (0.04-0.22)	-	-
Positive SARS-CoV-2 test	3-6	0.19 (0.13-0.27)	0.43 (0.32-0.58)	0.68 (0.52-0.88)	0.22 (0.18-0.27)	0.75 (0.69-0.82)	0.35 (0.29-0.41)	0.27 (0.23-0.31)	0.78 (0.75-0.81)	0.37 (0.33-0.43)	0.24 (0.23-0.26)
	7-10	0.35 (0.28-0.44)	0.70 (0.59-0.84)	0.55 (0.48-0.62)	0.38 (0.35-0.41)	0.77 (0.73-0.81)	0.52 (0.49-0.56)	0.45 (0.41-0.51)	0.93 (0.90-0.97)	0.50 (0.45-0.55)	0.41 (0.40-0.43)
	11-14	0.41 (0.35-0.47)	0.70 (0.62-0.80)	0.61 (0.57-0.66)	0.45 (0.42-0.48)	0.85 (0.81-0.89)	0.58 (0.56-0.61)	0.75 (0.68-0.82)	1.25 (1.20-1.30)	0.58 (0.53-0.62)	0.58 (0.56-0.60)
	15-18	0.56 (0.50-0.64)	0.82 (0.73-0.91)	0.70 (0.66-0.73)	0.57 (0.53-0.61)	0.99 (0.94-1.04)	0.64 (0.61-0.67)	0.95 (0.88-1.02)	1.54 (1.49-1.60)	0.64 (0.60-0.68)	0.96 (0.93-0.99)
	19-22	0.66 (0.58-0.74)	1.00 (0.90-1.11)	0.71 (0.68-0.74)	0.81 (0.76-0.87)	1.22 (1.16-1.28)	0.68 (0.65-0.71)	1.21 (1.13-1.29)	1.75 (1.69-1.81)	0.69 (0.65-0.73)	1.42 (1.36-1.49)
	23-26	0.87 (0.78-0.97)	1.23 (1.11-1.36)	0.75 (0.72-0.78)	0.90 (0.84-0.95)	1.35 (1.29-1.41)	0.70 (0.67-0.72)	1.01 (0.90-1.14)	1.86 (1.79-1.93)	0.59 (0.54-0.65)	1.72 (1.11-2.68)
Non-COVID-19 death	3-6	0.29 (0.24-0.35)	0.36 (0.30-0.44)	0.84 (0.74-0.95)	0.47 (0.34-0.64)	0.36 (0.27-0.46)	1.24 (0.97-1.57)	-	0.29 (0.20-0.44)	-	-
	7-10	0.42 (0.35-0.50)	0.52 (0.42-0.64)	0.87 (0.78-0.97)	0.30 (0.22-0.42)	0.30 (0.24-0.39)	0.98 (0.78-1.23)	-	0.33 (0.23-0.47)	-	-
	11-14	0.51 (0.43-0.62)	0.64 (0.53-0.79)	0.88 (0.80-0.97)	0.32 (0.23-0.44)	0.36 (0.28-0.46)	0.91 (0.73-1.13)	-	0.51 (0.36-0.72)	-	-
	15-18	0.49 (0.41-0.59)	0.61 (0.51-0.74)	0.84 (0.77-0.92)	0.46 (0.33-0.64)	0.63 (0.48-0.84)	0.96 (0.78-1.18)	-	0.65 (0.44-0.95)	-	-
	19-22	0.59 (0.49-0.71)	0.68 (0.56-0.84)	0.96 (0.87-1.06)	0.43 (0.31-0.60)	0.51 (0.39-0.66)	0.85 (0.69-1.06)	-	0.64 (0.44-0.93)	-	-
	23-26	0.58 (0.48-0.69)	0.74 (0.61-0.90)	0.93 (0.85-1.02)	0.55 (0.40-0.76)	0.70 (0.52-0.93)	0.98 (0.80-1.19)	-	0.64 (0.43-0.95)	-	-
Any SARS-CoV-2 test	3-6	1.55 (1.50-1.61)	2.01 (1.95-2.08)	0.86 (0.85-0.87)	2.04 (2.00-2.09)	2.23 (2.19-2.27)	0.79 (0.78-0.79)	2.09 (2.03-2.15)	2.79 (2.75-2.83)	0.93 (0.91-0.95)	1.83 (1.81-1.85)
	7-10	1.78 (1.73-1.84)	2.09 (2.03-2.16)	0.87 (0.86-0.88)	1.94 (1.90-1.98)	2.10 (2.07-2.14)	0.80 (0.79-0.81)	2.15 (2.09-2.21)	2.67 (2.63-2.70)	0.90 (0.88-0.92)	2.10 (2.07-2.12)
	11-14	1.81 (1.75-1.87)	2.12 (2.05-2.19)	0.87 (0.87-0.88)	1.83 (1.79-1.87)	1.98 (1.95-2.02)	0.83 (0.82-0.84)	2.41 (2.35-2.48)	2.82 (2.78-2.85)	0.92 (0.90-0.94)	2.14 (2.11-2.16)
	15-18	1.81 (1.75-1.87)	2.10 (2.03-2.17)	0.87 (0.86-0.88)	1.77 (1.74-1.81)	2.00 (1.97-2.04)	0.83 (0.82-0.84)	2.59 (2.52-2.67)	3.10 (3.06-3.15)	0.92 (0.91-0.94)	2.41 (2.38-2.44)
	19-22	1.90 (1.84-1.96)	2.25 (2.18-2.33)	0.87 (0.87-0.88)	1.99 (1.95-2.03)	2.20 (2.16-2.24)	0.84 (0.83-0.85)	2.72 (2.65-2.80)	3.29 (3.24-3.33)	0.92 (0.90-0.94)	3.14 (3.08-3.20)
	23-26	2.06 (2.00-2.13)	2.36 (2.29-2.44)	0.89 (0.88-0.90)	2.09 (2.04-2.13)	2.31 (2.27-2.36)	0.83 (0.83-0.84)	2.93 (2.82-3.05)	3.70 (3.64-3.76)	0.85 (0.82-0.87)	3.06 (2.66-3.53)

[a] And not clinically vulnerable

Supplementary Table 8: Per-comparison-period ratio of adjusted hazard ratios for effect of vaccination.

	65+ years			18-64 years and clinically vulnerable			40-64 years[a]			18-39 years[a]
**Outcome**	**BNT162b2 vs unvaccinated**	**ChAdOx1 vs unvaccinated**	**BNT162b2 vs ChAdOx1**	**BNT162b2 vs unvaccinated**	**ChAdOx1 vs unvaccinated**	**BNT162b2 vs ChAdOx1**	**BNT162b2 vs unvaccinated**	**ChAdOx1 vs unvaccinated**	**BNT162b2 vs ChAdOx1**	**BNT162b2 vs unvaccinated**
COVID-19 hospitalisation	1.26 (1.17-1.36)	1.23 (1.07-1.40)	1.06 (0.99-1.13)	1.23 (1.13-1.35)	1.20 (1.14-1.27)	1.07 (0.98-1.16)	-	1.24 (1.16-1.33)	-	1.31 (1.10-1.57)
COVID-19 death	1.34 (1.09-1.64)	1.31 (1.07-1.61)	1.03 (0.79-1.35)	1.38 (0.77-2.47)	1.21 (0.99-1.49)	1.02 (0.46-2.26)	-	1.66 (1.08-2.55)	-	-
Positive SARS-CoV-2 test	1.29 (1.23-1.35)	1.19 (1.14-1.24)	1.06 (1.03-1.09)	1.30 (1.23-1.36)	1.14 (1.11-1.17)	1.12 (1.05-1.18)	1.32 (1.17-1.49)	1.20 (1.16-1.25)	1.10 (1.03-1.17)	1.54 (1.50-1.59)
Non-COVID-19 death	1.13 (1.07-1.21)	1.13 (1.07-1.20)	1.02 (1.00-1.05)	1.07 (0.96-1.19)	1.17 (1.07-1.29)	0.96 (0.91-1.01)	1.07 (0.77-1.47)	1.19 (1.09-1.31)	1.07 (0.77-1.49)	0.91 (0.56-1.46)
Any SARS-CoV-2 test	1.05 (1.03-1.07)	1.03 (1.02-1.04)	1.00 (1.00-1.01)	1.00 (0.97-1.04)	1.01 (0.98-1.04)	1.01 (1.01-1.02)	1.07 (1.06-1.08)	1.06 (1.04-1.09)	0.99 (0.98-1.00)	1.12 (1.08-1.16)

[a] And not clinically vulnerable

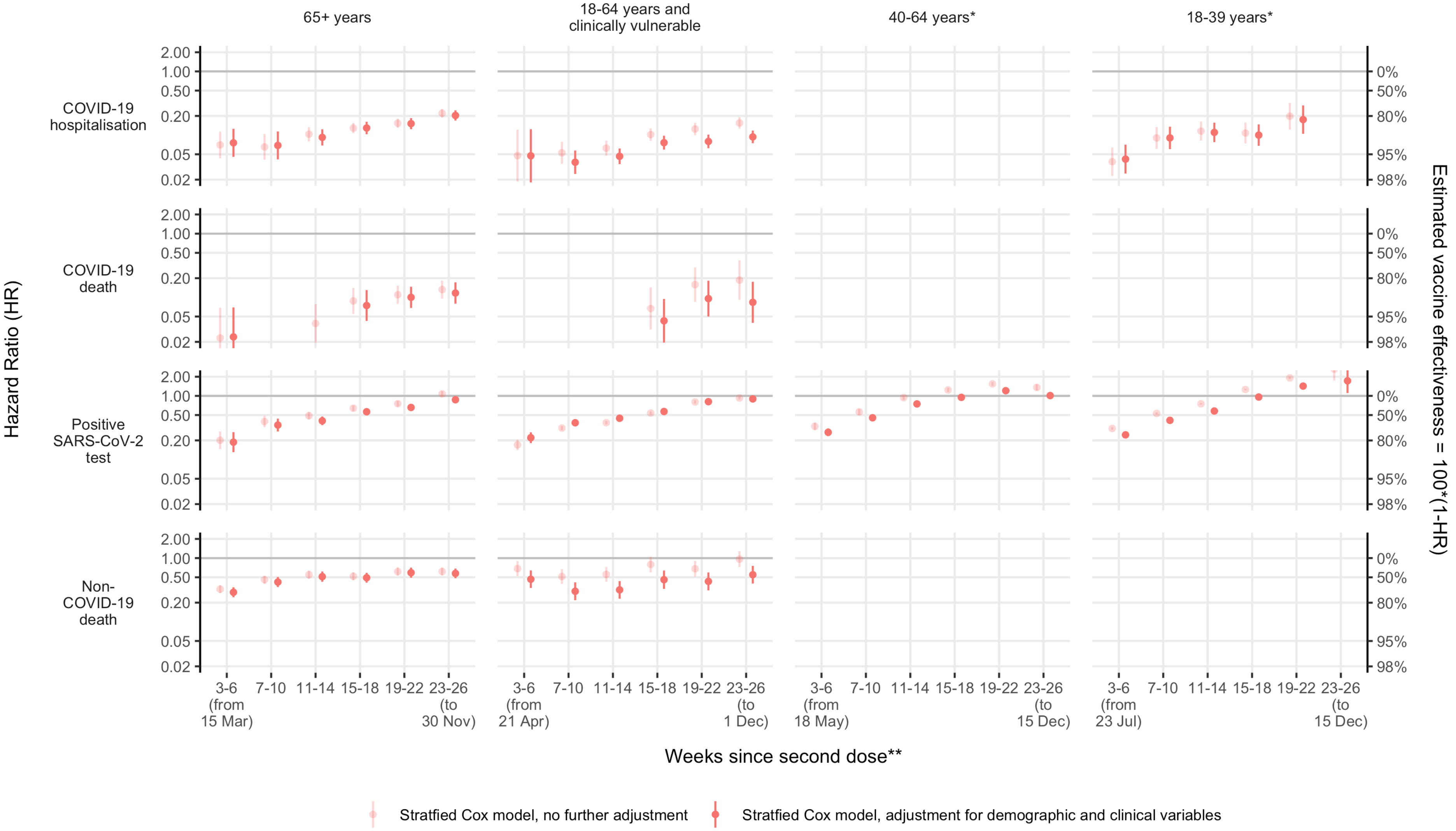

Supplementary Figure 24: Unadjusted and adjusted hazard ratios for BNT162b2 vs unvaccinated

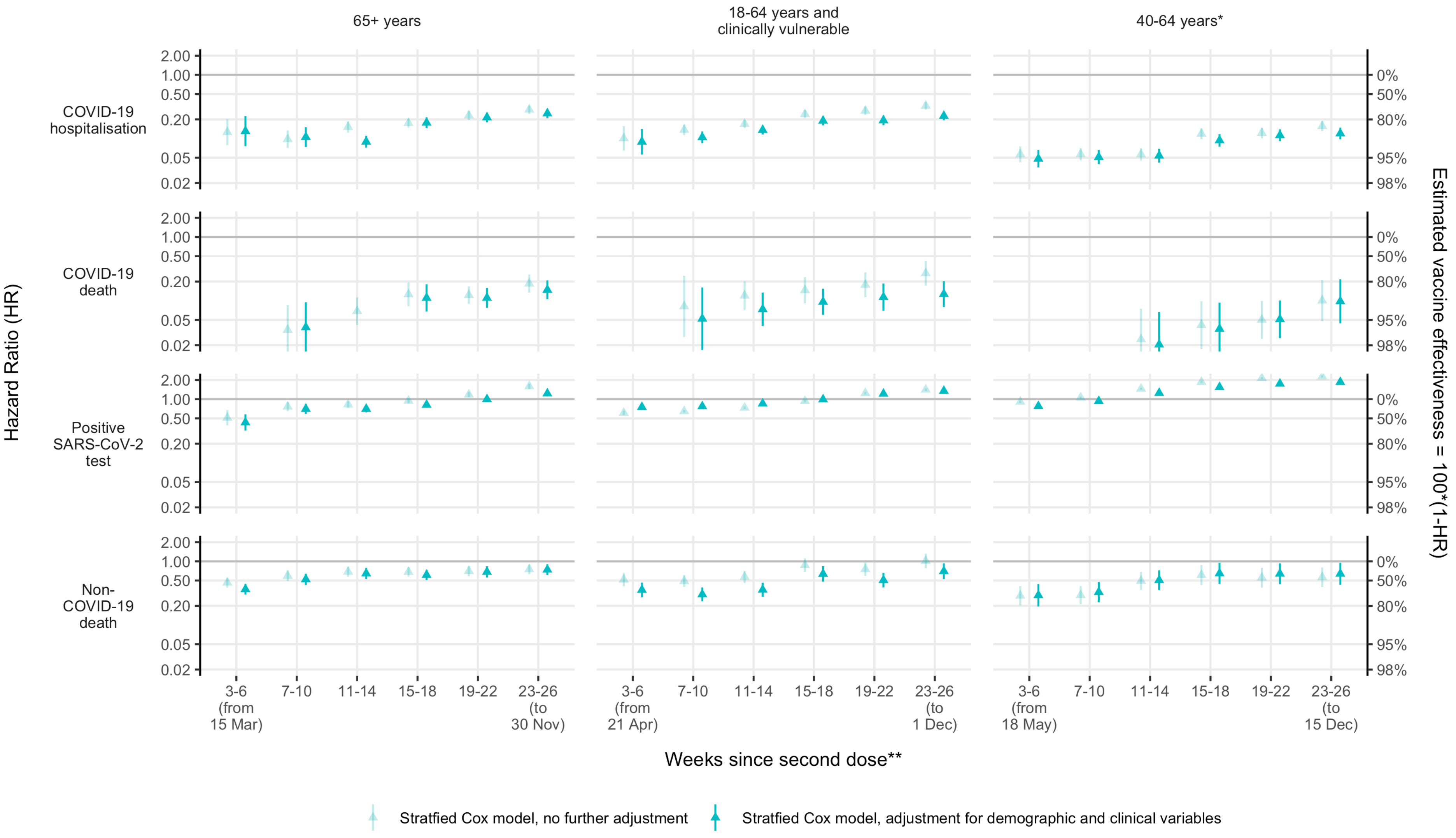

Supplementary Figure 25: Unadjusted and adjusted hazard ratios for ChAdOx1 vs unvaccinated

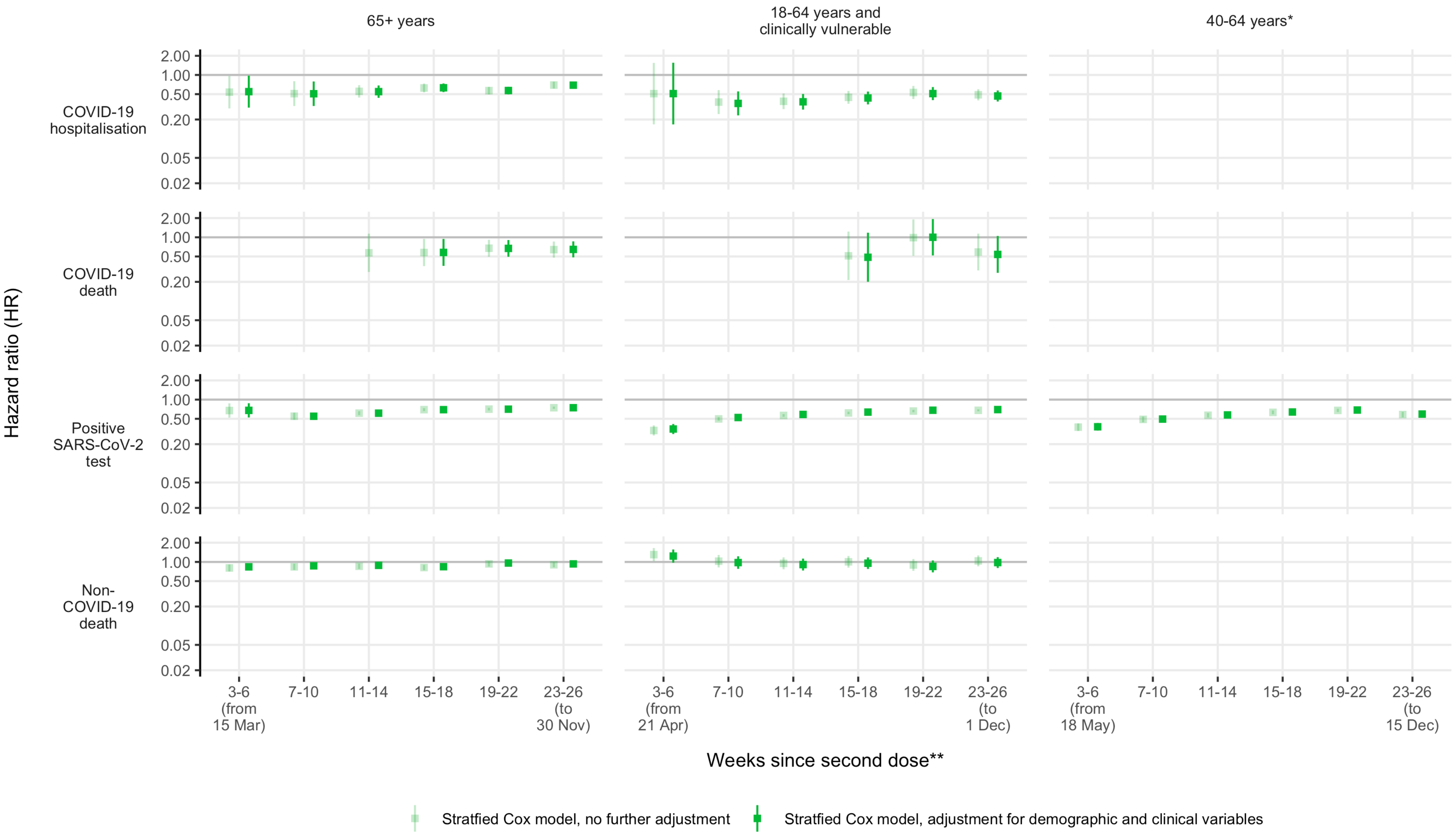

Supplementary Figure 26: Unadjusted and adjusted hazard ratios for BNT162b2 vs ChAdOx1

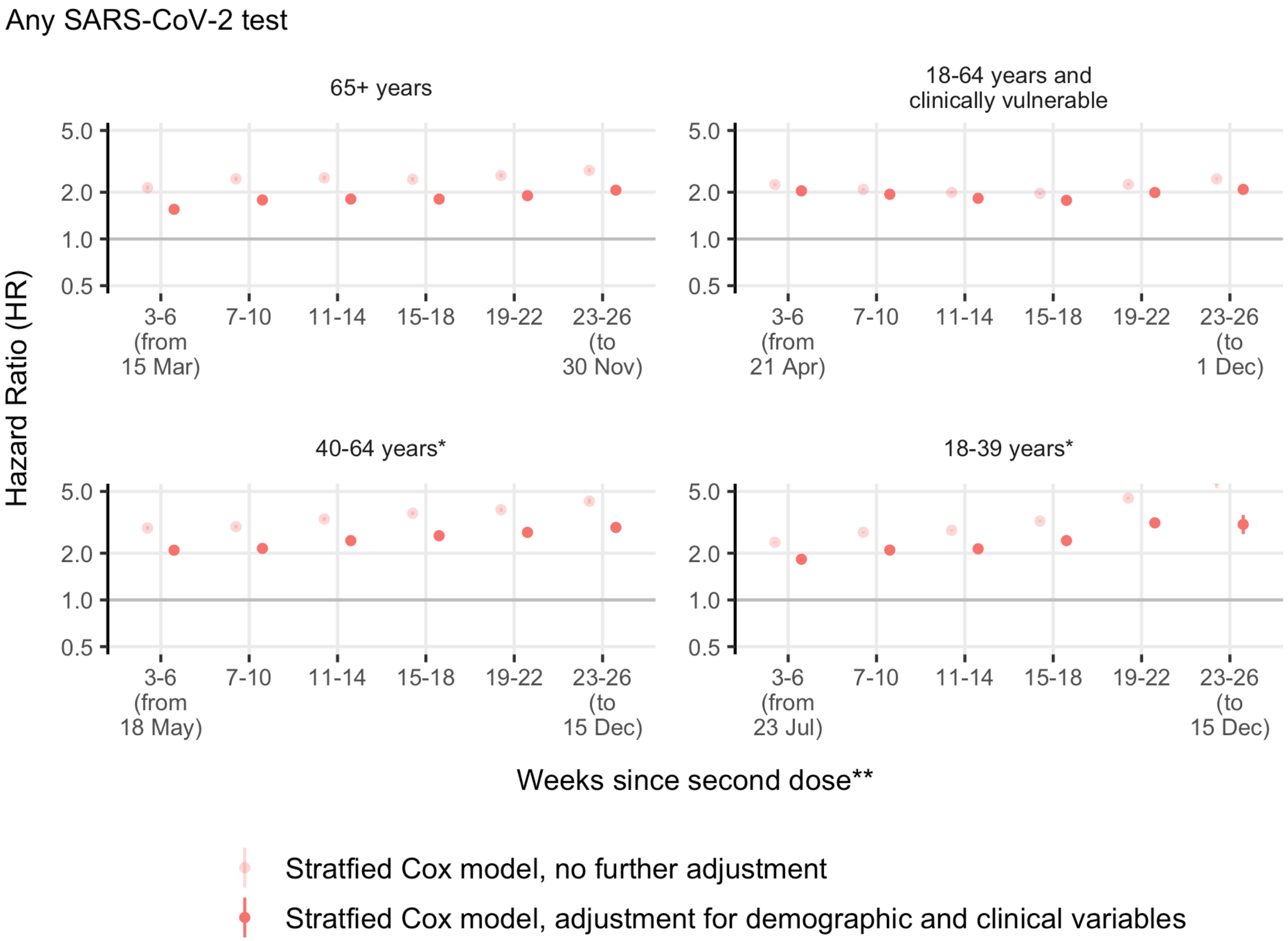

Supplementary Figure 27: Unadjusted and adjusted hazard ratios for any SARS-CoV-2 test for BNT162b2 vs unvaccinated

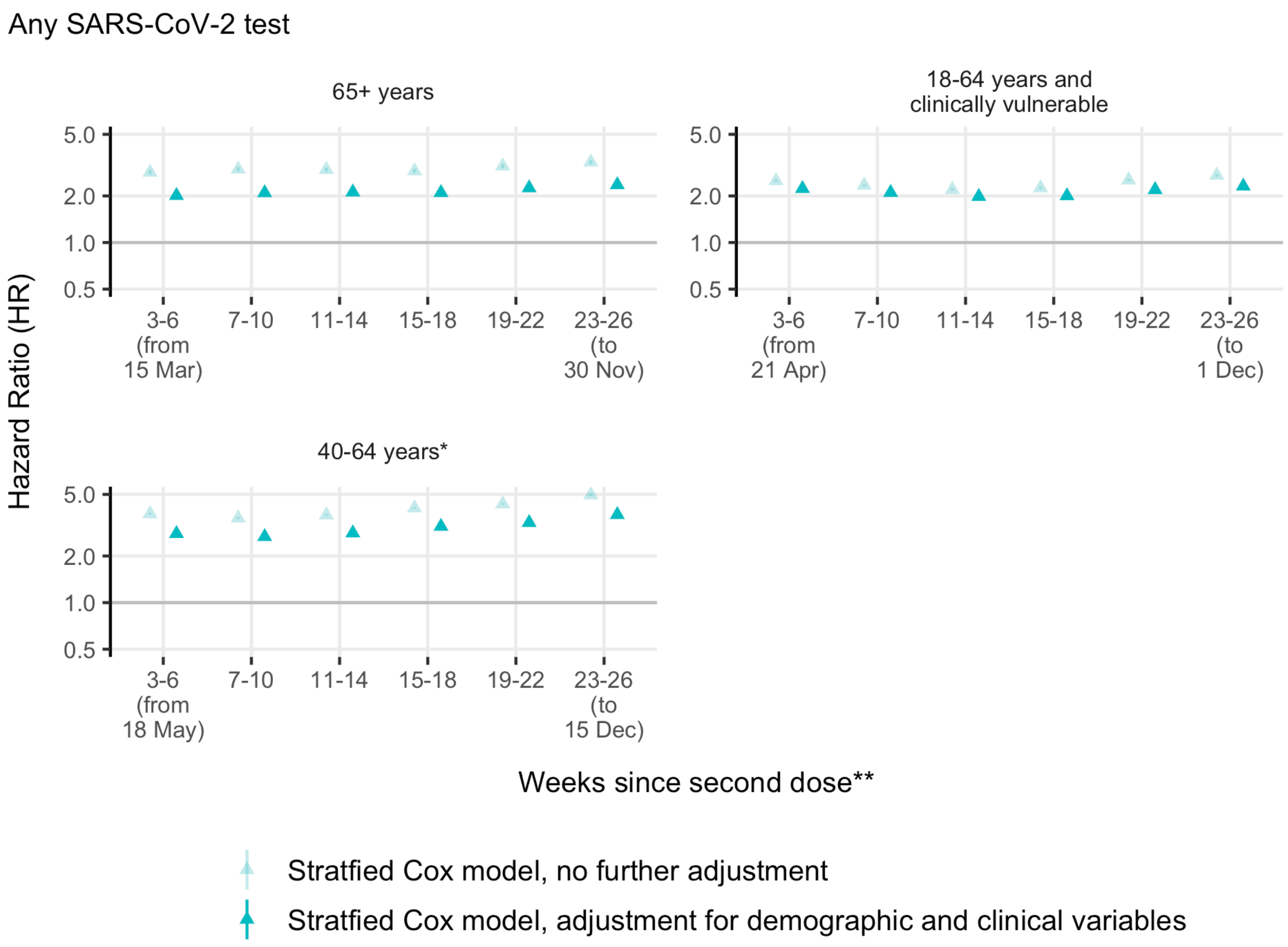

Supplementary Figure 28: Unadjusted and adjusted hazard ratios for any SARS-CoV-2 test for ChAdOx1 vs unvaccinated

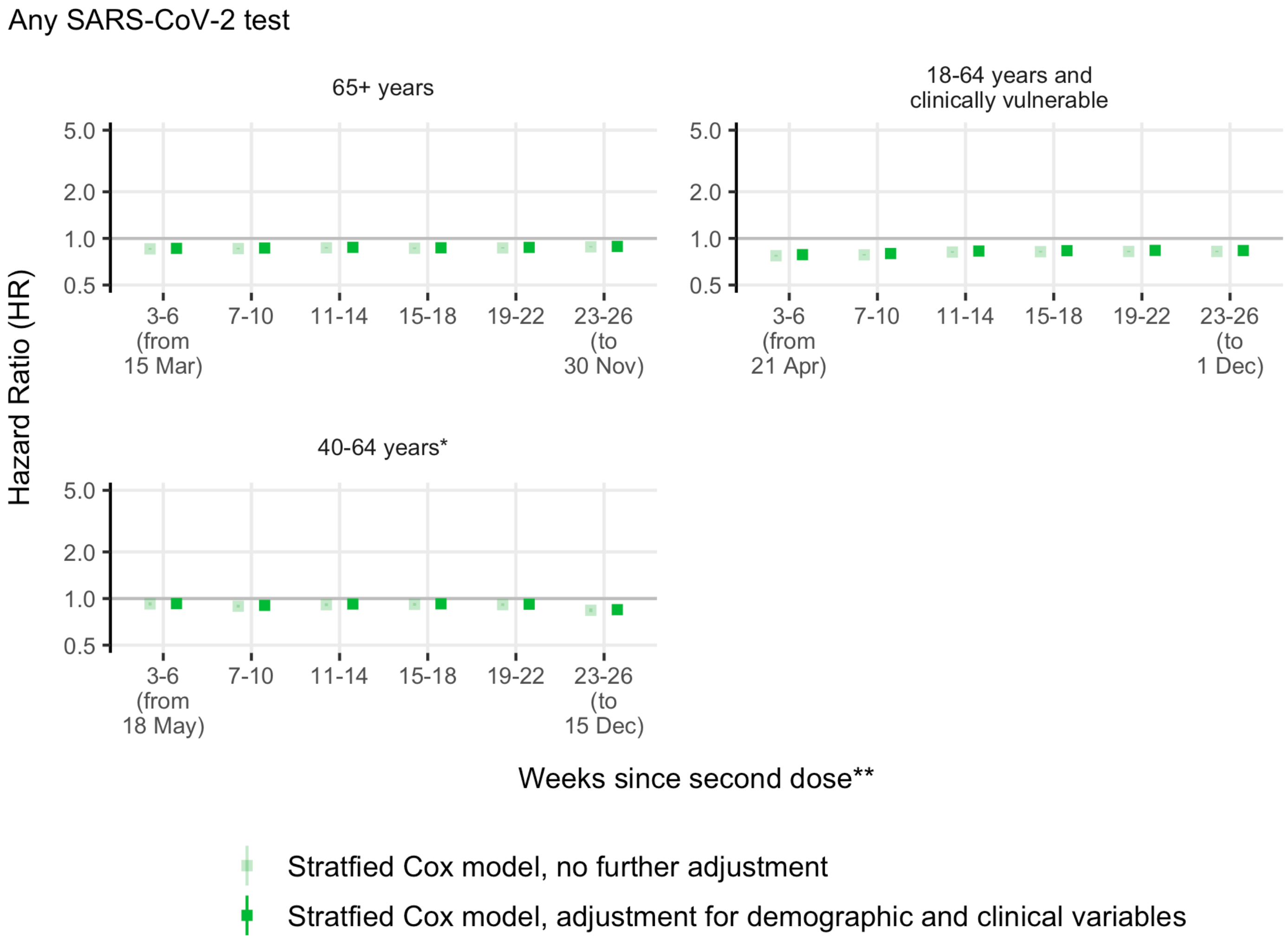

Supplementary Figure 29: Unadjusted and adjusted hazard ratios for any SARS-CoV-2 test for BNT162b2 vs ChAdOx1

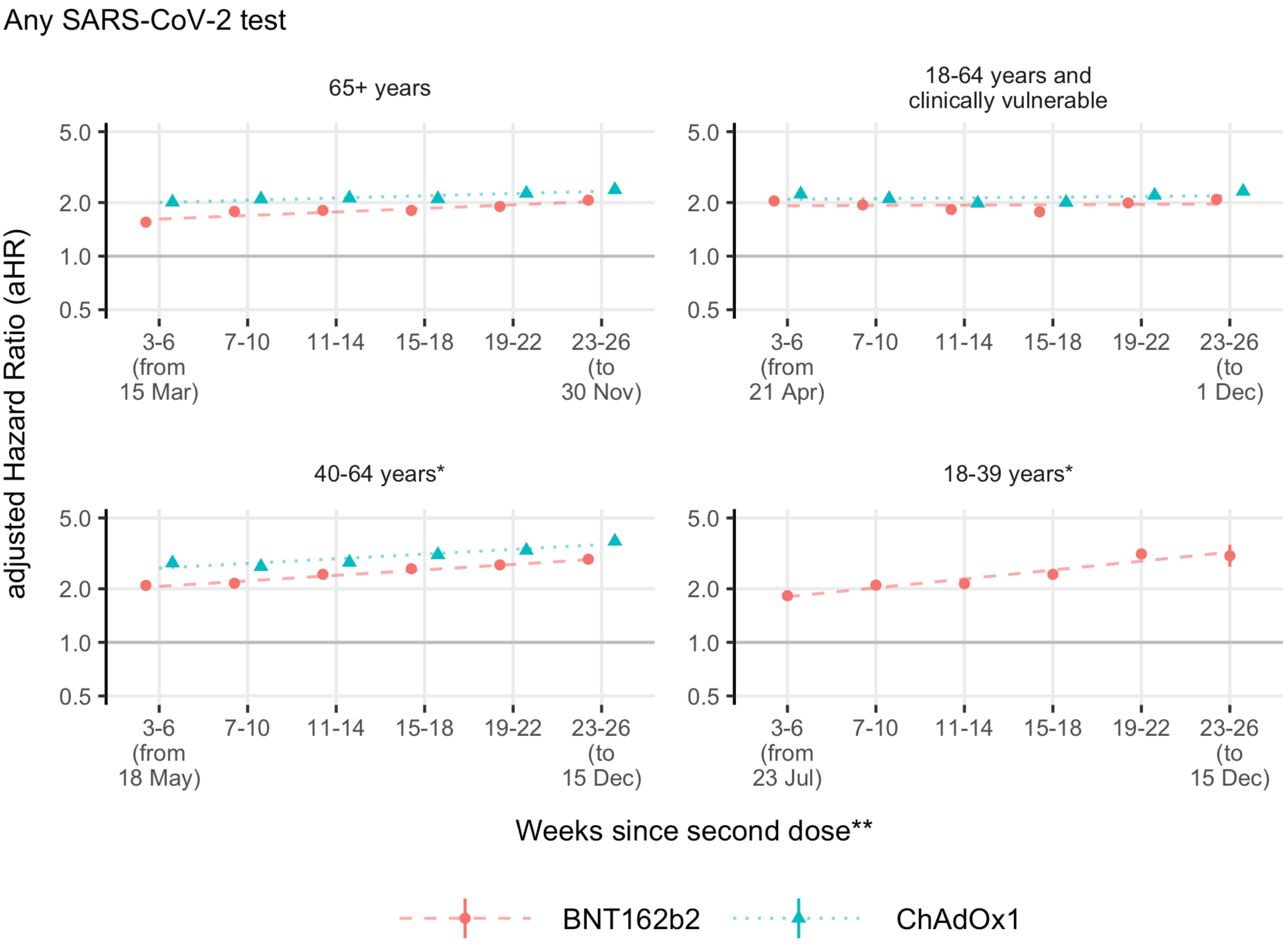

Supplementary Figure 30: Adjusted hazard ratios for any SARS-CoV-2 test for BNT162b2 and ChAdOx1 vs unvaccinated

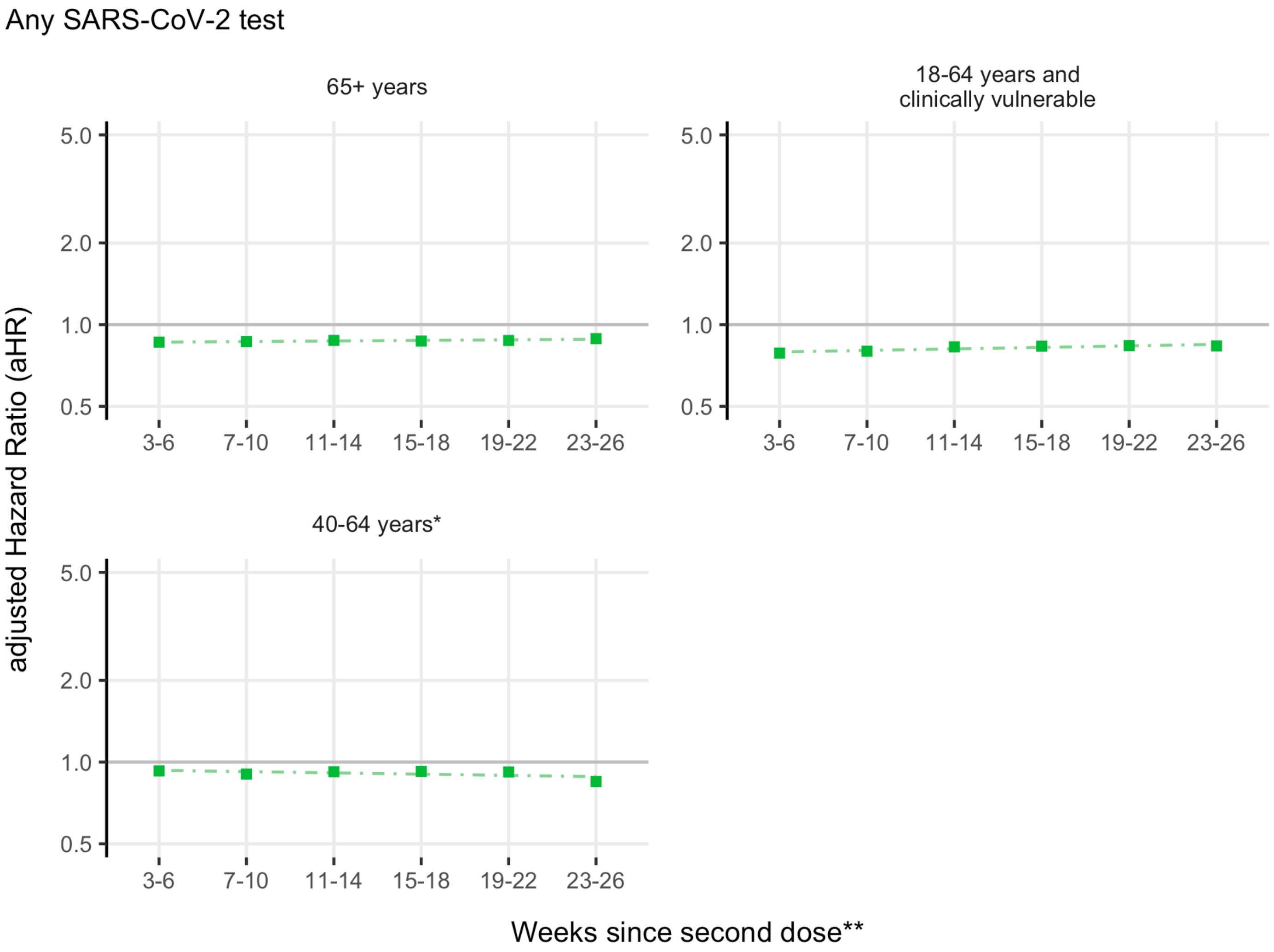

Supplementary Figure 31: Adjusted hazard ratios for any SARS-CoV-2 test for BNT162b2 vs ChAdOx1

Supplementary Table 9: Covariate hazard ratios for COVID-19 hospitalisation in the BNT162b2 vs unvaccinated comparison and the 65+ years subgroup.

		Weeks since second dose					
**Variable**	**Category**	**3-6**	**7-10**	**11-14**	**15-18**	**19-22**	**23-26**
Age*	65-70	1.10 (0.87-1.38)	1.07 (0.92-1.25)	1.02 (0.91-1.14)	1.06 (0.95-1.18)	1.04 (0.93-1.17)	1.07 (0.96-1.20)
	70-75	1.42 (1.02-1.99)	1.05 (0.83-1.31)	0.96 (0.82-1.11)	1.01 (0.90-1.13)	1.05 (0.95-1.17)	1.08 (0.97-1.20)
	75-80	1.15 (0.82-1.60)	1.11 (0.83-1.48)	1.09 (0.90-1.32)	0.99 (0.89-1.10)	1.07 (0.96-1.19)	1.05 (0.95-1.16)
	80+	0.83 (0.14-4.87)	26.51 (0.66-1069.12)	24.33 (0.81-731.05)	1.64 (0.51-5.31)	1.14 (0.47-2.76)	0.93 (0.46-1.91)
	80+ squared	1.00 (0.99-1.01)	0.98 (0.96-1.00)	0.98 (0.96-1.00)	1.00 (0.99-1.00)	1.00 (0.99-1.00)	1.00 (1.00-1.00)
Sex	Female	1.00	1.00	1.00	1.00	1.00	1.00
	Male	1.62 (1.08-2.42)	1.34 (0.96-1.87)	1.12 (0.90-1.41)	1.42 (1.21-1.68)	1.45 (1.25-1.68)	1.54 (1.34-1.76)
IMD	1	1.00	1.00	1.00	1.00	1.00	1.00
	2	0.84 (0.49-1.46)	0.57 (0.35-0.91)	1.03 (0.75-1.42)	0.82 (0.65-1.03)	0.77 (0.62-0.96)	0.88 (0.72-1.06)
	3	0.63 (0.33-1.23)	0.87 (0.54-1.38)	0.81 (0.57-1.17)	0.68 (0.53-0.88)	0.72 (0.57-0.90)	0.69 (0.56-0.84)
	4 / 5	0.55 (0.32-0.94)	-	-	-	-	-
	4	-	0.46 (0.26-0.82)	0.73 (0.50-1.06)	0.69 (0.54-0.89)	0.65 (0.51-0.82)	0.62 (0.50-0.77)
	5	-	0.54 (0.29-0.98)	0.91 (0.63-1.33)	0.62 (0.48-0.82)	0.47 (0.36-0.62)	0.52 (0.42-0.66)
Ethnicity	White	1.00	1.00	1.00	1.00	1.00	1.00
	Black	0.74 (0.24-2.33)	1.61 (0.81-3.21)	-	-	1.08 (0.72-1.65)	0.89 (0.58-1.37)
	South Asian	2.16 (1.18-3.97)	-	-	-	1.22 (0.90-1.66)	0.88 (0.63-1.21)
	Mixed / Other	1.34 (0.48-3.78)	-	-	-	0.83 (0.51-1.37)	-
	South Asian / Mixed / Other	-	1.76 (1.13-2.74)	-	-	-	-
	Black / South Asian / Mixed / Other	-	-	1.10 (0.78-1.53)	1.16 (0.91-1.48)	-	-
	Mixed	-	-	-	-	-	0.38 (0.12-1.20)
	Other	-	-	-	-	-	1.07 (0.62-1.84)
BMI	<30 or missing	1.00	1.00	1.00	1.00	1.00	1.00
	40+ / 30-34.9 / 35-39.9	1.38 (0.88-2.15)	1.40 (0.97-2.02)	-	-	-	-
	40+	-	-	1.96 (1.20-3.21)	1.27 (0.79-2.03)	2.16 (1.54-3.03)	2.90 (2.19-3.85)
	30-34.9	-	-	1.33 (0.99-1.78)	1.32 (1.07-1.63)	1.34 (1.11-1.62)	1.28 (1.08-1.52)
	35-39.9	-	-	1.81 (1.24-2.65)	1.74 (1.30-2.32)	1.25 (0.92-1.70)	1.39 (1.06-1.81)
Serious mental illness		-	-	-	1.03 (0.57-1.89)	1.10 (0.63-1.91)	1.80 (1.18-2.75)
Morbidity count	0	1.00	1.00	1.00	1.00	1.00	1.00
	1	1.39 (0.81-2.36)	1.47 (0.95-2.28)	1.82 (1.34-2.46)	1.63 (1.31-2.02)	1.76 (1.42-2.18)	1.70 (1.41-2.05)
	2+	2.15 (1.27-3.65)	3.15 (2.10-4.74)	3.54 (2.59-4.82)	2.59 (2.08-3.23)	3.46 (2.80-4.26)	2.92 (2.43-3.51)
Flu vaccine		-	0.96 (0.66-1.41)	1.07 (0.82-1.40)	0.93 (0.75-1.14)	0.87 (0.72-1.06)	1.05 (0.88-1.26)
Number of SARS-CoV-2 tests	0	1.00	1.00	1.00	1.00	1.00	1.00
	1	1.84 (1.00-3.37)	1.68 (0.99-2.85)	1.17 (0.80-1.72)	1.42 (1.10-1.84)	1.65 (1.32-2.05)	1.02 (0.81-1.28)
	2 / 3+	2.37 (1.27-4.42)	2.22 (1.25-3.92)	-	-	-	-
	2	-	-	1.33 (0.71-2.50)	1.69 (1.12-2.53)	2.12 (1.52-2.94)	1.64 (1.20-2.24)
	3+	-	-	3.00 (1.93-4.68)	2.75 (1.99-3.80)	2.27 (1.65-3.13)	1.38 (0.97-1.95)

* Estimates correspond to a 1-year increase in age (or age-squared as indicated) within the given age categories.

Supplementary Table 10: Covariate hazard ratios for COVID-19 death in the BNT162b2 vs unvaccinated comparison and the 65+ years subgroup.

		Weeks since second dose					
**Variable**	**Category**	**3-6**	**7-10**	**11-14**	**15-18**	**19-22**	**23-26**
Age*	65-70	1.63 (0.64-4.14)	-	-	1.10 (0.87-1.38)	1.03 (0.80-1.31)	1.03 (0.81-1.32)
	70-75	4.95 (4.22-5.82)	-	-	0.94 (0.73-1.22)	1.09 (0.88-1.33)	1.14 (0.89-1.45)
	75-80	1.06 (0.27-4.22)	-	-	1.09 (0.82-1.44)	1.17 (0.96-1.42)	1.13 (0.87-1.47)
	80+	0.66 (0.08-5.72)	-	-	2.38 (0.20-27.84)	1.16 (0.30-4.48)	0.26 (0.10-0.65)
	80+ squared	1.00 (0.99-1.01)	-	-	1.00 (0.98-1.01)	1.00 (0.99-1.01)	1.01 (1.00-1.01)
Sex	Female	-	-	-	1.00	1.00	1.00
	Male	-	-	-	1.85 (1.24-2.77)	2.07 (1.54-2.78)	2.14 (1.59-2.86)
IMD	1	1.00	-	-	1.00	1.00	1.00
	2 / 3 / 4 / 5	1.02 (0.39-2.68)	-	-	-	-	-
	2	-	-	-	0.74 (0.43-1.27)	0.69 (0.46-1.04)	0.84 (0.55-1.29)
	3	-	-	-	0.51 (0.27-0.97)	0.59 (0.38-0.91)	0.93 (0.61-1.42)
	4	-	-	-	0.63 (0.35-1.16)	0.48 (0.30-0.78)	0.68 (0.43-1.08)
	5	-	-	-	0.75 (0.41-1.37)	0.36 (0.21-0.62)	0.60 (0.37-0.98)
Ethnicity	White	1.00	-	-	1.00	1.00	1.00
	Black	0.00 (0.00-0.00)	-	-	-	-	-
	South Asian / Mixed / Other	3.18 (1.26-8.07)	-	-	-	-	-
	Black / South Asian / Mixed / Other	-	-	-	1.01 (0.55-1.83)	1.31 (0.86-2.00)	1.55 (0.99-2.43)
BMI	<30 or missing	1.00	-	-	1.00	1.00	1.00
	40+ / 30-34.9 / 35-39.9	0.32 (0.08-1.38)	-	-	2.59 (1.68-4.01)	-	-
	40+	-	-	-	-	2.93 (1.55-5.55)	3.76 (2.10-6.73)
	30-34.9	-	-	-	-	1.67 (1.16-2.38)	1.67 (1.18-2.36)
	35-39.9	-	-	-	-	1.86 (1.07-3.24)	0.80 (0.38-1.72)
Morbidity count	0	1.00	-	-	1.00	1.00	1.00
	1	2.73 (0.76-9.76)	-	-	2.01 (1.19-3.38)	1.69 (1.11-2.57)	2.04 (1.33-3.13)
	2+	4.97 (1.49-16.62)	-	-	2.80 (1.62-4.85)	2.92 (1.94-4.38)	3.69 (2.44-5.60)
Flu vaccine		-	-	-	1.08 (0.66-1.75)	1.18 (0.81-1.72)	1.29 (0.88-1.89)
Number of SARS-CoV-2 tests	0	1.00	-	-	1.00	1.00	1.00
	1 / 2 / 3+	2.18 (0.91-5.22)	-	-	-	-	-
	1	-	-	-	1.94 (1.08-3.49)	1.70 (1.12-2.58)	1.23 (0.76-1.97)
	2 / 3+	-	-	-	2.16 (1.11-4.21)	-	-
	2	-	-	-	-	2.14 (1.11-4.10)	1.78 (0.94-3.39)
	3+	-	-	-	-	2.00 (1.05-3.81)	1.41 (0.69-2.88)

* Estimates correspond to a 1-year increase in age (or age-squared as indicated) within the given age categories.

Supplementary Table 11: Covariate hazard ratios for Positive SARS-CoV-2 test in the BNT162b2 vs unvaccinated comparison and the 65+ years subgroup.

		Weeks since second dose					
**Variable**	**Category**	**3-6**	**7-10**	**11-14**	**15-18**	**19-22**	**23-26**
Age*	65-70	0.95 (0.82-1.11)	0.93 (0.86-1.01)	0.97 (0.91-1.03)	0.97 (0.93-1.02)	0.97 (0.92-1.02)	0.97 (0.93-1.01)
	70-75	1.08 (0.89-1.33)	0.94 (0.83-1.06)	0.95 (0.90-1.01)	0.96 (0.92-1.00)	0.97 (0.93-1.01)	0.97 (0.94-1.01)
	75-80	0.92 (0.72-1.18)	1.22 (1.03-1.46)	0.97 (0.88-1.05)	1.01 (0.96-1.07)	0.96 (0.92-1.01)	0.98 (0.94-1.03)
	80+	0.88 (0.15-5.16)	3.22 (0.20-52.39)	3.18 (0.26-38.34)	0.85 (0.33-2.22)	0.66 (0.39-1.11)	0.81 (0.49-1.34)
	80+ squared	1.00 (0.99-1.01)	0.99 (0.98-1.01)	0.99 (0.98-1.01)	1.00 (1.00-1.01)	1.00 (1.00-1.01)	1.00 (1.00-1.00)
Sex	Female	1.00	1.00	1.00	1.00	1.00	1.00
	Male	1.26 (0.97-1.64)	1.23 (1.04-1.46)	1.08 (0.97-1.19)	1.21 (1.12-1.30)	1.11 (1.04-1.19)	1.20 (1.13-1.27)
IMD	1	1.00	1.00	1.00	1.00	1.00	1.00
	2	1.01 (0.69-1.48)	0.95 (0.73-1.23)	0.95 (0.80-1.12)	0.94 (0.84-1.06)	0.94 (0.84-1.05)	1.00 (0.90-1.10)
	3	0.72 (0.46-1.13)	1.00 (0.77-1.31)	0.87 (0.74-1.03)	0.82 (0.73-0.93)	0.89 (0.80-0.99)	0.92 (0.84-1.02)
	4	0.84 (0.54-1.28)	0.95 (0.74-1.24)	0.91 (0.77-1.08)	0.83 (0.74-0.94)	0.85 (0.76-0.94)	0.93 (0.84-1.02)
	5	0.90 (0.60-1.36)	0.90 (0.68-1.20)	0.99 (0.84-1.17)	0.87 (0.77-0.98)	0.76 (0.68-0.86)	0.87 (0.79-0.96)
Ethnicity	White	1.00	1.00	1.00	1.00	1.00	1.00
	Black / South Asian / Mixed / Other	2.34 (1.63-3.36)	1.36 (1.05-1.75)	-	-	-	-
	Black	-	-	0.86 (0.57-1.30)	1.33 (1.01-1.74)	1.22 (0.94-1.59)	0.87 (0.65-1.16)
	South Asian / Mixed / Other	-	-	1.08 (0.88-1.33)	-	-	-
	South Asian	-	-	-	1.25 (1.04-1.50)	1.24 (1.05-1.47)	0.83 (0.69-1.00)
	Mixed	-	-	-	1.13 (0.72-1.78)	0.60 (0.34-1.07)	0.78 (0.49-1.24)
	Other	-	-	-	0.78 (0.53-1.16)	0.72 (0.49-1.05)	0.64 (0.44-0.92)
BMI	<30 or missing	1.00	1.00	1.00	1.00	1.00	1.00
	40+	1.28 (0.59-2.78)	1.01 (0.61-1.67)	1.46 (1.14-1.89)	1.19 (0.96-1.47)	1.54 (1.29-1.83)	1.40 (1.20-1.64)
	30-34.9	1.05 (0.72-1.51)	1.47 (1.19-1.81)	1.22 (1.07-1.40)	1.29 (1.17-1.42)	1.28 (1.17-1.39)	1.18 (1.09-1.27)
	35-39.9	1.17 (0.67-2.03)	1.31 (0.95-1.81)	1.49 (1.24-1.79)	1.18 (1.02-1.37)	1.10 (0.96-1.27)	1.19 (1.06-1.34)
Learning disability		-	-	-	1.57 (0.78-3.15)	-	-
Serious mental illness		-	-	-	0.86 (0.61-1.22)	0.76 (0.54-1.08)	0.81 (0.60-1.09)
Morbidity count	0	1.00	1.00	1.00	1.00	1.00	1.00
	1	1.03 (0.75-1.42)	1.04 (0.85-1.29)	1.33 (1.18-1.51)	1.17 (1.07-1.28)	1.12 (1.04-1.22)	1.05 (0.98-1.13)
	2+	1.12 (0.79-1.59)	1.51 (1.21-1.89)	1.58 (1.37-1.82)	1.35 (1.22-1.49)	1.29 (1.18-1.41)	1.18 (1.09-1.27)
Flu vaccine		1.47 (1.05-2.06)	1.22 (0.97-1.54)	1.26 (1.08-1.46)	1.25 (1.11-1.40)	1.23 (1.11-1.37)	1.37 (1.24-1.51)
Number of SARS-CoV-2 tests	0	1.00	1.00	1.00	1.00	1.00	1.00
	1	2.77 (1.96-3.92)	1.72 (1.33-2.21)	1.62 (1.40-1.88)	1.42 (1.27-1.59)	1.55 (1.41-1.71)	1.17 (1.07-1.29)
	2	2.40 (1.28-4.47)	1.67 (1.06-2.65)	1.35 (1.01-1.81)	1.57 (1.30-1.91)	1.38 (1.15-1.65)	1.21 (1.02-1.42)
	3+	3.23 (1.85-5.64)	1.83 (1.14-2.94)	1.97 (1.50-2.58)	1.58 (1.29-1.95)	1.43 (1.18-1.74)	1.41 (1.19-1.67)

* Estimates correspond to a 1-year increase in age (or age-squared as indicated) within the given age categories.

Supplementary Table 12: Covariate hazard ratios for Non-COVID-19 death in the BNT162b2 vs unvaccinated comparison and the 65+ years subgroup.

		Weeks since second dose					
**Variable**	**Category**	**3-6**	**7-10**	**11-14**	**15-18**	**19-22**	**23-26**
Age*	65-70	0.97 (0.86-1.09)	1.14 (1.00-1.30)	1.10 (0.97-1.26)	1.05 (0.93-1.19)	1.19 (1.05-1.34)	1.11 (0.99-1.23)
	70-75	1.13 (1.01-1.25)	1.08 (0.98-1.17)	1.11 (1.02-1.22)	1.09 (1.00-1.18)	1.02 (0.94-1.10)	1.05 (0.97-1.14)
	75-80	1.06 (0.97-1.16)	1.08 (0.99-1.18)	1.06 (0.98-1.14)	1.09 (1.01-1.18)	1.12 (1.05-1.21)	1.05 (0.98-1.13)
	80+	1.13 (0.72-1.77)	1.13 (0.71-1.80)	0.87 (0.60-1.26)	1.04 (0.68-1.59)	1.17 (0.76-1.82)	1.33 (0.89-2.00)
	80+ squared	1.00 (1.00-1.00)	1.00 (1.00-1.00)	1.00 (1.00-1.00)	1.00 (1.00-1.00)	1.00 (1.00-1.00)	1.00 (1.00-1.00)
Sex	Female	1.00	1.00	1.00	1.00	1.00	1.00
	Male	1.62 (1.44-1.82)	1.47 (1.32-1.64)	1.42 (1.28-1.57)	1.35 (1.23-1.50)	1.27 (1.15-1.40)	1.37 (1.25-1.50)
IMD	1	1.00	1.00	1.00	1.00	1.00	1.00
	2	0.82 (0.68-0.99)	0.89 (0.75-1.06)	0.81 (0.68-0.96)	1.05 (0.89-1.24)	0.72 (0.61-0.84)	0.86 (0.73-1.00)
	3	0.70 (0.58-0.85)	0.71 (0.60-0.85)	0.72 (0.61-0.85)	0.81 (0.69-0.96)	0.69 (0.59-0.80)	0.74 (0.63-0.86)
	4	0.69 (0.57-0.83)	0.75 (0.63-0.89)	0.73 (0.62-0.86)	0.87 (0.74-1.03)	0.61 (0.52-0.72)	0.72 (0.62-0.84)
	5	0.67 (0.56-0.81)	0.67 (0.56-0.80)	0.67 (0.57-0.79)	0.82 (0.69-0.97)	0.58 (0.50-0.68)	0.64 (0.54-0.74)
Ethnicity	White	1.00	1.00	1.00	1.00	1.00	1.00
	Black	0.59 (0.36-0.97)	0.47 (0.26-0.85)	0.55 (0.31-0.96)	0.61 (0.37-1.00)	0.78 (0.49-1.22)	0.70 (0.43-1.13)
	South Asian	0.53 (0.37-0.77)	0.41 (0.27-0.63)	0.45 (0.30-0.68)	0.50 (0.35-0.71)	0.43 (0.29-0.64)	0.41 (0.28-0.62)
	Mixed / Other	0.61 (0.36-1.06)	-	0.62 (0.36-1.06)	0.58 (0.35-0.98)	0.65 (0.39-1.08)	-
	Mixed	-	0.00 (0.00-0.00)	-	-	-	0.67 (0.30-1.51)
	Other	-	0.93 (0.53-1.63)	-	-	-	0.47 (0.22-0.99)
BMI	<30 or missing	1.00	1.00	1.00	1.00	1.00	1.00
	40+	1.03 (0.70-1.51)	1.08 (0.75-1.55)	1.04 (0.73-1.48)	1.16 (0.83-1.61)	0.90 (0.63-1.28)	1.13 (0.83-1.54)
	30-34.9	0.72 (0.61-0.86)	0.72 (0.61-0.85)	0.99 (0.86-1.14)	0.90 (0.78-1.04)	0.84 (0.73-0.97)	0.80 (0.70-0.92)
	35-39.9	0.90 (0.69-1.18)	0.88 (0.68-1.13)	0.80 (0.62-1.04)	0.92 (0.72-1.16)	0.74 (0.58-0.95)	0.78 (0.62-0.99)
Serious mental illness		1.75 (1.17-2.62)	1.77 (1.18-2.65)	1.86 (1.28-2.72)	1.39 (0.91-2.11)	2.03 (1.43-2.87)	1.32 (0.87-2.01)
Morbidity count	0	1.00	1.00	1.00	1.00	1.00	1.00
	1	2.14 (1.80-2.55)	1.69 (1.44-1.99)	1.67 (1.44-1.95)	1.71 (1.48-1.98)	1.70 (1.47-1.97)	1.48 (1.29-1.70)
	2+	3.70 (3.10-4.40)	2.83 (2.42-3.31)	2.81 (2.42-3.27)	2.80 (2.42-3.23)	3.03 (2.62-3.50)	2.64 (2.31-3.02)
Flu vaccine		1.05 (0.87-1.27)	0.93 (0.78-1.11)	0.90 (0.76-1.07)	0.84 (0.72-0.99)	0.89 (0.75-1.05)	0.93 (0.80-1.10)
Number of SARS-CoV-2 tests	0	1.00	1.00	1.00	1.00	1.00	1.00
	1	1.22 (1.02-1.48)	1.61 (1.37-1.89)	1.37 (1.17-1.60)	1.30 (1.11-1.52)	1.27 (1.08-1.49)	1.30 (1.12-1.50)
	2	1.38 (1.03-1.84)	1.92 (1.52-2.43)	2.01 (1.62-2.50)	1.91 (1.54-2.38)	2.21 (1.81-2.71)	1.84 (1.50-2.26)
	3+	3.38 (2.79-4.09)	3.31 (2.75-4.00)	3.27 (2.74-3.91)	3.43 (2.89-4.08)	3.57 (3.02-4.23)	3.34 (2.84-3.93)

* Estimates correspond to a 1-year increase in age (or age-squared as indicated) within the given age categories.

Supplementary Table 13: Covariate hazard ratios for Any SARS-CoV-2 test in the BNT162b2 vs unvaccinated comparison and the 65+ years subgroup.

		Weeks since second dose					
**Variable**	**Category**	**3-6**	**7-10**	**11-14**	**15-18**	**19-22**	**23-26**
Age*	65-70	0.95 (0.94-0.96)	0.95 (0.94-0.96)	0.95 (0.94-0.96)	0.95 (0.94-0.96)	0.95 (0.94-0.96)	0.96 (0.95-0.97)
	70-75	0.96 (0.95-0.97)	0.95 (0.94-0.96)	0.96 (0.95-0.96)	0.95 (0.94-0.96)	0.95 (0.95-0.96)	0.95 (0.95-0.96)
	75-80	0.97 (0.95-0.98)	0.97 (0.96-0.98)	0.96 (0.95-0.97)	0.96 (0.95-0.97)	0.96 (0.95-0.97)	0.96 (0.95-0.97)
	80+	0.68 (0.61-0.77)	0.66 (0.59-0.74)	0.63 (0.56-0.70)	0.60 (0.53-0.66)	0.62 (0.56-0.69)	0.60 (0.54-0.66)
	80+ squared	1.00 (1.00-1.00)	1.00 (1.00-1.00)	1.00 (1.00-1.00)	1.00 (1.00-1.00)	1.00 (1.00-1.00)	1.00 (1.00-1.00)
Sex	Female	1.00	1.00	1.00	1.00	1.00	1.00
	Male	1.02 (1.01-1.04)	1.02 (1.00-1.03)	1.03 (1.02-1.05)	1.03 (1.01-1.04)	1.03 (1.01-1.04)	1.04 (1.02-1.05)
IMD	1	1.00	1.00	1.00	1.00	1.00	1.00
	2	1.08 (1.05-1.11)	1.10 (1.07-1.13)	1.12 (1.09-1.15)	1.12 (1.09-1.15)	1.10 (1.07-1.13)	1.12 (1.09-1.15)
	3	1.13 (1.10-1.17)	1.18 (1.14-1.21)	1.18 (1.15-1.21)	1.18 (1.15-1.21)	1.18 (1.16-1.21)	1.22 (1.19-1.25)
	4	1.17 (1.14-1.20)	1.23 (1.20-1.26)	1.23 (1.20-1.26)	1.25 (1.22-1.28)	1.27 (1.24-1.30)	1.30 (1.27-1.33)
	5	1.24 (1.20-1.27)	1.30 (1.27-1.34)	1.32 (1.29-1.35)	1.33 (1.30-1.36)	1.35 (1.32-1.38)	1.39 (1.35-1.42)
Ethnicity	White	1.00	1.00	1.00	1.00	1.00	1.00
	Black	0.83 (0.76-0.90)	0.84 (0.77-0.91)	0.81 (0.75-0.88)	0.86 (0.79-0.93)	0.83 (0.77-0.90)	0.83 (0.77-0.90)
	South Asian	0.58 (0.54-0.61)	0.61 (0.58-0.64)	0.57 (0.54-0.60)	0.60 (0.57-0.63)	0.62 (0.58-0.65)	0.65 (0.62-0.69)
	Mixed	0.74 (0.65-0.83)	0.75 (0.66-0.84)	0.76 (0.68-0.85)	0.77 (0.68-0.86)	0.84 (0.75-0.94)	0.81 (0.73-0.91)
	Other	0.62 (0.57-0.69)	0.67 (0.61-0.73)	0.62 (0.56-0.68)	0.66 (0.61-0.72)	0.67 (0.62-0.73)	0.67 (0.61-0.73)
BMI	<30 or missing	1.00	1.00	1.00	1.00	1.00	1.00
	40+	0.87 (0.83-0.92)	0.85 (0.81-0.89)	0.86 (0.82-0.90)	0.83 (0.80-0.88)	0.83 (0.79-0.86)	0.82 (0.78-0.86)
	30-34.9	0.96 (0.94-0.98)	0.96 (0.94-0.98)	0.95 (0.93-0.97)	0.95 (0.93-0.96)	0.94 (0.92-0.96)	0.94 (0.93-0.96)
	35-39.9	0.93 (0.90-0.96)	0.95 (0.92-0.98)	0.92 (0.90-0.95)	0.92 (0.89-0.95)	0.88 (0.86-0.91)	0.87 (0.85-0.90)
Learning disability		1.34 (1.12-1.61)	1.36 (1.15-1.62)	1.20 (1.01-1.44)	1.50 (1.27-1.77)	1.36 (1.15-1.60)	1.23 (1.04-1.46)
Serious mental illness		0.98 (0.91-1.05)	1.01 (0.94-1.08)	1.02 (0.95-1.09)	1.01 (0.94-1.08)	1.03 (0.96-1.10)	0.98 (0.92-1.05)
Morbidity count	0	1.00	1.00	1.00	1.00	1.00	1.00
	1	1.02 (1.00-1.04)	1.02 (1.00-1.03)	1.01 (0.99-1.02)	1.03 (1.01-1.04)	1.01 (0.99-1.02)	0.99 (0.97-1.00)
	2+	1.15 (1.13-1.18)	1.13 (1.11-1.16)	1.10 (1.08-1.12)	1.12 (1.10-1.14)	1.09 (1.07-1.11)	1.06 (1.04-1.08)
Flu vaccine		1.55 (1.50-1.59)	1.50 (1.46-1.55)	1.48 (1.45-1.52)	1.45 (1.41-1.49)	1.46 (1.43-1.50)	1.46 (1.42-1.49)
Number of SARS-CoV-2 tests	0	1.00	1.00	1.00	1.00	1.00	1.00
	1	1.67 (1.64-1.71)	1.57 (1.54-1.61)	1.52 (1.49-1.55)	1.48 (1.45-1.51)	1.45 (1.42-1.48)	1.43 (1.40-1.46)
	2	1.96 (1.90-2.03)	1.79 (1.73-1.85)	1.70 (1.65-1.76)	1.73 (1.67-1.79)	1.63 (1.57-1.68)	1.55 (1.50-1.60)
	3+	3.49 (3.39-3.61)	3.12 (3.02-3.21)	2.84 (2.75-2.93)	2.71 (2.63-2.80)	2.54 (2.46-2.62)	2.38 (2.30-2.45)

* Estimates correspond to a 1-year increase in age (or age-squared as indicated) within the given age categories.

Supplementary Table 14: Covariate hazard ratios for COVID-19 hospitalisation in the ChAdOx1 vs unvaccinated comparison and the 65+ years subgroup.

		Weeks since second dose					
**Variable**	**Category**	**3-6**	**7-10**	**11-14**	**15-18**	**19-22**	**23-26**
Age*	65-70	1.04 (0.83-1.30)	1.06 (0.93-1.21)	1.06 (0.96-1.16)	1.03 (0.94-1.13)	1.06 (0.97-1.16)	0.98 (0.90-1.06)
	70-75	1.29 (1.03-1.61)	1.09 (0.92-1.30)	1.07 (0.96-1.19)	1.03 (0.96-1.11)	1.02 (0.96-1.09)	1.04 (0.97-1.11)
	75-80	1.06 (0.78-1.45)	1.09 (0.85-1.39)	1.00 (0.89-1.12)	1.02 (0.94-1.12)	1.08 (1.00-1.16)	1.03 (0.96-1.12)
	80+	0.83 (0.11-6.05)	30.61 (0.59-1585.22)	88.94 (0.31-25706.52)	3.05 (0.44-21.23)	0.70 (0.23-2.11)	1.29 (0.32-5.20)
	80+ squared	1.00 (0.99-1.01)	0.98 (0.96-1.00)	0.97 (0.94-1.01)	0.99 (0.98-1.00)	1.00 (1.00-1.01)	1.00 (0.99-1.01)
Sex	Female	1.00	1.00	1.00	1.00	1.00	1.00
	Male	1.69 (1.16-2.47)	1.49 (1.12-1.98)	1.29 (1.09-1.54)	1.37 (1.20-1.57)	1.30 (1.15-1.47)	1.41 (1.25-1.59)
IMD	1	1.00	1.00	1.00	1.00	1.00	1.00
	2	0.74 (0.44-1.26)	0.57 (0.37-0.87)	0.80 (0.63-1.02)	0.75 (0.62-0.91)	0.80 (0.67-0.95)	0.90 (0.75-1.07)
	3	0.59 (0.32-1.07)	0.80 (0.53-1.20)	0.61 (0.47-0.81)	0.65 (0.53-0.80)	0.72 (0.60-0.86)	0.72 (0.60-0.86)
	4	0.66 (0.38-1.17)	0.69 (0.45-1.07)	0.57 (0.43-0.76)	0.56 (0.45-0.70)	0.63 (0.52-0.77)	0.70 (0.58-0.84)
	5	0.51 (0.27-0.98)	0.52 (0.32-0.86)	0.55 (0.41-0.75)	0.47 (0.37-0.60)	0.48 (0.39-0.60)	0.63 (0.51-0.76)
Ethnicity	White	1.00	1.00	1.00	1.00	1.00	1.00
	Black	0.72 (0.23-2.28)	1.78 (0.92-3.47)	-	1.47 (1.02-2.12)	1.09 (0.73-1.63)	0.97 (0.63-1.50)
	South Asian / Mixed / Other	2.15 (1.25-3.70)	2.07 (1.38-3.11)	-	-	1.03 (0.80-1.32)	-
	Black / South Asian / Mixed / Other	-	-	1.11 (0.85-1.45)	-	-	-
	South Asian	-	-	-	1.25 (0.93-1.68)	-	1.06 (0.80-1.41)
	Mixed / Other	-	-	-	1.24 (0.81-1.90)	-	-
	Mixed	-	-	-	-	-	0.37 (0.12-1.15)
	Other	-	-	-	-	-	1.00 (0.59-1.70)
BMI	<30 or missing	1.00	1.00	1.00	1.00	1.00	1.00
	40+ / 30-34.9 / 35-39.9	1.32 (0.86-2.04)	-	-	-	-	-
	40+	-	2.41 (1.31-4.41)	2.79 (2.08-3.76)	2.20 (1.66-2.91)	2.29 (1.77-2.97)	2.81 (2.24-3.53)
	30-34.9	-	1.87 (1.34-2.61)	1.39 (1.11-1.74)	1.42 (1.20-1.68)	1.80 (1.56-2.09)	1.44 (1.24-1.67)
	35-39.9	-	2.21 (1.41-3.47)	1.95 (1.48-2.58)	1.80 (1.44-2.25)	1.93 (1.58-2.37)	1.91 (1.57-2.31)
Serious mental illness		-	-	1.84 (1.23-2.76)	0.94 (0.55-1.60)	1.43 (0.96-2.14)	1.48 (1.00-2.18)
Morbidity count	0	1.00	1.00	1.00	1.00	1.00	1.00
	1	2.04 (1.25-3.34)	1.52 (1.05-2.20)	1.85 (1.45-2.37)	1.89 (1.58-2.26)	1.93 (1.63-2.30)	1.99 (1.69-2.33)
	2+	3.15 (1.85-5.38)	3.21 (2.25-4.58)	4.06 (3.20-5.15)	3.13 (2.60-3.76)	3.49 (2.94-4.14)	3.44 (2.92-4.05)
Flu vaccine		0.96 (0.59-1.56)	0.97 (0.70-1.35)	1.18 (0.95-1.46)	0.97 (0.82-1.16)	0.99 (0.84-1.16)	1.12 (0.95-1.31)
Number of SARS-CoV-2 tests	0	1.00	1.00	1.00	1.00	1.00	1.00
	1	2.00 (1.16-3.45)	1.31 (0.83-2.07)	1.20 (0.90-1.59)	1.40 (1.14-1.72)	1.40 (1.16-1.68)	1.30 (1.09-1.56)
	2 / 3+	1.76 (0.90-3.44)	-	-	-	-	-
	2	-	1.51 (0.71-3.18)	1.19 (0.73-1.95)	1.59 (1.14-2.21)	2.05 (1.57-2.67)	1.57 (1.19-2.08)
	3+	-	3.06 (1.71-5.46)	2.03 (1.41-2.91)	2.11 (1.56-2.85)	2.14 (1.64-2.81)	1.64 (1.23-2.20)

* Estimates correspond to a 1-year increase in age (or age-squared as indicated) within the given age categories.

Supplementary Table 15: Covariate hazard ratios for COVID-19 death in the ChAdOx1 vs unvaccinated comparison and the 65+ years subgroup.

		Weeks since second dose					
**Variable**	**Category**	**3-6**	**7-10**	**11-14**	**15-18**	**19-22**	**23-26**
Age*	65-70	-	1.00 (0.72-1.39)	-	0.96 (0.79-1.16)	1.07 (0.89-1.30)	1.10 (0.89-1.36)
	70-75	-	1.68 (1.05-2.69)	-	1.01 (0.82-1.24)	1.08 (0.94-1.24)	1.11 (0.93-1.32)
	75-80	-	0.62 (0.59-0.65)	-	1.10 (0.89-1.37)	1.13 (0.96-1.32)	1.15 (0.96-1.37)
	80+	-	26.29 (0.15-4764.08)	-	3.03 (0.18-51.02)	0.40 (0.11-1.47)	0.25 (0.06-1.00)
	80+ squared	-	0.98 (0.95-1.01)	-	0.99 (0.98-1.01)	1.01 (1.00-1.01)	1.01 (1.00-1.02)
Sex	Female	-	-	-	1.00	1.00	1.00
	Male	-	-	-	1.69 (1.19-2.40)	1.43 (1.09-1.86)	1.61 (1.22-2.13)
IMD	1	-	1.00	-	1.00	1.00	1.00
	2 / 3 / 4 / 5	-	0.61 (0.28-1.32)	-	-	-	-
	2	-	-	-	0.70 (0.45-1.08)	0.77 (0.52-1.13)	0.80 (0.54-1.18)
	3	-	-	-	0.42 (0.24-0.71)	0.68 (0.46-1.02)	0.74 (0.49-1.10)
	4	-	-	-	0.45 (0.26-0.77)	0.54 (0.35-0.85)	0.62 (0.40-0.96)
	5	-	-	-	0.49 (0.28-0.85)	0.58 (0.37-0.91)	0.42 (0.26-0.70)
Ethnicity	White	-	1.00	-	1.00	1.00	1.00
	Black / South Asian / Mixed / Other	-	2.24 (1.04-4.84)	-	-	-	-
	Black	-	-	-	1.30 (0.59-2.89)	1.30 (0.64-2.66)	1.16 (0.53-2.54)
	South Asian	-	-	-	0.85 (0.39-1.84)	1.49 (0.91-2.46)	-
	Mixed / Other	-	-	-	0.85 (0.27-2.74)	1.12 (0.50-2.50)	-
	South Asian / Mixed / Other	-	-	-	-	-	1.17 (0.72-1.89)
BMI	<30 or missing	-	1.00	-	1.00	1.00	1.00
	40+ / 30-34.9 / 35-39.9	-	1.76 (0.88-3.51)	-	-	-	-
	40+	-	-	-	5.04 (2.84-8.92)	2.65 (1.54-4.56)	2.33 (1.27-4.29)
	30-34.9	-	-	-	2.62 (1.76-3.89)	1.88 (1.37-2.58)	2.05 (1.49-2.84)
	35-39.9	-	-	-	2.60 (1.47-4.61)	2.26 (1.47-3.48)	2.12 (1.36-3.31)
Serious mental illness		-	-	-	-	1.75 (0.82-3.77)	-
Morbidity count	0	-	1.00	-	1.00	1.00	1.00
	1 / 2+	-	3.16 (1.42-7.03)	-	-	-	-
	1	-	-	-	2.22 (1.37-3.60)	2.43 (1.61-3.65)	1.97 (1.32-2.93)
	2+	-	-	-	3.85 (2.37-6.26)	5.27 (3.57-7.78)	4.13 (2.80-6.10)
Flu vaccine		-	-	-	1.12 (0.73-1.73)	1.09 (0.76-1.56)	1.47 (1.03-2.08)
Number of SARS-CoV-2 tests	0	-	1.00	-	1.00	1.00	1.00
	1 / 2 / 3+	-	2.22 (0.99-4.95)	-	-	-	-
	1	-	-	-	1.88 (1.16-3.02)	1.31 (0.88-1.95)	0.99 (0.61-1.59)
	2 / 3+	-	-	-	1.80 (1.00-3.24)	-	-
	2	-	-	-	-	1.58 (0.83-3.00)	1.56 (0.82-2.97)
	3+	-	-	-	-	1.52 (0.80-2.91)	1.61 (0.85-3.07)

* Estimates correspond to a 1-year increase in age (or age-squared as indicated) within the given age categories.

Supplementary Table 16: Covariate hazard ratios for Positive SARS-CoV-2 test in the ChAdOx1 vs unvaccinated comparison and the 65+ years subgroup.

		Weeks since second dose					
**Variable**	**Category**	**3-6**	**7-10**	**11-14**	**15-18**	**19-22**	**23-26**
Age*	65-70	0.90 (0.80-1.00)	0.94 (0.90-0.99)	0.95 (0.92-0.99)	0.95 (0.92-0.98)	0.94 (0.91-0.97)	0.96 (0.93-0.98)
	70-75	1.07 (0.94-1.23)	0.97 (0.91-1.04)	0.92 (0.89-0.95)	0.94 (0.92-0.97)	0.95 (0.93-0.98)	0.97 (0.95-0.99)
	75-80	1.00 (0.82-1.21)	0.97 (0.84-1.12)	0.98 (0.93-1.04)	0.96 (0.92-1.00)	0.94 (0.91-0.97)	0.97 (0.93-1.00)
	80+	1.82 (0.18-18.15)	9.15 (0.30-280.28)	119.93 (1.24-11573.46)	1.09 (0.30-3.93)	0.60 (0.26-1.37)	0.93 (0.38-2.27)
	80+ squared	1.00 (0.98-1.01)	0.99 (0.97-1.01)	0.97 (0.95-1.00)	1.00 (0.99-1.01)	1.00 (1.00-1.01)	1.00 (1.00-1.01)
Sex	Female	1.00	1.00	1.00	1.00	1.00	1.00
	Male	1.08 (0.88-1.32)	1.13 (1.02-1.26)	1.13 (1.06-1.20)	1.16 (1.10-1.22)	1.06 (1.01-1.11)	1.09 (1.04-1.13)
IMD	1	1.00	1.00	1.00	1.00	1.00	1.00
	2	1.01 (0.74-1.39)	0.86 (0.73-1.02)	0.95 (0.86-1.06)	0.84 (0.77-0.91)	0.93 (0.86-1.01)	1.02 (0.95-1.10)
	3	0.90 (0.65-1.25)	0.81 (0.68-0.96)	0.82 (0.74-0.92)	0.80 (0.74-0.87)	0.88 (0.81-0.95)	1.04 (0.97-1.11)
	4	1.02 (0.74-1.41)	0.85 (0.72-1.01)	0.85 (0.76-0.94)	0.79 (0.72-0.86)	0.89 (0.82-0.96)	0.99 (0.92-1.06)
	5	0.86 (0.61-1.20)	0.95 (0.81-1.12)	0.87 (0.78-0.97)	0.77 (0.71-0.84)	0.80 (0.74-0.87)	1.00 (0.93-1.07)
Ethnicity	White	1.00	1.00	1.00	1.00	1.00	1.00
	Black / South Asian / Mixed / Other	2.02 (1.48-2.77)	-	-	-	-	-
	Black	-	1.33 (0.87-2.03)	1.01 (0.73-1.39)	1.25 (0.99-1.59)	1.08 (0.85-1.39)	0.98 (0.77-1.24)
	South Asian	-	1.71 (1.36-2.16)	1.37 (1.16-1.61)	1.36 (1.19-1.56)	1.19 (1.04-1.36)	1.03 (0.90-1.17)
	Mixed / Other	-	0.71 (0.43-1.16)	-	-	-	-
	Mixed	-	-	0.74 (0.43-1.28)	1.19 (0.83-1.70)	0.62 (0.39-0.98)	0.80 (0.55-1.15)
	Other	-	-	0.89 (0.63-1.27)	0.85 (0.63-1.16)	0.68 (0.50-0.93)	0.71 (0.54-0.93)
BMI	<30 or missing	1.00	1.00	1.00	1.00	1.00	1.00
	40+	1.26 (0.71-2.23)	1.17 (0.88-1.56)	1.50 (1.28-1.75)	1.23 (1.06-1.41)	1.42 (1.25-1.60)	1.21 (1.08-1.36)
	30-34.9	1.39 (1.07-1.80)	1.41 (1.24-1.61)	1.26 (1.16-1.37)	1.28 (1.20-1.37)	1.26 (1.18-1.34)	1.21 (1.15-1.28)
	35-39.9	1.10 (0.72-1.69)	1.20 (0.98-1.48)	1.23 (1.09-1.40)	1.34 (1.21-1.47)	1.30 (1.19-1.42)	1.22 (1.13-1.33)
Learning disability		-	-	-	0.69 (0.33-1.46)	-	-
Serious mental illness		1.47 (0.75-2.86)	0.97 (0.62-1.51)	0.60 (0.41-0.86)	0.61 (0.46-0.82)	0.75 (0.58-0.96)	0.71 (0.56-0.89)
Morbidity count	0	1.00	1.00	1.00	1.00	1.00	1.00
	1	1.22 (0.96-1.55)	1.13 (1.00-1.28)	1.16 (1.08-1.25)	1.05 (0.99-1.12)	1.02 (0.96-1.08)	1.01 (0.96-1.06)
	2+	1.33 (1.00-1.77)	1.31 (1.13-1.51)	1.22 (1.12-1.33)	1.19 (1.11-1.28)	1.13 (1.06-1.21)	1.04 (0.98-1.10)
Flu vaccine		1.62 (1.22-2.16)	1.18 (1.01-1.38)	1.32 (1.19-1.47)	1.38 (1.26-1.50)	1.34 (1.24-1.45)	1.51 (1.41-1.62)
Number of SARS-CoV-2 tests	0	1.00	1.00	1.00	1.00	1.00	1.00
	1	2.06 (1.55-2.73)	1.40 (1.19-1.64)	1.32 (1.20-1.45)	1.37 (1.27-1.48)	1.28 (1.19-1.38)	1.30 (1.22-1.38)
	2	1.94 (1.16-3.22)	1.50 (1.13-2.01)	1.39 (1.16-1.65)	1.25 (1.08-1.45)	1.22 (1.06-1.39)	1.36 (1.21-1.52)
	3+	2.02 (1.19-3.43)	1.75 (1.28-2.40)	1.15 (0.93-1.44)	1.41 (1.21-1.65)	1.22 (1.05-1.42)	1.28 (1.12-1.46)

* Estimates correspond to a 1-year increase in age (or age-squared as indicated) within the given age categories.

Supplementary Table 17: Covariate hazard ratios for Non-COVID-19 death in the ChAdOx1 vs unvaccinated comparison and the 65+ years subgroup.

		Weeks since second dose					
**Variable**	**Category**	**3-6**	**7-10**	**11-14**	**15-18**	**19-22**	**23-26**
Age*	65-70	0.97 (0.87-1.07)	1.12 (1.02-1.23)	1.08 (0.98-1.19)	1.08 (0.98-1.18)	1.06 (0.97-1.17)	1.14 (1.04-1.24)
	70-75	1.17 (1.09-1.26)	1.04 (0.97-1.11)	1.09 (1.02-1.16)	1.10 (1.04-1.17)	1.08 (1.01-1.15)	1.05 (0.99-1.11)
	75-80	1.10 (1.02-1.19)	1.10 (1.03-1.18)	1.07 (1.00-1.14)	1.12 (1.05-1.19)	1.06 (0.99-1.13)	1.02 (0.96-1.09)
	80+	1.49 (0.80-2.76)	2.85 (1.30-6.25)	1.45 (0.66-3.16)	1.07 (0.56-2.07)	1.35 (0.65-2.82)	1.51 (0.74-3.06)
	80+ squared	1.00 (0.99-1.00)	0.99 (0.99-1.00)	1.00 (0.99-1.00)	1.00 (1.00-1.00)	1.00 (0.99-1.00)	1.00 (0.99-1.00)
Sex	Female	1.00	1.00	1.00	1.00	1.00	1.00
	Male	1.44 (1.28-1.62)	1.26 (1.13-1.42)	1.33 (1.19-1.48)	1.40 (1.26-1.55)	1.26 (1.14-1.40)	1.38 (1.25-1.52)
IMD	1	1.00	1.00	1.00	1.00	1.00	1.00
	2	0.93 (0.77-1.11)	0.82 (0.69-0.98)	0.82 (0.69-0.98)	0.84 (0.71-0.98)	0.89 (0.76-1.05)	0.74 (0.64-0.87)
	3	0.81 (0.68-0.98)	0.68 (0.57-0.82)	0.73 (0.61-0.86)	0.76 (0.64-0.89)	0.72 (0.61-0.85)	0.69 (0.60-0.81)
	4	0.73 (0.60-0.88)	0.70 (0.58-0.83)	0.70 (0.59-0.83)	0.72 (0.61-0.84)	0.68 (0.57-0.80)	0.60 (0.51-0.70)
	5	0.71 (0.58-0.86)	0.63 (0.52-0.75)	0.67 (0.56-0.80)	0.65 (0.55-0.77)	0.63 (0.53-0.75)	0.59 (0.50-0.70)
Ethnicity	White	1.00	1.00	1.00	1.00	1.00	1.00
	Black	0.41 (0.23-0.72)	0.45 (0.25-0.82)	0.55 (0.31-0.96)	0.61 (0.37-1.01)	0.58 (0.33-1.01)	0.64 (0.38-1.07)
	South Asian	0.49 (0.33-0.71)	0.33 (0.21-0.52)	0.46 (0.31-0.68)	0.54 (0.38-0.75)	0.54 (0.38-0.78)	0.69 (0.50-0.94)
	Mixed / Other	0.55 (0.31-0.96)	-	0.86 (0.54-1.35)	0.69 (0.43-1.12)	0.73 (0.45-1.19)	-
	Mixed	-	0.34 (0.11-1.07)	-	-	-	0.46 (0.17-1.23)
	Other	-	0.47 (0.23-1.00)	-	-	-	0.27 (0.10-0.74)
BMI	<30 or missing	1.00	1.00	1.00	1.00	1.00	1.00
	40+	1.39 (1.03-1.87)	1.44 (1.10-1.89)	1.12 (0.85-1.49)	1.30 (1.00-1.68)	1.14 (0.87-1.49)	1.22 (0.95-1.56)
	30-34.9	0.82 (0.69-0.97)	0.69 (0.58-0.81)	0.72 (0.62-0.84)	0.85 (0.74-0.98)	0.82 (0.71-0.94)	0.81 (0.70-0.92)
	35-39.9	0.98 (0.77-1.25)	0.95 (0.76-1.19)	0.81 (0.65-1.02)	0.95 (0.78-1.17)	0.66 (0.52-0.85)	0.67 (0.53-0.84)
Serious mental illness		1.69 (1.16-2.47)	2.20 (1.57-3.08)	1.73 (1.21-2.47)	1.70 (1.21-2.40)	1.68 (1.18-2.39)	1.88 (1.36-2.60)
Morbidity count	0	1.00	1.00	1.00	1.00	1.00	1.00
	1	1.97 (1.66-2.33)	2.24 (1.91-2.64)	1.82 (1.56-2.11)	1.94 (1.68-2.24)	1.93 (1.66-2.24)	1.79 (1.56-2.06)
	2+	3.73 (3.14-4.42)	3.98 (3.38-4.69)	3.39 (2.92-3.93)	3.72 (3.22-4.31)	3.78 (3.26-4.39)	3.46 (3.01-3.97)
Flu vaccine		1.18 (0.99-1.40)	0.88 (0.75-1.04)	0.81 (0.69-0.95)	0.87 (0.75-1.01)	0.76 (0.65-0.89)	0.77 (0.66-0.89)
Number of SARS-CoV-2 tests	0	1.00	1.00	1.00	1.00	1.00	1.00
	1	1.24 (1.03-1.50)	1.49 (1.26-1.77)	1.48 (1.26-1.73)	1.50 (1.29-1.75)	1.42 (1.21-1.67)	1.18 (1.00-1.39)
	2	1.79 (1.37-2.34)	1.85 (1.44-2.39)	2.36 (1.90-2.93)	1.95 (1.56-2.44)	2.01 (1.59-2.53)	2.01 (1.62-2.49)
	3+	3.86 (3.21-4.65)	3.80 (3.16-4.57)	3.24 (2.68-3.92)	3.54 (2.97-4.23)	3.55 (2.97-4.24)	3.98 (3.37-4.69)

* Estimates correspond to a 1-year increase in age (or age-squared as indicated) within the given age categories.

Supplementary Table 18: Covariate hazard ratios for Any SARS-CoV-2 test in the ChAdOx1 vs unvaccinated comparison and the 65+ years subgroup.

		Weeks since second dose					
**Variable**	**Category**	**3-6**	**7-10**	**11-14**	**15-18**	**19-22**	**23-26**
Age*	65-70	0.94 (0.93-0.94)	0.93 (0.92-0.94)	0.93 (0.93-0.94)	0.94 (0.93-0.95)	0.94 (0.93-0.94)	0.94 (0.93-0.95)
	70-75	0.95 (0.95-0.96)	0.95 (0.94-0.95)	0.95 (0.94-0.95)	0.94 (0.94-0.95)	0.94 (0.94-0.95)	0.95 (0.94-0.95)
	75-80	0.96 (0.95-0.97)	0.96 (0.95-0.96)	0.95 (0.94-0.96)	0.96 (0.95-0.96)	0.95 (0.94-0.96)	0.94 (0.94-0.95)
	80+	1.09 (0.91-1.30)	1.03 (0.86-1.24)	0.88 (0.74-1.06)	0.91 (0.75-1.10)	0.96 (0.80-1.15)	0.95 (0.79-1.15)
	80+ squared	1.00 (1.00-1.00)	1.00 (1.00-1.00)	1.00 (1.00-1.00)	1.00 (1.00-1.00)	1.00 (1.00-1.00)	1.00 (1.00-1.00)
Sex	Female	1.00	1.00	1.00	1.00	1.00	1.00
	Male	0.99 (0.97-1.00)	0.99 (0.98-1.00)	1.01 (1.00-1.03)	1.01 (1.00-1.02)	1.01 (1.00-1.02)	1.02 (1.01-1.03)
IMD	1	1.00	1.00	1.00	1.00	1.00	1.00
	2	1.11 (1.09-1.14)	1.11 (1.08-1.13)	1.13 (1.10-1.15)	1.11 (1.09-1.14)	1.11 (1.09-1.14)	1.15 (1.12-1.17)
	3	1.16 (1.13-1.19)	1.17 (1.15-1.20)	1.20 (1.17-1.22)	1.17 (1.15-1.20)	1.20 (1.18-1.23)	1.25 (1.23-1.28)
	4	1.26 (1.23-1.29)	1.27 (1.24-1.30)	1.29 (1.26-1.31)	1.27 (1.24-1.29)	1.32 (1.29-1.35)	1.37 (1.34-1.40)
	5	1.32 (1.29-1.35)	1.37 (1.34-1.40)	1.39 (1.37-1.42)	1.37 (1.34-1.40)	1.42 (1.39-1.45)	1.50 (1.47-1.53)
Ethnicity	White	1.00	1.00	1.00	1.00	1.00	1.00
	Black	0.85 (0.79-0.92)	0.83 (0.77-0.90)	0.84 (0.78-0.90)	0.82 (0.76-0.88)	0.81 (0.75-0.87)	0.80 (0.75-0.86)
	South Asian	0.56 (0.54-0.59)	0.57 (0.54-0.59)	0.57 (0.55-0.60)	0.63 (0.60-0.66)	0.60 (0.58-0.63)	0.64 (0.61-0.67)
	Mixed	0.73 (0.65-0.82)	0.76 (0.68-0.84)	0.82 (0.74-0.90)	0.76 (0.69-0.84)	0.84 (0.76-0.92)	0.81 (0.74-0.89)
	Other	0.67 (0.62-0.73)	0.66 (0.61-0.71)	0.67 (0.62-0.72)	0.66 (0.62-0.72)	0.71 (0.66-0.76)	0.67 (0.62-0.72)
BMI	<30 or missing	1.00	1.00	1.00	1.00	1.00	1.00
	40+	0.85 (0.82-0.88)	0.83 (0.80-0.86)	0.85 (0.82-0.88)	0.85 (0.82-0.88)	0.83 (0.80-0.86)	0.82 (0.80-0.85)
	30-34.9	0.95 (0.94-0.97)	0.96 (0.95-0.98)	0.96 (0.94-0.97)	0.95 (0.93-0.96)	0.95 (0.93-0.96)	0.96 (0.94-0.97)
	35-39.9	0.89 (0.87-0.92)	0.90 (0.88-0.92)	0.89 (0.86-0.91)	0.90 (0.88-0.92)	0.90 (0.87-0.92)	0.89 (0.87-0.91)
Learning disability		1.60 (1.41-1.81)	1.62 (1.44-1.82)	1.55 (1.38-1.75)	1.66 (1.48-1.87)	1.54 (1.37-1.73)	1.45 (1.29-1.63)
Serious mental illness		1.00 (0.95-1.06)	0.94 (0.88-0.99)	1.01 (0.96-1.07)	1.03 (0.97-1.08)	0.93 (0.88-0.98)	0.91 (0.86-0.96)
Morbidity count	0	1.00	1.00	1.00	1.00	1.00	1.00
	1	1.00 (0.99-1.02)	0.98 (0.97-1.00)	0.98 (0.97-0.99)	0.98 (0.97-0.99)	0.96 (0.94-0.97)	0.95 (0.94-0.96)
	2+	1.08 (1.06-1.10)	1.06 (1.04-1.08)	1.05 (1.03-1.07)	1.05 (1.04-1.07)	1.00 (0.98-1.01)	0.98 (0.96-0.99)
Flu vaccine		1.58 (1.55-1.62)	1.56 (1.53-1.59)	1.51 (1.48-1.55)	1.51 (1.48-1.54)	1.51 (1.48-1.54)	1.54 (1.51-1.57)
Number of SARS-CoV-2 tests	0	1.00	1.00	1.00	1.00	1.00	1.00
	1	1.57 (1.55-1.60)	1.55 (1.52-1.57)	1.51 (1.49-1.54)	1.48 (1.45-1.50)	1.42 (1.40-1.44)	1.41 (1.39-1.43)
	2	1.83 (1.78-1.89)	1.82 (1.77-1.87)	1.74 (1.69-1.79)	1.65 (1.60-1.70)	1.61 (1.56-1.65)	1.55 (1.50-1.59)
	3+	3.15 (3.07-3.24)	2.86 (2.79-2.94)	2.60 (2.53-2.67)	2.57 (2.51-2.65)	2.35 (2.29-2.42)	2.21 (2.15-2.27)

* Estimates correspond to a 1-year increase in age (or age-squared as indicated) within the given age categories.

Supplementary Table 19: Covariate hazard ratios for COVID-19 hospitalisation in the BNT162b2 vs ChAdOx1 comparison and the 65+ years subgroup.

Variable	Category	Weeks since second dose					
		**3-6**	**7-10**	**11-14**	**15-18**	**19-22**	**23-26**
Age*	65-70	0.69 (0.45-1.05)	1.02 (0.80-1.30)	1.09 (0.96-1.23)	1.07 (0.96-1.19)	1.10 (1.00-1.22)	0.94 (0.86-1.03)
	70-75	1.13 (0.89-1.42)	1.11 (0.92-1.35)	1.08 (0.96-1.20)	1.03 (0.95-1.11)	1.03 (0.97-1.10)	1.03 (0.97-1.10)
	75-80	1.01 (0.71-1.43)	1.07 (0.85-1.35)	1.08 (0.97-1.21)	1.06 (0.97-1.15)	1.06 (0.99-1.14)	1.05 (0.98-1.13)
	80+	1.95 (0.08-49.64)	23.72 (0.01-112523.80)	68.82 (0.65-7330.95)	2.52 (0.60-10.56)	1.15 (0.41-3.24)	0.74 (0.34-1.63)
	80+ squared	1.00 (0.98-1.01)	0.98 (0.93-1.03)	0.98 (0.95-1.00)	1.00 (0.99-1.00)	1.00 (0.99-1.01)	1.00 (1.00-1.01)
Sex	Female	1.00	1.00	1.00	1.00	1.00	1.00
	Male	1.56 (0.95-2.57)	1.94 (1.33-2.83)	1.49 (1.23-1.80)	1.57 (1.36-1.80)	1.43 (1.27-1.62)	1.69 (1.51-1.88)
IMD	1	1.00	1.00	1.00	1.00	1.00	1.00
	2	0.37 (0.16-0.82)	0.85 (0.48-1.48)	0.73 (0.56-0.95)	0.70 (0.57-0.85)	0.77 (0.65-0.92)	0.83 (0.70-0.98)
	3	0.42 (0.21-0.85)	0.89 (0.52-1.53)	0.50 (0.38-0.67)	0.57 (0.46-0.69)	0.64 (0.54-0.77)	0.66 (0.56-0.78)
	4 / 5	0.49 (0.28-0.87)	-	-	-	-	-
	4	-	0.90 (0.51-1.59)	0.50 (0.38-0.67)	0.56 (0.46-0.69)	0.55 (0.46-0.66)	0.68 (0.57-0.80)
	5	-	0.60 (0.32-1.12)	0.46 (0.34-0.61)	0.45 (0.36-0.56)	0.43 (0.35-0.53)	0.56 (0.47-0.67)
Ethnicity	White	1.00	1.00	1.00	1.00	1.00	1.00
	Black	0.00 (0.00-0.00)	0.00 (0.00-0.00)	-	-	0.80 (0.35-1.83)	0.98 (0.48-1.99)
	South Asian / Mixed / Other	4.74 (2.16-10.39)	2.68 (1.40-5.16)	-	-	1.48 (1.13-1.94)	-
	Black / South Asian / Mixed / Other	-	-	0.91 (0.55-1.50)	1.55 (1.16-2.08)	-	-
	South Asian	-	-	-	-	-	1.60 (1.19-2.15)
	Mixed	-	-	-	-	-	0.00 (0.00-0.00)
	Other	-	-	-	-	-	1.65 (0.94-2.91)
BMI	<30 or missing	1.00	1.00	1.00	1.00	1.00	1.00
	40+ / 30-34.9 / 35-39.9	1.43 (0.85-2.42)	1.95 (1.35-2.81)	-	-	-	-
	40+	-	-	2.79 (1.99-3.91)	2.37 (1.80-3.10)	1.89 (1.45-2.45)	3.04 (2.48-3.73)
	30-34.9	-	-	1.35 (1.08-1.70)	1.24 (1.05-1.47)	1.49 (1.30-1.71)	1.27 (1.11-1.45)
	35-39.9	-	-	1.55 (1.12-2.14)	1.79 (1.44-2.23)	1.56 (1.28-1.91)	1.69 (1.40-2.03)
Serious mental illness		-	-	-	0.89 (0.46-1.72)	1.40 (0.88-2.24)	1.24 (0.78-1.98)
Morbidity count	0	1.00	1.00	1.00	1.00	1.00	1.00
	1	1.99 (0.99-4.00)	2.48 (1.42-4.31)	2.11 (1.59-2.80)	1.92 (1.57-2.35)	1.95 (1.62-2.34)	2.15 (1.83-2.53)
	2+	3.78 (1.89-7.59)	5.34 (3.14-9.07)	4.27 (3.24-5.64)	3.82 (3.13-4.67)	4.27 (3.57-5.11)	3.94 (3.35-4.63)
Flu vaccine		-	0.97 (0.51-1.85)	1.24 (0.86-1.79)	0.93 (0.74-1.18)	1.23 (0.97-1.57)	1.42 (1.13-1.79)
Number of SARS-CoV-2 tests	0	1.00	1.00	1.00	1.00	1.00	1.00
	1	1.89 (0.99-3.59)	1.74 (1.08-2.82)	1.37 (1.05-1.80)	1.49 (1.23-1.80)	1.57 (1.34-1.84)	1.28 (1.10-1.50)
	2 / 3+	2.77 (1.40-5.46)	2.38 (1.38-4.12)	-	-	-	-
	2	-	-	1.42 (0.89-2.26)	1.56 (1.14-2.14)	2.05 (1.61-2.61)	1.51 (1.18-1.93)
	3+	-	-	3.03 (2.13-4.31)	3.10 (2.42-3.97)	2.19 (1.70-2.81)	1.79 (1.41-2.29)

* Estimates correspond to a 1-year increase in age (or age-squared as indicated) within the given age categories.

Supplementary Table 20: Covariate hazard ratios for COVID-19 death in the BNT162b2 vs ChAdOx1 comparison and the 65+ years subgroup.

		Weeks since second dose					
**Variable**	**Category**	**3-6**	**7-10**	**11-14**	**15-18**	**19-22**	**23-26**
Age*	65-70	-	-	-	0.96 (0.75-1.23)	1.17 (0.93-1.47)	1.02 (0.76-1.37)
	70-75	-	-	-	1.00 (0.79-1.27)	1.10 (0.94-1.29)	1.05 (0.88-1.24)
	75-80	-	-	-	1.37 (1.10-1.71)	1.15 (0.98-1.35)	1.18 (0.99-1.40)
	80+	-	-	-	0.53 (0.06-4.62)	1.41 (0.21-9.59)	0.33 (0.10-1.13)
	80+ squared	-	-	-	1.00 (0.99-1.02)	1.00 (0.99-1.01)	1.01 (1.00-1.01)
Sex	Female	-	-	-	1.00	1.00	1.00
	Male	-	-	-	1.92 (1.31-2.83)	2.17 (1.63-2.88)	1.84 (1.40-2.41)
IMD	1	-	-	-	1.00	1.00	1.00
	2	-	-	-	0.61 (0.38-0.97)	0.69 (0.46-1.02)	0.87 (0.58-1.31)
	3	-	-	-	0.30 (0.17-0.54)	0.61 (0.41-0.91)	0.79 (0.53-1.18)
	4	-	-	-	0.26 (0.14-0.48)	0.50 (0.33-0.76)	0.65 (0.43-0.99)
	5	-	-	-	0.43 (0.25-0.73)	0.38 (0.24-0.60)	0.50 (0.32-0.78)
Ethnicity	White	-	-	-	1.00	1.00	1.00
	Black / South Asian / Mixed / Other	-	-	-	1.02 (0.42-2.50)	0.88 (0.46-1.68)	1.81 (1.10-3.00)
BMI	<30 or missing	-	-	-	1.00	1.00	1.00
	40+ / 30-34.9 / 35-39.9	-	-	-	2.46 (1.68-3.60)	-	-
	40+	-	-	-	-	1.67 (0.90-3.12)	1.99 (1.06-3.75)
	30-34.9	-	-	-	-	1.33 (0.97-1.82)	1.49 (1.09-2.04)
	35-39.9	-	-	-	-	1.77 (1.15-2.72)	1.76 (1.13-2.74)
Morbidity count	0	-	-	-	1.00	1.00	1.00
	1	-	-	-	2.83 (1.41-5.66)	2.53 (1.56-4.11)	1.80 (1.18-2.75)
	2+	-	-	-	7.13 (3.62-14.07)	6.70 (4.24-10.61)	4.44 (2.96-6.65)
Flu vaccine		-	-	-	1.16 (0.56-2.41)	1.06 (0.63-1.77)	1.74 (0.92-3.29)
Number of SARS-CoV-2 tests	0	-	-	-	1.00	1.00	1.00
	1	-	-	-	1.65 (1.01-2.69)	1.83 (1.30-2.58)	0.80 (0.51-1.25)
	2 / 3+	-	-	-	2.07 (1.19-3.60)	-	-
	2	-	-	-	-	1.67 (0.93-3.03)	0.98 (0.48-1.99)
	3+	-	-	-	-	1.79 (0.99-3.24)	1.53 (0.84-2.78)

* Estimates correspond to a 1-year increase in age (or age-squared as indicated) within the given age categories.

Supplementary Table 21: Covariate hazard ratios for Positive SARS-CoV-2 test in the BNT162b2 vs ChAdOx1 comparison and the 65+ years subgroup.

		Weeks since second dose					
**Variable**	**Category**	**3-6**	**7-10**	**11-14**	**15-18**	**19-22**	**23-26**
Age*	65-70	0.86 (0.75-0.99)	0.94 (0.89-0.98)	0.94 (0.91-0.97)	0.95 (0.92-0.98)	0.94 (0.92-0.97)	0.95 (0.93-0.97)
	70-75	1.01 (0.89-1.13)	0.96 (0.90-1.03)	0.93 (0.90-0.96)	0.95 (0.93-0.97)	0.96 (0.94-0.98)	0.96 (0.95-0.98)
	75-80	1.01 (0.85-1.20)	1.03 (0.92-1.17)	0.98 (0.93-1.03)	0.99 (0.95-1.02)	0.94 (0.91-0.97)	0.97 (0.94-1.00)
	80+	2.24 (0.10-49.35)	4.42 (0.04-556.84)	5.86 (0.25-138.49)	0.54 (0.22-1.32)	0.56 (0.32-0.99)	0.98 (0.56-1.69)
	80+ squared	1.00 (0.98-1.01)	0.99 (0.96-1.02)	0.99 (0.97-1.01)	1.00 (1.00-1.01)	1.00 (1.00-1.01)	1.00 (1.00-1.00)
Sex	Female	1.00	1.00	1.00	1.00	1.00	1.00
	Male	1.00 (0.81-1.23)	1.18 (1.07-1.31)	1.19 (1.12-1.26)	1.20 (1.15-1.26)	1.09 (1.05-1.14)	1.14 (1.10-1.18)
IMD	1	1.00	1.00	1.00	1.00	1.00	1.00
	2	0.92 (0.64-1.33)	0.90 (0.76-1.06)	0.94 (0.85-1.03)	0.84 (0.78-0.91)	0.90 (0.84-0.97)	1.02 (0.95-1.08)
	3	0.86 (0.60-1.22)	0.81 (0.68-0.96)	0.80 (0.72-0.88)	0.80 (0.75-0.87)	0.85 (0.79-0.91)	1.01 (0.95-1.07)
	4	0.97 (0.69-1.36)	0.93 (0.79-1.09)	0.82 (0.74-0.90)	0.79 (0.73-0.85)	0.85 (0.79-0.91)	0.98 (0.93-1.05)
	5	0.99 (0.71-1.39)	0.95 (0.80-1.12)	0.86 (0.78-0.95)	0.78 (0.72-0.84)	0.77 (0.72-0.82)	0.97 (0.91-1.03)
Ethnicity	White	1.00	1.00	1.00	1.00	1.00	1.00
	Black / South Asian / Mixed / Other	2.36 (1.55-3.59)	1.20 (0.93-1.54)	-	-	-	-
	Black	-	-	1.08 (0.72-1.62)	1.00 (0.72-1.39)	0.92 (0.68-1.26)	0.72 (0.53-0.98)
	South Asian / Mixed / Other	-	-	1.14 (0.97-1.34)	-	-	-
	South Asian	-	-	-	1.55 (1.37-1.76)	1.40 (1.25-1.58)	1.10 (0.98-1.24)
	Mixed	-	-	-	1.00 (0.66-1.50)	0.51 (0.30-0.86)	0.82 (0.57-1.16)
	Other	-	-	-	0.92 (0.67-1.25)	0.63 (0.45-0.89)	0.81 (0.63-1.05)
BMI	<30 or missing	1.00	1.00	1.00	1.00	1.00	1.00
	40+	1.22 (0.68-2.21)	1.08 (0.81-1.43)	1.45 (1.25-1.68)	1.23 (1.08-1.39)	1.37 (1.23-1.53)	1.24 (1.13-1.37)
	30-34.9	1.19 (0.90-1.56)	1.40 (1.24-1.58)	1.21 (1.12-1.30)	1.25 (1.18-1.33)	1.22 (1.16-1.28)	1.17 (1.12-1.23)
	35-39.9	1.32 (0.89-1.98)	1.20 (0.98-1.46)	1.20 (1.07-1.34)	1.25 (1.15-1.37)	1.22 (1.13-1.32)	1.19 (1.11-1.27)
Learning disability		-	-	-	0.74 (0.38-1.42)	1.01 (0.60-1.71)	0.88 (0.55-1.41)
Serious mental illness		-	-	-	0.48 (0.35-0.66)	0.68 (0.53-0.87)	0.64 (0.52-0.80)
Morbidity count	0	1.00	1.00	1.00	1.00	1.00	1.00
	1	1.14 (0.89-1.46)	1.08 (0.96-1.22)	1.13 (1.05-1.21)	1.05 (0.99-1.10)	1.02 (0.97-1.07)	1.00 (0.96-1.04)
	2+	1.13 (0.84-1.51)	1.27 (1.11-1.47)	1.22 (1.13-1.33)	1.22 (1.15-1.29)	1.14 (1.08-1.20)	1.06 (1.01-1.11)
Flu vaccine		1.83 (1.18-2.84)	1.14 (0.97-1.34)	1.34 (1.20-1.49)	1.36 (1.25-1.48)	1.39 (1.29-1.50)	1.54 (1.44-1.64)
Number of SARS-CoV-2 tests	0	1.00	1.00	1.00	1.00	1.00	1.00
	1	2.01 (1.53-2.66)	1.45 (1.25-1.68)	1.41 (1.30-1.54)	1.34 (1.25-1.43)	1.33 (1.25-1.41)	1.26 (1.19-1.33)
	2	1.56 (0.89-2.73)	1.49 (1.13-1.96)	1.39 (1.18-1.63)	1.24 (1.10-1.41)	1.23 (1.10-1.38)	1.27 (1.15-1.40)
	3+	2.31 (1.37-3.90)	1.50 (1.08-2.09)	1.30 (1.07-1.57)	1.47 (1.29-1.68)	1.22 (1.07-1.39)	1.30 (1.16-1.46)

* Estimates correspond to a 1-year increase in age (or age-squared as indicated) within the given age categories.

Supplementary Table 22: Covariate hazard ratios for Non-COVID-19 death in the BNT162b2 vs ChAdOx1 comparison and the 65+ years subgroup.

		Weeks since second dose					
**Variable**	**Category**	**3-6**	**7-10**	**11-14**	**15-18**	**19-22**	**23-26**
Age*	65-70	1.05 (0.96-1.16)	1.18 (1.08-1.30)	1.11 (1.02-1.22)	1.07 (0.98-1.16)	1.03 (0.94-1.11)	1.13 (1.05-1.23)
	70-75	1.15 (1.08-1.23)	1.08 (1.01-1.14)	1.11 (1.05-1.17)	1.12 (1.06-1.18)	1.07 (1.01-1.13)	1.05 (0.99-1.10)
	75-80	1.10 (1.03-1.17)	1.11 (1.05-1.18)	1.09 (1.04-1.15)	1.11 (1.06-1.17)	1.08 (1.03-1.14)	1.04 (0.99-1.09)
	80+	0.89 (0.56-1.41)	0.98 (0.61-1.56)	0.83 (0.56-1.24)	0.86 (0.57-1.29)	1.11 (0.70-1.74)	1.17 (0.78-1.75)
	80+ squared	1.00 (1.00-1.00)	1.00 (1.00-1.00)	1.00 (1.00-1.00)	1.00 (1.00-1.00)	1.00 (1.00-1.00)	1.00 (1.00-1.00)
Sex	Female	1.00	1.00	1.00	1.00	1.00	1.00
	Male	1.55 (1.41-1.71)	1.44 (1.31-1.57)	1.44 (1.33-1.57)	1.36 (1.26-1.48)	1.33 (1.22-1.43)	1.37 (1.27-1.47)
IMD	1	1.00	1.00	1.00	1.00	1.00	1.00
	2	0.91 (0.77-1.08)	0.80 (0.69-0.93)	0.82 (0.71-0.94)	0.89 (0.78-1.02)	0.85 (0.74-0.97)	0.80 (0.71-0.90)
	3	0.78 (0.66-0.92)	0.68 (0.59-0.78)	0.72 (0.63-0.82)	0.80 (0.70-0.91)	0.75 (0.66-0.85)	0.74 (0.66-0.83)
	4	0.77 (0.65-0.90)	0.68 (0.59-0.78)	0.74 (0.65-0.84)	0.79 (0.69-0.89)	0.72 (0.63-0.81)	0.66 (0.59-0.75)
	5	0.73 (0.62-0.86)	0.60 (0.52-0.69)	0.67 (0.59-0.77)	0.71 (0.63-0.81)	0.64 (0.56-0.72)	0.61 (0.54-0.69)
Ethnicity	White	1.00	1.00	1.00	1.00	1.00	1.00
	Black	0.57 (0.23-1.39)	0.52 (0.23-1.18)	0.61 (0.30-1.24)	1.15 (0.71-1.88)	1.04 (0.63-1.73)	1.15 (0.72-1.82)
	South Asian	0.66 (0.44-0.98)	0.53 (0.36-0.79)	0.62 (0.44-0.88)	0.58 (0.42-0.81)	0.71 (0.52-0.96)	0.73 (0.54-0.97)
	Mixed / Other	0.81 (0.44-1.48)	-	1.20 (0.80-1.80)	-	-	-
	Mixed	-	0.50 (0.16-1.56)	-	1.18 (0.61-2.28)	0.80 (0.36-1.79)	1.21 (0.65-2.26)
	Other	-	1.06 (0.60-1.89)	-	0.83 (0.47-1.48)	1.06 (0.64-1.77)	0.72 (0.40-1.31)
BMI	<30 or missing	1.00	1.00	1.00	1.00	1.00	1.00
	40+	1.28 (0.97-1.68)	1.35 (1.06-1.70)	1.16 (0.92-1.47)	1.33 (1.07-1.65)	1.07 (0.85-1.35)	1.28 (1.05-1.56)
	30-34.9	0.75 (0.65-0.87)	0.68 (0.60-0.78)	0.86 (0.77-0.96)	0.87 (0.78-0.97)	0.83 (0.75-0.93)	0.81 (0.73-0.90)
	35-39.9	1.05 (0.86-1.28)	0.88 (0.73-1.07)	0.88 (0.73-1.05)	0.95 (0.80-1.12)	0.69 (0.57-0.84)	0.78 (0.66-0.92)
Serious mental illness		1.73 (1.18-2.54)	1.69 (1.19-2.39)	1.90 (1.40-2.57)	1.24 (0.86-1.77)	1.62 (1.18-2.23)	1.69 (1.26-2.27)
Morbidity count	0	1.00	1.00	1.00	1.00	1.00	1.00
	1	1.88 (1.63-2.18)	1.83 (1.61-2.08)	1.77 (1.57-1.99)	1.74 (1.55-1.95)	1.77 (1.58-1.99)	1.70 (1.52-1.89)
	2+	3.44 (2.98-3.97)	3.19 (2.80-3.64)	3.18 (2.82-3.58)	3.16 (2.82-3.55)	3.34 (2.98-3.74)	3.25 (2.92-3.62)
Flu vaccine		0.76 (0.64-0.89)	0.78 (0.67-0.91)	0.72 (0.63-0.82)	0.80 (0.70-0.92)	0.73 (0.64-0.84)	0.78 (0.69-0.88)
Number of SARS-CoV-2 tests	0	1.00	1.00	1.00	1.00	1.00	1.00
	1	1.28 (1.10-1.49)	1.46 (1.28-1.66)	1.43 (1.27-1.61)	1.33 (1.18-1.50)	1.34 (1.19-1.51)	1.21 (1.07-1.35)
	2	1.71 (1.37-2.14)	1.96 (1.62-2.37)	2.23 (1.90-2.63)	1.96 (1.66-2.32)	2.02 (1.71-2.39)	1.87 (1.60-2.20)
	3+	3.98 (3.40-4.65)	3.83 (3.31-4.42)	3.31 (2.87-3.82)	3.53 (3.08-4.04)	3.69 (3.23-4.21)	3.62 (3.19-4.09)

* Estimates correspond to a 1-year increase in age (or age-squared as indicated) within the given age categories.

Supplementary Table 23: Covariate hazard ratios for Any SARS-CoV-2 test in the BNT162b2 vs ChAdOx1 comparison and the 65+ years subgroup.

		Weeks since second dose					
**Variable**	**Category**	**3-6**	**7-10**	**11-14**	**15-18**	**19-22**	**23-26**
Age*	65-70	0.94 (0.93-0.94)	0.93 (0.93-0.94)	0.93 (0.93-0.94)	0.94 (0.93-0.94)	0.94 (0.93-0.94)	0.94 (0.94-0.95)
	70-75	0.95 (0.95-0.96)	0.95 (0.94-0.95)	0.95 (0.94-0.95)	0.94 (0.94-0.95)	0.94 (0.94-0.95)	0.95 (0.94-0.95)
	75-80	0.96 (0.95-0.97)	0.96 (0.95-0.96)	0.95 (0.95-0.96)	0.96 (0.95-0.96)	0.95 (0.95-0.96)	0.95 (0.94-0.96)
	80+	0.64 (0.57-0.71)	0.62 (0.56-0.69)	0.59 (0.53-0.65)	0.56 (0.51-0.62)	0.59 (0.53-0.65)	0.57 (0.51-0.62)
	80+ squared	1.00 (1.00-1.00)	1.00 (1.00-1.00)	1.00 (1.00-1.00)	1.00 (1.00-1.00)	1.00 (1.00-1.00)	1.00 (1.00-1.00)
Sex	Female	1.00	1.00	1.00	1.00	1.00	1.00
	Male	1.01 (1.00-1.02)	1.01 (1.00-1.02)	1.03 (1.02-1.04)	1.02 (1.01-1.03)	1.02 (1.01-1.03)	1.03 (1.02-1.04)
IMD	1	1.00	1.00	1.00	1.00	1.00	1.00
	2	1.12 (1.10-1.14)	1.12 (1.10-1.14)	1.14 (1.12-1.16)	1.11 (1.09-1.13)	1.12 (1.10-1.14)	1.14 (1.12-1.16)
	3	1.17 (1.14-1.19)	1.19 (1.16-1.21)	1.20 (1.18-1.22)	1.18 (1.16-1.20)	1.20 (1.18-1.22)	1.25 (1.23-1.27)
	4	1.24 (1.22-1.26)	1.27 (1.25-1.29)	1.28 (1.26-1.30)	1.26 (1.24-1.28)	1.31 (1.29-1.33)	1.36 (1.33-1.38)
	5	1.30 (1.28-1.33)	1.36 (1.33-1.38)	1.38 (1.35-1.40)	1.36 (1.33-1.38)	1.40 (1.38-1.43)	1.47 (1.44-1.49)
Ethnicity	White	1.00	1.00	1.00	1.00	1.00	1.00
	Black	0.87 (0.80-0.93)	0.85 (0.79-0.92)	0.84 (0.78-0.90)	0.84 (0.78-0.90)	0.82 (0.76-0.88)	0.85 (0.79-0.91)
	South Asian	0.58 (0.56-0.61)	0.59 (0.57-0.62)	0.59 (0.56-0.61)	0.63 (0.61-0.66)	0.61 (0.59-0.64)	0.63 (0.61-0.65)
	Mixed	0.74 (0.67-0.81)	0.82 (0.75-0.90)	0.81 (0.74-0.88)	0.77 (0.71-0.84)	0.80 (0.73-0.87)	0.85 (0.78-0.91)
	Other	0.69 (0.64-0.74)	0.68 (0.63-0.73)	0.68 (0.63-0.72)	0.72 (0.68-0.77)	0.74 (0.70-0.78)	0.72 (0.68-0.76)
BMI	<30 or missing	1.00	1.00	1.00	1.00	1.00	1.00
	40+	0.84 (0.81-0.87)	0.84 (0.81-0.87)	0.85 (0.82-0.87)	0.84 (0.81-0.86)	0.82 (0.80-0.84)	0.82 (0.79-0.84)
	30-34.9	0.96 (0.94-0.97)	0.96 (0.94-0.97)	0.95 (0.94-0.96)	0.94 (0.93-0.95)	0.94 (0.93-0.95)	0.94 (0.93-0.96)
	35-39.9	0.90 (0.89-0.92)	0.91 (0.89-0.93)	0.90 (0.88-0.91)	0.90 (0.88-0.92)	0.88 (0.86-0.90)	0.87 (0.86-0.89)
Learning disability		1.55 (1.39-1.73)	1.51 (1.36-1.68)	1.44 (1.29-1.59)	1.60 (1.44-1.77)	1.52 (1.38-1.68)	1.38 (1.25-1.52)
Serious mental illness		0.92 (0.87-0.97)	0.92 (0.88-0.97)	0.97 (0.93-1.02)	0.99 (0.94-1.04)	0.92 (0.88-0.96)	0.90 (0.86-0.94)
Morbidity count	0	1.00	1.00	1.00	1.00	1.00	1.00
	1	1.00 (0.98-1.01)	0.99 (0.98-1.00)	0.98 (0.97-0.99)	0.99 (0.97-1.00)	0.97 (0.96-0.98)	0.95 (0.94-0.96)
	2+	1.09 (1.08-1.11)	1.07 (1.06-1.09)	1.05 (1.04-1.07)	1.07 (1.05-1.08)	1.02 (1.01-1.03)	1.00 (0.99-1.01)
Flu vaccine		1.52 (1.49-1.55)	1.52 (1.49-1.55)	1.50 (1.47-1.52)	1.49 (1.47-1.52)	1.49 (1.47-1.52)	1.52 (1.50-1.55)
Number of SARS-CoV-2 tests	0	1.00	1.00	1.00	1.00	1.00	1.00
	1	1.57 (1.55-1.59)	1.52 (1.50-1.54)	1.49 (1.47-1.51)	1.45 (1.43-1.47)	1.41 (1.39-1.43)	1.40 (1.38-1.41)
	2	1.83 (1.79-1.88)	1.76 (1.72-1.80)	1.69 (1.65-1.72)	1.64 (1.60-1.68)	1.58 (1.54-1.61)	1.52 (1.49-1.55)
	3+	3.14 (3.07-3.21)	2.84 (2.78-2.91)	2.59 (2.53-2.64)	2.56 (2.51-2.62)	2.39 (2.34-2.44)	2.23 (2.19-2.28)

* Estimates correspond to a 1-year increase in age (or age-squared as indicated) within the given age categories.

Supplementary Table 24: Covariate hazard ratios for COVID-19 hospitalisation in the BNT162b2 vs unvaccinated comparison and the 18-64 years and clinically vulnerable subgroup.

		Weeks since second dose					
**Variable**	**Category**	**3-6**	**7-10**	**11-14**	**15-18**	**19-22**	**23-26**
Age*	18-64	1.14 (1.03-1.25)	1.01 (0.96-1.07)	1.02 (0.97-1.06)	1.00 (0.96-1.05)	1.04 (0.98-1.10)	1.04 (0.98-1.10)
	18-64 squared	1.00 (1.00-1.00)	1.00 (1.00-1.00)	1.00 (1.00-1.00)	1.00 (1.00-1.00)	1.00 (1.00-1.00)	1.00 (1.00-1.00)
Sex	Female	-	1.00	1.00	1.00	1.00	1.00
	Male	-	0.89 (0.74-1.08)	0.96 (0.82-1.13)	0.94 (0.80-1.11)	1.08 (0.90-1.28)	1.19 (1.00-1.41)
IMD	1	1.00	1.00	1.00	1.00	1.00	1.00
	2 / 3 / 4 / 5	0.77 (0.55-1.08)	-	-	-	-	-
	2	-	0.95 (0.75-1.21)	1.16 (0.95-1.42)	0.92 (0.74-1.14)	1.08 (0.85-1.36)	1.14 (0.91-1.43)
	3	-	1.25 (0.97-1.62)	1.01 (0.79-1.30)	0.78 (0.60-1.00)	1.10 (0.84-1.44)	1.30 (1.03-1.66)
	4 / 5	-	0.81 (0.62-1.07)	-	-	-	-
	4	-	-	0.95 (0.72-1.26)	0.95 (0.73-1.24)	1.10 (0.82-1.48)	0.98 (0.74-1.30)
	5	-	-	1.25 (0.92-1.68)	0.72 (0.51-1.02)	1.16 (0.83-1.61)	1.22 (0.90-1.67)
Ethnicity	White	1.00	1.00	1.00	1.00	1.00	1.00
	Black / South Asian / Mixed / Other	1.75 (1.24-2.46)	-	1.61 (1.35-1.91)	1.22 (1.01-1.49)	-	0.93 (0.74-1.15)
	Black	-	1.34 (0.97-1.84)	-	-	1.42 (1.05-1.92)	-
	South Asian / Mixed / Other	-	1.27 (1.00-1.61)	-	-	1.34 (1.06-1.68)	-
BMI	<30 or missing	1.00	1.00	1.00	1.00	1.00	1.00
	40+ / 30-34.9 / 35-39.9	1.78 (1.31-2.42)	-	-	-	-	-
	40+	-	2.49 (1.90-3.25)	2.27 (1.79-2.88)	2.45 (1.96-3.08)	3.03 (2.39-3.85)	3.42 (2.73-4.28)
	30-34.9	-	1.39 (1.06-1.82)	1.78 (1.44-2.21)	1.47 (1.18-1.84)	1.67 (1.31-2.12)	1.41 (1.11-1.80)
	35-39.9	-	2.41 (1.81-3.22)	2.45 (1.92-3.12)	1.41 (1.05-1.90)	2.53 (1.94-3.31)	1.99 (1.50-2.62)
Learning disability		-	-	-	-	-	1.13 (0.65-1.95)
Serious mental illness		-	0.60 (0.39-0.92)	0.77 (0.55-1.07)	-	0.88 (0.61-1.27)	0.69 (0.48-1.01)
Morbidity count	0	1.00	1.00	1.00	1.00	1.00	1.00
	1 / 2+	0.95 (0.67-1.35)	-	1.24 (0.99-1.56)	-	-	-
	1	-	1.07 (0.81-1.40)	-	1.44 (1.15-1.80)	1.26 (0.98-1.62)	1.16 (0.90-1.48)
	2+	-	1.36 (0.97-1.90)	-	2.15 (1.66-2.79)	1.86 (1.39-2.49)	1.86 (1.40-2.47)
Flu vaccine		-	1.08 (0.88-1.32)	1.20 (1.02-1.42)	1.17 (0.98-1.41)	1.25 (1.03-1.50)	1.10 (0.91-1.32)
Number of SARS-CoV-2 tests	0	1.00	1.00	1.00	1.00	1.00	1.00
	1 / 2 / 3+	1.34 (0.96-1.86)	-	-	-	-	-
	1	-	1.73 (1.38-2.18)	1.21 (0.97-1.51)	1.22 (0.97-1.54)	1.58 (1.26-1.99)	1.65 (1.33-2.05)
	2	-	1.57 (1.09-2.27)	1.61 (1.18-2.19)	1.83 (1.36-2.45)	1.97 (1.43-2.71)	1.92 (1.41-2.64)
	3+	-	1.62 (1.14-2.31)	2.21 (1.71-2.87)	1.81 (1.36-2.41)	2.14 (1.58-2.90)	2.64 (2.00-3.50)

* Estimates correspond to a 1-year increase in age (or age-squared as indicated) within the given age categories.

Supplementary Table 25: Covariate hazard ratios for COVID-19 death in the BNT162b2 vs unvaccinated comparison and the 18-64 years and clinically vulnerable subgroup.

		Weeks since second dose					
**Variable**	**Category**	**3-6**	**7-10**	**11-14**	**15-18**	**19-22**	**23-26**
Age*	18-64	-	-	-	1.13 (0.95-1.33)	1.10 (0.89-1.35)	1.14 (0.91-1.42)
	18-64 squared	-	-	-	1.00 (1.00-1.00)	1.00 (1.00-1.00)	1.00 (1.00-1.00)
Sex	Female	-	-	-	-	1.00	1.00
	Male	-	-	-	-	1.83 (1.16-2.89)	1.76 (0.99-3.14)
IMD	1	-	-	-	1.00	1.00	1.00
	2 / 3 / 4 / 5	-	-	-	0.68 (0.43-1.09)	1.36 (0.83-2.24)	-
	2	-	-	-	-	-	1.56 (0.81-3.03)
	3 / 4 / 5	-	-	-	-	-	0.94 (0.48-1.86)
Ethnicity	White	-	-	-	1.00	1.00	1.00
	Black	-	-	-	1.26 (0.55-2.84)	1.59 (0.70-3.58)	-
	South Asian	-	-	-	0.88 (0.39-2.01)	-	-
	Mixed	-	-	-	0.00 (0.00-0.00)	-	-
	Other	-	-	-	1.90 (0.66-5.46)	-	-
	South Asian / Mixed / Other	-	-	-	-	1.46 (0.79-2.68)	-
	Black / South Asian / Mixed / Other	-	-	-	-	-	0.55 (0.27-1.14)
BMI	<30 or missing	-	-	-	1.00	1.00	1.00
	40+ / 30-34.9 / 35-39.9	-	-	-	2.11 (1.35-3.29)	2.84 (1.79-4.50)	-
	40+	-	-	-	-	-	5.39 (2.48-11.70)
	30-34.9 / 35-39.9	-	-	-	-	-	2.51 (1.33-4.73)
Morbidity count	0	-	-	-	1.00	1.00	1.00
	1 / 2+	-	-	-	1.00 (0.57-1.76)	-	-
	1	-	-	-	-	0.65 (0.35-1.22)	1.21 (0.52-2.80)
	2+	-	-	-	-	2.20 (1.15-4.21)	2.95 (1.25-6.99)
Number of SARS-CoV-2 tests	0	-	-	-	1.00	1.00	1.00
	1 / 2 / 3+	-	-	-	1.13 (0.67-1.90)	-	1.54 (0.88-2.69)
	1	-	-	-	-	1.23 (0.66-2.30)	-
	2 / 3+	-	-	-	-	1.31 (0.68-2.53)	-

* Estimates correspond to a 1-year increase in age (or age-squared as indicated) within the given age categories.

Supplementary Table 26: Covariate hazard ratios for Positive SARS-CoV-2 test in the BNT162b2 vs unvaccinated comparison and the 18-64 years and clinically vulnerable subgroup.

		Weeks since second dose					
**Variable**	**Category**	**3-6**	**7-10**	**11-14**	**15-18**	**19-22**	**23-26**
Age*	18-64	0.96 (0.93-0.98)	0.97 (0.95-0.98)	0.98 (0.97-1.00)	1.02 (1.01-1.04)	1.09 (1.07-1.11)	1.09 (1.08-1.11)
	18-64 squared	1.00 (1.00-1.00)	1.00 (1.00-1.00)	1.00 (1.00-1.00)	1.00 (1.00-1.00)	1.00 (1.00-1.00)	1.00 (1.00-1.00)
Sex	Female	1.00	1.00	1.00	1.00	1.00	1.00
	Male	0.89 (0.80-0.98)	0.94 (0.88-1.00)	0.89 (0.84-0.94)	0.87 (0.82-0.91)	0.81 (0.77-0.85)	0.83 (0.80-0.87)
IMD	1	1.00	1.00	1.00	1.00	1.00	1.00
	2	1.00 (0.88-1.15)	1.04 (0.96-1.12)	1.06 (0.99-1.14)	1.08 (1.00-1.16)	1.12 (1.04-1.20)	1.05 (0.98-1.12)
	3	1.11 (0.96-1.29)	1.13 (1.03-1.23)	0.97 (0.90-1.05)	1.04 (0.96-1.13)	1.14 (1.06-1.24)	1.15 (1.07-1.23)
	4	1.18 (1.01-1.38)	1.09 (0.99-1.20)	1.09 (1.00-1.18)	1.05 (0.97-1.14)	1.16 (1.07-1.26)	1.19 (1.11-1.28)
	5	1.15 (0.96-1.38)	1.16 (1.05-1.29)	1.02 (0.93-1.12)	0.99 (0.90-1.09)	1.21 (1.11-1.32)	1.24 (1.15-1.33)
Ethnicity	White	1.00	1.00	1.00	1.00	1.00	1.00
	Black / South Asian / Mixed / Other	1.24 (1.11-1.39)	-	-	-	-	-
	Black	-	1.06 (0.94-1.21)	1.08 (0.97-1.21)	0.96 (0.85-1.08)	0.90 (0.79-1.03)	0.71 (0.62-0.81)
	South Asian	-	0.74 (0.66-0.82)	0.88 (0.80-0.96)	0.85 (0.77-0.94)	0.84 (0.76-0.93)	0.91 (0.83-1.00)
	Mixed	-	0.95 (0.79-1.15)	1.00 (0.85-1.18)	1.17 (1.00-1.38)	1.19 (1.01-1.41)	0.81 (0.67-0.97)
	Other	-	0.55 (0.43-0.70)	0.97 (0.82-1.14)	0.72 (0.59-0.88)	0.78 (0.64-0.96)	0.72 (0.60-0.87)
BMI	<30 or missing	1.00	1.00	1.00	1.00	1.00	1.00
	40+	1.00 (0.85-1.17)	1.07 (0.97-1.18)	1.13 (1.04-1.23)	1.26 (1.16-1.36)	1.26 (1.16-1.36)	1.19 (1.11-1.28)
	30-34.9	1.14 (0.98-1.32)	1.27 (1.17-1.38)	1.19 (1.11-1.28)	1.16 (1.08-1.25)	1.15 (1.07-1.24)	1.13 (1.06-1.20)
	35-39.9	1.33 (1.11-1.59)	1.24 (1.11-1.38)	1.18 (1.08-1.29)	1.34 (1.23-1.46)	1.26 (1.15-1.38)	1.17 (1.08-1.27)
Learning disability		-	0.43 (0.35-0.53)	0.54 (0.45-0.64)	0.55 (0.46-0.66)	0.55 (0.46-0.66)	0.59 (0.50-0.69)
Serious mental illness		0.49 (0.39-0.62)	0.52 (0.45-0.60)	0.60 (0.53-0.68)	0.54 (0.47-0.62)	0.50 (0.44-0.58)	0.55 (0.49-0.61)
Morbidity count	0	1.00	1.00	1.00	1.00	1.00	1.00
	1	0.99 (0.86-1.14)	0.94 (0.85-1.03)	1.00 (0.93-1.08)	1.04 (0.96-1.12)	0.98 (0.91-1.06)	0.95 (0.89-1.02)
	2+	0.93 (0.75-1.15)	0.86 (0.76-0.97)	0.95 (0.86-1.05)	1.05 (0.95-1.16)	0.92 (0.83-1.02)	0.92 (0.85-1.00)
Flu vaccine		0.96 (0.86-1.06)	0.96 (0.90-1.03)	1.00 (0.95-1.06)	1.08 (1.02-1.15)	1.10 (1.03-1.16)	1.09 (1.04-1.15)
Number of SARS-CoV-2 tests	0	1.00	1.00	1.00	1.00	1.00	1.00
	1	1.64 (1.46-1.86)	1.73 (1.61-1.86)	1.58 (1.48-1.68)	1.50 (1.41-1.60)	1.35 (1.27-1.44)	1.39 (1.31-1.47)
	2	1.92 (1.61-2.30)	1.73 (1.55-1.93)	1.77 (1.61-1.94)	1.65 (1.50-1.81)	1.39 (1.26-1.54)	1.52 (1.40-1.65)
	3+	1.94 (1.62-2.32)	1.89 (1.69-2.11)	1.79 (1.62-1.97)	1.72 (1.56-1.89)	1.48 (1.33-1.64)	1.56 (1.42-1.70)
Pregnancy		-	0.90 (0.77-1.05)	1.03 (0.90-1.19)	1.18 (1.01-1.38)	1.12 (0.94-1.34)	1.33 (1.13-1.57)

* Estimates correspond to a 1-year increase in age (or age-squared as indicated) within the given age categories.

Supplementary Table 27: Covariate hazard ratios for Non-COVID-19 death in the BNT162b2 vs unvaccinated comparison and the 18-64 years and clinically vulnerable subgroup.

		Weeks since second dose					
**Variable**	**Category**	**3-6**	**7-10**	**11-14**	**15-18**	**19-22**	**23-26**
Age*	18-64	1.17 (1.04-1.32)	1.11 (0.98-1.26)	1.10 (0.98-1.23)	1.19 (1.03-1.38)	1.12 (0.99-1.25)	1.13 (0.99-1.28)
	18-64 squared	1.00 (1.00-1.00)	1.00 (1.00-1.00)	1.00 (1.00-1.00)	1.00 (1.00-1.00)	1.00 (1.00-1.00)	1.00 (1.00-1.00)
Sex	Female	1.00	1.00	1.00	1.00	1.00	1.00
	Male	1.89 (1.46-2.46)	1.43 (1.12-1.82)	1.57 (1.23-2.01)	1.35 (1.04-1.74)	1.77 (1.36-2.30)	1.51 (1.17-1.94)
IMD	1	1.00	1.00	1.00	1.00	1.00	1.00
	2	0.84 (0.61-1.17)	0.82 (0.60-1.13)	0.75 (0.54-1.05)	0.78 (0.55-1.11)	0.69 (0.47-1.01)	0.93 (0.67-1.29)
	3	0.87 (0.61-1.23)	0.74 (0.52-1.05)	0.68 (0.47-1.00)	0.70 (0.48-1.02)	0.84 (0.58-1.23)	0.85 (0.59-1.22)
	4	0.62 (0.41-0.95)	0.78 (0.54-1.13)	0.87 (0.60-1.26)	0.87 (0.60-1.25)	0.95 (0.65-1.39)	-
	5	0.70 (0.44-1.12)	0.54 (0.34-0.87)	0.70 (0.46-1.08)	0.56 (0.35-0.90)	0.78 (0.51-1.20)	-
	4 / 5	-	-	-	-	-	0.62 (0.44-0.86)
Ethnicity	White	1.00	1.00	1.00	1.00	1.00	1.00
	Black	0.52 (0.27-1.02)	-	0.22 (0.08-0.60)	-	0.99 (0.55-1.76)	0.84 (0.43-1.64)
	South Asian / Mixed / Other	0.40 (0.24-0.67)	-	0.59 (0.38-0.92)	-	0.42 (0.24-0.73)	0.90 (0.57-1.40)
	Black / South Asian / Mixed / Other	-	0.56 (0.38-0.83)	-	0.53 (0.34-0.84)	-	-
BMI	<30 or missing	1.00	1.00	1.00	1.00	1.00	1.00
	40+	1.11 (0.74-1.68)	0.73 (0.46-1.14)	1.08 (0.73-1.59)	0.77 (0.49-1.23)	0.82 (0.54-1.24)	1.03 (0.68-1.54)
	30-34.9	0.77 (0.54-1.10)	0.59 (0.42-0.84)	0.71 (0.50-0.99)	0.75 (0.53-1.05)	0.53 (0.36-0.78)	0.66 (0.47-0.94)
	35-39.9	1.09 (0.73-1.63)	0.79 (0.53-1.19)	0.50 (0.29-0.84)	0.86 (0.57-1.31)	0.54 (0.32-0.90)	0.91 (0.61-1.35)
Learning disability		1.29 (0.62-2.69)	-	1.06 (0.47-2.39)	-	2.19 (1.14-4.20)	2.18 (1.11-4.29)
Serious mental illness		1.73 (1.11-2.69)	1.65 (1.06-2.56)	1.58 (1.01-2.47)	0.99 (0.55-1.76)	1.12 (0.66-1.89)	1.49 (0.93-2.36)
Morbidity count	0	1.00	1.00	1.00	1.00	1.00	1.00
	1	1.25 (0.83-1.90)	2.15 (1.33-3.47)	1.09 (0.74-1.63)	1.22 (0.77-1.95)	0.77 (0.52-1.14)	1.67 (1.05-2.64)
	2+	2.87 (1.87-4.41)	4.19 (2.53-6.93)	2.15 (1.39-3.33)	2.62 (1.64-4.19)	1.64 (1.09-2.46)	3.28 (2.03-5.30)
Flu vaccine		0.85 (0.63-1.16)	1.07 (0.80-1.44)	1.32 (0.98-1.78)	1.03 (0.76-1.39)	0.94 (0.69-1.27)	1.07 (0.80-1.43)
Number of SARS-CoV-2 tests	0	1.00	1.00	1.00	1.00	1.00	1.00
	1	0.89 (0.60-1.33)	1.17 (0.82-1.67)	1.52 (1.09-2.10)	1.67 (1.19-2.35)	1.36 (0.97-1.92)	1.17 (0.82-1.67)
	2	1.68 (1.04-2.71)	1.99 (1.29-3.08)	1.96 (1.24-3.12)	1.96 (1.21-3.17)	1.90 (1.19-3.02)	1.38 (0.81-2.36)
	3+	5.02 (3.73-6.75)	4.68 (3.44-6.36)	4.88 (3.59-6.65)	6.00 (4.41-8.17)	3.32 (2.30-4.79)	5.92 (4.39-7.99)

* Estimates correspond to a 1-year increase in age (or age-squared as indicated) within the given age categories.

Supplementary Table 28: Covariate hazard ratios for Any SARS-CoV-2 test in the BNT162b2 vs unvaccinated comparison and the 18-64 years and clinically vulnerable subgroup.

		Weeks since second dose					
**Variable**	**Category**	**3-6**	**7-10**	**11-14**	**15-18**	**19-22**	**23-26**
Age*	18-64	1.02 (1.01-1.02)	1.02 (1.01-1.02)	1.01 (1.01-1.02)	1.01 (1.01-1.02)	1.03 (1.02-1.03)	1.03 (1.03-1.04)
	18-64 squared	1.00 (1.00-1.00)	1.00 (1.00-1.00)	1.00 (1.00-1.00)	1.00 (1.00-1.00)	1.00 (1.00-1.00)	1.00 (1.00-1.00)
Sex	Female	1.00	1.00	1.00	1.00	1.00	1.00
	Male	0.73 (0.72-0.74)	0.75 (0.74-0.77)	0.80 (0.79-0.81)	0.78 (0.77-0.80)	0.78 (0.76-0.79)	0.78 (0.76-0.79)
IMD	1	1.00	1.00	1.00	1.00	1.00	1.00
	2	1.11 (1.08-1.13)	1.12 (1.09-1.14)	1.11 (1.08-1.13)	1.13 (1.10-1.15)	1.14 (1.12-1.17)	1.15 (1.12-1.17)
	3	1.19 (1.16-1.21)	1.22 (1.19-1.25)	1.20 (1.18-1.23)	1.24 (1.21-1.27)	1.27 (1.24-1.30)	1.29 (1.26-1.32)
	4	1.27 (1.24-1.30)	1.30 (1.27-1.33)	1.27 (1.24-1.30)	1.32 (1.29-1.35)	1.37 (1.34-1.40)	1.40 (1.37-1.43)
	5	1.37 (1.33-1.40)	1.44 (1.41-1.48)	1.40 (1.37-1.43)	1.46 (1.43-1.50)	1.53 (1.49-1.56)	1.57 (1.54-1.61)
Ethnicity	White	1.00	1.00	1.00	1.00	1.00	1.00
	Black	0.96 (0.92-1.01)	0.92 (0.88-0.95)	0.96 (0.92-1.00)	0.94 (0.90-0.98)	0.94 (0.90-0.98)	0.90 (0.86-0.95)
	South Asian	0.69 (0.66-0.71)	0.64 (0.62-0.66)	0.65 (0.63-0.67)	0.66 (0.63-0.68)	0.67 (0.64-0.69)	0.70 (0.68-0.72)
	Mixed	0.91 (0.86-0.97)	0.88 (0.83-0.93)	0.94 (0.89-1.00)	0.97 (0.92-1.03)	0.90 (0.85-0.96)	0.90 (0.85-0.96)
	Other	0.73 (0.68-0.78)	0.71 (0.67-0.75)	0.78 (0.73-0.83)	0.78 (0.73-0.83)	0.81 (0.76-0.86)	0.76 (0.71-0.81)
BMI	<30 or missing	1.00	1.00	1.00	1.00	1.00	1.00
	40+	0.98 (0.96-1.00)	0.96 (0.94-0.99)	0.98 (0.95-1.00)	0.95 (0.92-0.97)	0.96 (0.94-0.98)	0.97 (0.95-0.99)
	30-34.9	1.01 (0.99-1.03)	1.00 (0.98-1.02)	1.01 (0.99-1.03)	0.99 (0.97-1.01)	1.00 (0.98-1.02)	1.01 (0.99-1.03)
	35-39.9	0.98 (0.95-1.01)	0.97 (0.95-0.99)	0.96 (0.93-0.98)	0.97 (0.95-1.00)	0.98 (0.96-1.00)	0.96 (0.93-0.98)
Learning disability		1.08 (1.03-1.12)	0.92 (0.88-0.96)	0.95 (0.91-0.99)	0.98 (0.94-1.02)	0.94 (0.90-0.98)	0.94 (0.90-0.98)
Serious mental illness		0.81 (0.78-0.84)	0.77 (0.75-0.80)	0.80 (0.78-0.83)	0.77 (0.75-0.80)	0.74 (0.72-0.77)	0.77 (0.74-0.80)
Morbidity count	0	1.00	1.00	1.00	1.00	1.00	1.00
	1	0.98 (0.96-1.01)	0.98 (0.96-1.01)	0.97 (0.95-0.99)	0.98 (0.96-1.00)	0.96 (0.94-0.99)	0.96 (0.94-0.98)
	2+	0.94 (0.91-0.97)	0.92 (0.90-0.95)	0.92 (0.90-0.95)	0.92 (0.89-0.94)	0.92 (0.90-0.94)	0.91 (0.89-0.93)
Flu vaccine		1.26 (1.24-1.29)	1.23 (1.21-1.25)	1.21 (1.19-1.23)	1.22 (1.20-1.24)	1.21 (1.19-1.23)	1.22 (1.20-1.25)
Number of SARS-CoV-2 tests	0	1.00	1.00	1.00	1.00	1.00	1.00
	1	1.52 (1.49-1.55)	1.52 (1.50-1.55)	1.49 (1.46-1.51)	1.45 (1.42-1.47)	1.43 (1.41-1.46)	1.42 (1.39-1.44)
	2	1.90 (1.85-1.95)	1.84 (1.80-1.89)	1.82 (1.77-1.87)	1.79 (1.74-1.84)	1.71 (1.66-1.75)	1.70 (1.66-1.75)
	3+	3.18 (3.11-3.27)	2.92 (2.85-2.99)	2.75 (2.68-2.82)	2.64 (2.58-2.71)	2.38 (2.32-2.44)	2.34 (2.28-2.41)
Pregnancy		2.00 (1.91-2.09)	1.89 (1.81-1.98)	1.76 (1.67-1.85)	1.69 (1.60-1.78)	1.41 (1.32-1.50)	1.39 (1.30-1.49)

* Estimates correspond to a 1-year increase in age (or age-squared as indicated) within the given age categories.

Supplementary Table 29: Covariate hazard ratios for COVID-19 hospitalisation in the ChAdOx1 vs unvaccinated comparison and the 18-64 years and clinically vulnerable subgroup.

		Weeks since second dose					
**Variable**	**Category**	**3-6**	**7-10**	**11-14**	**15-18**	**19-22**	**23-26**
Age*	18-64	1.13 (1.03-1.24)	0.99 (0.94-1.04)	1.01 (0.97-1.05)	1.03 (0.99-1.08)	1.06 (1.01-1.12)	1.06 (1.02-1.11)
	18-64 squared	1.00 (1.00-1.00)	1.00 (1.00-1.00)	1.00 (1.00-1.00)	1.00 (1.00-1.00)	1.00 (1.00-1.00)	1.00 (1.00-1.00)
Sex	Female	1.00	1.00	1.00	1.00	1.00	1.00
	Male	0.79 (0.59-1.07)	1.04 (0.88-1.22)	1.00 (0.88-1.15)	1.08 (0.94-1.23)	1.16 (1.01-1.34)	1.14 (1.01-1.30)
IMD	1	1.00	1.00	1.00	1.00	1.00	1.00
	2 / 3 / 4 / 5	0.76 (0.55-1.05)	-	-	-	-	-
	2	-	1.03 (0.83-1.27)	1.20 (1.01-1.43)	0.94 (0.78-1.12)	0.93 (0.77-1.13)	1.05 (0.88-1.25)
	3	-	1.23 (0.98-1.56)	1.02 (0.83-1.25)	0.85 (0.69-1.04)	1.03 (0.83-1.27)	1.07 (0.88-1.29)
	4	-	0.83 (0.62-1.11)	1.01 (0.80-1.27)	0.94 (0.76-1.16)	0.93 (0.73-1.17)	0.83 (0.66-1.03)
	5	-	0.89 (0.65-1.23)	1.06 (0.82-1.38)	0.88 (0.69-1.13)	0.85 (0.65-1.10)	0.94 (0.75-1.19)
Ethnicity	White	1.00	1.00	1.00	1.00	1.00	1.00
	Black	1.61 (0.94-2.73)	1.33 (0.97-1.80)	1.55 (1.20-2.00)	1.33 (1.02-1.74)	1.41 (1.06-1.86)	0.79 (0.56-1.11)
	South Asian	2.06 (1.42-2.99)	1.24 (0.95-1.61)	1.63 (1.33-1.99)	1.31 (1.05-1.64)	1.31 (1.04-1.66)	0.99 (0.78-1.27)
	Mixed / Other	0.77 (0.35-1.69)	1.24 (0.88-1.75)	-	-	-	-
	Mixed	-	-	1.11 (0.71-1.74)	1.40 (0.94-2.10)	1.66 (1.11-2.48)	1.29 (0.86-1.95)
	Other	-	-	2.52 (1.86-3.40)	0.77 (0.46-1.30)	0.89 (0.52-1.52)	0.79 (0.48-1.30)
BMI	<30 or missing	1.00	1.00	1.00	1.00	1.00	1.00
	40+	1.89 (1.26-2.84)	2.67 (2.11-3.39)	2.31 (1.88-2.83)	2.98 (2.48-3.58)	2.92 (2.41-3.54)	2.86 (2.39-3.42)
	30-34.9	1.14 (0.73-1.76)	1.47 (1.17-1.85)	1.78 (1.49-2.13)	1.64 (1.37-1.96)	1.62 (1.34-1.96)	1.36 (1.13-1.64)
	35-39.9	2.04 (1.31-3.16)	2.47 (1.92-3.17)	2.24 (1.81-2.76)	1.71 (1.36-2.15)	1.98 (1.58-2.49)	2.06 (1.69-2.53)
Learning disability		-	1.12 (0.69-1.81)	0.69 (0.42-1.13)	1.00 (0.66-1.52)	1.22 (0.80-1.84)	1.08 (0.72-1.61)
Serious mental illness		-	0.63 (0.42-0.93)	0.72 (0.53-0.98)	0.90 (0.66-1.22)	0.81 (0.58-1.11)	0.74 (0.54-1.01)
Morbidity count	0	1.00	1.00	1.00	1.00	1.00	1.00
	1	0.92 (0.63-1.34)	1.18 (0.92-1.53)	1.15 (0.94-1.42)	1.50 (1.22-1.83)	1.27 (1.03-1.57)	1.19 (0.98-1.45)
	2+	1.45 (0.89-2.37)	1.75 (1.30-2.34)	1.64 (1.30-2.07)	2.50 (2.01-3.12)	1.93 (1.52-2.45)	1.94 (1.56-2.42)
Flu vaccine		1.31 (0.96-1.79)	1.09 (0.91-1.30)	1.17 (1.01-1.36)	1.13 (0.97-1.32)	1.24 (1.05-1.45)	1.25 (1.08-1.45)
Number of SARS-CoV-2 tests	0	1.00	1.00	1.00	1.00	1.00	1.00
	1	1.15 (0.78-1.70)	1.52 (1.23-1.87)	1.38 (1.16-1.64)	1.17 (0.97-1.40)	1.57 (1.31-1.87)	1.32 (1.11-1.57)
	2 / 3+	1.35 (0.88-2.08)	-	-	-	-	-
	2	-	1.62 (1.19-2.21)	1.48 (1.13-1.92)	1.64 (1.29-2.09)	2.08 (1.62-2.66)	1.35 (1.04-1.76)
	3+	-	2.16 (1.64-2.86)	1.96 (1.56-2.48)	1.98 (1.59-2.47)	2.38 (1.88-3.01)	2.57 (2.09-3.17)

* Estimates correspond to a 1-year increase in age (or age-squared as indicated) within the given age categories.

Supplementary Table 30: Covariate hazard ratios for COVID-19 death in the ChAdOx1 vs unvaccinated comparison and the 18-64 years and clinically vulnerable subgroup.

		Weeks since second dose					
**Variable**	**Category**	**3-6**	**7-10**	**11-14**	**15-18**	**19-22**	**23-26**
Age*	18-64	-	1.09 (0.84-1.40)	1.23 (0.99-1.53)	1.10 (0.95-1.29)	1.15 (0.94-1.41)	1.02 (0.87-1.20)
	18-64 squared	-	1.00 (1.00-1.00)	1.00 (1.00-1.00)	1.00 (1.00-1.00)	1.00 (1.00-1.00)	1.00 (1.00-1.00)
Sex	Female	-	1.00	1.00	1.00	1.00	1.00
	Male	-	1.27 (0.62-2.60)	2.82 (1.71-4.67)	1.15 (0.77-1.74)	1.84 (1.22-2.79)	1.94 (1.23-3.06)
IMD	1	-	1.00	1.00	1.00	1.00	1.00
	2 / 3 / 4 / 5	-	0.63 (0.29-1.39)	-	-	-	-
	2	-	-	0.62 (0.33-1.17)	1.15 (0.71-1.88)	0.95 (0.54-1.70)	1.50 (0.81-2.78)
	3 / 4 / 5	-	-	0.64 (0.37-1.11)	-	-	-
	3	-	-	-	0.89 (0.48-1.66)	1.36 (0.79-2.33)	1.86 (0.97-3.56)
	4 / 5	-	-	-	0.69 (0.37-1.26)	-	-
	4	-	-	-	-	0.80 (0.39-1.64)	1.17 (0.54-2.51)
	5	-	-	-	-	0.74 (0.33-1.68)	1.90 (0.89-4.07)
Ethnicity	White	-	1.00	1.00	1.00	1.00	1.00
	Black / South Asian / Mixed / Other	-	0.63 (0.22-1.79)	1.51 (0.89-2.57)	0.98 (0.57-1.70)	-	1.06 (0.55-2.03)
	Black	-	-	-	-	1.66 (0.77-3.59)	-
	South Asian / Mixed / Other	-	-	-	-	1.74 (1.00-3.05)	-
BMI	<30 or missing	-	1.00	1.00	1.00	1.00	1.00
	40+ / 30-34.9 / 35-39.9	-	1.80 (0.81-4.01)	-	-	-	-
	40+	-	-	3.27 (1.71-6.24)	4.87 (2.94-8.08)	4.90 (2.91-8.25)	5.67 (3.19-10.09)
	30-34.9	-	-	1.24 (0.66-2.34)	-	1.65 (0.95-2.88)	2.29 (1.29-4.04)
	35-39.9	-	-	2.70 (1.42-5.15)	-	2.07 (1.05-4.10)	2.52 (1.26-5.06)
	30-34.9 / 35-39.9	-	-	-	1.83 (1.13-2.96)	-	-
Serious mental illness		-	-	-	-	-	1.97 (0.92-4.22)
Morbidity count	0	-	1.00	1.00	1.00	1.00	1.00
	1 / 2+	-	0.73 (0.29-1.85)	2.51 (1.11-5.67)	1.64 (0.93-2.87)	1.41 (0.83-2.42)	-
	1	-	-	-	-	-	1.48 (0.75-2.91)
	2+	-	-	-	-	-	2.21 (1.04-4.66)
Flu vaccine		-	-	1.29 (0.78-2.11)	0.95 (0.62-1.44)	1.33 (0.86-2.06)	1.27 (0.81-1.98)
Number of SARS-CoV-2 tests	0	-	1.00	1.00	1.00	1.00	1.00
	1 / 2 / 3+	-	1.33 (0.61-2.91)	1.00 (0.59-1.69)	-	-	1.58 (1.00-2.49)
	1	-	-	-	0.88 (0.47-1.65)	0.94 (0.51-1.75)	-
	2 / 3+	-	-	-	1.50 (0.85-2.66)	2.03 (1.22-3.40)	-

* Estimates correspond to a 1-year increase in age (or age-squared as indicated) within the given age categories.

Supplementary Table 31: Covariate hazard ratios for Positive SARS-CoV-2 test in the ChAdOx1 vs unvaccinated comparison and the 18-64 years and clinically vulnerable subgroup.

		Weeks since second dose					
**Variable**	**Category**	**3-6**	**7-10**	**11-14**	**15-18**	**19-22**	**23-26**
Age*	18-64	0.97 (0.95-0.99)	0.97 (0.96-0.98)	0.97 (0.96-0.98)	1.04 (1.03-1.05)	1.10 (1.09-1.11)	1.10 (1.09-1.11)
	18-64 squared	1.00 (1.00-1.00)	1.00 (1.00-1.00)	1.00 (1.00-1.00)	1.00 (1.00-1.00)	1.00 (1.00-1.00)	1.00 (1.00-1.00)
Sex	Female	1.00	1.00	1.00	1.00	1.00	1.00
	Male	0.91 (0.84-0.98)	0.97 (0.93-1.01)	0.94 (0.91-0.98)	0.86 (0.83-0.90)	0.84 (0.81-0.87)	0.84 (0.82-0.87)
IMD	1	1.00	1.00	1.00	1.00	1.00	1.00
	2	1.06 (0.96-1.18)	1.02 (0.96-1.08)	1.04 (0.98-1.09)	1.08 (1.02-1.14)	1.10 (1.05-1.16)	1.14 (1.09-1.20)
	3	1.15 (1.03-1.29)	1.08 (1.01-1.15)	1.03 (0.97-1.09)	1.03 (0.97-1.09)	1.16 (1.09-1.22)	1.21 (1.15-1.27)
	4	1.14 (1.01-1.28)	1.04 (0.97-1.12)	1.05 (0.99-1.11)	1.06 (1.00-1.13)	1.20 (1.13-1.27)	1.23 (1.17-1.30)
	5	1.28 (1.13-1.45)	1.17 (1.09-1.25)	1.00 (0.94-1.07)	1.05 (0.98-1.12)	1.25 (1.18-1.33)	1.27 (1.20-1.33)
Ethnicity	White	1.00	1.00	1.00	1.00	1.00	1.00
	Black	1.05 (0.87-1.27)	0.96 (0.85-1.07)	1.03 (0.93-1.13)	0.93 (0.83-1.03)	0.85 (0.76-0.95)	0.65 (0.58-0.73)
	South Asian	1.17 (1.04-1.31)	0.77 (0.71-0.83)	0.92 (0.86-0.98)	0.86 (0.80-0.93)	0.84 (0.79-0.91)	0.79 (0.74-0.85)
	Mixed	1.22 (0.96-1.55)	1.04 (0.89-1.21)	0.89 (0.77-1.03)	1.05 (0.92-1.21)	0.99 (0.86-1.14)	0.87 (0.76-1.00)
	Other	0.87 (0.67-1.14)	0.56 (0.46-0.68)	0.81 (0.70-0.94)	0.60 (0.51-0.72)	0.68 (0.58-0.80)	0.75 (0.66-0.86)
BMI	<30 or missing	1.00	1.00	1.00	1.00	1.00	1.00
	40+	0.95 (0.84-1.08)	0.99 (0.92-1.07)	1.12 (1.06-1.19)	1.18 (1.11-1.25)	1.16 (1.10-1.23)	1.15 (1.10-1.21)
	30-34.9	1.09 (0.98-1.22)	1.17 (1.10-1.24)	1.14 (1.08-1.20)	1.15 (1.09-1.21)	1.14 (1.09-1.20)	1.17 (1.12-1.22)
	35-39.9	1.23 (1.08-1.41)	1.18 (1.09-1.27)	1.14 (1.07-1.22)	1.24 (1.16-1.32)	1.22 (1.15-1.30)	1.19 (1.13-1.26)
Learning disability		0.38 (0.29-0.50)	0.38 (0.32-0.44)	0.47 (0.41-0.54)	0.53 (0.46-0.60)	0.58 (0.51-0.66)	0.63 (0.57-0.70)
Serious mental illness		0.51 (0.42-0.61)	0.55 (0.49-0.61)	0.58 (0.53-0.64)	0.56 (0.51-0.62)	0.50 (0.45-0.55)	0.51 (0.46-0.55)
Morbidity count	0	1.00	1.00	1.00	1.00	1.00	1.00
	1	0.92 (0.81-1.03)	0.97 (0.91-1.04)	0.95 (0.90-1.01)	0.99 (0.93-1.04)	0.96 (0.91-1.01)	0.94 (0.90-0.99)
	2+	0.81 (0.68-0.96)	0.89 (0.82-0.98)	0.94 (0.87-1.01)	0.97 (0.91-1.05)	0.88 (0.82-0.94)	0.85 (0.80-0.91)
Flu vaccine		0.99 (0.91-1.07)	0.96 (0.92-1.01)	0.99 (0.95-1.04)	1.05 (1.00-1.09)	1.08 (1.04-1.12)	1.05 (1.01-1.09)
Number of SARS-CoV-2 tests	0	1.00	1.00	1.00	1.00	1.00	1.00
	1	1.53 (1.40-1.68)	1.60 (1.52-1.69)	1.43 (1.37-1.50)	1.39 (1.33-1.46)	1.31 (1.25-1.37)	1.33 (1.28-1.39)
	2	1.76 (1.54-2.01)	1.54 (1.42-1.66)	1.53 (1.43-1.64)	1.47 (1.38-1.58)	1.48 (1.39-1.58)	1.38 (1.30-1.47)
	3+	1.81 (1.57-2.09)	1.73 (1.59-1.88)	1.60 (1.49-1.72)	1.66 (1.55-1.78)	1.50 (1.40-1.62)	1.48 (1.38-1.57)
Pregnancy		0.87 (0.70-1.09)	0.89 (0.77-1.03)	1.03 (0.90-1.17)	1.09 (0.95-1.27)	1.09 (0.93-1.27)	1.19 (1.03-1.38)

* Estimates correspond to a 1-year increase in age (or age-squared as indicated) within the given age categories.

Supplementary Table 32: Covariate hazard ratios for Non-COVID-19 death in the ChAdOx1 vs unvaccinated comparison and the 18-64 years and clinically vulnerable subgroup.

		**Weeks since second dose**					
**Variable**	**Category**	**3-6**	**7-10**	**11-14**	**15-18**	**19-22**	**23-26**
Age*	18-64	1.15 (1.04-1.28)	1.03 (0.93-1.13)	1.17 (1.05-1.30)	1.20 (1.05-1.37)	1.13 (1.03-1.25)	1.10 (1.01-1.21)
	18-64 squared	1.00 (1.00-1.00)	1.00 (1.00-1.00)	1.00 (1.00-1.00)	1.00 (1.00-1.00)	1.00 (1.00-1.00)	1.00 (1.00-1.00)
Sex	Female	1.00	1.00	1.00	1.00	1.00	1.00
	Male	1.78 (1.41-2.25)	1.28 (1.05-1.57)	1.45 (1.19-1.77)	1.38 (1.13-1.68)	1.65 (1.35-2.03)	1.43 (1.17-1.76)
IMD	1	1.00	1.00	1.00	1.00	1.00	1.00
	2	1.10 (0.82-1.47)	0.82 (0.62-1.08)	0.93 (0.70-1.22)	0.83 (0.64-1.09)	0.83 (0.63-1.10)	0.82 (0.63-1.07)
	3	0.84 (0.60-1.18)	0.71 (0.53-0.96)	0.92 (0.69-1.24)	0.80 (0.60-1.07)	0.84 (0.62-1.12)	0.63 (0.47-0.85)
	4	0.72 (0.50-1.05)	0.69 (0.50-0.95)	0.87 (0.63-1.19)	0.60 (0.43-0.84)	0.61 (0.43-0.86)	-
	5	0.70 (0.47-1.07)	0.71 (0.51-0.99)	0.77 (0.55-1.09)	0.62 (0.44-0.87)	0.81 (0.58-1.13)	-
	4 / 5	-	-	-	-	-	0.56 (0.42-0.73)
Ethnicity	White	1.00	1.00	1.00	1.00	1.00	1.00
	Black / South Asian / Mixed / Other	0.48 (0.32-0.71)	0.51 (0.36-0.72)	-	0.61 (0.42-0.88)	-	0.63 (0.44-0.91)
	Black	-	-	0.44 (0.22-0.87)	-	1.00 (0.60-1.67)	-
	South Asian / Mixed / Other	-	-	0.64 (0.44-0.94)	-	0.54 (0.36-0.83)	-
BMI	<30 or missing	1.00	1.00	1.00	1.00	1.00	1.00
	40+	1.01 (0.69-1.48)	0.77 (0.53-1.12)	0.80 (0.56-1.14)	0.82 (0.58-1.17)	0.84 (0.59-1.18)	0.85 (0.60-1.19)
	30-34.9	0.89 (0.67-1.19)	0.79 (0.60-1.04)	0.78 (0.60-1.02)	0.60 (0.45-0.80)	0.67 (0.50-0.89)	0.74 (0.56-0.96)
	35-39.9	0.59 (0.37-0.93)	0.85 (0.60-1.20)	0.72 (0.50-1.04)	0.72 (0.51-1.04)	0.89 (0.64-1.25)	0.55 (0.37-0.82)
Learning disability		0.93 (0.44-1.96)	-	1.38 (0.77-2.46)	-	1.04 (0.53-2.03)	1.54 (0.88-2.72)
Serious mental illness		1.83 (1.23-2.75)	1.76 (1.22-2.55)	1.41 (0.95-2.09)	1.34 (0.89-2.02)	1.17 (0.76-1.79)	1.42 (0.95-2.12)
Morbidity count	0	1.00	1.00	1.00	1.00	1.00	1.00
	1	1.28 (0.86-1.90)	1.49 (1.03-2.14)	1.41 (1.01-1.96)	1.18 (0.84-1.65)	0.79 (0.58-1.09)	1.15 (0.83-1.61)
	2+	2.77 (1.83-4.19)	3.16 (2.17-4.61)	2.03 (1.43-2.88)	2.29 (1.63-3.24)	1.67 (1.20-2.32)	2.11 (1.49-2.98)
Flu vaccine		0.98 (0.75-1.28)	1.06 (0.83-1.36)	1.05 (0.82-1.36)	0.81 (0.63-1.03)	1.02 (0.80-1.31)	1.00 (0.78-1.28)
Number of SARS-CoV-2 tests	0	1.00	1.00	1.00	1.00	1.00	1.00
	1	1.09 (0.78-1.51)	1.14 (0.85-1.52)	1.44 (1.10-1.89)	1.02 (0.76-1.38)	0.94 (0.69-1.27)	0.98 (0.73-1.33)
	2	1.82 (1.19-2.78)	1.73 (1.19-2.52)	1.78 (1.21-2.62)	1.70 (1.17-2.48)	2.24 (1.60-3.12)	1.25 (0.82-1.93)
	3+	5.21 (3.97-6.84)	4.26 (3.30-5.49)	5.28 (4.12-6.76)	4.96 (3.87-6.36)	4.15 (3.20-5.38)	5.08 (3.96-6.51)

* Estimates correspond to a 1-year increase in age (or age-squared as indicated) within the given age categories.

Supplementary Table 33: Covariate hazard ratios for Any SARS-CoV-2 test in the ChAdOx1 vs unvaccinated comparison and the 18-64 years and clinically vulnerable subgroup.

		Weeks since second dose					
**Variable**	**Category**	**3-6**	**7-10**	**11-14**	**15-18**	**19-22**	**23-26**
Age*	18-64	1.02 (1.02-1.03)	1.02 (1.02-1.03)	1.01 (1.01-1.01)	1.02 (1.02-1.03)	1.04 (1.04-1.04)	1.04 (1.04-1.05)
	18-64 squared	1.00 (1.00-1.00)	1.00 (1.00-1.00)	1.00 (1.00-1.00)	1.00 (1.00-1.00)	1.00 (1.00-1.00)	1.00 (1.00-1.00)
Sex	Female	1.00	1.00	1.00	1.00	1.00	1.00
	Male	0.72 (0.71-0.73)	0.76 (0.75-0.77)	0.81 (0.80-0.82)	0.78 (0.77-0.79)	0.77 (0.76-0.78)	0.77 (0.76-0.78)
IMD	1	1.00	1.00	1.00	1.00	1.00	1.00
	2	1.14 (1.11-1.16)	1.12 (1.10-1.14)	1.12 (1.10-1.15)	1.13 (1.11-1.15)	1.14 (1.12-1.16)	1.16 (1.14-1.18)
	3	1.22 (1.20-1.25)	1.22 (1.20-1.24)	1.21 (1.18-1.23)	1.22 (1.20-1.24)	1.24 (1.22-1.27)	1.27 (1.24-1.29)
	4	1.30 (1.28-1.33)	1.30 (1.28-1.33)	1.29 (1.26-1.31)	1.32 (1.29-1.34)	1.37 (1.35-1.40)	1.37 (1.35-1.40)
	5	1.43 (1.40-1.46)	1.45 (1.42-1.47)	1.40 (1.37-1.42)	1.44 (1.42-1.47)	1.53 (1.50-1.56)	1.54 (1.51-1.57)
Ethnicity	White	1.00	1.00	1.00	1.00	1.00	1.00
	Black	0.92 (0.89-0.95)	0.86 (0.83-0.89)	0.94 (0.90-0.97)	0.94 (0.91-0.98)	0.90 (0.86-0.93)	0.87 (0.84-0.90)
	South Asian	0.64 (0.62-0.65)	0.59 (0.57-0.60)	0.62 (0.61-0.64)	0.62 (0.60-0.64)	0.66 (0.64-0.68)	0.67 (0.65-0.68)
	Mixed	0.93 (0.88-0.98)	0.87 (0.83-0.91)	0.90 (0.86-0.95)	0.92 (0.88-0.97)	0.91 (0.87-0.96)	0.90 (0.86-0.95)
	Other	0.72 (0.69-0.76)	0.70 (0.66-0.73)	0.75 (0.71-0.78)	0.76 (0.72-0.80)	0.75 (0.72-0.79)	0.76 (0.73-0.80)
BMI	<30 or missing	1.00	1.00	1.00	1.00	1.00	1.00
	40+	0.94 (0.92-0.96)	0.95 (0.93-0.97)	0.95 (0.93-0.97)	0.95 (0.93-0.97)	0.95 (0.93-0.97)	0.95 (0.93-0.96)
	30-34.9	0.99 (0.97-1.01)	1.00 (0.99-1.02)	0.99 (0.97-1.00)	0.98 (0.97-1.00)	1.00 (0.98-1.01)	0.99 (0.98-1.01)
	35-39.9	0.98 (0.95-1.00)	1.00 (0.98-1.02)	0.96 (0.94-0.98)	0.97 (0.95-0.99)	0.98 (0.96-1.00)	0.98 (0.96-0.99)
Learning disability		1.02 (0.98-1.05)	0.92 (0.89-0.95)	0.97 (0.94-1.00)	1.02 (0.99-1.05)	0.95 (0.92-0.98)	1.00 (0.97-1.03)
Serious mental illness		0.77 (0.75-0.79)	0.75 (0.73-0.77)	0.79 (0.76-0.81)	0.76 (0.74-0.79)	0.75 (0.73-0.77)	0.74 (0.72-0.76)
Morbidity count	0	1.00	1.00	1.00	1.00	1.00	1.00
	1	0.96 (0.94-0.98)	0.96 (0.94-0.97)	0.96 (0.94-0.98)	0.99 (0.97-1.01)	0.97 (0.95-0.99)	0.96 (0.95-0.98)
	2+	0.90 (0.88-0.92)	0.89 (0.87-0.91)	0.90 (0.88-0.92)	0.92 (0.91-0.94)	0.89 (0.87-0.91)	0.89 (0.87-0.91)
Flu vaccine		1.24 (1.22-1.25)	1.21 (1.19-1.22)	1.19 (1.17-1.21)	1.18 (1.17-1.20)	1.18 (1.17-1.20)	1.20 (1.19-1.22)
Number of SARS-CoV-2 tests	0	1.00	1.00	1.00	1.00	1.00	1.00
	1	1.48 (1.46-1.50)	1.49 (1.47-1.51)	1.45 (1.42-1.47)	1.41 (1.39-1.43)	1.40 (1.39-1.42)	1.38 (1.36-1.40)
	2	1.82 (1.78-1.85)	1.78 (1.75-1.82)	1.72 (1.69-1.76)	1.71 (1.67-1.74)	1.68 (1.64-1.71)	1.66 (1.62-1.69)
	3+	3.05 (2.99-3.11)	2.83 (2.78-2.89)	2.70 (2.65-2.75)	2.61 (2.56-2.66)	2.42 (2.38-2.47)	2.36 (2.31-2.40)
Pregnancy		1.74 (1.67-1.82)	1.66 (1.59-1.74)	1.57 (1.49-1.64)	1.47 (1.40-1.55)	1.23 (1.16-1.30)	1.23 (1.16-1.31)

* Estimates correspond to a 1-year increase in age (or age-squared as indicated) within the given age categories.

Supplementary Table 34: Covariate hazard ratios for COVID-19 hospitalisation in the BNT162b2 vs ChAdOx1 comparison and the 18-64 years and clinically vulnerable subgroup.

		Weeks since second dose					
**Variable**	**Category**	**3-6**	**7-10**	**11-14**	**15-18**	**19-22**	**23-26**
Age*	18-64	1.11 (0.87-1.40)	0.96 (0.86-1.07)	0.99 (0.92-1.06)	1.08 (1.00-1.15)	1.04 (0.97-1.11)	1.09 (1.02-1.15)
	18-64 squared	1.00 (1.00-1.00)	1.00 (1.00-1.00)	1.00 (1.00-1.00)	1.00 (1.00-1.00)	1.00 (1.00-1.00)	1.00 (1.00-1.00)
Sex	Female	-	1.00	1.00	1.00	1.00	1.00
	Male	-	1.38 (1.03-1.86)	1.26 (1.02-1.56)	1.20 (1.01-1.43)	1.15 (0.96-1.37)	1.34 (1.14-1.58)
IMD	1	1.00	1.00	1.00	1.00	1.00	1.00
	2 / 3 / 4 / 5	0.56 (0.26-1.21)	-	-	-	-	-
	2	-	1.13 (0.76-1.67)	1.03 (0.76-1.39)	0.94 (0.72-1.22)	0.67 (0.51-0.88)	0.96 (0.76-1.20)
	3	-	0.98 (0.64-1.52)	0.77 (0.55-1.08)	0.90 (0.68-1.17)	0.86 (0.66-1.13)	0.85 (0.67-1.08)
	4 / 5	-	0.84 (0.56-1.25)	-	-	-	-
	4	-	-	0.98 (0.71-1.35)	1.05 (0.80-1.38)	0.65 (0.48-0.87)	0.78 (0.61-1.01)
	5	-	-	1.07 (0.76-1.50)	0.84 (0.62-1.15)	0.76 (0.57-1.03)	0.87 (0.67-1.12)
Ethnicity	White	1.00	1.00	1.00	1.00	1.00	1.00
	Black / South Asian / Mixed / Other	1.76 (0.62-5.03)	-	1.79 (1.30-2.46)	1.37 (1.03-1.83)	-	0.92 (0.68-1.24)
	Black	-	0.97 (0.30-3.14)	-	-	0.84 (0.40-1.78)	-
	South Asian / Mixed / Other	-	1.22 (0.71-2.08)	-	-	1.15 (0.83-1.59)	-
BMI	<30 or missing	1.00	1.00	1.00	1.00	1.00	1.00
	40+ / 30-34.9 / 35-39.9	1.26 (0.63-2.54)	-	-	-	-	-
	40+	-	1.95 (1.28-2.96)	2.44 (1.82-3.28)	2.33 (1.84-2.96)	2.35 (1.85-2.99)	2.34 (1.88-2.92)
	30-34.9	-	1.23 (0.83-1.82)	1.34 (1.01-1.79)	1.23 (0.97-1.55)	1.32 (1.04-1.68)	1.24 (1.00-1.54)
	35-39.9	-	1.89 (1.23-2.92)	2.22 (1.64-3.00)	1.50 (1.14-1.99)	1.26 (0.93-1.71)	1.74 (1.37-2.21)
Learning disability		-	-	-	-	-	1.11 (0.70-1.75)
Serious mental illness		-	1.60 (0.88-2.91)	0.96 (0.53-1.71)	-	1.05 (0.65-1.69)	1.00 (0.66-1.52)
Morbidity count	0	1.00	1.00	1.00	1.00	1.00	1.00
	1 / 2+	1.59 (0.59-4.31)	-	1.81 (1.29-2.53)	-	-	-
	1	-	1.48 (0.89-2.44)	-	1.64 (1.22-2.20)	1.57 (1.16-2.13)	1.38 (1.06-1.81)
	2+	-	3.25 (1.92-5.51)	-	3.13 (2.30-4.24)	2.86 (2.07-3.95)	2.48 (1.87-3.28)
Flu vaccine		-	1.21 (0.84-1.73)	1.27 (0.96-1.66)	1.12 (0.89-1.42)	1.43 (1.11-1.85)	1.48 (1.18-1.85)
Number of SARS-CoV-2 tests	0	1.00	1.00	1.00	1.00	1.00	1.00
	1 / 2 / 3+	1.26 (0.59-2.66)	-	-	-	-	-
	1	-	1.08 (0.72-1.61)	1.70 (1.32-2.18)	1.30 (1.03-1.63)	1.56 (1.25-1.95)	1.25 (1.02-1.53)
	2	-	1.86 (1.13-3.06)	1.66 (1.13-2.42)	1.90 (1.41-2.54)	1.99 (1.47-2.69)	1.35 (1.00-1.83)
	3+	-	3.64 (2.41-5.48)	2.66 (1.90-3.71)	2.62 (2.02-3.42)	2.99 (2.28-3.93)	2.72 (2.14-3.46)

* Estimates correspond to a 1-year increase in age (or age-squared as indicated) within the given age categories.

Supplementary Table 35: Covariate hazard ratios for COVID-19 death in the BNT162b2 vs ChAdOx1 comparison and the 18-64 years and clinically vulnerable subgroup.

		Weeks since second dose					
**Variable**	**Category**	**3-6**	**7-10**	**11-14**	**15-18**	**19-22**	**23-26**
Age*	18-64	-	-	-	1.10 (0.83-1.46)	1.05 (0.79-1.39)	0.98 (0.78-1.24)
	18-64 squared	-	-	-	1.00 (1.00-1.00)	1.00 (1.00-1.00)	1.00 (1.00-1.00)
Sex	Female	-	-	-	-	1.00	1.00
	Male	-	-	-	-	1.46 (0.84-2.55)	1.36 (0.78-2.35)
IMD	1	-	-	-	1.00	1.00	1.00
	2 / 3 / 4 / 5	-	-	-	1.15 (0.56-2.35)	0.92 (0.47-1.79)	-
	2	-	-	-	-	-	1.01 (0.44-2.31)
	3 / 4 / 5	-	-	-	-	-	1.32 (0.64-2.73)
Ethnicity	White	-	-	-	1.00	1.00	1.00
	Black / South Asian / Mixed / Other	-	-	-	0.23 (0.03-1.91)	-	-
	Black	-	-	-	-	0.00 (0.00-0.00)	0.00 (0.00-0.00)
	South Asian	-	-	-	-	5.21 (2.28-11.90)	-
	Mixed	-	-	-	-	0.00 (0.00-0.00)	-
	Other	-	-	-	-	0.00 (0.00-0.00)	-
	South Asian / Mixed / Other	-	-	-	-	-	2.38 (0.95-5.95)
BMI	<30 or missing	-	-	-	1.00	1.00	1.00
	40+ / 30-34.9 / 35-39.9	-	-	-	3.66 (1.81-7.40)	1.31 (0.73-2.34)	-
	40+	-	-	-	-	-	4.97 (2.45-10.09)
	30-34.9 / 35-39.9	-	-	-	-	-	1.92 (1.01-3.64)
Morbidity count	0	-	-	-	1.00	1.00	1.00
	1 / 2+	-	-	-	2.29 (0.70-7.43)	10.54 (1.48-75.10)	-
	1	-	-	-	-	-	2.23 (0.75-6.62)
	2+	-	-	-	-	-	4.49 (1.47-13.73)
Number of SARS-CoV-2 tests	0	-	-	-	1.00	1.00	1.00
	1 / 2 / 3+	-	-	-	1.59 (0.81-3.11)	-	2.07 (1.21-3.54)
	1	-	-	-	-	1.25 (0.56-2.81)	-
	2 / 3+	-	-	-	-	3.65 (1.92-6.92)	-

* Estimates correspond to a 1-year increase in age (or age-squared as indicated) within the given age categories.

Supplementary Table 36: Covariate hazard ratios for Positive SARS-CoV-2 test in the BNT162b2 vs ChAdOx1 comparison and the 18-64 years and clinically vulnerable subgroup.

		Weeks since second dose					
**Variable**	**Category**	**3-6**	**7-10**	**11-14**	**15-18**	**19-22**	**23-26**
Age*	18-64	0.99 (0.96-1.02)	0.99 (0.97-1.00)	0.99 (0.97-1.00)	1.05 (1.04-1.06)	1.11 (1.10-1.13)	1.11 (1.10-1.12)
	18-64 squared	1.00 (1.00-1.00)	1.00 (1.00-1.00)	1.00 (1.00-1.00)	1.00 (1.00-1.00)	1.00 (1.00-1.00)	1.00 (1.00-1.00)
Sex	Female	1.00	1.00	1.00	1.00	1.00	1.00
	Male	0.93 (0.84-1.03)	1.04 (0.99-1.10)	1.01 (0.97-1.05)	0.93 (0.90-0.97)	0.87 (0.84-0.90)	0.90 (0.87-0.93)
IMD	1	1.00	1.00	1.00	1.00	1.00	1.00
	2	1.17 (1.00-1.35)	0.98 (0.91-1.05)	1.03 (0.97-1.09)	1.00 (0.95-1.06)	1.09 (1.03-1.15)	1.15 (1.10-1.21)
	3	1.15 (0.98-1.35)	0.97 (0.90-1.04)	0.99 (0.93-1.05)	1.00 (0.94-1.06)	1.16 (1.10-1.23)	1.21 (1.16-1.27)
	4	1.17 (0.99-1.37)	1.04 (0.96-1.12)	1.02 (0.96-1.09)	0.97 (0.92-1.03)	1.18 (1.12-1.25)	1.25 (1.19-1.31)
	5	1.32 (1.12-1.55)	1.11 (1.03-1.20)	0.98 (0.92-1.05)	0.96 (0.90-1.02)	1.26 (1.19-1.33)	1.30 (1.24-1.36)
Ethnicity	White	1.00	1.00	1.00	1.00	1.00	1.00
	Black / South Asian / Mixed / Other	1.00 (0.85-1.19)	-	-	-	-	-
	Black	-	0.67 (0.54-0.84)	0.79 (0.67-0.94)	0.80 (0.69-0.94)	0.75 (0.65-0.88)	0.61 (0.53-0.71)
	South Asian	-	0.74 (0.67-0.83)	0.96 (0.89-1.04)	0.91 (0.84-0.99)	0.86 (0.80-0.93)	0.84 (0.78-0.89)
	Mixed	-	1.08 (0.87-1.35)	0.82 (0.67-1.01)	0.90 (0.75-1.09)	0.78 (0.65-0.94)	0.76 (0.64-0.90)
	Other	-	0.60 (0.46-0.78)	0.85 (0.70-1.02)	0.82 (0.68-0.98)	0.66 (0.55-0.80)	0.76 (0.65-0.88)
BMI	<30 or missing	1.00	1.00	1.00	1.00	1.00	1.00
	40+	1.10 (0.94-1.28)	0.92 (0.85-1.00)	1.06 (0.99-1.12)	1.10 (1.03-1.16)	1.11 (1.05-1.17)	1.12 (1.07-1.17)
	30-34.9	1.02 (0.88-1.17)	1.11 (1.04-1.19)	1.12 (1.06-1.18)	1.08 (1.03-1.14)	1.11 (1.06-1.16)	1.13 (1.08-1.17)
	35-39.9	1.08 (0.91-1.29)	1.09 (1.00-1.18)	1.08 (1.01-1.15)	1.22 (1.15-1.30)	1.15 (1.08-1.22)	1.13 (1.07-1.19)
Learning disability		-	0.34 (0.28-0.41)	0.41 (0.36-0.47)	0.53 (0.47-0.60)	0.51 (0.45-0.58)	0.61 (0.55-0.67)
Serious mental illness		0.66 (0.50-0.87)	0.62 (0.54-0.71)	0.59 (0.52-0.66)	0.54 (0.48-0.61)	0.52 (0.47-0.58)	0.52 (0.47-0.57)
Morbidity count	0	1.00	1.00	1.00	1.00	1.00	1.00
	1	1.14 (0.98-1.33)	0.98 (0.90-1.06)	0.96 (0.90-1.02)	0.96 (0.91-1.02)	0.96 (0.91-1.01)	0.92 (0.88-0.96)
	2+	0.95 (0.77-1.17)	0.87 (0.79-0.97)	0.94 (0.87-1.01)	0.96 (0.89-1.03)	0.90 (0.85-0.97)	0.86 (0.81-0.91)
Flu vaccine		0.99 (0.89-1.11)	0.98 (0.93-1.03)	1.01 (0.97-1.06)	1.06 (1.02-1.11)	1.10 (1.06-1.15)	1.08 (1.04-1.11)
Number of SARS-CoV-2 tests	0	1.00	1.00	1.00	1.00	1.00	1.00
	1	1.47 (1.31-1.66)	1.49 (1.41-1.58)	1.32 (1.26-1.39)	1.33 (1.27-1.39)	1.26 (1.21-1.32)	1.28 (1.23-1.33)
	2	1.63 (1.36-1.94)	1.44 (1.31-1.57)	1.44 (1.34-1.55)	1.40 (1.31-1.50)	1.37 (1.28-1.45)	1.35 (1.28-1.43)
	3+	1.69 (1.38-2.07)	1.64 (1.49-1.81)	1.53 (1.42-1.66)	1.54 (1.44-1.66)	1.45 (1.35-1.55)	1.38 (1.30-1.47)
Pregnancy		-	0.66 (0.45-0.98)	0.81 (0.59-1.12)	0.99 (0.75-1.32)	0.95 (0.73-1.25)	0.85 (0.65-1.10)

* Estimates correspond to a 1-year increase in age (or age-squared as indicated) within the given age categories.

Supplementary Table 37: Covariate hazard ratios for Non-COVID-19 death in the BNT162b2 vs ChAdOx1 comparison and the 18-64 years and clinically vulnerable subgroup.

		Weeks since second dose					
**Variable**	**Category**	**3-6**	**7-10**	**11-14**	**15-18**	**19-22**	**23-26**
Age*	18-64	1.05 (0.94-1.19)	1.07 (0.94-1.22)	1.08 (0.97-1.20)	1.13 (1.01-1.26)	1.09 (0.98-1.21)	1.19 (1.07-1.34)
	18-64 squared	1.00 (1.00-1.00)	1.00 (1.00-1.00)	1.00 (1.00-1.00)	1.00 (1.00-1.00)	1.00 (1.00-1.00)	1.00 (1.00-1.00)
Sex	Female	1.00	1.00	1.00	1.00	1.00	1.00
	Male	1.95 (1.53-2.47)	1.33 (1.08-1.64)	1.37 (1.13-1.68)	1.41 (1.16-1.70)	1.57 (1.29-1.90)	1.41 (1.17-1.70)
IMD	1	1.00	1.00	1.00	1.00	1.00	1.00
	2	0.99 (0.72-1.37)	0.86 (0.64-1.15)	0.98 (0.74-1.30)	0.92 (0.69-1.21)	0.72 (0.54-0.95)	0.86 (0.66-1.11)
	3	0.84 (0.59-1.19)	0.68 (0.49-0.94)	0.86 (0.63-1.16)	0.90 (0.67-1.19)	0.71 (0.53-0.95)	0.71 (0.54-0.94)
	4	0.84 (0.59-1.21)	0.77 (0.56-1.06)	0.79 (0.58-1.09)	0.85 (0.63-1.14)	0.66 (0.49-0.90)	0.72 (0.54-0.95)
	5	0.75 (0.50-1.11)	0.65 (0.46-0.92)	0.74 (0.54-1.03)	0.72 (0.52-0.99)	0.78 (0.58-1.06)	0.64 (0.48-0.87)
Ethnicity	White	1.00	1.00	1.00	1.00	1.00	1.00
	Black / South Asian / Mixed / Other	0.38 (0.21-0.72)	0.60 (0.39-0.94)	-	-	-	0.67 (0.46-0.99)
	Black	-	-	0.71 (0.29-1.70)	1.22 (0.63-2.39)	1.19 (0.60-2.36)	-
	South Asian / Mixed / Other	-	-	0.52 (0.31-0.87)	-	-	-
	South Asian	-	-	-	0.73 (0.44-1.20)	0.61 (0.36-1.02)	-
	Mixed / Other	-	-	-	0.75 (0.33-1.70)	0.71 (0.31-1.63)	-
BMI	<30 or missing	1.00	1.00	1.00	1.00	1.00	1.00
	40+	1.15 (0.80-1.63)	0.60 (0.41-0.90)	0.92 (0.67-1.27)	0.80 (0.58-1.11)	0.80 (0.58-1.10)	0.93 (0.70-1.25)
	30-34.9	0.92 (0.69-1.22)	0.70 (0.53-0.92)	0.83 (0.64-1.07)	0.67 (0.52-0.87)	0.57 (0.43-0.76)	0.69 (0.54-0.88)
	35-39.9	0.86 (0.59-1.27)	0.85 (0.61-1.19)	0.80 (0.57-1.12)	0.60 (0.42-0.85)	0.92 (0.68-1.25)	0.65 (0.47-0.91)
Learning disability		1.06 (0.52-2.17)	-	1.36 (0.76-2.43)	2.08 (1.29-3.35)	1.46 (0.83-2.55)	1.60 (0.92-2.79)
Serious mental illness		2.04 (1.30-3.18)	1.95 (1.28-2.98)	0.87 (0.50-1.53)	1.85 (1.25-2.72)	1.34 (0.86-2.09)	1.15 (0.72-1.83)
Morbidity count	0	1.00	1.00	1.00	1.00	1.00	1.00
	1	0.98 (0.67-1.42)	1.18 (0.82-1.70)	1.02 (0.74-1.40)	0.88 (0.64-1.21)	0.73 (0.54-0.99)	1.30 (0.93-1.83)
	2+	2.18 (1.49-3.19)	2.46 (1.69-3.57)	1.74 (1.25-2.41)	1.94 (1.42-2.65)	1.46 (1.07-1.98)	2.47 (1.76-3.48)
Flu vaccine		0.74 (0.56-0.98)	0.86 (0.66-1.11)	0.94 (0.73-1.22)	0.83 (0.66-1.06)	1.07 (0.82-1.40)	1.10 (0.86-1.41)
Number of SARS-CoV-2 tests	0	1.00	1.00	1.00	1.00	1.00	1.00
	1	1.27 (0.94-1.74)	0.96 (0.71-1.30)	1.32 (1.02-1.71)	1.02 (0.77-1.35)	1.00 (0.76-1.32)	1.08 (0.84-1.40)
	2	1.94 (1.30-2.91)	1.58 (1.08-2.31)	1.24 (0.82-1.88)	1.88 (1.34-2.63)	2.07 (1.50-2.84)	1.32 (0.92-1.91)
	3+	5.37 (4.05-7.11)	4.23 (3.25-5.51)	4.71 (3.67-6.05)	5.00 (3.95-6.32)	3.68 (2.84-4.75)	4.36 (3.45-5.50)

* Estimates correspond to a 1-year increase in age (or age-squared as indicated) within the given age categories.

Supplementary Table 38: Covariate hazard ratios for Any SARS-CoV-2 test in the BNT162b2 vs ChAdOx1 comparison and the 18-64 years and clinically vulnerable subgroup.

		**Weeks since second dose**					
**Variable**	**Category**	**3-6**	**7-10**	**11-14**	**15-18**	**19-22**	**23-26**
Age*	18-64	1.04 (1.03-1.04)	1.04 (1.04-1.04)	1.03 (1.02-1.03)	1.03 (1.03-1.04)	1.05 (1.05-1.05)	1.05 (1.05-1.06)
	18-64 squared	1.00 (1.00-1.00)	1.00 (1.00-1.00)	1.00 (1.00-1.00)	1.00 (1.00-1.00)	1.00 (1.00-1.00)	1.00 (1.00-1.00)
Sex	Female	1.00	1.00	1.00	1.00	1.00	1.00
	Male	0.71 (0.70-0.72)	0.75 (0.74-0.76)	0.81 (0.80-0.82)	0.79 (0.78-0.80)	0.78 (0.77-0.79)	0.78 (0.77-0.79)
IMD	1	1.00	1.00	1.00	1.00	1.00	1.00
	2	1.17 (1.15-1.19)	1.15 (1.14-1.17)	1.15 (1.13-1.16)	1.14 (1.12-1.16)	1.16 (1.14-1.18)	1.19 (1.17-1.21)
	3	1.26 (1.24-1.28)	1.25 (1.23-1.27)	1.23 (1.21-1.25)	1.26 (1.24-1.28)	1.28 (1.26-1.30)	1.32 (1.30-1.34)
	4	1.34 (1.32-1.37)	1.34 (1.32-1.36)	1.31 (1.29-1.34)	1.35 (1.32-1.37)	1.41 (1.39-1.43)	1.43 (1.41-1.45)
	5	1.46 (1.44-1.49)	1.49 (1.46-1.51)	1.44 (1.42-1.47)	1.48 (1.45-1.50)	1.57 (1.54-1.59)	1.61 (1.58-1.63)
Ethnicity	White	1.00	1.00	1.00	1.00	1.00	1.00
	Black	0.92 (0.88-0.96)	0.86 (0.82-0.89)	0.92 (0.88-0.96)	0.92 (0.89-0.96)	0.88 (0.84-0.91)	0.85 (0.82-0.88)
	South Asian	0.58 (0.56-0.60)	0.53 (0.52-0.55)	0.58 (0.57-0.60)	0.59 (0.58-0.61)	0.63 (0.61-0.64)	0.65 (0.63-0.66)
	Mixed	0.88 (0.84-0.93)	0.85 (0.81-0.89)	0.90 (0.86-0.95)	0.88 (0.84-0.93)	0.86 (0.82-0.90)	0.87 (0.83-0.91)
	Other	0.71 (0.67-0.75)	0.68 (0.65-0.72)	0.74 (0.70-0.78)	0.76 (0.73-0.80)	0.75 (0.72-0.79)	0.76 (0.73-0.80)
BMI	<30 or missing	1.00	1.00	1.00	1.00	1.00	1.00
	40+	0.92 (0.90-0.93)	0.92 (0.91-0.94)	0.93 (0.91-0.94)	0.92 (0.90-0.93)	0.92 (0.91-0.94)	0.93 (0.92-0.94)
	30-34.9	0.98 (0.96-0.99)	0.98 (0.97-1.00)	0.98 (0.96-0.99)	0.96 (0.95-0.97)	0.98 (0.97-0.99)	0.98 (0.97-0.99)
	35-39.9	0.96 (0.94-0.97)	0.97 (0.95-0.99)	0.94 (0.92-0.96)	0.94 (0.93-0.96)	0.96 (0.95-0.98)	0.95 (0.94-0.97)
Learning disability		1.06 (1.03-1.09)	0.94 (0.91-0.96)	1.00 (0.97-1.03)	1.05 (1.02-1.07)	0.97 (0.95-1.00)	1.00 (0.97-1.03)
Serious mental illness		0.71 (0.69-0.73)	0.71 (0.69-0.73)	0.76 (0.74-0.78)	0.73 (0.71-0.75)	0.71 (0.69-0.73)	0.71 (0.69-0.73)
Morbidity count	0	1.00	1.00	1.00	1.00	1.00	1.00
	1	0.94 (0.93-0.96)	0.94 (0.92-0.95)	0.95 (0.93-0.96)	0.97 (0.95-0.98)	0.96 (0.95-0.97)	0.95 (0.93-0.96)
	2+	0.87 (0.85-0.88)	0.86 (0.84-0.87)	0.89 (0.87-0.90)	0.90 (0.88-0.91)	0.88 (0.86-0.89)	0.87 (0.86-0.89)
Flu vaccine		1.24 (1.22-1.25)	1.22 (1.20-1.23)	1.20 (1.19-1.22)	1.19 (1.18-1.21)	1.20 (1.18-1.21)	1.21 (1.20-1.23)
Number of SARS-CoV-2 tests	0	1.00	1.00	1.00	1.00	1.00	1.00
	1	1.42 (1.40-1.44)	1.44 (1.42-1.45)	1.39 (1.37-1.41)	1.37 (1.35-1.39)	1.36 (1.34-1.37)	1.34 (1.32-1.36)
	2	1.75 (1.72-1.79)	1.72 (1.69-1.75)	1.68 (1.65-1.71)	1.67 (1.64-1.70)	1.62 (1.59-1.65)	1.60 (1.57-1.63)
	3+	2.76 (2.71-2.81)	2.59 (2.55-2.64)	2.50 (2.46-2.55)	2.43 (2.39-2.47)	2.28 (2.24-2.32)	2.20 (2.16-2.24)
Pregnancy		0.67 (0.60-0.74)	0.78 (0.71-0.85)	0.78 (0.71-0.85)	0.78 (0.71-0.86)	0.78 (0.71-0.85)	0.94 (0.87-1.03)

* Estimates correspond to a 1-year increase in age (or age-squared as indicated) within the given age categories.

Supplementary Table 39: Covariate hazard ratios for Positive SARS-CoV-2 test in the BNT162b2 vs unvaccinated comparison and the 40-64 years subgroup.

		Weeks since second dose					
**Variable**	**Category**	**3-6**	**7-10**	**11-14**	**15-18**	**19-22**	**23-26**
Age*	40-45	0.97 (0.95-1.00)	1.00 (0.97-1.02)	0.98 (0.95-1.00)	0.99 (0.97-1.01)	0.98 (0.95-1.00)	0.94 (0.90-0.98)
	45-50	0.98 (0.95-1.01)	0.98 (0.95-1.01)	0.99 (0.95-1.02)	0.99 (0.96-1.02)	0.97 (0.95-1.00)	1.00 (0.96-1.04)
	50-55	0.93 (0.88-0.97)	0.97 (0.93-1.01)	0.96 (0.92-1.00)	1.00 (0.96-1.05)	0.96 (0.92-1.00)	0.97 (0.93-1.01)
	55-60	1.03 (0.96-1.10)	1.03 (0.98-1.09)	0.91 (0.86-0.96)	0.99 (0.93-1.04)	0.96 (0.91-1.02)	0.98 (0.93-1.02)
	60-64	0.89 (0.77-1.02)	0.93 (0.83-1.03)	1.00 (0.90-1.10)	1.00 (0.89-1.12)	0.90 (0.81-0.99)	0.92 (0.84-1.01)
Sex	Female	1.00	1.00	1.00	1.00	1.00	1.00
	Male	0.77 (0.73-0.81)	0.75 (0.71-0.78)	0.72 (0.69-0.76)	0.71 (0.68-0.75)	0.74 (0.71-0.78)	0.77 (0.72-0.81)
IMD	1	1.00	1.00	1.00	1.00	1.00	1.00
	2	1.00 (0.93-1.07)	1.09 (1.01-1.17)	1.07 (1.00-1.16)	1.13 (1.05-1.22)	1.12 (1.04-1.21)	1.16 (1.06-1.27)
	3	0.97 (0.90-1.05)	1.05 (0.97-1.13)	1.06 (0.98-1.14)	1.22 (1.14-1.32)	1.20 (1.12-1.30)	1.23 (1.12-1.34)
	4	0.99 (0.91-1.08)	1.10 (1.02-1.20)	1.11 (1.03-1.21)	1.26 (1.16-1.36)	1.22 (1.13-1.32)	1.35 (1.23-1.48)
	5	1.04 (0.95-1.13)	1.07 (0.98-1.17)	1.15 (1.06-1.26)	1.29 (1.19-1.40)	1.27 (1.17-1.37)	1.37 (1.24-1.51)
Ethnicity	White	1.00	1.00	1.00	1.00	1.00	1.00
	Black	0.87 (0.78-0.97)	0.95 (0.86-1.06)	0.90 (0.81-1.01)	0.72 (0.64-0.81)	0.66 (0.59-0.75)	0.80 (0.70-0.92)
	South Asian	0.63 (0.57-0.69)	0.65 (0.59-0.71)	0.65 (0.59-0.72)	0.54 (0.49-0.60)	0.56 (0.51-0.62)	0.48 (0.42-0.55)
	Mixed	0.83 (0.71-0.98)	-	0.72 (0.60-0.86)	0.79 (0.67-0.93)	0.88 (0.75-1.02)	-
	Other	0.55 (0.47-0.64)	-	0.56 (0.48-0.65)	0.56 (0.48-0.65)	0.55 (0.48-0.63)	-
	Mixed / Other	-	0.75 (0.68-0.84)	-	-	-	0.67 (0.59-0.77)
BMI	<30 or missing	1.00	1.00	1.00	1.00	1.00	1.00
	40+	1.62 (1.05-2.49)	-	1.64 (1.04-2.58)	1.45 (0.90-2.35)	1.57 (1.01-2.44)	-
	30-34.9	1.38 (1.28-1.50)	-	1.40 (1.29-1.51)	1.41 (1.31-1.53)	1.25 (1.16-1.36)	-
	35-39.9	1.38 (1.22-1.57)	-	1.38 (1.22-1.57)	1.34 (1.18-1.51)	1.20 (1.06-1.36)	-
	40+ / 30-34.9 / 35-39.9	-	1.44 (1.35-1.54)	-	-	-	1.35 (1.25-1.47)
Morbidity count	0	1.00	1.00	1.00	1.00	1.00	1.00
	1 / 2+	1.38 (1.07-1.78)	1.04 (0.78-1.40)	1.14 (0.86-1.53)	-	-	1.12 (0.80-1.57)
	1	-	-	-	0.94 (0.68-1.28)	1.09 (0.81-1.46)	-
	2+	-	-	-	0.00 (0.00-0.00)	0.00 (0.00-0.00)	-
Flu vaccine		1.09 (0.99-1.20)	1.03 (0.94-1.14)	0.99 (0.90-1.09)	1.07 (0.98-1.17)	1.01 (0.92-1.10)	1.03 (0.92-1.15)
Number of SARS-CoV-2 tests	0	1.00	1.00	1.00	1.00	1.00	1.00
	1	2.13 (1.99-2.29)	2.17 (2.03-2.33)	2.04 (1.91-2.19)	2.03 (1.90-2.17)	1.98 (1.85-2.11)	2.21 (2.04-2.39)
	2	2.73 (2.47-3.02)	2.21 (1.97-2.47)	2.19 (1.96-2.44)	2.29 (2.07-2.54)	2.23 (2.01-2.47)	2.49 (2.19-2.83)
	3+	2.74 (2.49-3.02)	2.64 (2.40-2.90)	2.74 (2.50-3.01)	2.59 (2.36-2.85)	2.44 (2.22-2.68)	2.55 (2.25-2.88)
Pregnancy		-	-	1.11 (0.78-1.57)	1.01 (0.72-1.44)	1.19 (0.85-1.66)	-

* Estimates correspond to a 1-year increase in age (or age-squared as indicated) within the given age categories.

Supplementary Table 40: Covariate hazard ratios for Any SARS-CoV-2 test in the BNT162b2 vs unvaccinated comparison and the 40-64 years subgroup.

		Weeks since second dose					
**Variable**	**Category**	**3-6**	**7-10**	**11-14**	**15-18**	**19-22**	**23-26**
Age*	40-45	0.99 (0.98-1.00)	1.01 (1.00-1.02)	0.99 (0.98-1.00)	1.00 (0.99-1.01)	0.99 (0.98-1.00)	1.00 (0.98-1.02)
	45-50	1.00 (0.98-1.01)	1.00 (0.99-1.02)	0.99 (0.97-1.00)	1.01 (0.99-1.02)	1.00 (0.98-1.01)	1.00 (0.99-1.02)
	50-55	0.98 (0.96-0.99)	0.99 (0.97-1.00)	0.99 (0.97-1.01)	1.00 (0.98-1.01)	0.99 (0.98-1.01)	1.00 (0.98-1.02)
	55-60	0.98 (0.96-1.00)	1.01 (0.99-1.03)	0.96 (0.95-0.98)	0.99 (0.97-1.01)	0.98 (0.96-1.00)	0.99 (0.97-1.01)
	60-64	0.97 (0.94-1.00)	0.94 (0.91-0.98)	0.98 (0.95-1.02)	0.97 (0.93-1.00)	0.97 (0.94-1.00)	0.94 (0.91-0.98)
Sex	Female	1.00	1.00	1.00	1.00	1.00	1.00
	Male	0.74 (0.73-0.75)	0.71 (0.69-0.72)	0.70 (0.68-0.71)	0.69 (0.68-0.70)	0.71 (0.70-0.73)	0.70 (0.69-0.72)
IMD	1	1.00	1.00	1.00	1.00	1.00	1.00
	2	1.07 (1.04-1.10)	1.13 (1.10-1.16)	1.13 (1.09-1.16)	1.16 (1.13-1.20)	1.16 (1.12-1.19)	1.18 (1.13-1.22)
	3	1.13 (1.09-1.16)	1.19 (1.16-1.23)	1.19 (1.15-1.23)	1.28 (1.24-1.33)	1.29 (1.25-1.33)	1.29 (1.24-1.34)
	4	1.20 (1.16-1.23)	1.26 (1.22-1.30)	1.31 (1.27-1.35)	1.35 (1.31-1.40)	1.41 (1.36-1.46)	1.45 (1.40-1.51)
	5	1.26 (1.22-1.30)	1.35 (1.30-1.39)	1.39 (1.34-1.43)	1.53 (1.48-1.58)	1.56 (1.51-1.62)	1.62 (1.55-1.69)
Ethnicity	White	1.00	1.00	1.00	1.00	1.00	1.00
	Black	0.91 (0.87-0.95)	0.90 (0.86-0.94)	0.92 (0.88-0.96)	0.90 (0.86-0.94)	0.88 (0.84-0.92)	0.91 (0.86-0.97)
	South Asian	0.58 (0.55-0.60)	0.57 (0.55-0.59)	0.57 (0.55-0.60)	0.57 (0.54-0.59)	0.57 (0.54-0.59)	0.54 (0.51-0.58)
	Mixed	0.84 (0.79-0.89)	0.90 (0.84-0.95)	0.83 (0.78-0.88)	0.82 (0.77-0.88)	0.84 (0.79-0.90)	0.82 (0.76-0.90)
	Other	0.70 (0.66-0.73)	0.64 (0.60-0.67)	0.64 (0.61-0.68)	0.61 (0.57-0.64)	0.62 (0.59-0.66)	0.53 (0.49-0.58)
BMI	<30 or missing	1.00	1.00	1.00	1.00	1.00	1.00
	40+	1.36 (1.13-1.64)	1.02 (0.83-1.26)	1.35 (1.12-1.64)	1.30 (1.06-1.60)	1.36 (1.11-1.66)	1.23 (0.94-1.62)
	30-34.9	1.18 (1.15-1.22)	1.21 (1.17-1.24)	1.19 (1.15-1.23)	1.21 (1.17-1.24)	1.15 (1.11-1.19)	1.16 (1.11-1.21)
	35-39.9	1.12 (1.06-1.17)	1.11 (1.05-1.17)	1.13 (1.07-1.19)	1.11 (1.05-1.17)	1.15 (1.09-1.22)	1.13 (1.06-1.21)
Serious mental illness		0.90 (0.76-1.07)	0.91 (0.77-1.07)	0.97 (0.82-1.14)	0.68 (0.56-0.83)	0.94 (0.78-1.13)	0.76 (0.60-0.96)
Morbidity count	0	1.00	1.00	1.00	1.00	1.00	1.00
	1 / 2+	1.26 (1.14-1.39)	-	1.12 (1.00-1.25)	1.13 (1.00-1.26)	1.07 (0.95-1.21)	1.10 (0.96-1.27)
	1	-	1.19 (1.07-1.33)	-	-	-	-
	2+	-	2.96 (1.72-5.09)	-	-	-	-
Flu vaccine		1.26 (1.22-1.30)	1.20 (1.16-1.23)	1.16 (1.12-1.20)	1.17 (1.13-1.21)	1.23 (1.19-1.27)	1.25 (1.20-1.30)
Number of SARS-CoV-2 tests	0	1.00	1.00	1.00	1.00	1.00	1.00
	1	2.14 (2.08-2.20)	2.10 (2.05-2.16)	2.03 (1.98-2.09)	1.98 (1.93-2.04)	1.98 (1.92-2.03)	2.06 (1.99-2.14)
	2	2.72 (2.62-2.83)	2.44 (2.34-2.54)	2.41 (2.31-2.51)	2.42 (2.32-2.52)	2.40 (2.30-2.51)	2.53 (2.39-2.67)
	3+	5.82 (5.65-6.01)	5.13 (4.97-5.29)	4.70 (4.55-4.86)	4.28 (4.13-4.43)	3.93 (3.79-4.08)	4.41 (4.22-4.62)
Pregnancy		1.84 (1.66-2.04)	1.59 (1.42-1.78)	1.46 (1.29-1.65)	1.36 (1.18-1.56)	1.29 (1.12-1.49)	0.93 (0.72-1.22)

* Estimates correspond to a 1-year increase in age (or age-squared as indicated) within the given age categories.

Supplementary Table 41: Covariate hazard ratios for COVID-19 hospitalisation in the ChAdOx1 vs unvaccinated comparison and the 40-64 years subgroup.

		Weeks since second dose					
**Variable**	**Category**	**3-6**	**7-10**	**11-14**	**15-18**	**19-22**	**23-26**
Age*	40-45	1.01 (0.91-1.11)	0.99 (0.90-1.10)	1.03 (0.92-1.14)	1.03 (0.93-1.15)	1.14 (1.02-1.28)	1.05 (0.87-1.27)
	45-50	1.06 (0.95-1.17)	1.01 (0.89-1.15)	0.97 (0.86-1.09)	1.02 (0.91-1.14)	1.04 (0.93-1.16)	1.12 (0.97-1.30)
	50-55	1.10 (0.96-1.26)	1.05 (0.94-1.16)	0.98 (0.88-1.09)	1.08 (0.96-1.22)	1.08 (0.97-1.20)	0.98 (0.89-1.09)
	55-60	1.01 (0.86-1.19)	1.09 (0.97-1.21)	0.90 (0.80-1.00)	0.98 (0.87-1.11)	1.09 (0.97-1.21)	1.06 (0.96-1.17)
	60-64	1.17 (0.89-1.53)	0.95 (0.78-1.16)	1.05 (0.90-1.24)	0.97 (0.81-1.17)	0.87 (0.71-1.06)	0.94 (0.79-1.11)
Sex	Female	1.00	1.00	1.00	1.00	1.00	1.00
	Male	1.21 (1.02-1.43)	1.21 (1.04-1.42)	1.29 (1.11-1.51)	1.44 (1.23-1.69)	1.38 (1.18-1.60)	1.46 (1.24-1.72)
IMD	1	1.00	1.00	1.00	1.00	1.00	1.00
	2	0.81 (0.65-1.02)	0.98 (0.79-1.22)	0.97 (0.78-1.20)	0.97 (0.78-1.20)	0.96 (0.77-1.20)	1.15 (0.90-1.47)
	3	0.79 (0.62-1.01)	0.89 (0.70-1.13)	0.78 (0.62-0.99)	0.90 (0.71-1.14)	0.95 (0.75-1.19)	0.94 (0.72-1.22)
	4	0.83 (0.64-1.08)	0.84 (0.65-1.09)	0.86 (0.67-1.10)	0.89 (0.69-1.13)	0.91 (0.72-1.16)	1.10 (0.85-1.43)
	5	0.87 (0.66-1.15)	0.95 (0.73-1.24)	0.84 (0.64-1.10)	0.75 (0.57-0.99)	0.91 (0.71-1.18)	1.21 (0.93-1.58)
Ethnicity	White	1.00	1.00	1.00	1.00	1.00	1.00
	Black / South Asian / Mixed / Other	1.12 (0.92-1.36)	-	1.46 (1.21-1.76)	1.00 (0.82-1.23)	0.97 (0.79-1.18)	1.18 (0.95-1.48)
	Black	-	1.53 (1.16-2.01)	-	-	-	-
	South Asian / Mixed / Other	-	1.15 (0.93-1.42)	-	-	-	-
BMI	<30 or missing	1.00	1.00	1.00	1.00	1.00	1.00
	40+ / 30-34.9 / 35-39.9	2.84 (2.36-3.43)	2.50 (2.10-2.97)	2.49 (2.09-2.97)	2.75 (2.32-3.26)	2.73 (2.31-3.23)	2.29 (1.91-2.74)
Morbidity count	0	1.00	1.00	1.00	1.00	1.00	1.00
	1 / 2+	1.80 (0.92-3.52)	-	2.09 (1.15-3.80)	1.65 (0.85-3.20)	1.11 (0.50-2.49)	2.24 (1.23-4.07)
	1	-	2.02 (1.11-3.69)	-	-	-	-
	2+	-	0.00 (0.00-0.00)	-	-	-	-
Flu vaccine		1.19 (0.89-1.58)	1.07 (0.83-1.39)	1.00 (0.78-1.30)	1.52 (1.20-1.92)	0.96 (0.76-1.21)	1.48 (1.20-1.82)
Number of SARS-CoV-2 tests	0	1.00	1.00	1.00	1.00	1.00	1.00
	1	1.73 (1.38-2.18)	1.54 (1.23-1.92)	1.57 (1.25-1.96)	1.55 (1.25-1.93)	1.32 (1.05-1.64)	1.43 (1.15-1.79)
	2	2.09 (1.47-2.97)	1.54 (1.05-2.24)	1.76 (1.22-2.53)	1.64 (1.14-2.34)	1.14 (0.76-1.70)	1.60 (1.11-2.31)
	3+	2.21 (1.60-3.06)	1.94 (1.42-2.65)	1.87 (1.35-2.60)	1.88 (1.35-2.62)	1.44 (1.02-2.04)	1.58 (1.10-2.25)

* Estimates correspond to a 1-year increase in age (or age-squared as indicated) within the given age categories.

Supplementary Table 42: Covariate hazard ratios for COVID-19 death in the ChAdOx1 vs unvaccinated comparison and the 40-64 years subgroup.

		Weeks since second dose					
**Variable**	**Category**	**3-6**	**7-10**	**11-14**	**15-18**	**19-22**	**23-26**
Age*	40-45	-	-	2.48 (1.77-3.46)	1.22 (0.74-2.02)	0.95 (0.40-2.26)	3.09 (2.98-3.20)
	45-50	-	-	1.27 (0.99-1.63)	0.94 (0.92-0.96)	0.78 (0.53-1.15)	1.17 (0.94-1.45)
	50-55	-	-	1.04 (0.52-2.09)	1.06 (0.58-1.93)	0.78 (0.52-1.15)	1.13 (0.79-1.61)
	55-60	-	-	0.98 (0.59-1.64)	1.28 (0.83-1.97)	1.27 (0.99-1.62)	0.88 (0.60-1.29)
	60-64	-	-	1.03 (0.68-1.56)	1.93 (1.00-3.72)	1.08 (0.61-1.91)	1.04 (0.60-1.81)
Sex	Female	-	-	-	-	1.00	1.00
	Male	-	-	-	-	3.05 (1.52-6.11)	2.97 (1.44-6.15)
IMD	1	-	-	1.00	1.00	1.00	1.00
	2 / 3 / 4 / 5	-	-	0.70 (0.35-1.41)	0.78 (0.35-1.73)	0.85 (0.46-1.56)	-
	2	-	-	-	-	-	1.27 (0.49-3.27)
	3 / 4 / 5	-	-	-	-	-	0.82 (0.33-2.06)
Ethnicity	White	-	-	1.00	1.00	1.00	1.00
	Black	-	-	0.00 (0.00-0.00)	-	2.48 (1.00-6.13)	-
	South Asian / Mixed / Other	-	-	0.96 (0.35-2.59)	-	0.87 (0.34-2.24)	-
	Black / South Asian / Mixed / Other	-	-	-	1.63 (0.64-4.15)	-	1.16 (0.46-2.93)
BMI	<30 or missing	-	-	1.00	1.00	1.00	1.00
	40+ / 30-34.9 / 35-39.9	-	-	2.26 (1.07-4.77)	2.88 (1.38-6.00)	4.38 (2.46-7.80)	-
	40+	-	-	-	-	-	0.00 (0.00-0.00)
	30-34.9 / 35-39.9	-	-	-	-	-	2.64 (1.35-5.18)
Morbidity count	0	-	-	1.00	1.00	1.00	1.00
	1 / 2+	-	-	3.00 (0.42-21.15)	-	2.61 (0.36-18.98)	3.14 (0.44-22.61)
	1	-	-	-	0.00 (0.00-0.00)	-	-
	2+	-	-	-	0.00 (0.00-0.00)	-	-
Flu vaccine		-	-	0.96 (0.34-2.71)	-	-	-
Number of SARS-CoV-2 tests	0	-	-	1.00	1.00	1.00	1.00
	1 / 2 / 3+	-	-	2.63 (1.33-5.20)	2.30 (1.09-4.86)	0.62 (0.25-1.57)	1.20 (0.52-2.79)

* Estimates correspond to a 1-year increase in age (or age-squared as indicated) within the given age categories.

Supplementary Table 43: Covariate hazard ratios for Positive SARS-CoV-2 test in the ChAdOx1 vs unvaccinated comparison and the 40-64 years subgroup.

		Weeks since second dose					
**Variable**	**Category**	**3-6**	**7-10**	**11-14**	**15-18**	**19-22**	**23-26**
Age*	40-45	0.97 (0.96-0.99)	1.00 (0.98-1.02)	1.00 (0.99-1.02)	1.00 (0.98-1.01)	0.97 (0.96-0.99)	0.98 (0.96-1.00)
	45-50	0.97 (0.95-0.99)	0.98 (0.96-1.00)	0.97 (0.95-0.99)	0.95 (0.94-0.97)	0.96 (0.94-0.97)	0.95 (0.93-0.97)
	50-55	0.94 (0.92-0.97)	0.96 (0.94-0.98)	0.96 (0.94-0.97)	0.93 (0.92-0.95)	0.93 (0.92-0.95)	0.96 (0.95-0.97)
	55-60	0.94 (0.91-0.97)	0.94 (0.92-0.96)	0.94 (0.92-0.96)	0.96 (0.94-0.98)	0.96 (0.94-0.97)	0.97 (0.96-0.99)
	60-64	0.89 (0.84-0.95)	0.96 (0.92-1.00)	0.97 (0.94-1.00)	0.92 (0.89-0.96)	0.95 (0.92-0.98)	0.94 (0.92-0.97)
Sex	Female	1.00	1.00	1.00	1.00	1.00	1.00
	Male	0.91 (0.88-0.94)	0.94 (0.91-0.96)	0.87 (0.85-0.90)	0.86 (0.84-0.89)	0.92 (0.90-0.94)	0.99 (0.97-1.01)
IMD	1	1.00	1.00	1.00	1.00	1.00	1.00
	2	0.98 (0.93-1.04)	1.03 (0.98-1.07)	1.10 (1.05-1.15)	1.12 (1.07-1.17)	1.12 (1.08-1.17)	1.16 (1.11-1.21)
	3	0.97 (0.92-1.02)	1.02 (0.97-1.07)	1.08 (1.03-1.13)	1.19 (1.14-1.24)	1.22 (1.17-1.27)	1.18 (1.14-1.23)
	4	1.00 (0.95-1.05)	1.00 (0.96-1.05)	1.12 (1.07-1.17)	1.24 (1.19-1.30)	1.28 (1.24-1.33)	1.30 (1.25-1.36)
	5	1.09 (1.03-1.15)	1.03 (0.98-1.08)	1.16 (1.11-1.22)	1.30 (1.24-1.35)	1.38 (1.33-1.44)	1.40 (1.34-1.46)
Ethnicity	White	1.00	1.00	1.00	1.00	1.00	1.00
	Black	0.84 (0.76-0.92)	0.91 (0.83-0.99)	0.85 (0.77-0.93)	0.70 (0.64-0.77)	0.63 (0.58-0.69)	0.71 (0.65-0.78)
	South Asian	0.67 (0.62-0.72)	0.73 (0.68-0.78)	0.73 (0.68-0.78)	0.68 (0.64-0.73)	0.63 (0.59-0.66)	0.54 (0.50-0.58)
	Mixed	0.80 (0.70-0.92)	0.95 (0.85-1.07)	0.83 (0.73-0.93)	0.81 (0.72-0.91)	0.80 (0.72-0.89)	0.82 (0.73-0.92)
	Other	0.58 (0.51-0.65)	0.62 (0.56-0.69)	0.59 (0.53-0.66)	0.64 (0.58-0.70)	0.58 (0.53-0.64)	0.55 (0.50-0.61)
BMI	<30 or missing	1.00	1.00	1.00	1.00	1.00	1.00
	40+	1.34 (0.98-1.83)	1.12 (0.82-1.51)	1.44 (1.13-1.84)	1.01 (0.77-1.34)	1.30 (1.04-1.61)	1.40 (1.12-1.75)
	30-34.9	1.22 (1.16-1.28)	1.22 (1.17-1.27)	1.18 (1.13-1.23)	1.17 (1.12-1.21)	1.13 (1.10-1.17)	1.14 (1.11-1.18)
	35-39.9	1.20 (1.11-1.30)	1.25 (1.17-1.34)	1.24 (1.16-1.31)	1.21 (1.14-1.28)	1.17 (1.11-1.23)	1.18 (1.12-1.24)
Serious mental illness		0.76 (0.52-1.12)	1.04 (0.78-1.40)	0.73 (0.52-1.02)	0.78 (0.56-1.08)	0.54 (0.38-0.77)	0.71 (0.52-0.96)
Morbidity count	0	1.00	1.00	1.00	1.00	1.00	1.00
	1 / 2+	1.14 (0.93-1.39)	1.11 (0.94-1.32)	1.09 (0.93-1.29)	-	-	1.04 (0.91-1.20)
	1	-	-	-	0.96 (0.80-1.13)	1.10 (0.96-1.26)	-
	2+	-	-	-	1.24 (0.40-3.82)	1.24 (0.46-3.31)	-
Flu vaccine		1.06 (1.01-1.11)	1.03 (0.99-1.07)	1.03 (1.00-1.06)	1.06 (1.02-1.09)	1.13 (1.10-1.16)	1.10 (1.07-1.13)
Number of SARS-CoV-2 tests	0	1.00	1.00	1.00	1.00	1.00	1.00
	1	1.61 (1.55-1.68)	1.52 (1.46-1.58)	1.44 (1.39-1.49)	1.40 (1.35-1.44)	1.34 (1.31-1.38)	1.35 (1.31-1.39)
	2	1.87 (1.75-1.99)	1.66 (1.57-1.77)	1.56 (1.47-1.64)	1.55 (1.47-1.63)	1.51 (1.44-1.58)	1.46 (1.39-1.53)
	3+	2.08 (1.96-2.21)	1.94 (1.84-2.05)	1.95 (1.86-2.05)	2.03 (1.94-2.11)	1.97 (1.90-2.05)	2.01 (1.93-2.09)
Pregnancy		1.45 (1.10-1.92)	1.66 (1.24-2.23)	1.34 (0.97-1.85)	1.15 (0.82-1.60)	0.97 (0.67-1.39)	-

* Estimates correspond to a 1-year increase in age (or age-squared as indicated) within the given age categories.

Supplementary Table 44: Covariate hazard ratios for Non-COVID-19 death in the ChAdOx1 vs unvaccinated comparison and the 40-64 years subgroup.

		Weeks since second dose					
**Variable**	**Category**	**3-6**	**7-10**	**11-14**	**15-18**	**19-22**	**23-26**
Age*	40-45	0.87 (0.58-1.32)	1.36 (0.96-1.94)	0.98 (0.73-1.32)	1.30 (0.99-1.70)	1.29 (0.89-1.89)	1.52 (1.50-1.55)
	45-50	1.05 (0.81-1.37)	1.10 (0.86-1.40)	1.24 (0.95-1.60)	0.95 (0.72-1.25)	1.11 (0.84-1.46)	1.40 (0.89-2.19)
	50-55	1.09 (0.87-1.38)	0.99 (0.79-1.25)	1.11 (0.90-1.37)	1.07 (0.89-1.29)	1.16 (0.96-1.42)	1.04 (0.85-1.27)
	55-60	1.28 (1.02-1.62)	0.93 (0.76-1.14)	1.01 (0.85-1.20)	1.05 (0.90-1.23)	1.14 (0.98-1.33)	1.07 (0.89-1.29)
	60-64	1.23 (0.95-1.59)	1.12 (0.90-1.40)	1.15 (0.88-1.49)	1.22 (0.98-1.53)	0.97 (0.79-1.20)	1.05 (0.83-1.32)
Sex	Female	1.00	1.00	1.00	1.00	1.00	1.00
	Male	2.62 (1.84-3.73)	1.87 (1.37-2.55)	1.58 (1.16-2.16)	1.62 (1.22-2.14)	1.33 (1.01-1.74)	1.38 (1.03-1.84)
IMD	1	1.00	1.00	1.00	1.00	1.00	1.00
	2	0.65 (0.42-1.02)	0.48 (0.31-0.73)	0.85 (0.56-1.27)	0.71 (0.48-1.04)	0.71 (0.50-1.03)	0.72 (0.48-1.10)
	3	0.65 (0.41-1.02)	0.51 (0.33-0.78)	0.62 (0.40-0.97)	0.53 (0.35-0.80)	0.47 (0.31-0.71)	0.73 (0.48-1.10)
	4	0.55 (0.34-0.91)	0.43 (0.27-0.67)	0.61 (0.39-0.96)	0.55 (0.37-0.82)	0.43 (0.28-0.65)	0.44 (0.27-0.71)
	5	0.38 (0.21-0.67)	0.39 (0.24-0.63)	0.48 (0.29-0.79)	0.35 (0.22-0.56)	0.30 (0.18-0.48)	0.47 (0.30-0.74)
Ethnicity	White	1.00	1.00	1.00	1.00	1.00	1.00
	Black	0.36 (0.12-1.13)	-	-	0.39 (0.12-1.21)	-	-
	South Asian / Mixed / Other	0.31 (0.14-0.66)	-	-	0.42 (0.22-0.82)	-	-
	Black / South Asian / Mixed / Other	-	0.63 (0.38-1.05)	0.55 (0.32-0.93)	-	0.60 (0.36-1.02)	0.56 (0.29-1.08)
BMI	<30 or missing	1.00	1.00	1.00	1.00	1.00	1.00
	40+	0.00 (0.00-0.00)	-	0.00 (0.00-0.00)	0.00 (0.00-0.00)	0.00 (0.00-0.00)	-
	30-34.9	0.85 (0.52-1.38)	-	-	-	-	-
	35-39.9	0.95 (0.44-2.06)	-	-	-	-	-
	40+ / 30-34.9 / 35-39.9	-	0.91 (0.61-1.37)	-	-	-	0.61 (0.40-0.94)
	30-34.9 / 35-39.9	-	-	1.16 (0.79-1.68)	0.77 (0.52-1.13)	0.73 (0.50-1.08)	-
Morbidity count	0	1.00	1.00	1.00	1.00	1.00	1.00
	1 / 2+	7.82 (4.05-15.11)	4.87 (2.26-10.49)	6.48 (3.28-12.82)	5.35 (2.62-10.91)	-	5.90 (2.84-12.25)
	1	-	-	-	-	7.17 (3.90-13.18)	-
	2+	-	-	-	-	0.00 (0.00-0.00)	-
Flu vaccine		0.87 (0.57-1.32)	0.87 (0.60-1.27)	0.93 (0.65-1.33)	-	0.95 (0.69-1.31)	0.96 (0.69-1.34)
Number of SARS-CoV-2 tests	0	1.00	1.00	1.00	1.00	1.00	1.00
	1	1.83 (1.22-2.75)	1.18 (0.77-1.80)	1.05 (0.68-1.61)	1.28 (0.90-1.83)	0.79 (0.51-1.22)	-
	2	1.79 (0.90-3.54)	1.60 (0.84-3.04)	-	-	1.39 (0.78-2.50)	-
	3+	2.19 (1.16-4.14)	2.45 (1.44-4.18)	-	-	2.31 (1.44-3.69)	-
	2 / 3+	-	-	2.07 (1.37-3.14)	1.00 (0.60-1.66)	-	-
	1 / 2 / 3+	-	-	-	-	-	0.93 (0.66-1.32)

* Estimates correspond to a 1-year increase in age (or age-squared as indicated) within the given age categories.

Supplementary Table 45: Covariate hazard ratios for Any SARS-CoV-2 test in the ChAdOx1 vs unvaccinated comparison and the 40-64 years subgroup.

		Weeks since second dose					
**Variable**	**Category**	**3-6**	**7-10**	**11-14**	**15-18**	**19-22**	**23-26**
Age*	40-45	0.99 (0.99-1.00)	0.99 (0.99-1.00)	1.00 (0.99-1.00)	1.00 (0.99-1.00)	0.99 (0.98-0.99)	0.99 (0.98-1.00)
	45-50	0.99 (0.98-1.00)	0.99 (0.99-1.00)	0.98 (0.98-0.99)	0.98 (0.98-0.99)	0.98 (0.98-0.99)	0.98 (0.97-0.98)
	50-55	0.98 (0.97-0.98)	0.98 (0.98-0.99)	0.97 (0.97-0.98)	0.98 (0.97-0.98)	0.97 (0.97-0.98)	0.98 (0.97-0.98)
	55-60	0.98 (0.98-0.99)	0.98 (0.98-0.99)	0.98 (0.98-0.99)	0.99 (0.98-0.99)	0.99 (0.98-0.99)	0.99 (0.99-1.00)
	60-64	0.95 (0.94-0.95)	0.95 (0.94-0.96)	0.96 (0.95-0.97)	0.96 (0.95-0.96)	0.96 (0.95-0.97)	0.97 (0.96-0.97)
Sex	Female	1.00	1.00	1.00	1.00	1.00	1.00
	Male	0.77 (0.76-0.77)	0.80 (0.79-0.80)	0.80 (0.79-0.80)	0.80 (0.79-0.80)	0.81 (0.81-0.82)	0.82 (0.82-0.83)
IMD	1	1.00	1.00	1.00	1.00	1.00	1.00
	2	1.11 (1.09-1.13)	1.11 (1.10-1.13)	1.14 (1.13-1.16)	1.18 (1.16-1.19)	1.18 (1.16-1.19)	1.20 (1.18-1.22)
	3	1.19 (1.17-1.21)	1.20 (1.19-1.22)	1.26 (1.24-1.28)	1.32 (1.31-1.34)	1.33 (1.31-1.35)	1.34 (1.32-1.36)
	4	1.28 (1.26-1.30)	1.28 (1.26-1.30)	1.36 (1.35-1.38)	1.45 (1.43-1.47)	1.47 (1.45-1.49)	1.49 (1.47-1.51)
	5	1.38 (1.36-1.40)	1.38 (1.36-1.40)	1.48 (1.46-1.50)	1.58 (1.56-1.60)	1.61 (1.59-1.63)	1.63 (1.61-1.66)
Ethnicity	White	1.00	1.00	1.00	1.00	1.00	1.00
	Black	0.86 (0.83-0.88)	0.86 (0.83-0.88)	0.86 (0.83-0.88)	0.83 (0.80-0.85)	0.82 (0.79-0.84)	0.85 (0.82-0.88)
	South Asian	0.54 (0.53-0.55)	0.55 (0.54-0.56)	0.58 (0.56-0.59)	0.60 (0.59-0.62)	0.60 (0.59-0.61)	0.58 (0.56-0.59)
	Mixed	0.83 (0.80-0.86)	0.86 (0.83-0.90)	0.83 (0.80-0.87)	0.84 (0.81-0.87)	0.84 (0.80-0.87)	0.83 (0.79-0.86)
	Other	0.67 (0.65-0.69)	0.66 (0.64-0.68)	0.66 (0.64-0.68)	0.65 (0.63-0.67)	0.64 (0.62-0.66)	0.62 (0.60-0.64)
BMI	<30 or missing	1.00	1.00	1.00	1.00	1.00	1.00
	40+	0.96 (0.88-1.05)	0.87 (0.80-0.96)	0.97 (0.89-1.05)	0.91 (0.83-0.99)	0.97 (0.90-1.06)	0.96 (0.88-1.05)
	30-34.9	1.00 (0.99-1.01)	1.00 (0.99-1.02)	1.00 (0.99-1.01)	0.99 (0.98-1.00)	0.99 (0.97-1.00)	0.99 (0.98-1.01)
	35-39.9	0.98 (0.96-1.00)	0.99 (0.97-1.01)	0.99 (0.97-1.01)	0.97 (0.95-0.98)	0.96 (0.94-0.98)	0.97 (0.95-0.99)
Serious mental illness		0.75 (0.68-0.83)	0.74 (0.67-0.82)	0.75 (0.68-0.83)	0.69 (0.62-0.76)	0.76 (0.69-0.84)	0.70 (0.63-0.78)
Morbidity count	0	1.00	1.00	1.00	1.00	1.00	1.00
	1	1.01 (0.96-1.06)	1.03 (0.98-1.08)	1.00 (0.95-1.05)	0.99 (0.94-1.03)	0.99 (0.95-1.04)	0.99 (0.94-1.04)
	2+	1.16 (0.82-1.65)	1.37 (1.00-1.86)	0.86 (0.58-1.27)	0.90 (0.62-1.30)	0.96 (0.68-1.37)	1.00 (0.68-1.47)
Flu vaccine		1.31 (1.30-1.32)	1.29 (1.28-1.30)	1.26 (1.25-1.27)	1.26 (1.25-1.27)	1.29 (1.28-1.30)	1.31 (1.30-1.32)
Number of SARS-CoV-2 tests	0	1.00	1.00	1.00	1.00	1.00	1.00
	1	1.46 (1.45-1.48)	1.42 (1.41-1.44)	1.40 (1.39-1.42)	1.36 (1.35-1.37)	1.35 (1.34-1.36)	1.33 (1.32-1.34)
	2	1.87 (1.84-1.90)	1.75 (1.73-1.78)	1.71 (1.68-1.73)	1.65 (1.63-1.68)	1.61 (1.59-1.64)	1.58 (1.56-1.61)
	3+	4.81 (4.75-4.87)	3.71 (3.67-3.76)	3.21 (3.17-3.25)	3.25 (3.21-3.29)	3.01 (2.97-3.04)	2.96 (2.92-3.00)
Pregnancy		1.87 (1.68-2.07)	1.66 (1.48-1.87)	1.57 (1.39-1.77)	1.43 (1.25-1.65)	1.35 (1.17-1.55)	0.97 (0.77-1.22)

* Estimates correspond to a 1-year increase in age (or age-squared as indicated) within the given age categories.

Supplementary Table 46: Covariate hazard ratios for Positive SARS-CoV-2 test in the BNT162b2 vs ChAdOx1 comparison and the 40-64 years subgroup.

		Weeks since second dose					
**Variable**	**Category**	**3-6**	**7-10**	**11-14**	**15-18**	**19-22**	**23-26**
Age*	40-45	0.97 (0.94-1.00)	0.99 (0.97-1.02)	1.00 (0.98-1.02)	0.99 (0.97-1.01)	0.97 (0.96-0.99)	0.98 (0.95-1.00)
	45-50	0.97 (0.94-1.00)	0.97 (0.95-1.00)	0.96 (0.94-0.98)	0.94 (0.93-0.96)	0.95 (0.93-0.97)	0.94 (0.92-0.96)
	50-55	0.94 (0.92-0.97)	0.96 (0.93-0.98)	0.95 (0.93-0.97)	0.92 (0.90-0.94)	0.93 (0.91-0.95)	0.96 (0.94-0.97)
	55-60	0.92 (0.89-0.95)	0.93 (0.91-0.95)	0.94 (0.92-0.96)	0.96 (0.94-0.98)	0.96 (0.94-0.97)	0.97 (0.96-0.99)
	60-64	0.90 (0.84-0.96)	0.96 (0.92-1.00)	0.97 (0.93-1.00)	0.92 (0.88-0.95)	0.95 (0.93-0.98)	0.94 (0.92-0.97)
Sex	Female	1.00	1.00	1.00	1.00	1.00	1.00
	Male	1.03 (0.98-1.07)	1.05 (1.01-1.08)	0.94 (0.92-0.97)	0.92 (0.89-0.94)	0.97 (0.94-0.99)	1.03 (1.01-1.06)
IMD	1	1.00	1.00	1.00	1.00	1.00	1.00
	2	1.00 (0.92-1.07)	0.97 (0.92-1.03)	1.11 (1.05-1.17)	1.10 (1.05-1.16)	1.13 (1.08-1.18)	1.15 (1.10-1.21)
	3	0.97 (0.90-1.05)	0.98 (0.93-1.04)	1.08 (1.02-1.14)	1.18 (1.12-1.24)	1.23 (1.18-1.29)	1.17 (1.12-1.23)
	4	1.01 (0.94-1.08)	0.95 (0.89-1.01)	1.13 (1.07-1.19)	1.23 (1.17-1.30)	1.30 (1.24-1.36)	1.30 (1.24-1.36)
	5	1.12 (1.05-1.20)	1.00 (0.95-1.06)	1.18 (1.12-1.25)	1.31 (1.25-1.38)	1.40 (1.34-1.46)	1.40 (1.34-1.46)
Ethnicity	White	1.00	1.00	1.00	1.00	1.00	1.00
	Black	0.63 (0.50-0.80)	0.72 (0.60-0.86)	0.65 (0.55-0.76)	0.60 (0.51-0.70)	0.57 (0.50-0.65)	0.61 (0.53-0.71)
	South Asian	0.75 (0.67-0.84)	0.84 (0.76-0.92)	0.85 (0.78-0.92)	0.81 (0.76-0.87)	0.69 (0.64-0.74)	0.58 (0.53-0.63)
	Mixed	0.75 (0.59-0.96)	-	0.93 (0.79-1.09)	0.86 (0.74-1.00)	0.74 (0.64-0.84)	-
	Other	0.67 (0.56-0.81)	-	0.67 (0.58-0.77)	0.71 (0.63-0.80)	0.66 (0.59-0.73)	-
	Mixed / Other	-	0.72 (0.64-0.82)	-	-	-	0.65 (0.59-0.71)
BMI	<30 or missing	1.00	1.00	1.00	1.00	1.00	1.00
	40+	1.02 (0.65-1.60)	-	1.41 (1.07-1.86)	1.07 (0.80-1.43)	1.20 (0.95-1.52)	-
	30-34.9	1.12 (1.05-1.19)	-	1.11 (1.07-1.16)	1.09 (1.05-1.14)	1.09 (1.05-1.13)	-
	35-39.9	1.07 (0.97-1.18)	-	1.17 (1.09-1.25)	1.15 (1.08-1.23)	1.13 (1.07-1.20)	-
	40+ / 30-34.9 / 35-39.9	-	1.14 (1.09-1.19)	-	-	-	1.13 (1.09-1.16)
Morbidity count	0	1.00	1.00	1.00	1.00	1.00	1.00
	1 / 2+	0.94 (0.70-1.26)	1.19 (0.97-1.45)	1.02 (0.84-1.23)	-	-	1.03 (0.89-1.20)
	1	-	-	-	0.96 (0.80-1.17)	1.03 (0.89-1.21)	-
	2+	-	-	-	1.69 (0.55-5.18)	1.55 (0.58-4.14)	-
Flu vaccine		1.06 (1.01-1.11)	1.05 (1.01-1.10)	1.07 (1.04-1.11)	1.09 (1.05-1.12)	1.16 (1.13-1.19)	1.13 (1.10-1.16)
Number of SARS-CoV-2 tests	0	1.00	1.00	1.00	1.00	1.00	1.00
	1	1.36 (1.29-1.43)	1.32 (1.26-1.38)	1.27 (1.23-1.32)	1.26 (1.22-1.31)	1.23 (1.20-1.27)	1.27 (1.23-1.31)
	2	1.45 (1.33-1.58)	1.49 (1.39-1.59)	1.39 (1.31-1.48)	1.37 (1.29-1.45)	1.35 (1.29-1.42)	1.35 (1.29-1.42)
	3+	1.75 (1.63-1.88)	1.71 (1.61-1.82)	1.79 (1.70-1.89)	1.86 (1.78-1.95)	1.87 (1.80-1.95)	1.94 (1.86-2.02)
Pregnancy		-	-	1.04 (0.60-1.80)	0.56 (0.30-1.03)	0.72 (0.45-1.16)	-

* Estimates correspond to a 1-year increase in age (or age-squared as indicated) within the given age categories.

Supplementary Table 47: Covariate hazard ratios for Any SARS-CoV-2 test in the BNT162b2 vs ChAdOx1 comparison and the 40-64 years subgroup.

		Weeks since second dose					
**Variable**	**Category**	**3-6**	**7-10**	**11-14**	**15-18**	**19-22**	**23-26**
Age*	40-45	0.99 (0.98-1.00)	0.99 (0.98-1.00)	0.99 (0.99-1.00)	0.99 (0.98-1.00)	0.98 (0.98-0.99)	0.98 (0.97-0.99)
	45-50	0.99 (0.98-0.99)	0.99 (0.98-0.99)	0.98 (0.97-0.99)	0.98 (0.97-0.98)	0.97 (0.97-0.98)	0.97 (0.96-0.98)
	50-55	0.98 (0.97-0.98)	0.98 (0.97-0.99)	0.97 (0.97-0.98)	0.97 (0.97-0.98)	0.97 (0.96-0.97)	0.97 (0.97-0.98)
	55-60	0.98 (0.98-0.99)	0.98 (0.98-0.99)	0.98 (0.98-0.99)	0.99 (0.98-0.99)	0.99 (0.98-0.99)	0.99 (0.99-1.00)
	60-64	0.94 (0.94-0.95)	0.95 (0.94-0.96)	0.95 (0.95-0.96)	0.95 (0.95-0.96)	0.96 (0.95-0.97)	0.97 (0.96-0.97)
Sex	Female	1.00	1.00	1.00	1.00	1.00	1.00
	Male	0.78 (0.77-0.79)	0.82 (0.81-0.82)	0.82 (0.81-0.82)	0.82 (0.81-0.82)	0.83 (0.83-0.84)	0.84 (0.83-0.85)
IMD	1	1.00	1.00	1.00	1.00	1.00	1.00
	2	1.13 (1.11-1.15)	1.12 (1.10-1.14)	1.16 (1.14-1.18)	1.18 (1.17-1.20)	1.18 (1.17-1.20)	1.21 (1.19-1.23)
	3	1.21 (1.19-1.22)	1.21 (1.20-1.23)	1.28 (1.26-1.30)	1.34 (1.32-1.36)	1.34 (1.32-1.36)	1.35 (1.33-1.37)
	4	1.30 (1.28-1.32)	1.29 (1.27-1.31)	1.39 (1.36-1.41)	1.46 (1.44-1.48)	1.48 (1.45-1.50)	1.50 (1.48-1.52)
	5	1.39 (1.37-1.41)	1.39 (1.37-1.41)	1.50 (1.47-1.52)	1.59 (1.57-1.62)	1.62 (1.59-1.64)	1.64 (1.62-1.67)
Ethnicity	White	1.00	1.00	1.00	1.00	1.00	1.00
	Black	0.81 (0.78-0.84)	0.82 (0.79-0.85)	0.80 (0.77-0.83)	0.76 (0.73-0.79)	0.77 (0.74-0.80)	0.80 (0.76-0.83)
	South Asian	0.53 (0.51-0.54)	0.54 (0.53-0.56)	0.59 (0.58-0.61)	0.63 (0.61-0.64)	0.62 (0.61-0.64)	0.59 (0.58-0.61)
	Mixed	0.84 (0.80-0.88)	0.85 (0.81-0.89)	0.84 (0.80-0.88)	0.85 (0.81-0.88)	0.83 (0.79-0.86)	0.82 (0.78-0.86)
	Other	0.69 (0.66-0.71)	0.69 (0.67-0.72)	0.69 (0.66-0.71)	0.68 (0.66-0.71)	0.66 (0.64-0.69)	0.66 (0.63-0.68)
BMI	<30 or missing	1.00	1.00	1.00	1.00	1.00	1.00
	40+	0.92 (0.84-1.01)	0.87 (0.79-0.95)	0.91 (0.83-1.00)	0.89 (0.81-0.97)	0.92 (0.84-1.00)	0.94 (0.85-1.03)
	30-34.9	0.98 (0.97-0.99)	0.98 (0.97-0.99)	0.98 (0.97-0.99)	0.97 (0.96-0.98)	0.97 (0.96-0.98)	0.98 (0.97-0.99)
	35-39.9	0.96 (0.94-0.98)	0.97 (0.95-0.99)	0.97 (0.95-0.99)	0.94 (0.93-0.96)	0.94 (0.92-0.96)	0.96 (0.94-0.98)
Serious mental illness		0.70 (0.62-0.79)	0.67 (0.59-0.76)	0.68 (0.60-0.77)	0.68 (0.61-0.77)	0.72 (0.64-0.80)	0.69 (0.61-0.77)
Morbidity count	0	1.00	1.00	1.00	1.00	1.00	1.00
	1 / 2+	0.96 (0.91-1.01)	-	0.97 (0.92-1.02)	0.95 (0.90-1.00)	0.95 (0.91-1.00)	0.96 (0.92-1.01)
	1	-	0.98 (0.93-1.03)	-	-	-	-
	2+	-	1.16 (0.82-1.65)	-	-	-	-
Flu vaccine		1.30 (1.29-1.31)	1.28 (1.27-1.29)	1.27 (1.26-1.28)	1.26 (1.25-1.27)	1.30 (1.29-1.31)	1.33 (1.31-1.34)
Number of SARS-CoV-2 tests	0	1.00	1.00	1.00	1.00	1.00	1.00
	1	1.37 (1.35-1.38)	1.33 (1.32-1.35)	1.32 (1.30-1.33)	1.29 (1.27-1.30)	1.29 (1.27-1.30)	1.28 (1.27-1.29)
	2	1.74 (1.71-1.77)	1.64 (1.62-1.67)	1.60 (1.57-1.62)	1.56 (1.53-1.58)	1.53 (1.51-1.55)	1.51 (1.48-1.53)
	3+	4.50 (4.45-4.56)	3.47 (3.43-3.52)	3.00 (2.96-3.04)	3.08 (3.04-3.12)	2.86 (2.83-2.90)	2.85 (2.81-2.89)
Pregnancy		1.02 (0.86-1.21)	0.97 (0.83-1.15)	0.84 (0.71-0.99)	0.83 (0.70-0.99)	0.80 (0.68-0.94)	0.55 (0.40-0.77)

* Estimates correspond to a 1-year increase in age (or age-squared as indicated) within the given age categories.

Supplementary Table 48: Covariate hazard ratios for COVID-19 hospitalisation in the BNT162b2 vs unvaccinated comparison and the 18-39 years subgroup.

		Weeks since second dose					
**Variable**	**Category**	**3-6**	**7-10**	**11-14**	**15-18**	**19-22**	**23-26**
Age*	18-25	1.05 (0.96-1.16)	1.13 (1.02-1.24)	1.09 (0.98-1.21)	1.08 (0.93-1.25)	-	-
	25-30	1.09 (0.98-1.20)	1.10 (0.98-1.23)	1.06 (0.95-1.19)	1.05 (0.90-1.24)	1.18 (0.80-1.74)	-
	30-36	0.94 (0.88-1.00)	1.09 (1.01-1.18)	1.06 (0.99-1.14)	1.14 (1.05-1.24)	0.92 (0.82-1.04)	-
	36-40	1.08 (0.96-1.22)	1.10 (0.96-1.25)	1.00 (0.87-1.15)	1.17 (1.02-1.33)	1.10 (0.91-1.33)	-
Sex	Female	1.00	1.00	1.00	1.00	1.00	-
	Male	0.81 (0.68-0.97)	0.69 (0.57-0.85)	0.70 (0.58-0.85)	0.68 (0.54-0.84)	0.77 (0.55-1.09)	-
IMD	1	1.00	1.00	1.00	1.00	1.00	-
	2 / 3 / 4 / 5	0.84 (0.72-0.99)	-	-	-	-	-
	2	-	1.10 (0.88-1.36)	1.11 (0.89-1.38)	0.84 (0.65-1.09)	0.94 (0.61-1.45)	-
	3	-	0.90 (0.70-1.15)	0.90 (0.70-1.16)	0.98 (0.75-1.28)	0.98 (0.62-1.54)	-
	4	-	0.88 (0.67-1.16)	0.88 (0.67-1.17)	0.67 (0.48-0.93)	-	-
	5	-	0.86 (0.63-1.17)	0.91 (0.66-1.25)	0.87 (0.62-1.23)	-	-
	4 / 5	-	-	-	-	0.92 (0.59-1.42)	-
Ethnicity	White	1.00	1.00	1.00	1.00	1.00	-
	Black	1.40 (1.03-1.89)	0.73 (0.46-1.15)	-	-	-	-
	South Asian	1.22 (0.99-1.51)	-	-	-	-	-
	Mixed	1.03 (0.68-1.57)	-	-	-	-	-
	Other	0.76 (0.52-1.13)	-	-	-	-	-
	South Asian / Mixed / Other	-	0.95 (0.76-1.18)	-	-	-	-
	Black / South Asian / Mixed / Other	-	-	1.05 (0.86-1.28)	1.02 (0.82-1.28)	0.85 (0.57-1.27)	-
BMI	<30 or missing	1.00	1.00	1.00	1.00	1.00	-
	40+ / 30-34.9 / 35-39.9	2.47 (2.06-2.96)	-	2.78 (2.26-3.42)	-	-	-
	40+	-	3.21 (1.34-7.68)	-	4.18 (1.56-11.21)	0.00 (0.00-0.00)	-
	30-34.9 / 35-39.9	-	2.01 (1.62-2.50)	-	1.94 (1.50-2.52)	-	-
	30-34.9	-	-	-	-	2.60 (1.67-4.04)	-
	35-39.9	-	-	-	-	2.55 (1.32-4.93)	-
Morbidity count	0	1.00	1.00	1.00	1.00	1.00	-
	1 / 2+	0.92 (0.53-1.62)	-	-	-	1.02 (0.24-4.35)	-
	1	-	1.09 (0.59-2.02)	0.35 (0.11-1.10)	0.71 (0.26-1.93)	-	-
	2+	-	0.00 (0.00-0.00)	0.00 (0.00-0.00)	0.00 (0.00-0.00)	-	-
Flu vaccine		0.95 (0.77-1.16)	1.23 (0.98-1.55)	1.00 (0.78-1.27)	0.93 (0.69-1.25)	0.76 (0.45-1.28)	-
Number of SARS-CoV-2 tests	0	1.00	1.00	1.00	1.00	1.00	-
	1 / 2 / 3+	1.61 (1.38-1.89)	-	-	-	-	-
	1	-	1.61 (1.29-2.00)	1.57 (1.26-1.96)	1.32 (1.00-1.73)	1.56 (1.04-2.33)	-
	2	-	1.87 (1.41-2.48)	1.49 (1.09-2.04)	1.34 (0.91-1.96)	-	-
	3+	-	1.63 (1.25-2.13)	1.95 (1.50-2.52)	2.32 (1.75-3.06)	-	-
	2 / 3+	-	-	-	-	1.80 (1.18-2.75)	-

Supplementary Table 48: Covariate hazard ratios for COVID-19 hospitalisation in the BNT162b2 vs unvaccinated comparison and the 18-39 years subgroup. *(continued)*

		Weeks since second dose					
**Variable**	**Category**	**3-6**	**7-10**	**11-14**	**15-18**	**19-22**	**23-26**
Pregnancy		9.35 (7.68-11.39)	8.10 (6.46-10.16)	6.83 (5.41-8.62)	6.23 (4.69-8.27)	6.66 (4.12-10.77)	-

* Estimates correspond to a 1-year increase in age (or age-squared as indicated) within the given age categories.

Supplementary Table 49: Covariate hazard ratios for Positive SARS-CoV-2 test in the BNT162b2 vs unvaccinated comparison and the 18-39 years subgroup.

		Weeks since second dose					
**Variable**	**Category**	**3-6**	**7-10**	**11-14**	**15-18**	**19-22**	**23-26**
Age*	18-25	0.98 (0.97-1.00)	1.00 (0.99-1.02)	1.02 (1.01-1.03)	1.03 (1.02-1.05)	-	-
	25-30	1.00 (0.98-1.02)	1.04 (1.01-1.06)	1.02 (1.00-1.04)	1.00 (0.98-1.02)	0.98 (0.94-1.02)	-
	30-36	0.99 (0.98-1.00)	1.05 (1.03-1.06)	1.03 (1.02-1.05)	1.02 (1.00-1.03)	0.99 (0.97-1.00)	-
	36-40	0.97 (0.94-1.00)	1.02 (0.99-1.05)	1.02 (0.99-1.04)	1.03 (1.01-1.05)	1.01 (0.99-1.03)	1.05 (0.93-1.18)
Sex	Female	1.00	1.00	1.00	1.00	1.00	1.00
	Male	0.80 (0.77-0.82)	0.73 (0.70-0.75)	0.72 (0.70-0.74)	0.73 (0.71-0.75)	0.79 (0.76-0.82)	0.46 (0.34-0.63)
IMD	1	1.00	1.00	1.00	1.00	1.00	1.00
	2	0.98 (0.94-1.02)	1.04 (1.00-1.08)	1.06 (1.02-1.10)	1.06 (1.02-1.10)	1.06 (1.00-1.12)	1.07 (0.70-1.62)
	3	0.98 (0.94-1.02)	1.01 (0.97-1.06)	1.05 (1.00-1.09)	1.11 (1.07-1.15)	1.13 (1.06-1.20)	1.08 (0.70-1.68)
	4	0.92 (0.88-0.97)	0.97 (0.93-1.02)	1.01 (0.97-1.06)	1.12 (1.08-1.17)	1.12 (1.06-1.20)	1.41 (0.90-2.20)
	5	0.89 (0.84-0.94)	0.91 (0.86-0.96)	1.04 (1.00-1.09)	1.17 (1.12-1.22)	1.20 (1.13-1.28)	1.10 (0.64-1.88)
Ethnicity	White	1.00	1.00	1.00	1.00	1.00	1.00
	Black	0.88 (0.81-0.94)	0.65 (0.59-0.71)	0.74 (0.69-0.80)	0.79 (0.73-0.85)	0.79 (0.70-0.89)	-
	South Asian	0.63 (0.60-0.67)	0.65 (0.61-0.68)	0.59 (0.55-0.62)	0.52 (0.49-0.55)	0.44 (0.41-0.49)	-
	Mixed	0.90 (0.82-0.98)	0.73 (0.67-0.81)	0.80 (0.74-0.87)	0.82 (0.75-0.89)	0.92 (0.81-1.04)	-
	Other	0.44 (0.41-0.49)	0.42 (0.38-0.46)	0.43 (0.40-0.47)	0.44 (0.40-0.48)	0.50 (0.44-0.57)	-
	Black / South Asian / Mixed / Other	-	-	-	-	-	0.61 (0.41-0.92)
BMI	<30 or missing	1.00	1.00	1.00	1.00	1.00	1.00
	40+	1.42 (1.12-1.81)	1.28 (0.99-1.66)	1.44 (1.16-1.79)	1.27 (1.01-1.59)	0.97 (0.66-1.44)	0.00 (0.00-0.00)
	30-34.9	1.29 (1.23-1.37)	1.29 (1.23-1.37)	1.21 (1.15-1.27)	1.11 (1.06-1.17)	1.10 (1.03-1.18)	1.50 (0.92-2.44)
	35-39.9	1.32 (1.22-1.43)	1.24 (1.14-1.34)	1.23 (1.15-1.33)	1.15 (1.07-1.23)	0.91 (0.81-1.02)	0.78 (0.29-2.15)
Serious mental illness		-	-	-	0.80 (0.53-1.20)	-	-
Morbidity count	0	1.00	1.00	1.00	1.00	1.00	1.00
	1	0.92 (0.77-1.11)	-	-	-	1.17 (0.89-1.53)	-
	2+	0.00 (0.00-0.00)	-	-	-	0.00 (0.00-0.00)	-
	1 / 2+	-	1.06 (0.89-1.27)	0.97 (0.81-1.15)	1.24 (1.05-1.46)	-	2.02 (0.49-8.36)
Flu vaccine		1.03 (0.98-1.08)	1.04 (0.99-1.09)	1.03 (0.99-1.07)	0.99 (0.96-1.03)	0.99 (0.94-1.04)	0.78 (0.47-1.29)
Number of SARS-CoV-2 tests	0	1.00	1.00	1.00	1.00	1.00	1.00
	1	1.97 (1.90-2.04)	1.86 (1.79-1.93)	1.84 (1.78-1.90)	1.77 (1.71-1.83)	1.74 (1.65-1.83)	2.29 (1.55-3.39)
	2	2.12 (2.01-2.23)	2.07 (1.96-2.18)	2.04 (1.95-2.13)	1.87 (1.79-1.95)	1.98 (1.86-2.12)	3.48 (2.14-5.65)
	3+	2.51 (2.40-2.63)	2.50 (2.39-2.61)	2.36 (2.27-2.45)	2.22 (2.14-2.31)	2.12 (2.00-2.24)	2.27 (1.37-3.76)
Pregnancy		1.15 (1.07-1.23)	1.15 (1.07-1.23)	1.09 (1.02-1.17)	0.98 (0.90-1.06)	1.01 (0.89-1.14)	-

* Estimates correspond to a 1-year increase in age (or age-squared as indicated) within the given age categories.

Supplementary Table 50: Covariate hazard ratios for Any SARS-CoV-2 test in the BNT162b2 vs unvaccinated comparison and the 18-39 years subgroup.

		Weeks since second dose					
**Variable**	**Category**	**3-6**	**7-10**	**11-14**	**15-18**	**19-22**	**23-26**
Age*	18-25	0.99 (0.99-0.99)	1.02 (1.02-1.03)	1.03 (1.03-1.04)	1.03 (1.02-1.03)	-	-
	25-30	0.99 (0.98-0.99)	1.01 (1.00-1.01)	1.00 (1.00-1.01)	1.01 (1.00-1.02)	0.99 (0.97-1.01)	-
	30-36	0.98 (0.98-0.99)	0.99 (0.99-0.99)	0.99 (0.99-1.00)	0.99 (0.99-1.00)	0.98 (0.98-0.99)	-
	36-40	0.98 (0.97-0.99)	0.99 (0.98-0.99)	1.00 (0.99-1.00)	1.00 (0.99-1.01)	0.99 (0.98-0.99)	1.06 (1.01-1.11)
Sex	Female	1.00	1.00	1.00	1.00	1.00	1.00
	Male	0.80 (0.80-0.81)	0.76 (0.75-0.77)	0.76 (0.75-0.76)	0.75 (0.75-0.76)	0.79 (0.77-0.80)	0.59 (0.53-0.67)
IMD	1	1.00	1.00	1.00	1.00	1.00	1.00
	2	1.08 (1.06-1.09)	1.07 (1.06-1.09)	1.07 (1.06-1.09)	1.09 (1.08-1.11)	1.08 (1.06-1.11)	1.23 (1.04-1.45)
	3	1.13 (1.12-1.15)	1.12 (1.10-1.14)	1.13 (1.12-1.15)	1.16 (1.15-1.18)	1.21 (1.18-1.24)	1.24 (1.04-1.48)
	4	1.19 (1.17-1.20)	1.16 (1.14-1.18)	1.17 (1.15-1.19)	1.22 (1.20-1.24)	1.27 (1.24-1.30)	1.40 (1.17-1.67)
	5	1.24 (1.22-1.25)	1.22 (1.20-1.24)	1.22 (1.20-1.24)	1.28 (1.26-1.30)	1.35 (1.31-1.38)	1.37 (1.13-1.66)
Ethnicity	White	1.00	1.00	1.00	1.00	1.00	1.00
	Black	0.93 (0.90-0.95)	0.87 (0.85-0.90)	0.95 (0.92-0.97)	0.96 (0.93-0.99)	0.92 (0.88-0.97)	-
	South Asian	0.64 (0.63-0.65)	0.69 (0.68-0.70)	0.70 (0.69-0.71)	0.66 (0.65-0.67)	0.60 (0.58-0.62)	-
	Mixed	0.96 (0.93-0.99)	0.92 (0.89-0.95)	0.95 (0.92-0.98)	0.96 (0.93-0.99)	0.92 (0.88-0.97)	-
	Other	0.65 (0.64-0.67)	0.66 (0.65-0.68)	0.60 (0.59-0.62)	0.61 (0.60-0.63)	0.63 (0.61-0.66)	-
	Black / South Asian / Mixed / Other	-	-	-	-	-	0.61 (0.52-0.72)
BMI	<30 or missing	1.00	1.00	1.00	1.00	1.00	1.00
	40+	1.06 (0.97-1.15)	1.08 (0.99-1.17)	1.13 (1.04-1.23)	1.12 (1.02-1.22)	0.98 (0.85-1.13)	1.23 (0.38-4.01)
	30-34.9	1.03 (1.01-1.05)	1.07 (1.05-1.08)	1.09 (1.07-1.11)	1.07 (1.05-1.09)	1.08 (1.06-1.11)	1.17 (0.96-1.44)
	35-39.9	1.03 (1.00-1.05)	1.05 (1.02-1.07)	1.09 (1.06-1.12)	1.08 (1.05-1.11)	1.06 (1.02-1.11)	1.49 (1.13-1.97)
Learning disability		0.98 (0.58-1.65)	1.64 (1.05-2.55)	1.68 (1.07-2.64)	1.84 (1.13-2.99)	-	-
Serious mental illness		0.93 (0.81-1.07)	1.02 (0.88-1.17)	0.93 (0.80-1.08)	0.87 (0.74-1.03)	1.03 (0.82-1.31)	-
Morbidity count	0	1.00	1.00	1.00	1.00	1.00	1.00
	1	1.10 (1.04-1.17)	1.06 (1.00-1.13)	0.90 (0.84-0.97)	0.98 (0.91-1.06)	-	-
	2+	0.97 (0.56-1.68)	1.69 (0.97-2.93)	1.55 (0.95-2.53)	1.40 (0.74-2.63)	-	-
	1 / 2+	-	-	-	-	1.00 (0.89-1.12)	0.94 (0.45-1.97)
Flu vaccine		0.94 (0.93-0.95)	0.96 (0.95-0.98)	0.99 (0.98-1.00)	1.01 (1.00-1.02)	1.02 (1.00-1.04)	1.16 (0.98-1.37)
Number of SARS-CoV-2 tests	0	1.00	1.00	1.00	1.00	1.00	1.00
	1	1.63 (1.61-1.65)	1.61 (1.60-1.63)	1.61 (1.59-1.63)	1.59 (1.57-1.61)	1.61 (1.58-1.64)	2.29 (1.95-2.68)
	2	1.96 (1.94-1.99)	1.91 (1.88-1.94)	1.92 (1.89-1.95)	1.87 (1.84-1.90)	1.99 (1.94-2.04)	3.20 (2.62-3.91)
	3+	3.21 (3.17-3.25)	3.15 (3.11-3.19)	3.08 (3.04-3.12)	2.96 (2.92-3.00)	3.27 (3.20-3.33)	5.63 (4.82-6.57)
Pregnancy		2.00 (1.96-2.04)	1.83 (1.79-1.87)	1.69 (1.65-1.73)	1.47 (1.43-1.51)	1.22 (1.17-1.28)	1.00 (0.67-1.50)

* Estimates correspond to a 1-year increase in age (or age-squared as indicated) within the given age categories.

Waning vaccine effectiveness in risk- and sex-based subgroups

This subsection provides additional results for the waning of vaccine effectiveness across eight subgroups: the four risk-based subgroups, each split into females and males. Supplementary Tables 51 and 52 give the event counts / person-years for each subgroup, outcome and comparison period for females and males respectively. Supplementary Tables 53 and 54 give the unadjusted hazard ratios for females and males respectively. Supplementary Tables 55 and 56 give the adjusted hazard ratios for females and males respectively. Supplementary Tables 57 and 58 give the per-comparison-period ratio of adjusted hazard ratios for females and males respectively. The adjusted hazard ratios are plotted in Supplementary Figures 32 and 33 for BNT162b2 vs unvaccinated and ChAdOx1 vs unvaccinated respectively. The adjusted hazard ratios with any SARS-CoV-2 test as the outcome are plotted in Supplementary Figures 34 and 35 for BNT162b2 vs unvaccinated and ChAdOx1 vs unvaccinated respectively.

Supplementary Table 51: Event counts / person-years (females only).

		65+ years			18-64 years and clinically vulnerable			40-64 years[a]			18-39 years[a]	
**Outcome**	**Weeks since 2nd dose**	**BNT162b2**	**ChAdOx1**	**Unvaccinated**	**BNT162b2**	**ChAdOx1**	**Unvaccinated**	**BNT162b2**	**ChAdOx1**	**Unvaccinated**	**BNT162b2**	**Unvaccinated**
COVID-19 hospitalisation	3-6	14 / 33,746	21 / 43,585	35 / 5,867	7 / 13,737	21 / 25,281	105 / 11,806	7 / 2,451	21 / 53,074	224 / 19,641	14 / 25,737	497 / 46,208
	7-10	14 / 33,799	35 / 43,520	56 / 5,459	21 / 13,718	70 / 25,239	266 / 10,443	7 / 2,449	42 / 53,010	245 / 18,559	21 / 25,555	378 / 43,556
	11-14	56 / 33,736	133 / 43,444	105 / 5,171	28 / 13,700	147 / 25,192	343 / 9,385	7 / 2,445	42 / 52,949	224 / 17,788	28 / 25,412	350 / 41,315
	15-18	119 / 33,663	238 / 43,358	161 / 5,018	56 / 13,676	196 / 25,136	280 / 8,794	7 / 2,442	70 / 52,884	182 / 17,319	28 / 20,511	259 / 28,921
	19-22	161 / 33,583	322 / 43,267	168 / 4,853	56 / 13,646	189 / 25,073	224 / 8,373	7 / 2,338	77 / 52,551	189 / 15,734	21 / 6,476	84 / 9,176
	23-26	217 / 33,477	329 / 43,130	175 / 4,765	49 / 13,573	238 / 24,929	245 / 8,072	7 / 931	98 / 44,833	147 / 10,619	0 / 25	7 / 215
	Total	581 / 202,004	1,078 / 260,304	700 / 31,133	217 / 82,050	861 / 150,850	1,463 / 56,873	42 / 13,056	350 / 309,301	1,211 / 99,660	112 / 103,716	1,575 / 169,391
COVID-19 death	3-6	7 / 33,748	0 / 43,586	14 / 5,870	0 / 13,737	0 / 25,282	7 / 11,811	0 / 2,451	0 / 53,075	7 / 19,655	0 / 25,738	7 / 46,274
	7-10	7 / 33,801	7 / 43,522	14 / 5,464	0 / 13,719	7 / 25,243	14 / 10,461	0 / 2,449	0 / 53,013	7 / 18,590	0 / 25,558	7 / 43,649
	11-14	7 / 33,739	14 / 43,452	21 / 5,180	0 / 13,702	7 / 25,203	21 / 9,422	0 / 2,446	7 / 52,955	14 / 17,837	0 / 25,416	7 / 41,428
	15-18	14 / 33,672	35 / 43,379	28 / 5,034	7 / 13,681	14 / 25,160	42 / 8,854	0 / 2,443	7 / 52,894	7 / 17,377	0 / 20,516	0 / 29,020
	19-22	21 / 33,600	56 / 43,307	49 / 4,879	7 / 13,655	21 / 25,110	28 / 8,448	0 / 2,339	7 / 52,567	7 / 15,801	0 / 6,478	7 / 9,210
	23-26	35 / 33,507	56 / 43,189	42 / 4,799	7 / 13,586	21 / 24,981	21 / 8,158	0 / 931	7 / 44,851	7 / 10,666	0 / 25	0 / 216
	Total	91 / 202,067	168 / 260,435	168 / 31,226	21 / 82,080	70 / 150,979	133 / 57,154	0 / 13,059	28 / 309,355	49 / 99,926	0 / 103,731	28 / 169,797
Positive SARS-CoV-2 test	3-6	56 / 33,742	133 / 43,576	63 / 5,864	70 / 13,731	777 / 25,249	910 / 11,766	133 / 2,438	4,130 / 52,818	2,933 / 19,402	1,477 / 25,518	8,981 / 44,958
	7-10	140 / 33,789	560 / 43,491	119 / 5,453	700 / 13,690	2,884 / 25,074	1,925 / 10,312	217 / 2,422	6,230 / 52,342	3,017 / 18,131	2,359 / 25,197	7,777 / 41,813
	11-14	504 / 33,705	1,589 / 43,342	238 / 5,158	1,211 / 13,597	4,102 / 24,766	2,303 / 9,128	399 / 2,398	8,540 / 51,739	2,926 / 17,159	4,200 / 24,817	9,030 / 39,051
	15-18	1,050 / 33,582	2,548 / 43,114	301 / 4,996	1,603 / 13,468	4,627 / 24,380	1,883 / 8,415	595 / 2,355	10,136 / 50,950	2,961 / 16,521	6,671 / 19,701	7,490 / 26,986
	19-22	1,505 / 33,412	3,213 / 42,818	308 / 4,820	1,813 / 13,317	5,551 / 23,959	1,652 / 7,906	756 / 2,211	13,762 / 49,772	2,933 / 14,844	3,409 / 6,052	2,751 / 8,429
	23-26	1,946 / 33,195	4,333 / 42,435	329 / 4,724	2,674 / 13,076	7,084 / 23,347	1,848 / 7,513	238 / 887	12,817 / 42,040	2,121 / 10,017	28 / 23	98 / 196
	Total	5,201 / 201,425	12,376 / 258,776	1,358 / 31,015	8,071 / 80,879	25,025 / 146,775	10,521 / 55,040	2,338 / 12,711	55,615 / 299,661	16,891 / 96,074	18,144 / 101,308	36,127 / 161,433
Non-COVID-19 death	3-6	336 / 33,748	343 / 43,586	168 / 5,870	42 / 13,737	70 / 25,282	56 / 11,811	7 / 2,451	21 / 53,075	28 / 19,655	7 / 25,738	7 / 46,274
	7-10	455 / 33,801	427 / 43,522	154 / 5,464	49 / 13,719	112 / 25,243	70 / 10,461	0 / 2,449	35 / 53,013	28 / 18,590	0 / 25,558	7 / 43,649
	11-14	546 / 33,739	483 / 43,452	147 / 5,180	56 / 13,702	119 / 25,203	56 / 9,422	7 / 2,446	49 / 52,955	21 / 17,837	0 / 25,416	7 / 41,428
	15-18	602 / 33,672	525 / 43,379	147 / 5,034	63 / 13,681	126 / 25,160	49 / 8,854	7 / 2,443	56 / 52,894	28 / 17,377	0 / 20,516	7 / 29,020
	19-22	630 / 33,600	511 / 43,307	147 / 4,879	56 / 13,655	119 / 25,110	42 / 8,448	7 / 2,339	63 / 52,567	35 / 15,801	0 / 6,478	7 / 9,210
	23-26	700 / 33,507	574 / 43,189	126 / 4,799	70 / 13,586	126 / 24,981	35 / 8,158	7 / 931	63 / 44,851	21 / 10,666	0 / 25	0 / 216
	Total	3,269 / 202,067	2,863 / 260,435	889 / 31,226	336 / 82,080	672 / 150,979	308 / 57,154	35 / 13,059	287 / 309,355	161 / 99,926	7 / 103,731	35 / 169,797
Any SARS-CoV-2 test	3-6	33,544 / 32,291	51,625 / 41,228	3,248 / 5,586	27,335 / 12,366	54,222 / 22,599	12,411 / 10,742	5,292 / 2,192	133,273 / 46,221	18,557 / 17,949	64,967 / 22,811	56,756 / 40,793
	7-10	37,646 / 32,113	57,260 / 40,914	3,199 / 5,183	33,243 / 12,130	62,951 / 22,105	12,565 / 9,348	6,209 / 2,150	134,939 / 45,926	18,452 / 16,717	66,640 / 22,291	48,643 / 38,095
	11-14	41,293 / 31,866	61,537 / 40,549	3,101 / 4,904	30,919 / 12,122	57,512 / 22,190	11,767 / 8,274	6,580 / 2,102	134,540 / 45,819	17,507 / 15,798	65,338 / 22,083	44,058 / 36,043
	15-18	41,391 / 31,754	60,725 / 40,440	3,115 / 4,750	31,647 / 12,117	61,383 / 21,763	10,836 / 7,618	6,741 / 2,058	146,706 / 44,208	16,478 / 15,254	65,065 / 17,509	34,440 / 25,358
	19-22	44,373 / 31,530	67,424 / 39,930	3,080 / 4,577	34,895 / 11,745	65,625 / 21,103	9,975 / 7,139	7,091 / 1,926	149,471 / 43,141	14,973 / 13,814	30,793 / 5,353	13,377 / 8,119
	23-26	48,594 / 31,137	73,325 / 39,331	3,108 / 4,473	35,413 / 11,501	64,743 / 20,573	9,107 / 6,851	3,276 / 789	133,035 / 36,671	11,102 / 9,445	259 / 22	532 / 195
	Total	246,841 / 190,691	371,896 / 242,392	18,851 / 29,473	193,452 / 71,981	366,436 / 130,333	66,661 / 49,972	35,189 / 11,217	831,964 / 261,986	97,069 / 88,977	293,062 / 90,069	197,806 / 148,603

[a] And not clinically vulnerable

Supplementary Table 52: Event counts / person-years (males only).

		65+ years			18-64 years and clinically vulnerable			40-64 years[a]			18-39 years[a]	
**Outcome**	**Weeks since 2nd dose**	**BNT162b2**	**ChAdOx1**	**Unvaccinated**	**BNT162b2**	**ChAdOx1**	**Unvaccinated**	**BNT162b2**	**ChAdOx1**	**Unvaccinated**	**BNT162b2**	**Unvaccinated**
COVID-19 hospitalisation	3-6	21 / 29,925	28 / 40,503	49 / 5,068	7 / 14,532	14 / 24,481	70 / 11,160	0 / 2,380	42 / 59,894	315 / 28,147	7 / 26,977	252 / 56,866
	7-10	28 / 29,948	63 / 40,428	63 / 4,732	14 / 14,510	91 / 24,437	196 / 10,098	0 / 2,377	77 / 59,817	350 / 26,541	7 / 26,826	182 / 54,530
	11-14	70 / 29,874	203 / 40,340	98 / 4,454	42 / 14,486	154 / 24,386	259 / 9,194	7 / 2,373	77 / 59,741	336 / 25,952	14 / 26,691	189 / 52,545
	15-18	189 / 29,786	357 / 40,233	161 / 4,340	56 / 14,459	210 / 24,329	224 / 8,666	0 / 2,369	105 / 59,662	322 / 25,137	14 / 21,020	161 / 38,034
	19-22	252 / 29,691	434 / 40,115	161 / 4,175	49 / 14,428	203 / 24,262	203 / 8,411	0 / 2,259	133 / 59,226	322 / 23,124	7 / 6,359	63 / 12,740
	23-26	364 / 29,570	511 / 39,958	161 / 4,119	84 / 14,349	266 / 24,105	210 / 8,149	0 / 864	161 / 50,022	238 / 14,812	0 / 25	7 / 339
	Total	924 / 178,794	1,596 / 241,577	693 / 26,888	252 / 86,764	938 / 146,000	1,162 / 55,678	7 / 12,622	595 / 348,362	1,883 / 143,713	49 / 107,898	854 / 215,054
COVID-19 death	3-6	7 / 29,927	7 / 40,504	14 / 5,072	0 / 14,533	7 / 24,481	14 / 11,165	0 / 2,380	7 / 59,898	14 / 28,170	0 / 26,978	7 / 56,902
	7-10	7 / 29,951	7 / 40,432	14 / 4,738	0 / 14,511	7 / 24,440	21 / 10,111	0 / 2,377	7 / 59,825	21 / 26,588	0 / 26,827	14 / 54,583
	11-14	14 / 29,879	28 / 40,353	35 / 4,463	7 / 14,489	21 / 24,399	42 / 9,222	0 / 2,373	7 / 59,755	21 / 26,021	0 / 26,693	7 / 52,605
	15-18	28 / 29,799	56 / 40,263	35 / 4,357	7 / 14,465	21 / 24,354	35 / 8,710	0 / 2,369	7 / 59,682	28 / 25,225	0 / 21,022	7 / 38,091
	19-22	77 / 29,717	84 / 40,173	49 / 4,198	14 / 14,437	21 / 24,301	42 / 8,463	0 / 2,259	7 / 59,255	35 / 23,222	0 / 6,359	7 / 12,764
	23-26	77 / 29,613	77 / 40,043	56 / 4,151	7 / 14,362	28 / 24,159	35 / 8,216	0 / 864	14 / 50,055	21 / 14,893	0 / 25	0 / 340
	Total	210 / 178,886	259 / 241,768	203 / 26,979	35 / 86,797	105 / 146,134	189 / 55,887	0 / 12,622	49 / 348,470	140 / 144,119	0 / 107,904	42 / 215,285
Positive SARS-CoV-2 test	3-6	56 / 29,921	119 / 40,494	70 / 5,065	77 / 14,528	623 / 24,457	651 / 11,134	91 / 2,367	4,767 / 59,594	2,947 / 27,908	1,211 / 26,741	7,350 / 55,737
	7-10	182 / 29,938	637 / 40,400	119 / 4,728	721 / 14,484	2,604 / 24,297	1,393 / 10,005	168 / 2,353	7,175 / 59,042	2,884 / 26,129	1,694 / 26,482	6,006 / 52,983
	11-14	581 / 29,840	1,792 / 40,231	182 / 4,445	1,155 / 14,385	3,577 / 24,016	1,575 / 9,013	301 / 2,333	8,862 / 58,383	2,751 / 25,365	3,017 / 26,179	7,112 / 50,623
	15-18	1,232 / 29,695	2,863 / 39,968	287 / 4,322	1,498 / 14,259	3,668 / 23,688	1,162 / 8,418	406 / 2,302	10,220 / 57,570	2,793 / 24,398	4,998 / 20,380	5,992 / 36,397
	19-22	1,568 / 29,508	3,220 / 39,644	266 / 4,154	1,519 / 14,120	4,249 / 23,352	1,036 / 8,107	609 / 2,161	14,469 / 56,265	2,912 / 22,300	2,786 / 6,037	2,331 / 12,126
	23-26	2,177 / 29,273	4,466 / 39,249	287 / 4,085	2,436 / 13,897	5,649 / 22,832	1,113 / 7,796	175 / 829	14,196 / 46,950	2,107 / 14,261	14 / 23	63 / 321
	Total	5,796 / 178,175	13,097 / 239,986	1,211 / 26,799	7,406 / 85,673	20,370 / 142,642	6,930 / 54,473	1,750 / 12,345	59,689 / 337,804	16,394 / 140,361	13,720 / 105,842	28,854 / 208,187
Non-COVID-19 death	3-6	518 / 29,927	469 / 40,504	189 / 5,072	91 / 14,533	126 / 24,481	91 / 11,165	7 / 2,380	70 / 59,898	70 / 28,170	7 / 26,978	14 / 56,902
	7-10	644 / 29,951	546 / 40,432	133 / 4,738	84 / 14,511	140 / 24,440	98 / 10,111	7 / 2,377	77 / 59,825	56 / 26,588	7 / 26,827	21 / 54,583
	11-14	742 / 29,879	665 / 40,353	119 / 4,463	84 / 14,489	161 / 24,399	91 / 9,222	7 / 2,373	77 / 59,755	56 / 26,021	7 / 26,693	21 / 52,605
	15-18	735 / 29,799	721 / 40,263	154 / 4,357	91 / 14,465	182 / 24,354	63 / 8,710	7 / 2,369	105 / 59,682	42 / 25,225	7 / 21,022	14 / 38,091
	19-22	770 / 29,717	672 / 40,173	112 / 4,198	91 / 14,437	175 / 24,301	84 / 8,463	7 / 2,259	105 / 59,255	42 / 23,222	0 / 6,359	7 / 12,764
	23-26	861 / 29,613	784 / 40,043	133 / 4,151	112 / 14,362	182 / 24,159	63 / 8,216	0 / 864	98 / 50,055	35 / 14,893	0 / 25	0 / 340
	Total	4,270 / 178,886	3,857 / 241,768	840 / 26,979	553 / 86,797	966 / 146,134	490 / 55,887	35 / 12,622	532 / 348,470	301 / 144,119	28 / 107,904	77 / 215,285
Any SARS-CoV-2 test	3-6	31,766 / 28,558	48,272 / 38,334	2,695 / 4,844	20,342 / 13,578	36,918 / 22,790	7,525 / 10,559	3,843 / 2,199	108,066 / 54,820	16,436 / 26,763	51,975 / 24,608	43,057 / 52,661
	7-10	35,532 / 28,376	53,781 / 38,016	2,506 / 4,526	25,977 / 13,356	45,171 / 22,329	7,903 / 9,452	4,382 / 2,169	114,415 / 53,990	15,897 / 25,012	50,722 / 24,356	35,756 / 50,327
	11-14	39,172 / 28,115	58,821 / 37,601	2,520 / 4,245	25,599 / 13,222	44,100 / 22,134	7,805 / 8,473	4,522 / 2,147	115,696 / 53,556	15,533 / 24,259	49,189 / 24,191	33,621 / 48,409
	15-18	38,808 / 27,993	57,750 / 37,467	2,646 / 4,121	26,110 / 13,164	45,871 / 21,795	6,972 / 7,916	4,732 / 2,109	127,260 / 52,206	14,476 / 23,367	48,804 / 18,805	26,390 / 35,217
	19-22	41,573 / 27,754	63,973 / 36,956	2,499 / 3,958	28,721 / 12,890	48,972 / 21,330	6,741 / 7,595	5,159 / 1,973	132,433 / 50,912	13,797 / 21,424	23,198 / 5,528	10,500 / 11,888
	23-26	45,822 / 27,351	69,888 / 36,349	2,506 / 3,891	29,365 / 12,654	48,657 / 20,846	6,216 / 7,338	2,240 / 762	117,537 / 42,641	9,856 / 13,801	154 / 22	364 / 320
	Total	232,673 / 168,147	352,485 / 224,723	15,372 / 25,585	156,114 / 78,864	269,689 / 131,224	43,162 / 51,333	24,878 / 11,359	715,407 / 308,125	85,995 / 134,626	224,042 / 97,510	149,688 / 198,822

[a] And not clinically vulnerable

Supplementary Table 53: Unadjusted hazard ratios for effect of vaccination (female-only model).

		65+ years			18-64 years and clinically vulnerable			40-64 years[a]			18-39 years[a]
**Outcome**	**Weeks since 2nd dose**	**BNT162b2 vs unvaccinated**	**ChAdOx1 vs unvaccinated**	**BNT162b2 vs ChAdOx1**	**BNT162b2 vs unvaccinated**	**ChAdOx1 vs unvaccinated**	**BNT162b2 vs ChAdOx1**	**BNT162b2 vs unvaccinated**	**ChAdOx1 vs unvaccinated**	**BNT162b2 vs ChAdOx1**	**BNT162b2 vs unvaccinated**
COVID-19 hospitalisation	3-6	0.08 (0.04-0.17)	0.15 (0.06-0.34)	0.65 (0.23-1.84)	-	0.10 (0.06-0.18)	-	-	0.04 (0.02-0.06)	-	0.03 (0.01-0.06)
	7-10	0.04 (0.02-0.10)	0.06 (0.04-0.09)	0.46 (0.22-0.95)	0.05 (0.03-0.09)	0.11 (0.08-0.14)	0.50 (0.26-0.94)	-	0.05 (0.03-0.07)	-	0.10 (0.06-0.15)
	11-14	0.10 (0.07-0.14)	0.12 (0.09-0.16)	0.52 (0.36-0.74)	0.05 (0.03-0.07)	0.14 (0.11-0.17)	0.35 (0.23-0.54)	-	0.05 (0.04-0.07)	-	0.12 (0.08-0.18)
	15-18	0.10 (0.07-0.13)	0.15 (0.12-0.19)	0.57 (0.45-0.73)	0.10 (0.07-0.13)	0.21 (0.17-0.25)	0.49 (0.35-0.67)	-	0.13 (0.09-0.17)	-	0.11 (0.07-0.18)
	19-22	0.11 (0.09-0.15)	0.20 (0.16-0.25)	0.55 (0.45-0.68)	0.13 (0.10-0.18)	0.24 (0.20-0.30)	0.60 (0.43-0.83)	-	0.13 (0.09-0.18)	-	0.22 (0.12-0.39)
	23-26	0.16 (0.13-0.20)	0.22 (0.18-0.28)	0.64 (0.52-0.79)	0.11 (0.08-0.16)	0.29 (0.24-0.34)	0.37 (0.27-0.50)	-	0.14 (0.11-0.19)	-	-
COVID-19 death	3-6	-	-	-	-	-	-	-	-	-	-
	7-10	-	-	-	-	-	-	-	-	-	-
	11-14	-	0.04 (0.02-0.12)	-	-	0.09 (0.03-0.27)	-	-	-	-	-
	15-18	0.05 (0.02-0.11)	0.11 (0.05-0.21)	0.31 (0.13-0.77)	0.10 (0.04-0.26)	0.12 (0.06-0.23)	0.99 (0.37-2.70)	-	-	-	-
	19-22	0.05 (0.03-0.09)	0.10 (0.06-0.16)	0.49 (0.29-0.82)	0.12 (0.05-0.29)	0.19 (0.10-0.38)	0.84 (0.30-2.35)	-	0.09 (0.03-0.27)	-	-
	23-26	0.12 (0.07-0.19)	0.16 (0.10-0.27)	0.43 (0.28-0.67)	0.33 (0.11-0.99)	0.33 (0.16-0.68)	0.86 (0.34-2.22)	-	0.12 (0.03-0.48)	-	-
Positive SARS-CoV-2 test	3-6	0.25 (0.16-0.41)	0.60 (0.41-0.88)	0.64 (0.44-0.92)	0.14 (0.11-0.19)	0.57 (0.51-0.63)	0.28 (0.22-0.37)	0.31 (0.26-0.37)	0.76 (0.72-0.80)	0.41 (0.35-0.49)	0.28 (0.26-0.29)
	7-10	0.36 (0.27-0.48)	0.74 (0.58-0.94)	0.51 (0.42-0.62)	0.28 (0.26-0.31)	0.59 (0.56-0.63)	0.49 (0.45-0.53)	0.49 (0.42-0.56)	0.87 (0.82-0.91)	0.51 (0.45-0.59)	0.49 (0.47-0.51)
	11-14	0.42 (0.35-0.50)	0.70 (0.60-0.82)	0.61 (0.55-0.68)	0.34 (0.31-0.37)	0.65 (0.61-0.69)	0.57 (0.53-0.61)	0.81 (0.73-0.91)	1.27 (1.21-1.33)	0.55 (0.50-0.61)	0.69 (0.66-0.71)
	15-18	0.59 (0.50-0.68)	0.88 (0.77-1.00)	0.68 (0.63-0.73)	0.47 (0.43-0.50)	0.83 (0.79-0.88)	0.60 (0.57-0.64)	1.13 (1.02-1.24)	1.60 (1.53-1.68)	0.67 (0.61-0.73)	1.12 (1.08-1.16)
	19-22	0.67 (0.58-0.77)	1.12 (0.98-1.28)	0.70 (0.65-0.75)	0.74 (0.68-0.80)	1.13 (1.06-1.20)	0.65 (0.62-0.69)	1.32 (1.21-1.45)	1.84 (1.76-1.92)	0.67 (0.62-0.72)	1.61 (1.52-1.70)
	23-26	0.96 (0.84-1.09)	1.52 (1.34-1.73)	0.72 (0.68-0.76)	0.78 (0.73-0.84)	1.22 (1.16-1.29)	0.66 (0.63-0.69)	1.25 (1.08-1.46)	1.90 (1.80-2.00)	0.64 (0.56-0.73)	2.65 (1.59-4.39)
Non-COVID-19 death	3-6	0.28 (0.22-0.34)	0.50 (0.38-0.66)	0.65 (0.53-0.81)	0.63 (0.40-0.98)	0.53 (0.36-0.79)	1.22 (0.81-1.85)	-	0.28 (0.14-0.56)	-	-
	7-10	0.37 (0.30-0.45)	0.54 (0.40-0.71)	0.70 (0.59-0.84)	0.40 (0.26-0.61)	0.48 (0.34-0.67)	0.91 (0.64-1.30)	-	0.22 (0.12-0.40)	-	-
	11-14	0.47 (0.38-0.58)	0.55 (0.43-0.70)	0.81 (0.69-0.94)	0.56 (0.38-0.84)	0.66 (0.47-0.94)	0.96 (0.69-1.33)	-	0.79 (0.45-1.39)	-	-
	15-18	0.49 (0.40-0.61)	0.70 (0.54-0.90)	0.81 (0.70-0.94)	0.74 (0.50-1.10)	0.81 (0.55-1.17)	0.90 (0.65-1.25)	-	0.42 (0.25-0.71)	-	-
	19-22	0.49 (0.40-0.61)	0.59 (0.46-0.77)	0.89 (0.77-1.03)	0.71 (0.43-1.16)	0.83 (0.55-1.27)	0.93 (0.65-1.32)	-	0.41 (0.23-0.71)	-	-
	23-26	0.58 (0.47-0.71)	0.72 (0.56-0.93)	0.81 (0.71-0.93)	0.97 (0.62-1.52)	1.10 (0.72-1.67)	1.01 (0.74-1.37)	-	0.45 (0.26-0.77)	-	-
Any SARS-CoV-2 test	3-6	2.05 (1.97-2.14)	2.85 (2.73-2.97)	0.84 (0.83-0.85)	2.20 (2.15-2.26)	2.47 (2.42-2.53)	0.76 (0.75-0.77)	2.67 (2.57-2.77)	3.49 (3.43-3.55)	0.93 (0.90-0.95)	2.09 (2.06-2.11)
	7-10	2.31 (2.22-2.41)	2.92 (2.80-3.05)	0.85 (0.84-0.86)	2.01 (1.96-2.05)	2.26 (2.22-2.31)	0.77 (0.76-0.78)	2.58 (2.50-2.67)	3.13 (3.08-3.19)	0.89 (0.87-0.92)	2.41 (2.38-2.44)
	11-14	2.39 (2.29-2.49)	2.90 (2.78-3.02)	0.86 (0.85-0.87)	1.90 (1.85-1.94)	2.09 (2.04-2.13)	0.82 (0.81-0.83)	2.97 (2.87-3.07)	3.25 (3.19-3.31)	0.93 (0.91-0.96)	2.51 (2.47-2.54)
	15-18	2.38 (2.29-2.48)	2.93 (2.81-3.05)	0.86 (0.85-0.87)	1.82 (1.77-1.86)	2.09 (2.04-2.13)	0.82 (0.81-0.83)	3.26 (3.15-3.37)	3.65 (3.59-3.72)	0.94 (0.91-0.96)	2.84 (2.80-2.88)
	19-22	2.44 (2.34-2.54)	3.09 (2.96-3.22)	0.86 (0.85-0.87)	2.11 (2.06-2.17)	2.39 (2.34-2.44)	0.81 (0.80-0.82)	3.41 (3.29-3.53)	3.88 (3.81-3.95)	0.92 (0.90-0.95)	3.86 (3.78-3.95)
	23-26	2.60 (2.49-2.70)	3.22 (3.09-3.36)	0.88 (0.87-0.89)	2.30 (2.24-2.36)	2.56 (2.50-2.62)	0.81 (0.80-0.83)	3.95 (3.77-4.14)	4.42 (4.33-4.51)	0.86 (0.82-0.89)	5.19 (4.42-6.11)

[a] And not clinically vulnerable

Supplementary Table 54: Unadjusted hazard ratios for effect of vaccination (male-only model).

		65+ years			18-64 years and clinically vulnerable			40-64 years[a]			18-39 years[a]
**Outcome**	**Weeks since 2nd dose**	**BNT162b2 vs unvaccinated**	**ChAdOx1 vs unvaccinated**	**BNT162b2 vs ChAdOx1**	**BNT162b2 vs unvaccinated**	**ChAdOx1 vs unvaccinated**	**BNT162b2 vs ChAdOx1**	**BNT162b2 vs unvaccinated**	**ChAdOx1 vs unvaccinated**	**BNT162b2 vs ChAdOx1**	**BNT162b2 vs unvaccinated**
COVID-19 hospitalisation	3-6	0.06 (0.03-0.12)	0.12 (0.07-0.20)	0.48 (0.24-0.95)	0.07 (0.02-0.26)	0.10 (0.05-0.19)	1.02 (0.19-5.47)	-	0.07 (0.05-0.10)	-	0.06 (0.03-0.12)
	7-10	0.08 (0.05-0.15)	0.14 (0.09-0.22)	0.54 (0.31-0.94)	0.05 (0.03-0.10)	0.18 (0.14-0.23)	0.29 (0.16-0.52)	-	0.06 (0.05-0.09)	-	0.06 (0.02-0.15)
	11-14	0.11 (0.08-0.15)	0.19 (0.14-0.25)	0.58 (0.43-0.77)	0.08 (0.06-0.12)	0.21 (0.17-0.26)	0.41 (0.28-0.60)	-	0.06 (0.05-0.08)	-	0.09 (0.05-0.18)
	15-18	0.16 (0.12-0.20)	0.20 (0.16-0.25)	0.66 (0.54-0.81)	0.11 (0.08-0.15)	0.29 (0.23-0.36)	0.41 (0.29-0.57)	-	0.12 (0.09-0.15)	-	0.09 (0.04-0.17)
	19-22	0.19 (0.15-0.25)	0.26 (0.21-0.32)	0.58 (0.48-0.69)	0.12 (0.08-0.16)	0.31 (0.25-0.38)	0.47 (0.33-0.66)	-	0.12 (0.10-0.15)	-	0.13 (0.06-0.32)
	23-26	0.28 (0.22-0.35)	0.35 (0.28-0.43)	0.72 (0.62-0.85)	0.20 (0.15-0.27)	0.37 (0.31-0.45)	0.60 (0.47-0.78)	-	0.17 (0.13-0.21)	-	-
COVID-19 death	3-6	0.03 (0.01-0.10)	-	-	-	-	-	-	-	-	-
	7-10	-	0.05 (0.02-0.16)	-	-	-	-	-	-	-	-
	11-14	0.05 (0.02-0.12)	0.08 (0.05-0.14)	0.63 (0.30-1.33)	-	0.13 (0.07-0.23)	-	-	0.03 (0.01-0.09)	-	-
	15-18	0.12 (0.07-0.22)	0.14 (0.08-0.24)	0.77 (0.44-1.35)	-	0.18 (0.09-0.35)	-	-	0.04 (0.02-0.11)	-	-
	19-22	0.16 (0.10-0.24)	0.14 (0.09-0.22)	0.77 (0.53-1.12)	0.19 (0.08-0.45)	0.16 (0.09-0.30)	1.10 (0.47-2.60)	-	0.04 (0.02-0.10)	-	-
	23-26	0.14 (0.09-0.21)	0.20 (0.13-0.31)	0.79 (0.54-1.16)	0.12 (0.05-0.31)	0.24 (0.14-0.42)	0.42 (0.16-1.07)	-	0.10 (0.04-0.24)	-	-
Positive SARS-CoV-2 test	3-6	0.16 (0.11-0.25)	0.43 (0.30-0.64)	0.72 (0.50-1.03)	0.21 (0.16-0.28)	0.67 (0.59-0.76)	0.39 (0.30-0.50)	0.33 (0.27-0.41)	1.06 (1.00-1.11)	0.32 (0.26-0.39)	0.34 (0.32-0.36)
	7-10	0.43 (0.33-0.57)	0.79 (0.63-0.98)	0.59 (0.49-0.70)	0.35 (0.32-0.39)	0.72 (0.67-0.77)	0.51 (0.47-0.56)	0.61 (0.52-0.72)	1.25 (1.19-1.32)	0.46 (0.39-0.53)	0.56 (0.53-0.59)
	11-14	0.57 (0.47-0.70)	0.98 (0.82-1.17)	0.62 (0.56-0.68)	0.44 (0.40-0.48)	0.86 (0.81-0.92)	0.56 (0.52-0.60)	1.02 (0.89-1.16)	1.62 (1.53-1.70)	0.57 (0.51-0.65)	0.79 (0.76-0.83)
	15-18	0.69 (0.60-0.81)	1.01 (0.89-1.16)	0.71 (0.66-0.76)	0.66 (0.60-0.71)	1.10 (1.02-1.18)	0.64 (0.60-0.68)	1.25 (1.11-1.40)	2.08 (1.98-2.18)	0.57 (0.52-0.64)	1.37 (1.31-1.43)
	19-22	0.86 (0.73-1.00)	1.25 (1.09-1.44)	0.72 (0.67-0.77)	0.91 (0.83-1.00)	1.42 (1.32-1.53)	0.68 (0.64-0.72)	1.68 (1.53-1.85)	2.38 (2.28-2.49)	0.68 (0.62-0.74)	2.19 (2.06-2.32)
	23-26	1.21 (1.04-1.40)	1.67 (1.46-1.90)	0.78 (0.73-0.82)	1.16 (1.07-1.26)	1.71 (1.60-1.84)	0.71 (0.67-0.74)	1.37 (1.15-1.62)	2.63 (2.49-2.77)	0.51 (0.44-0.60)	2.11 (1.04-4.26)
Non-COVID-19 death	3-6	0.36 (0.30-0.44)	0.43 (0.34-0.54)	0.92 (0.78-1.08)	0.69 (0.49-0.97)	0.50 (0.37-0.68)	1.33 (0.99-1.77)	-	0.30 (0.20-0.45)	-	-
	7-10	0.55 (0.45-0.67)	0.65 (0.50-0.84)	0.95 (0.83-1.10)	0.59 (0.42-0.83)	0.49 (0.37-0.65)	1.10 (0.82-1.48)	-	0.34 (0.23-0.50)	-	-
	11-14	0.62 (0.50-0.77)	0.85 (0.65-1.10)	0.89 (0.78-1.01)	0.54 (0.38-0.77)	0.51 (0.39-0.68)	0.94 (0.71-1.24)	-	0.40 (0.27-0.60)	-	-
	15-18	0.53 (0.44-0.65)	0.67 (0.53-0.84)	0.82 (0.72-0.93)	0.82 (0.55-1.22)	0.92 (0.66-1.27)	1.07 (0.81-1.40)	-	0.76 (0.47-1.21)	-	-
	19-22	0.77 (0.62-0.96)	0.83 (0.64-1.08)	0.97 (0.85-1.10)	0.66 (0.46-0.93)	0.71 (0.53-0.95)	0.86 (0.66-1.13)	-	0.70 (0.44-1.11)	-	-
	23-26	0.65 (0.53-0.79)	0.78 (0.61-0.99)	0.97 (0.87-1.10)	0.96 (0.66-1.38)	0.97 (0.70-1.36)	1.05 (0.82-1.35)	-	0.65 (0.42-1.03)	-	-
Any SARS-CoV-2 test	3-6	2.23 (2.14-2.34)	2.83 (2.71-2.96)	0.87 (0.86-0.88)	2.39 (2.32-2.47)	2.60 (2.53-2.67)	0.80 (0.79-0.82)	2.94 (2.82-3.07)	3.87 (3.79-3.94)	0.90 (0.87-0.93)	2.63 (2.59-2.66)
	7-10	2.59 (2.48-2.71)	3.03 (2.90-3.17)	0.87 (0.86-0.88)	2.27 (2.21-2.34)	2.49 (2.43-2.56)	0.81 (0.80-0.83)	3.16 (3.04-3.29)	3.82 (3.75-3.89)	0.87 (0.84-0.90)	3.04 (2.99-3.08)
	11-14	2.57 (2.46-2.69)	3.03 (2.90-3.17)	0.88 (0.86-0.89)	2.17 (2.11-2.24)	2.38 (2.32-2.44)	0.82 (0.81-0.84)	3.40 (3.27-3.54)	4.00 (3.93-4.08)	0.86 (0.83-0.88)	3.08 (3.03-3.12)
	15-18	2.46 (2.35-2.57)	2.87 (2.75-3.00)	0.87 (0.86-0.88)	2.22 (2.16-2.29)	2.51 (2.45-2.58)	0.84 (0.82-0.85)	3.68 (3.54-3.83)	4.40 (4.32-4.49)	0.87 (0.84-0.90)	3.55 (3.49-3.61)
	19-22	2.70 (2.58-2.83)	3.15 (3.02-3.30)	0.87 (0.86-0.89)	2.47 (2.40-2.55)	2.74 (2.66-2.81)	0.85 (0.84-0.87)	3.91 (3.76-4.06)	4.64 (4.55-4.73)	0.87 (0.85-0.90)	5.07 (4.94-5.20)
	23-26	2.97 (2.84-3.10)	3.40 (3.25-3.55)	0.89 (0.88-0.90)	2.66 (2.58-2.74)	2.94 (2.86-3.02)	0.85 (0.83-0.86)	4.37 (4.14-4.62)	5.36 (5.23-5.48)	0.80 (0.76-0.83)	6.12 (4.99-7.52)

[a] And not clinically vulnerable

Supplementary Table 55: Adjusted hazard ratios for effect of vaccination (female-only model).

		65+ years			18-64 years and clinically vulnerable			40-64 years[a]			18-39 years[a]
**Outcome**	**Weeks since 2nd dose**	**BNT162b2 vs unvaccinated**	**ChAdOx1 vs unvaccinated**	**BNT162b2 vs ChAdOx1**	**BNT162b2 vs unvaccinated**	**ChAdOx1 vs unvaccinated**	**BNT162b2 vs ChAdOx1**	**BNT162b2 vs unvaccinated**	**ChAdOx1 vs unvaccinated**	**BNT162b2 vs ChAdOx1**	**BNT162b2 vs unvaccinated**
COVID-19 hospitalisation	3-6	0.09 (0.04-0.19)	0.16 (0.06-0.44)	0.66 (0.23-1.85)	-	0.09 (0.05-0.17)	-	-	0.03 (0.02-0.06)	-	0.04 (0.02-0.08)
	7-10	0.05 (0.02-0.10)	0.06 (0.04-0.10)	0.50 (0.25-1.00)	0.04 (0.02-0.08)	0.09 (0.07-0.12)	0.49 (0.26-0.92)	-	0.04 (0.03-0.07)	-	0.11 (0.07-0.18)
	11-14	0.09 (0.06-0.14)	0.13 (0.09-0.18)	0.52 (0.52-0.52)	0.04 (0.03-0.06)	0.12 (0.10-0.15)	0.35 (0.23-0.54)	-	0.05 (0.03-0.07)	-	0.13 (0.09-0.20)
	15-18	0.10 (0.07-0.14)	0.14 (0.11-0.19)	0.58 (0.46-0.75)	0.08 (0.05-0.11)	0.18 (0.14-0.22)	0.48 (0.35-0.67)	-	0.10 (0.07-0.14)	-	0.12 (0.08-0.20)
	19-22	0.10 (0.08-0.14)	0.19 (0.14-0.24)	0.56 (0.46-0.69)	0.10 (0.07-0.13)	0.19 (0.15-0.24)	0.59 (0.43-0.81)	-	0.12 (0.08-0.17)	-	0.23 (0.12-0.43)
	23-26	0.15 (0.11-0.20)	0.20 (0.16-0.26)	0.65 (0.53-0.79)	0.07 (0.05-0.10)	0.21 (0.16-0.26)	0.35 (0.26-0.48)	-	0.12 (0.09-0.16)	-	-
COVID-19 death	3-6	-	-	-	-	-	-	-	-	-	-
	7-10	-	-	-	-	-	-	-	-	-	-
	11-14	-	0.09 (0.09-0.09)	-	-	0.07 (0.02-0.23)	-	-	-	-	-
	15-18	0.05 (0.02-0.12)	0.11 (0.05-0.24)	0.32 (0.13-0.77)	0.06 (0.02-0.16)	0.07 (0.04-0.14)	0.95 (0.35-2.62)	-	-	-	-
	19-22	0.05 (0.03-0.10)	0.11 (0.06-0.19)	0.50 (0.30-0.83)	0.10 (0.04-0.25)	0.17 (0.08-0.34)	0.86 (0.31-2.41)	-	0.08 (0.02-0.25)	-	-
	23-26	0.12 (0.07-0.22)	0.16 (0.10-0.27)	0.45 (0.29-0.69)	0.14 (0.05-0.42)	0.18 (0.08-0.39)	0.81 (0.32-2.09)	-	0.12 (0.02-0.59)	-	-
Positive SARS-CoV-2 test	3-6	0.25 (0.14-0.42)	0.57 (0.37-0.86)	0.64 (0.44-0.92)	0.20 (0.15-0.26)	0.72 (0.64-0.81)	0.30 (0.23-0.39)	0.27 (0.22-0.32)	0.67 (0.63-0.71)	0.42 (0.35-0.50)	0.23 (0.21-0.24)
	7-10	0.34 (0.24-0.48)	0.69 (0.53-0.91)	0.51 (0.42-0.62)	0.35 (0.32-0.39)	0.73 (0.68-0.79)	0.51 (0.47-0.56)	0.42 (0.36-0.48)	0.78 (0.74-0.82)	0.52 (0.45-0.60)	0.40 (0.38-0.42)
	11-14	0.36 (0.29-0.45)	0.59 (0.49-0.70)	0.61 (0.55-0.67)	0.41 (0.38-0.45)	0.77 (0.72-0.82)	0.59 (0.55-0.63)	0.69 (0.61-0.77)	1.13 (1.08-1.19)	0.56 (0.50-0.62)	0.54 (0.52-0.57)
	15-18	0.51 (0.42-0.61)	0.76 (0.65-0.88)	0.68 (0.63-0.73)	0.53 (0.49-0.58)	0.93 (0.87-0.99)	0.62 (0.59-0.66)	0.93 (0.84-1.03)	1.36 (1.29-1.42)	0.68 (0.62-0.74)	0.89 (0.85-0.92)
	19-22	0.57 (0.48-0.66)	0.93 (0.81-1.08)	0.70 (0.65-0.74)	0.78 (0.72-0.86)	1.16 (1.09-1.24)	0.68 (0.64-0.72)	1.10 (1.00-1.20)	1.53 (1.46-1.60)	0.68 (0.63-0.73)	1.24 (1.17-1.32)
	23-26	0.80 (0.68-0.93)	1.18 (1.03-1.35)	0.72 (0.68-0.76)	0.81 (0.75-0.88)	1.24 (1.17-1.32)	0.68 (0.65-0.72)	1.01 (0.86-1.18)	1.58 (1.50-1.67)	0.65 (0.57-0.74)	2.06 (1.20-3.54)
Non-COVID-19 death	3-6	0.26 (0.19-0.34)	0.40 (0.30-0.54)	0.72 (0.59-0.88)	0.46 (0.28-0.75)	0.39 (0.25-0.60)	1.18 (0.78-1.79)	-	0.29 (0.12-0.70)	-	-
	7-10	0.36 (0.27-0.47)	0.42 (0.32-0.57)	0.76 (0.65-0.90)	0.23 (0.13-0.39)	0.28 (0.18-0.42)	0.88 (0.62-1.27)	-	0.25 (0.12-0.50)	-	-
	11-14	0.50 (0.38-0.64)	0.50 (0.39-0.66)	0.86 (0.74-0.99)	0.29 (0.18-0.47)	0.41 (0.27-0.62)	0.92 (0.67-1.28)	-	0.82 (0.46-1.46)	-	-
	15-18	0.46 (0.36-0.60)	0.62 (0.46-0.82)	0.86 (0.75-0.99)	0.40 (0.26-0.63)	0.59 (0.39-0.90)	0.88 (0.64-1.22)	-	0.45 (0.25-0.82)	-	-
	19-22	0.50 (0.39-0.65)	0.58 (0.43-0.78)	0.95 (0.82-1.09)	0.45 (0.25-0.80)	0.56 (0.35-0.89)	0.90 (0.63-1.28)	-	0.48 (0.26-0.86)	-	-
	23-26	0.59 (0.45-0.77)	0.68 (0.51-0.91)	0.87 (0.76-0.99)	0.59 (0.35-1.01)	0.73 (0.46-1.15)	0.96 (0.70-1.32)	-	0.49 (0.27-0.91)	-	-
Any SARS-CoV-2 test	3-6	1.53 (1.46-1.60)	2.03 (1.94-2.12)	0.85 (0.84-0.86)	2.14 (2.08-2.20)	2.30 (2.25-2.35)	0.77 (0.76-0.79)	2.04 (1.96-2.12)	2.65 (2.60-2.69)	0.94 (0.91-0.97)	1.72 (1.69-1.74)
	7-10	1.74 (1.66-1.82)	2.08 (1.99-2.18)	0.86 (0.85-0.87)	1.98 (1.93-2.03)	2.12 (2.07-2.17)	0.79 (0.78-0.80)	1.98 (1.91-2.05)	2.41 (2.37-2.45)	0.91 (0.89-0.94)	1.95 (1.93-1.98)
	11-14	1.78 (1.70-1.87)	2.09 (2.00-2.18)	0.87 (0.86-0.88)	1.83 (1.78-1.88)	1.94 (1.90-1.99)	0.83 (0.82-0.84)	2.30 (2.21-2.38)	2.54 (2.50-2.59)	0.95 (0.93-0.98)	1.99 (1.96-2.02)
	15-18	1.80 (1.72-1.88)	2.15 (2.06-2.25)	0.87 (0.85-0.88)	1.72 (1.67-1.77)	1.92 (1.88-1.97)	0.83 (0.82-0.84)	2.49 (2.40-2.59)	2.81 (2.76-2.86)	0.95 (0.92-0.97)	2.22 (2.19-2.26)
	19-22	1.83 (1.75-1.91)	2.22 (2.13-2.32)	0.87 (0.86-0.88)	1.98 (1.93-2.04)	2.16 (2.11-2.21)	0.82 (0.81-0.84)	2.59 (2.49-2.69)	2.99 (2.93-3.05)	0.94 (0.91-0.96)	2.82 (2.75-2.89)
	23-26	1.97 (1.88-2.05)	2.31 (2.21-2.41)	0.88 (0.87-0.90)	2.06 (2.00-2.12)	2.25 (2.20-2.31)	0.83 (0.82-0.84)	2.84 (2.70-2.99)	3.34 (3.26-3.41)	0.87 (0.84-0.90)	3.10 (2.59-3.71)

[a] And not clinically vulnerable

Supplementary Table 56: Adjusted hazard ratios for effect of vaccination (male-only model).

		65+ years			18-64 years and clinically vulnerable			40-64 years[a]			18-39 years[a]
**Outcome**	**Weeks since 2nd dose**	**BNT162b2 vs unvaccinated**	**ChAdOx1 vs unvaccinated**	**BNT162b2 vs ChAdOx1**	**BNT162b2 vs unvaccinated**	**ChAdOx1 vs unvaccinated**	**BNT162b2 vs ChAdOx1**	**BNT162b2 vs unvaccinated**	**ChAdOx1 vs unvaccinated**	**BNT162b2 vs ChAdOx1**	**BNT162b2 vs unvaccinated**
COVID-19 hospitalisation	3-6	0.06 (0.03-0.12)	0.11 (0.06-0.20)	0.48 (0.24-0.96)	0.06 (0.02-0.25)	0.08 (0.04-0.18)	1.04 (0.19-5.54)	-	0.06 (0.04-0.09)	-	0.05 (0.02-0.10)
	7-10	0.08 (0.05-0.15)	0.14 (0.09-0.23)	0.54 (0.31-0.95)	0.04 (0.02-0.07)	0.12 (0.09-0.17)	0.28 (0.15-0.51)	-	0.06 (0.04-0.08)	-	0.05 (0.02-0.11)
	11-14	0.09 (0.06-0.13)	0.06 (0.04-0.08)	0.57 (0.43-0.76)	0.06 (0.04-0.08)	0.16 (0.13-0.21)	0.40 (0.28-0.59)	-	0.06 (0.04-0.08)	-	0.07 (0.04-0.15)
	15-18	0.16 (0.12-0.22)	0.21 (0.16-0.28)	0.66 (0.54-0.80)	0.07 (0.05-0.11)	0.21 (0.16-0.26)	0.39 (0.28-0.55)	-	0.09 (0.07-0.12)	-	0.07 (0.04-0.15)
	19-22	0.21 (0.16-0.27)	0.24 (0.19-0.31)	0.57 (0.48-0.69)	0.07 (0.05-0.10)	0.20 (0.15-0.25)	0.45 (0.32-0.63)	-	0.11 (0.09-0.15)	-	0.11 (0.04-0.28)
	23-26	0.25 (0.20-0.33)	0.30 (0.23-0.38)	0.72 (0.61-0.84)	0.12 (0.09-0.17)	0.25 (0.20-0.32)	0.58 (0.45-0.75)	-	0.12 (0.09-0.16)	-	-
COVID-19 death	3-6	0.03 (0.01-0.10)	-	-	-	-	-	-	-	-	-
	7-10	-	0.05 (0.02-0.16)	-	-	-	-	-	-	-	-
	11-14	-	-	0.79 (0.79-0.79)	-	0.08 (0.04-0.16)	-	-	0.02 (0.01-0.06)	-	-
	15-18	0.10 (0.05-0.20)	0.11 (0.06-0.21)	0.76 (0.43-1.34)	-	0.12 (0.06-0.22)	-	-	0.03 (0.01-0.10)	-	-
	19-22	0.14 (0.08-0.22)	0.11 (0.07-0.18)	0.76 (0.52-1.11)	0.10 (0.04-0.24)	0.10 (0.05-0.19)	1.12 (0.47-2.66)	-	0.04 (0.02-0.10)	-	-
	23-26	0.12 (0.07-0.19)	0.16 (0.10-0.24)	0.79 (0.54-1.16)	0.06 (0.02-0.15)	0.13 (0.07-0.23)	0.40 (0.15-1.04)	-	0.09 (0.04-0.23)	-	-
Positive SARS-CoV-2 test	3-6	0.15 (0.09-0.23)	0.33 (0.22-0.50)	0.72 (0.50-1.03)	0.25 (0.18-0.33)	0.78 (0.68-0.90)	0.40 (0.31-0.52)	0.26 (0.21-0.33)	0.89 (0.84-0.94)	0.32 (0.26-0.40)	0.27 (0.25-0.28)
	7-10	0.36 (0.26-0.49)	0.71 (0.55-0.91)	0.58 (0.49-0.69)	0.40 (0.35-0.45)	0.82 (0.75-0.88)	0.53 (0.49-0.58)	0.49 (0.42-0.58)	1.09 (1.03-1.15)	0.46 (0.39-0.54)	0.43 (0.40-0.46)
	11-14	0.46 (0.37-0.57)	0.85 (0.70-1.03)	0.62 (0.57-0.69)	0.49 (0.44-0.54)	0.97 (0.90-1.04)	0.58 (0.54-0.62)	0.83 (0.72-0.94)	1.36 (1.29-1.44)	0.59 (0.52-0.66)	0.62 (0.59-0.66)
	15-18	0.62 (0.52-0.74)	0.87 (0.76-1.01)	0.71 (0.66-0.76)	0.62 (0.56-0.69)	1.08 (1.00-1.17)	0.65 (0.61-0.69)	0.96 (0.85-1.08)	1.73 (1.65-1.82)	0.58 (0.53-0.65)	1.05 (1.01-1.10)
	19-22	0.77 (0.65-0.91)	1.07 (0.92-1.24)	0.72 (0.67-0.77)	0.85 (0.76-0.95)	1.29 (1.19-1.40)	0.68 (0.64-0.73)	1.35 (1.22-1.50)	1.96 (1.87-2.05)	0.69 (0.64-0.75)	1.65 (1.54-1.76)
	23-26	0.96 (0.81-1.12)	1.28 (1.11-1.48)	0.77 (0.73-0.82)	1.02 (0.93-1.13)	1.51 (1.40-1.63)	0.71 (0.68-0.75)	0.99 (0.82-1.19)	2.12 (2.01-2.24)	0.53 (0.45-0.62)	1.24 (0.58-2.64)
Non-COVID-19 death	3-6	0.32 (0.25-0.41)	0.34 (0.26-0.43)	0.93 (0.80-1.09)	0.44 (0.29-0.67)	0.32 (0.23-0.45)	1.27 (0.95-1.70)	-	0.30 (0.19-0.47)	-	-
	7-10	0.49 (0.38-0.62)	0.63 (0.47-0.84)	0.96 (0.84-1.11)	0.37 (0.25-0.55)	0.32 (0.23-0.44)	1.04 (0.78-1.40)	-	0.37 (0.24-0.57)	-	-
	11-14	0.53 (0.41-0.69)	0.81 (0.60-1.09)	0.90 (0.79-1.02)	0.35 (0.23-0.53)	0.33 (0.24-0.45)	0.90 (0.68-1.19)	-	0.38 (0.25-0.59)	-	-
	15-18	0.51 (0.40-0.65)	0.60 (0.47-0.77)	0.83 (0.73-0.94)	0.48 (0.30-0.77)	0.66 (0.45-0.97)	1.01 (0.77-1.33)	-	0.78 (0.47-1.27)	-	-
	19-22	0.71 (0.54-0.92)	0.80 (0.60-1.07)	0.97 (0.86-1.10)	0.42 (0.28-0.62)	0.49 (0.35-0.67)	0.82 (0.63-1.07)	-	0.75 (0.46-1.22)	-	-
	23-26	0.57 (0.45-0.72)	0.79 (0.61-1.03)	0.98 (0.88-1.11)	0.50 (0.34-0.75)	0.67 (0.46-0.97)	0.99 (0.77-1.27)	-	0.68 (0.41-1.13)	-	-
Any SARS-CoV-2 test	3-6	1.58 (1.50-1.66)	2.00 (1.91-2.10)	0.87 (0.86-0.89)	1.90 (1.83-1.97)	2.13 (2.06-2.19)	0.80 (0.79-0.82)	2.18 (2.09-2.28)	2.97 (2.91-3.03)	0.92 (0.89-0.95)	1.97 (1.94-2.00)
	7-10	1.85 (1.76-1.94)	2.12 (2.02-2.22)	0.88 (0.86-0.89)	1.88 (1.82-1.94)	2.08 (2.02-2.14)	0.81 (0.80-0.83)	2.39 (2.29-2.49)	2.98 (2.92-3.03)	0.89 (0.87-0.92)	2.28 (2.25-2.32)
	11-14	1.84 (1.75-1.93)	2.16 (2.06-2.26)	0.88 (0.87-0.89)	1.82 (1.76-1.88)	2.04 (1.98-2.10)	0.83 (0.81-0.84)	2.57 (2.46-2.68)	3.12 (3.06-3.18)	0.88 (0.85-0.91)	2.32 (2.28-2.36)
	15-18	1.82 (1.73-1.90)	2.05 (1.96-2.15)	0.87 (0.86-0.89)	1.86 (1.79-1.92)	2.12 (2.06-2.18)	0.84 (0.82-0.85)	2.73 (2.62-2.84)	3.43 (3.36-3.50)	0.89 (0.86-0.92)	2.65 (2.61-2.70)
	19-22	1.99 (1.89-2.08)	2.29 (2.19-2.40)	0.88 (0.87-0.89)	2.00 (1.94-2.07)	2.24 (2.18-2.31)	0.85 (0.84-0.87)	2.89 (2.78-3.02)	3.60 (3.52-3.67)	0.90 (0.87-0.92)	3.52 (3.43-3.63)
	23-26	2.19 (2.08-2.30)	2.43 (2.32-2.54)	0.89 (0.88-0.90)	2.12 (2.05-2.20)	2.39 (2.32-2.47)	0.85 (0.83-0.86)	3.07 (2.89-3.27)	4.09 (3.99-4.19)	0.82 (0.78-0.85)	3.09 (2.44-3.90)

[a] And not clinically vulnerable

Supplementary Table 57: Per-comparison-period ratio of adjusted hazard ratios for effect of vaccination (female-only model).

	65+ years			18-64 years and clinically vulnerable			40-64 years[a]			18-39 years[a]
**Outcome**	**BNT162b2 vs unvaccinated**	**ChAdOx1 vs unvaccinated**	**BNT162b2 vs ChAdOx1**	**BNT162b2 vs unvaccinated**	**ChAdOx1 vs unvaccinated**	**BNT162b2 vs ChAdOx1**	**BNT162b2 vs unvaccinated**	**ChAdOx1 vs unvaccinated**	**BNT162b2 vs ChAdOx1**	**BNT162b2 vs unvaccinated**
COVID-19 hospitalisation	1.17 (1.05-1.32)	1.22 (1.09-1.38)	1.04 (0.93-1.17)	1.21 (0.99-1.47)	1.22 (1.13-1.31)	0.99 (0.82-1.19)	-	1.31 (1.19-1.44)	-	1.38 (1.09-1.76)
COVID-19 death	1.71 (1.04-2.81)	1.25 (0.80-1.93)	1.09 (0.70-1.70)	1.49 (0.72-3.06)	1.51 (1.00-2.28)	0.92 (0.46-1.84)	-	-	-	-
Positive SARS-CoV-2 test	1.26 (1.18-1.34)	1.18 (1.10-1.26)	1.05 (1.02-1.09)	1.30 (1.22-1.38)	1.14 (1.10-1.18)	1.13 (1.04-1.22)	1.32 (1.19-1.47)	1.20 (1.15-1.26)	1.09 (1.04-1.14)	1.52 (1.46-1.59)
Non-COVID-19 death	1.16 (1.08-1.23)	1.12 (1.04-1.20)	1.04 (1.00-1.08)	1.10 (0.95-1.29)	1.18 (1.06-1.31)	0.98 (0.90-1.07)	-	1.10 (0.89-1.37)	-	-
Any SARS-CoV-2 test	1.04 (1.02-1.06)	1.03 (1.01-1.04)	1.01 (1.00-1.01)	0.99 (0.95-1.03)	1.00 (0.96-1.04)	1.01 (1.00-1.02)	1.08 (1.05-1.10)	1.06 (1.02-1.09)	0.99 (0.98-1.01)	1.12 (1.09-1.16)

[a] And not clinically vulnerable

Supplementary Table 58: Per-comparison-period ratio of adjusted hazard ratios for effect of vaccination (male-only model).

	**65+ years**			**18-64 years and clinically vulnerable**			**40-64 years[a]**			**18-39 years[a]**
**Outcome**	**BNT162b2 vs unvaccinated**	**ChAdOx1 vs unvaccinated**	**BNT162b2 vs ChAdOx1**	**BNT162b2 vs unvaccinated**	**ChAdOx1 vs unvaccinated**	**BNT162b2 vs ChAdOx1**	**BNT162b2 vs unvaccinated**	**ChAdOx1 vs unvaccinated**	**BNT162b2 vs ChAdOx1**	**BNT162b2 vs unvaccinated**
COVID-19 hospitalisation	1.35 (1.22-1.49)	1.27 (1.00-1.60)	1.06 (0.98-1.15)	1.27 (1.12-1.44)	1.18 (1.10-1.28)	1.14 (1.01-1.27)	-	1.21 (1.12-1.30)	-	1.21 (0.93-1.57)
COVID-19 death	1.26 (0.99-1.61)	1.27 (0.97-1.65)	1.02 (0.74-1.42)	-	1.13 (0.85-1.49)	-	-	1.64 (1.03-2.62)	-	-
Positive SARS-CoV-2 test	1.32 (1.22-1.43)	1.20 (1.11-1.29)	1.06 (1.03-1.09)	1.29 (1.25-1.33)	1.15 (1.13-1.18)	1.08 (1.05-1.10)	1.32 (1.14-1.53)	1.20 (1.16-1.24)	1.11 (1.01-1.22)	1.57 (1.52-1.62)
Non-COVID-19 death	1.12 (1.03-1.21)	1.15 (1.02-1.29)	1.01 (0.97-1.05)	1.04 (0.94-1.14)	1.17 (1.06-1.30)	0.95 (0.89-1.02)	-	1.22 (1.09-1.37)	-	0.95 (0.56-1.60)
Any SARS-CoV-2 test	1.05 (1.03-1.08)	1.03 (1.01-1.05)	1.00 (1.00-1.01)	1.02 (1.00-1.04)	1.02 (1.00-1.05)	1.01 (1.01-1.02)	1.07 (1.06-1.08)	1.07 (1.05-1.09)	0.99 (0.97-1.00)	1.13 (1.08-1.18)

[a] And not clinically vulnerable

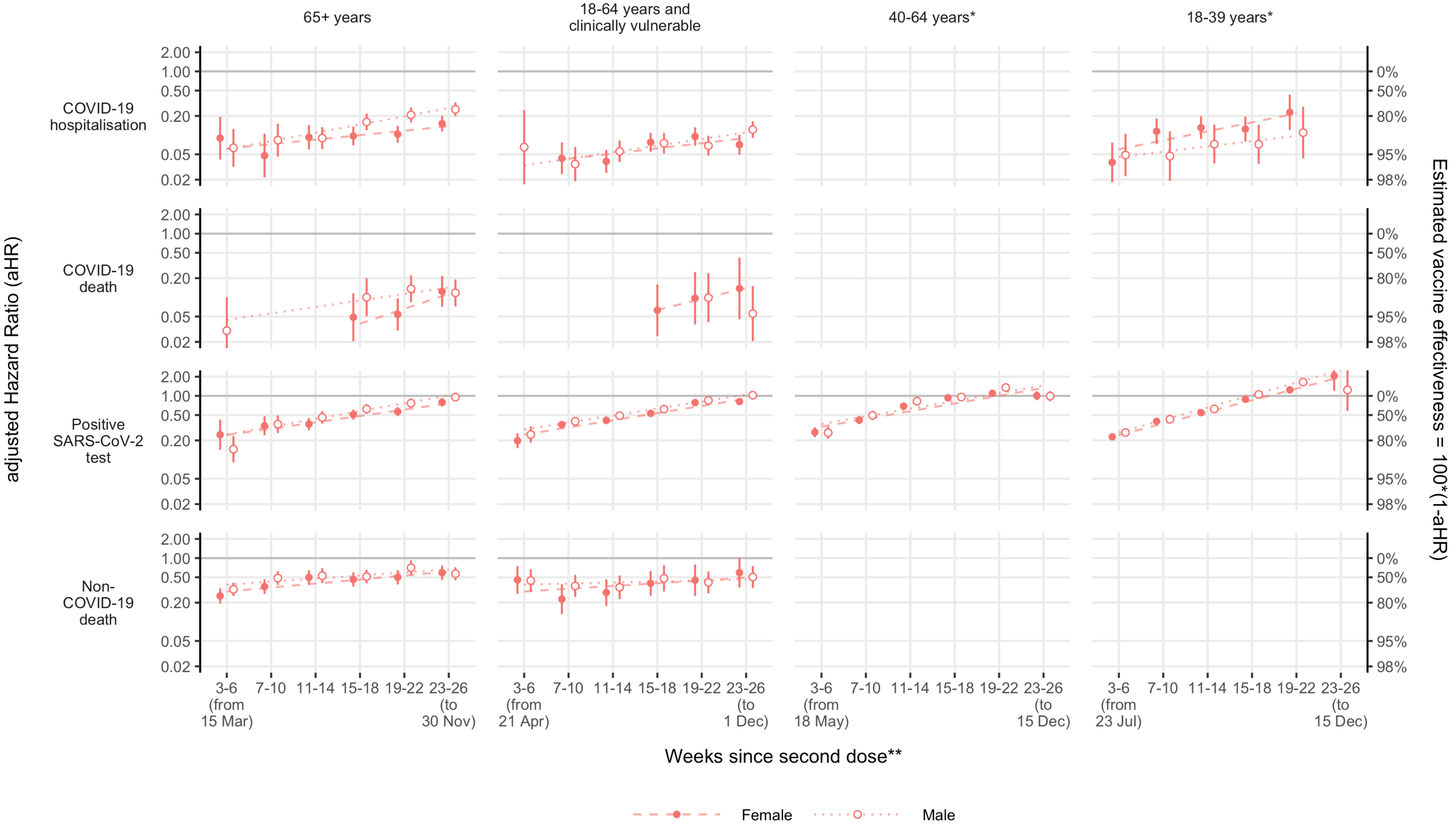

Supplementary Figure 32: Adjusted hazard ratios for BNT162b2 vs unvaccinated from the female- and male-only models.

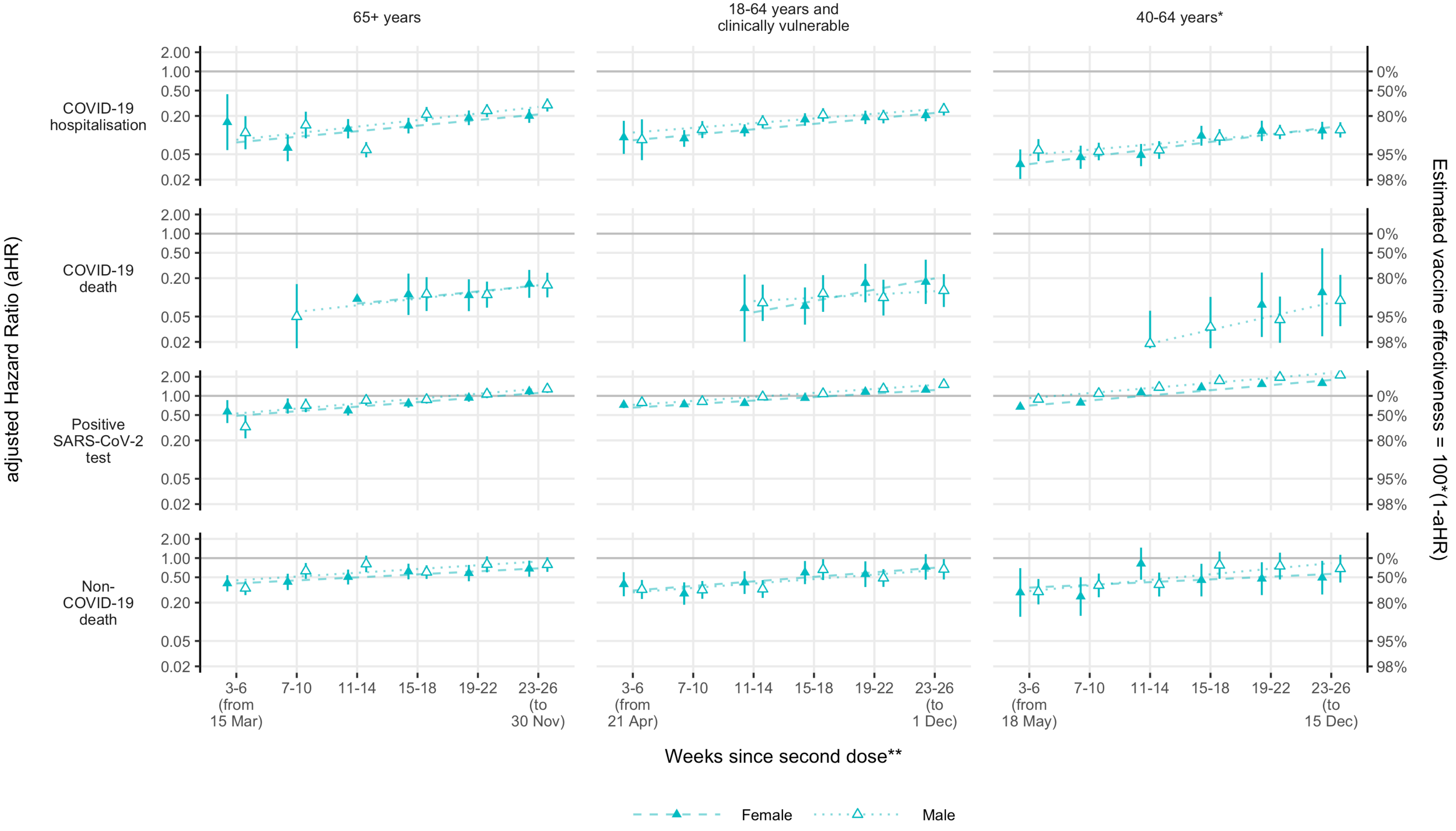

Supplementary Figure 33: Adjusted hazard ratios for ChAdOx1 vs unvaccinated from the female- and male-only models.

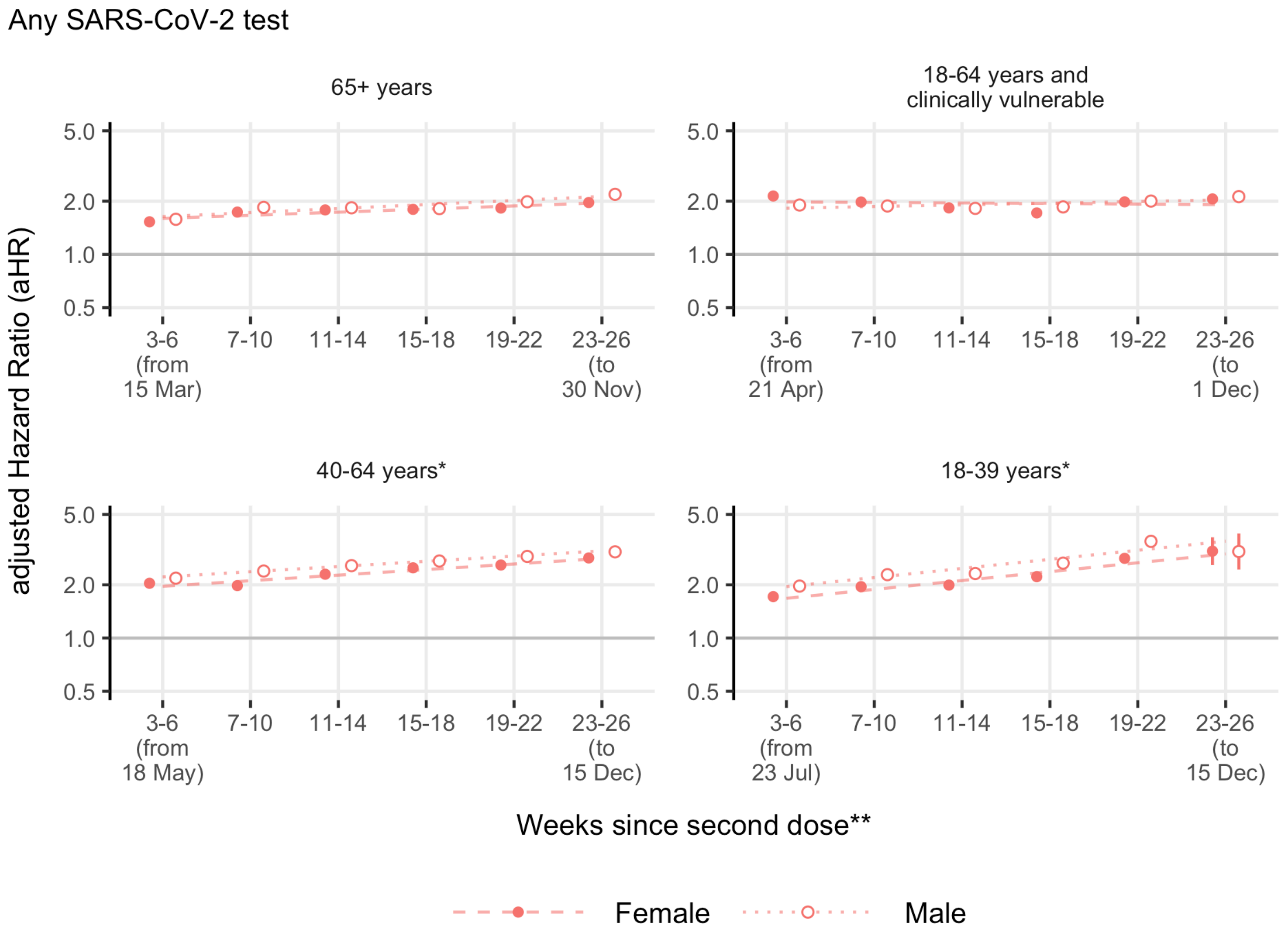

Supplementary Figure 34: Adjusted hazard ratios for any SARS-CoV-2 test for BNT162b2 vs unvaccinated from the female- and male-only models.

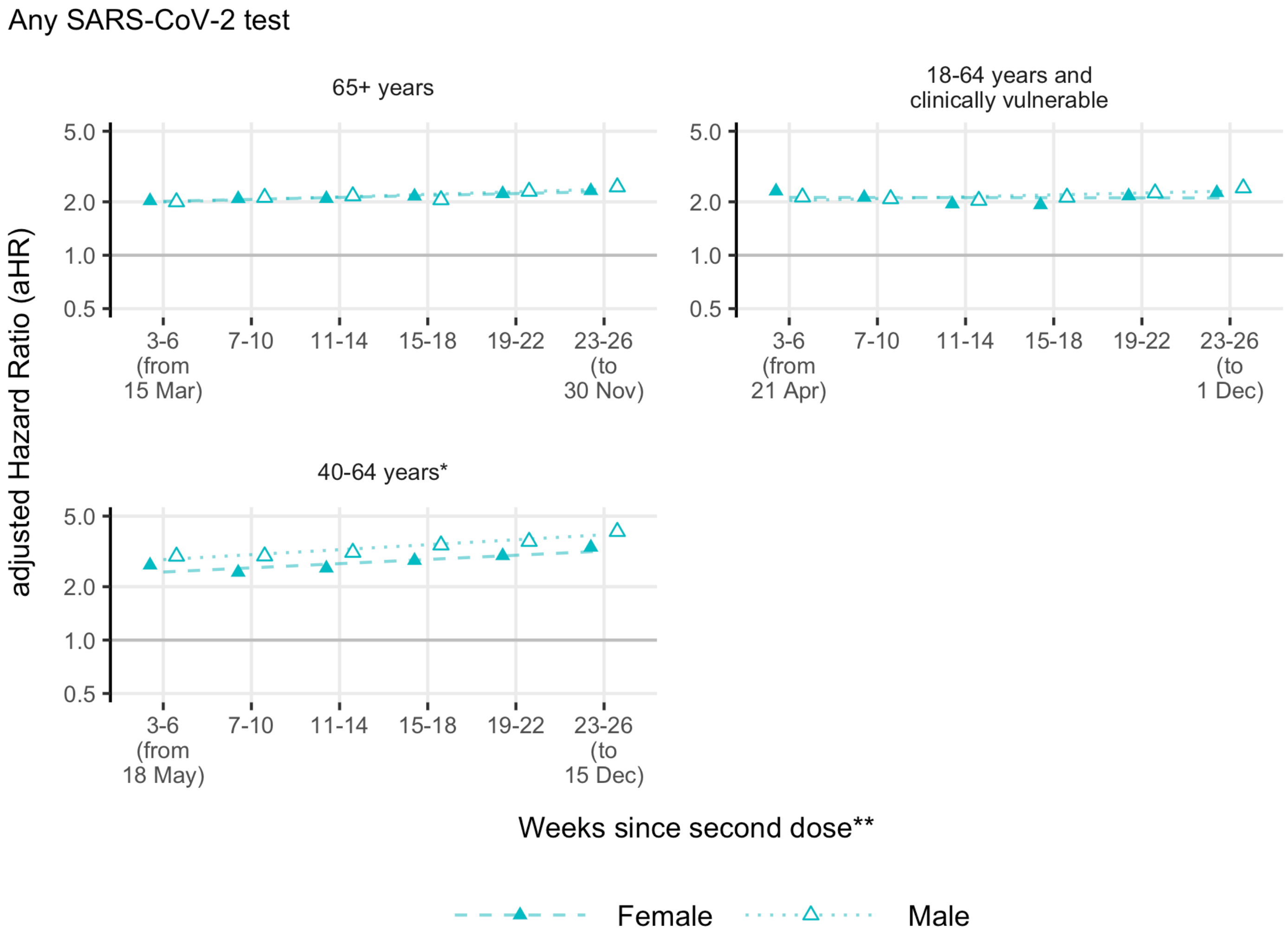

Supplementary Figure 35: Adjusted hazard ratios for any SARS-CoV-2 test for ChAdOx1 vs unvaccinated from the female- and male-only models.

Waning vaccine effectiveness in older adults

This subsection provides additional results for the waning of vaccine effectiveness across two additional subgroups: 65-74 years and 75+ years. Supplementary Table 59 gives the event counts / person-years for each subgroup, outcome and comparison period. Supplementary Tables 60 and 61 give the unadjusted and adjusted hazard ratios respectively. Supplementary Table 57 gives the per-comparison-period ratio of adjusted hazard ratios. The adjusted hazard ratios are plotted in Supplementary Figures 36 and 37 for BNT162b2 vs unvaccinated and ChAdOx1 vs unvaccinated respectively. The adjusted hazard ratios with any SARS-CoV-2 test as the outcome are plotted in Supplementary Figures 38 and 39 for BNT162b2 vs unvaccinated and ChAdOx1 vs unvaccinated respectively.

Supplementary Table 59: Event counts / person-years (older adults).

		65-74 years			75+ years		
**Outcome**	**Weeks since 2nd dose**	**BNT162b2**	**ChAdOx1**	**Unvaccinated**	**BNT162b2**	**ChAdOx1**	**Unvaccinated**
COVID-19 hospitalisation	3-6	7 / 29,386	28 / 59,925	35 / 6,956	28 / 34,286	14 / 24,163	42 / 3,979
	7-10	14 / 29,346	77 / 59,836	91 / 6,594	21 / 34,401	21 / 24,112	28 / 3,598
	11-14	63 / 29,303	224 / 59,731	161 / 6,278	63 / 34,306	119 / 24,054	42 / 3,347
	15-18	112 / 29,256	371 / 59,610	189 / 6,111	196 / 34,193	224 / 23,981	126 / 3,246
	19-22	133 / 29,205	483 / 59,478	182 / 5,932	287 / 34,069	280 / 23,904	140 / 3,097
	23-26	175 / 29,119	546 / 59,269	175 / 5,835	399 / 33,928	294 / 23,819	161 / 3,049
	Total	504 / 175,615	1,729 / 357,849	833 / 37,706	994 / 205,183	952 / 144,033	539 / 20,316
COVID-19 death	3-6	0 / 29,386	7 / 59,926	7 / 6,959	7 / 34,289	0 / 24,164	21 / 3,983
	7-10	7 / 29,347	7 / 59,840	21 / 6,599	0 / 34,405	7 / 24,115	7 / 3,602
	11-14	7 / 29,307	28 / 59,745	35 / 6,291	7 / 34,312	14 / 24,060	14 / 3,352
	15-18	14 / 29,265	49 / 59,643	42 / 6,134	28 / 34,206	35 / 23,998	28 / 3,257
	19-22	28 / 29,221	91 / 59,541	49 / 5,966	70 / 34,097	49 / 23,938	49 / 3,112
	23-26	28 / 29,144	70 / 59,362	42 / 5,876	84 / 33,976	56 / 23,870	56 / 3,074
	Total	84 / 175,670	252 / 358,057	196 / 37,825	196 / 205,285	161 / 144,145	175 / 20,380
Positive SARS-CoV-2 test	3-6	56 / 29,381	196 / 59,911	77 / 6,953	49 / 34,282	56 / 24,159	56 / 3,977
	7-10	245 / 29,333	1,106 / 59,787	203 / 6,586	77 / 34,394	98 / 24,105	35 / 3,595
	11-14	791 / 29,255	2,751 / 59,545	357 / 6,259	287 / 34,291	623 / 24,028	70 / 3,344
	15-18	1,428 / 29,132	4,361 / 59,178	406 / 6,077	854 / 34,146	1,057 / 23,904	182 / 3,240
	19-22	1,638 / 28,968	4,823 / 58,721	378 / 5,885	1,442 / 33,952	1,610 / 23,741	203 / 3,089
	23-26	2,387 / 28,751	7,098 / 58,126	399 / 5,773	1,736 / 33,717	1,701 / 23,557	217 / 3,037
	Total	6,545 / 174,820	20,335 / 355,268	1,820 / 37,533	4,445 / 204,782	5,145 / 143,494	763 / 20,282
Non-COVID-19 death	3-6	210 / 29,386	441 / 59,926	119 / 6,959	644 / 34,289	378 / 24,164	245 / 3,983
	7-10	266 / 29,347	511 / 59,840	98 / 6,599	826 / 34,405	462 / 24,115	196 / 3,602
	11-14	273 / 29,307	616 / 59,745	84 / 6,291	1,015 / 34,312	532 / 24,060	189 / 3,352
	15-18	301 / 29,265	686 / 59,643	98 / 6,134	1,036 / 34,206	560 / 23,998	203 / 3,257
	19-22	329 / 29,221	658 / 59,541	91 / 5,966	1,071 / 34,097	532 / 23,938	168 / 3,112
	23-26	371 / 29,144	756 / 59,362	91 / 5,876	1,197 / 33,976	609 / 23,870	168 / 3,074
	Total	1,750 / 175,670	3,668 / 358,057	581 / 37,825	5,789 / 205,285	3,073 / 144,145	1,169 / 20,380
Any SARS-CoV-2 test	3-6	34,867 / 27,815	75,194 / 56,446	3,451 / 6,664	30,450 / 33,034	24,696 / 23,116	2,492 / 3,766
	7-10	38,696 / 27,591	84,329 / 56,000	3,612 / 6,290	34,482 / 32,898	26,719 / 22,930	2,086 / 3,419
	11-14	42,441 / 27,325	90,237 / 55,418	3,654 / 5,970	38,031 / 32,657	30,114 / 22,732	1,974 / 3,179
	15-18	41,412 / 27,287	89,145 / 55,286	3,787 / 5,785	38,780 / 32,459	29,330 / 22,621	1,974 / 3,086
	19-22	46,151 / 26,982	100,030 / 54,434	3,703 / 5,593	39,795 / 32,302	31,367 / 22,453	1,876 / 2,942
	23-26	51,205 / 26,573	109,263 / 53,530	3,682 / 5,478	43,218 / 31,915	33,957 / 22,150	1,932 / 2,886
	Total	254,772 / 163,573	548,198 / 331,114	21,889 / 35,780	224,756 / 195,265	176,183 / 136,002	12,334 / 19,278

Supplementary Table 60: Unadjusted hazard ratios for effect of vaccination (older adults).

		65-74 years			75+ years		
**Outcome**	**Weeks since 2nd dose**	**BNT162b2 vs unvaccinated**	**ChAdOx1 vs unvaccinated**	**BNT162b2 vs ChAdOx1**	**BNT162b2 vs unvaccinated**	**ChAdOx1 vs unvaccinated**	**BNT162b2 vs ChAdOx1**
COVID-19 hospitalisation	3-6	0.05 (0.02-0.13)	0.11 (0.06-0.19)	0.42 (0.17-1.01)	0.08 (0.04-0.14)	0.18 (0.08-0.39)	0.71 (0.30-1.71)
	7-10	0.05 (0.03-0.09)	0.10 (0.07-0.14)	0.42 (0.24-0.74)	0.10 (0.04-0.22)	0.10 (0.05-0.22)	0.77 (0.34-1.71)
	11-14	0.08 (0.06-0.11)	0.12 (0.10-0.16)	0.59 (0.45-0.79)	0.18 (0.11-0.29)	0.28 (0.18-0.44)	0.50 (0.35-0.71)
	15-18	0.11 (0.09-0.15)	0.16 (0.13-0.20)	0.63 (0.51-0.77)	0.14 (0.11-0.19)	0.20 (0.15-0.26)	0.62 (0.49-0.79)
	19-22	0.12 (0.10-0.16)	0.22 (0.18-0.26)	0.51 (0.42-0.62)	0.18 (0.14-0.23)	0.25 (0.19-0.33)	0.63 (0.52-0.77)
	23-26	0.21 (0.17-0.27)	0.29 (0.24-0.36)	0.68 (0.57-0.81)	0.22 (0.18-0.28)	0.27 (0.21-0.35)	0.71 (0.60-0.85)
COVID-19 death	3-6	-	-	-	0.03 (0.01-0.08)	-	-
	7-10	-	0.03 (0.01-0.09)	-	-	-	-
	11-14	0.04 (0.02-0.10)	0.06 (0.03-0.11)	0.60 (0.27-1.32)	0.03 (0.01-0.11)	0.10 (0.03-0.26)	0.53 (0.15-1.86)
	15-18	0.07 (0.04-0.14)	0.10 (0.07-0.17)	0.66 (0.37-1.19)	0.11 (0.05-0.21)	0.18 (0.08-0.41)	0.50 (0.24-1.07)
	19-22	0.08 (0.05-0.14)	0.12 (0.08-0.18)	0.63 (0.41-0.97)	0.13 (0.08-0.20)	0.13 (0.08-0.21)	0.72 (0.46-1.13)
	23-26	0.16 (0.09-0.28)	0.18 (0.11-0.27)	0.72 (0.45-1.16)	0.12 (0.08-0.18)	0.20 (0.12-0.32)	0.58 (0.42-0.82)
Positive SARS-CoV-2 test	3-6	0.28 (0.19-0.42)	0.55 (0.40-0.76)	0.71 (0.52-0.96)	0.14 (0.09-0.22)	0.38 (0.23-0.63)	0.61 (0.39-0.96)
	7-10	0.41 (0.33-0.51)	0.79 (0.67-0.94)	0.52 (0.45-0.60)	0.34 (0.20-0.59)	0.52 (0.30-0.88)	0.79 (0.56-1.11)
	11-14	0.49 (0.42-0.57)	0.85 (0.74-0.96)	0.63 (0.58-0.68)	0.48 (0.35-0.65)	0.71 (0.53-0.94)	0.57 (0.49-0.66)
	15-18	0.75 (0.66-0.85)	1.06 (0.95-1.19)	0.69 (0.65-0.73)	0.45 (0.37-0.54)	0.64 (0.53-0.77)	0.72 (0.65-0.80)
	19-22	0.84 (0.74-0.96)	1.31 (1.17-1.47)	0.69 (0.65-0.73)	0.63 (0.53-0.74)	0.89 (0.74-1.06)	0.75 (0.70-0.82)
	23-26	1.33 (1.18-1.51)	1.85 (1.65-2.07)	0.73 (0.70-0.77)	0.71 (0.61-0.83)	0.98 (0.83-1.17)	0.78 (0.72-0.85)
Non-COVID-19 death	3-6	0.41 (0.31-0.54)	0.38 (0.30-0.48)	0.99 (0.83-1.18)	0.30 (0.25-0.35)	0.54 (0.42-0.69)	0.68 (0.57-0.81)
	7-10	0.46 (0.35-0.60)	0.43 (0.34-0.55)	1.07 (0.92-1.24)	0.46 (0.38-0.55)	0.76 (0.59-0.99)	0.69 (0.59-0.80)
	11-14	0.56 (0.42-0.75)	0.65 (0.50-0.84)	0.90 (0.78-1.04)	0.54 (0.46-0.65)	0.72 (0.56-0.91)	0.83 (0.72-0.94)
	15-18	0.51 (0.39-0.68)	0.62 (0.48-0.79)	0.88 (0.77-1.02)	0.52 (0.44-0.62)	0.74 (0.59-0.93)	0.77 (0.68-0.87)
	19-22	0.55 (0.42-0.73)	0.53 (0.42-0.68)	0.99 (0.86-1.13)	0.65 (0.54-0.78)	0.90 (0.70-1.16)	0.89 (0.78-1.02)
	23-26	0.61 (0.47-0.80)	0.62 (0.49-0.79)	0.99 (0.87-1.12)	0.62 (0.52-0.73)	0.87 (0.69-1.11)	0.84 (0.74-0.95)
Any SARS-CoV-2 test	3-6	2.68 (2.58-2.79)	3.06 (2.95-3.18)	0.87 (0.86-0.88)	1.52 (1.45-1.59)	2.39 (2.26-2.53)	0.83 (0.82-0.85)
	7-10	2.73 (2.63-2.84)	3.04 (2.94-3.16)	0.86 (0.85-0.88)	2.02 (1.92-2.12)	2.80 (2.64-2.97)	0.85 (0.83-0.87)
	11-14	2.71 (2.61-2.81)	3.05 (2.94-3.16)	0.87 (0.86-0.88)	2.11 (2.01-2.22)	2.74 (2.58-2.91)	0.87 (0.85-0.88)
	15-18	2.59 (2.50-2.69)	2.92 (2.82-3.03)	0.87 (0.86-0.88)	2.15 (2.04-2.25)	2.85 (2.68-3.03)	0.86 (0.84-0.87)
	19-22	2.79 (2.68-2.89)	3.15 (3.05-3.27)	0.87 (0.86-0.88)	2.18 (2.08-2.30)	3.01 (2.83-3.21)	0.86 (0.85-0.88)
	23-26	3.07 (2.96-3.19)	3.40 (3.28-3.52)	0.89 (0.88-0.90)	2.26 (2.15-2.37)	3.06 (2.88-3.26)	0.87 (0.86-0.89)

Supplementary Table 61: Adjusted hazard ratios for effect of vaccination (older adults).

Outcome	Weeks since 2nd dose	65-74 years			75+ years		
		BNT162b2 vs unvaccinated	ChAdOx1 vs unvaccinated	BNT162b2 vs ChAdOx1	BNT162b2 vs unvaccinated	ChAdOx1 vs unvaccinated	BNT162b2 vs ChAdOx1
COVID-19 hospitalisation	3-6	0.05 (0.02-0.14)	0.13 (0.06-0.27)	0.41 (0.17-1.00)	0.08 (0.04-0.15)	0.18 (0.09-0.40)	0.73 (0.31-1.75)
	7-10	0.05 (0.03-0.10)	0.11 (0.07-0.16)	0.41 (0.23-0.72)	0.09 (0.04-0.20)	0.12 (0.05-0.26)	0.79 (0.35-1.76)
	11-14	0.07 (0.05-0.10)	0.12 (0.09-0.15)	0.58 (0.44-0.78)	0.17 (0.10-0.31)	0.28 (0.17-0.45)	0.50 (0.35-0.71)
	15-18	0.12 (0.08-0.16)	0.17 (0.14-0.22)	0.62 (0.50-0.76)	0.14 (0.10-0.19)	0.19 (0.13-0.26)	0.64 (0.51-0.81)
	19-22	0.12 (0.09-0.16)	0.21 (0.17-0.26)	0.50 (0.41-0.61)	0.18 (0.14-0.24)	0.22 (0.17-0.29)	0.64 (0.53-0.78)
	23-26	0.21 (0.16-0.27)	0.26 (0.21-0.32)	0.66 (0.56-0.79)	0.20 (0.16-0.26)	0.23 (0.17-0.31)	0.72 (0.60-0.86)
COVID-19 death	3-6	-	-	-	0.03 (0.01-0.08)	-	-
	7-10	-	0.03 (0.01-0.10)	-	-	-	-
	11-14	0.04 (0.02-0.10)	0.06 (0.03-0.11)	0.59 (0.26-1.31)	-	0.17 (0.05-0.56)	0.54 (0.54-0.54)
	15-18	0.06 (0.03-0.12)	0.10 (0.06-0.16)	0.66 (0.37-1.18)	0.10 (0.05-0.22)	0.16 (0.06-0.43)	0.51 (0.24-1.08)
	19-22	0.08 (0.04-0.13)	0.12 (0.07-0.20)	0.62 (0.41-0.95)	0.12 (0.07-0.19)	0.12 (0.07-0.20)	0.72 (0.46-1.12)
	23-26	0.13 (0.07-0.24)	0.15 (0.09-0.24)	0.72 (0.44-1.15)	0.11 (0.07-0.19)	0.18 (0.11-0.29)	0.59 (0.42-0.83)
Positive SARS-CoV-2 test	3-6	0.26 (0.17-0.42)	0.48 (0.33-0.68)	0.71 (0.52-0.96)	0.12 (0.07-0.21)	0.36 (0.22-0.60)	0.62 (0.39-0.98)
	7-10	0.36 (0.28-0.46)	0.73 (0.60-0.88)	0.52 (0.45-0.60)	0.28 (0.15-0.53)	0.45 (0.25-0.82)	0.79 (0.56-1.11)
	11-14	0.40 (0.34-0.48)	0.71 (0.61-0.82)	0.63 (0.58-0.68)	0.40 (0.29-0.57)	0.68 (0.50-0.93)	0.57 (0.49-0.66)
	15-18	0.65 (0.56-0.76)	0.93 (0.82-1.05)	0.69 (0.65-0.73)	0.41 (0.33-0.50)	0.53 (0.43-0.66)	0.72 (0.65-0.80)
	19-22	0.69 (0.60-0.80)	1.11 (0.98-1.26)	0.69 (0.65-0.73)	0.61 (0.50-0.73)	0.74 (0.61-0.90)	0.75 (0.69-0.82)
	23-26	1.08 (0.94-1.24)	1.42 (1.26-1.60)	0.74 (0.70-0.77)	0.58 (0.48-0.69)	0.77 (0.63-0.93)	0.78 (0.72-0.84)
Non-COVID-19 death	3-6	0.39 (0.27-0.55)	0.32 (0.24-0.42)	0.97 (0.82-1.16)	0.26 (0.21-0.32)	0.42 (0.32-0.55)	0.73 (0.62-0.86)
	7-10	0.47 (0.34-0.65)	0.47 (0.36-0.63)	1.05 (0.91-1.23)	0.40 (0.32-0.50)	0.56 (0.42-0.74)	0.74 (0.64-0.85)
	11-14	0.51 (0.36-0.72)	0.70 (0.52-0.95)	0.89 (0.77-1.03)	0.52 (0.41-0.64)	0.61 (0.46-0.80)	0.87 (0.76-0.99)
	15-18	0.49 (0.36-0.67)	0.66 (0.50-0.86)	0.87 (0.76-1.00)	0.49 (0.40-0.61)	0.58 (0.45-0.74)	0.81 (0.72-0.92)
	19-22	0.56 (0.40-0.77)	0.60 (0.45-0.79)	0.98 (0.86-1.12)	0.61 (0.49-0.76)	0.77 (0.58-1.01)	0.94 (0.82-1.07)
	23-26	0.56 (0.42-0.76)	0.73 (0.56-0.97)	0.98 (0.86-1.11)	0.58 (0.47-0.72)	0.75 (0.58-0.98)	0.88 (0.78-1.00)
Any SARS-CoV-2 test	3-6	1.89 (1.81-1.98)	2.17 (2.09-2.26)	0.87 (0.86-0.88)	1.15 (1.09-1.21)	1.67 (1.57-1.77)	0.85 (0.83-0.86)
	7-10	1.94 (1.86-2.03)	2.14 (2.06-2.22)	0.87 (0.86-0.88)	1.53 (1.45-1.62)	1.97 (1.85-2.10)	0.86 (0.85-0.88)
	11-14	1.92 (1.84-2.00)	2.18 (2.10-2.26)	0.87 (0.86-0.89)	1.60 (1.52-1.69)	1.94 (1.82-2.06)	0.88 (0.86-0.89)
	15-18	1.89 (1.82-1.97)	2.11 (2.03-2.19)	0.87 (0.86-0.88)	1.64 (1.56-1.73)	2.06 (1.94-2.20)	0.87 (0.85-0.88)
	19-22	2.02 (1.94-2.11)	2.27 (2.19-2.35)	0.88 (0.87-0.89)	1.67 (1.58-1.76)	2.19 (2.05-2.34)	0.87 (0.86-0.89)
	23-26	2.25 (2.16-2.34)	2.42 (2.33-2.51)	0.89 (0.88-0.90)	1.74 (1.65-1.84)	2.17 (2.04-2.32)	0.88 (0.87-0.89)

Supplementary Table 62: Per-comparison-period ratio of adjusted hazard ratios for effect of vaccination (older adults).

Outcome	65-74 years			75+ years		
	BNT162b2 vs unvaccinated	ChAdOx1 vs unvaccinated	BNT162b2 vs ChAdOx1	BNT162b2 vs unvaccinated	ChAdOx1 vs unvaccinated	BNT162b2 vs ChAdOx1
COVID-19 hospitalisation	1.36 (1.22-1.52)	1.25 (1.15-1.35)	1.06 (0.96-1.18)	1.18 (1.06-1.31)	1.06 (0.94-1.19)	1.06 (0.96-1.16)
COVID-19 death	1.47 (1.07-2.01)	1.38 (1.12-1.70)	1.06 (0.80-1.39)	1.28 (1.02-1.60)	1.07 (0.75-1.53)	0.99 (0.69-1.43)
Positive SARS-CoV-2 test	1.32 (1.23-1.41)	1.22 (1.17-1.27)	1.06 (1.02-1.09)	1.29 (1.14-1.46)	1.14 (1.04-1.24)	1.06 (1.01-1.12)
Non-COVID-19 death	1.07 (0.99-1.15)	1.15 (1.05-1.27)	1.00 (0.96-1.03)	1.16 (1.08-1.26)	1.11 (1.05-1.19)	1.04 (1.01-1.08)
Any SARS-CoV-2 test	1.03 (1.01-1.05)	1.02 (1.00-1.04)	1.00 (1.00-1.01)	1.07 (1.02-1.12)	1.05 (1.03-1.07)	1.01 (1.00-1.01)

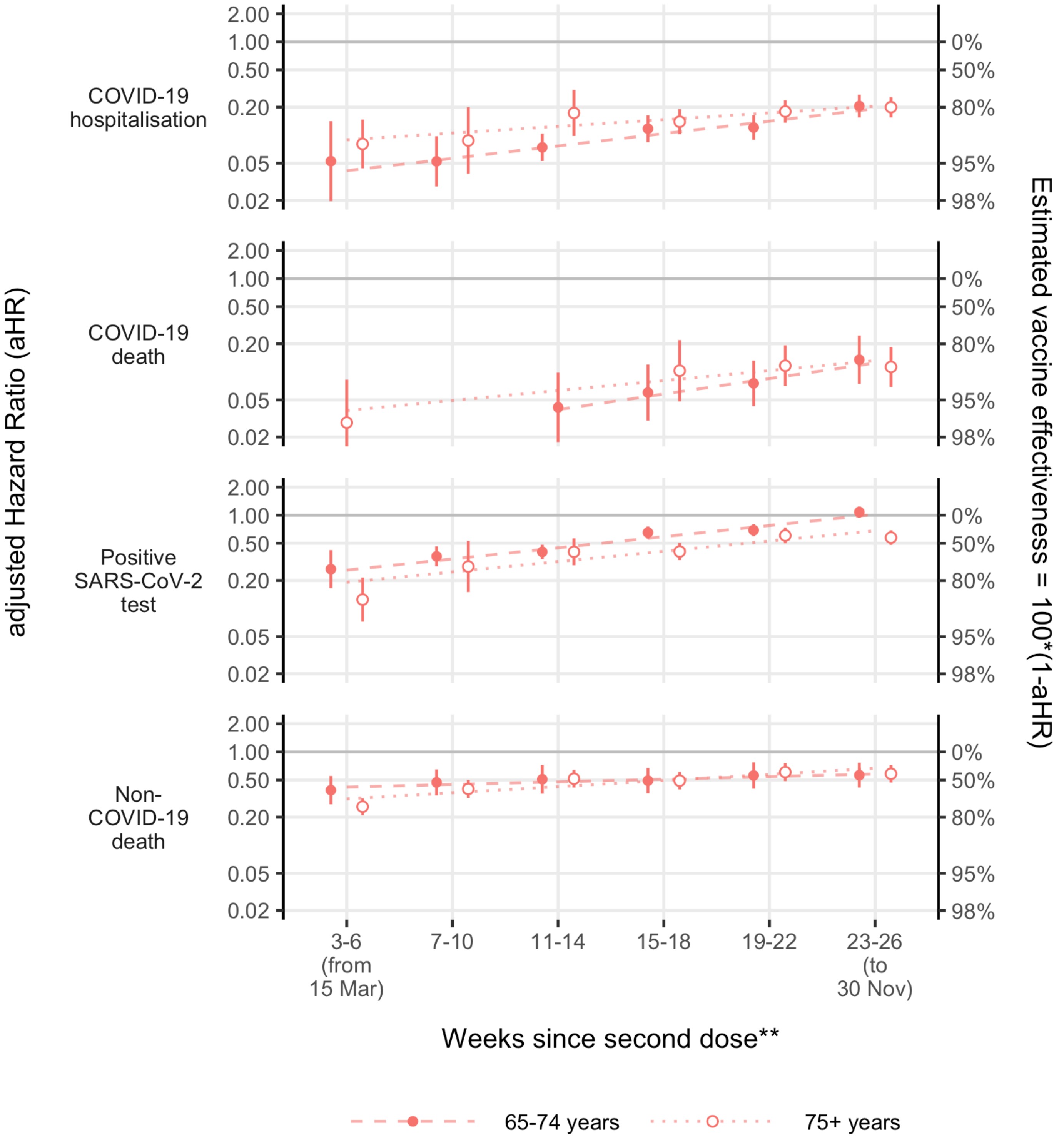

Supplementary Figure 36: Adjusted hazard ratios for BNT162b2 vs unvaccinated from the 65-74 years and 75+ years models.

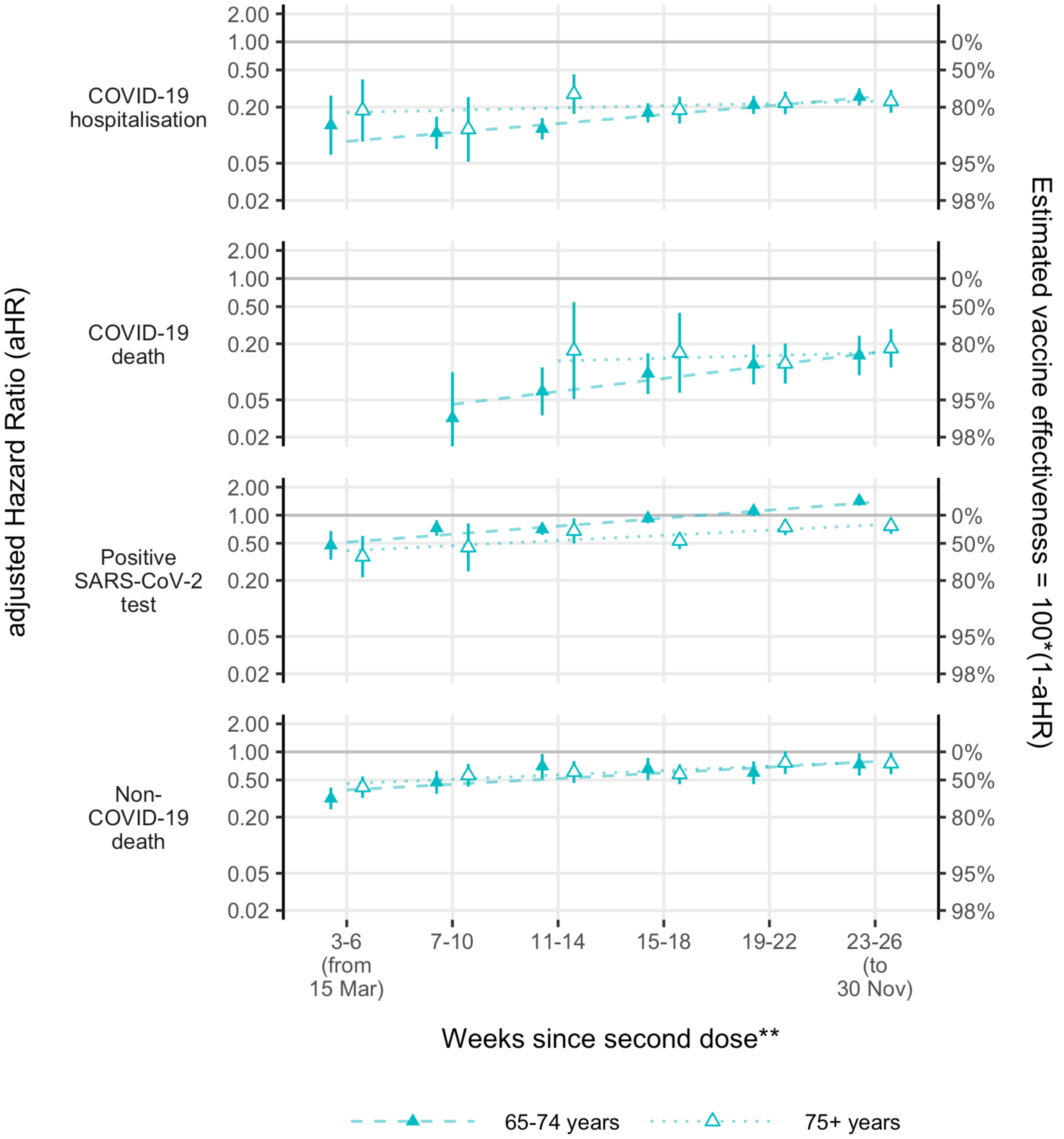

Supplementary Figure 37: Adjusted hazard ratios for ChAdOx1 vs unvaccinated from the 65-74 years and 75+ years models.

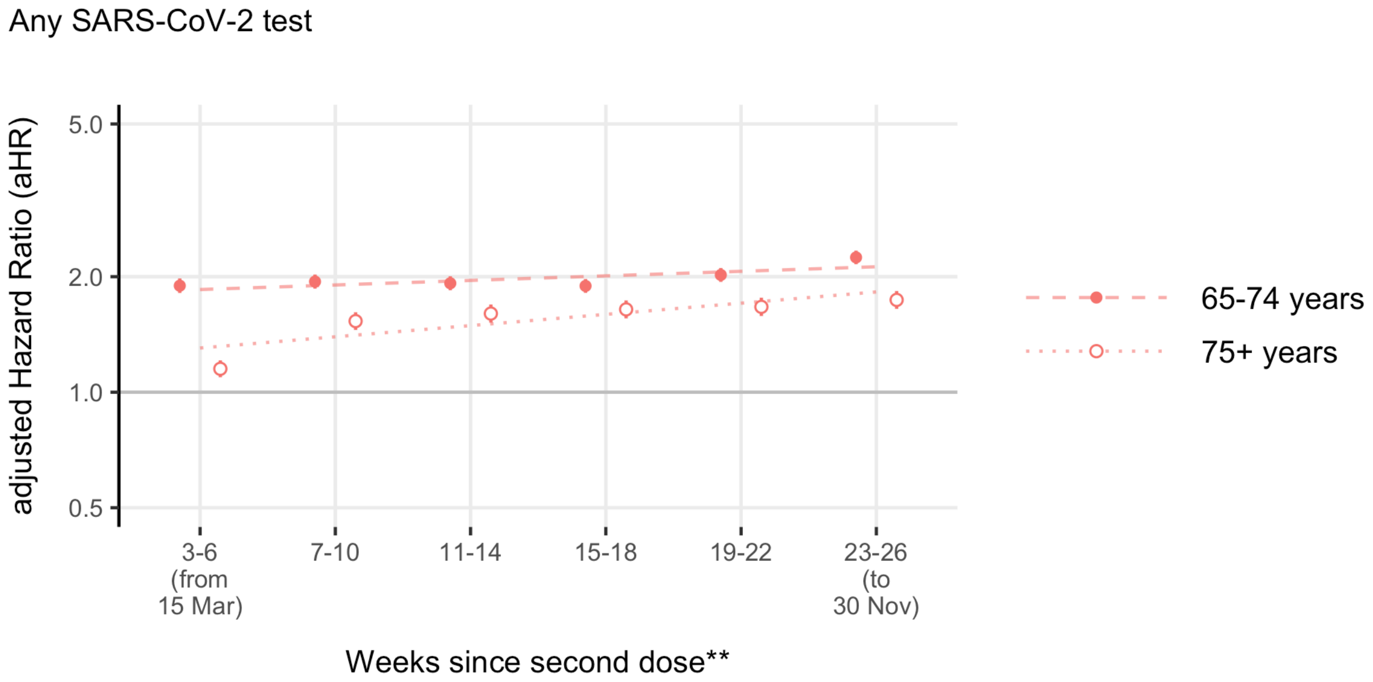

Supplementary Figure 38: Adjusted hazard ratios for any SARS-CoV-2 test for BNT162b2 vs unvaccinated from the 65-74 years and 75+ years models.

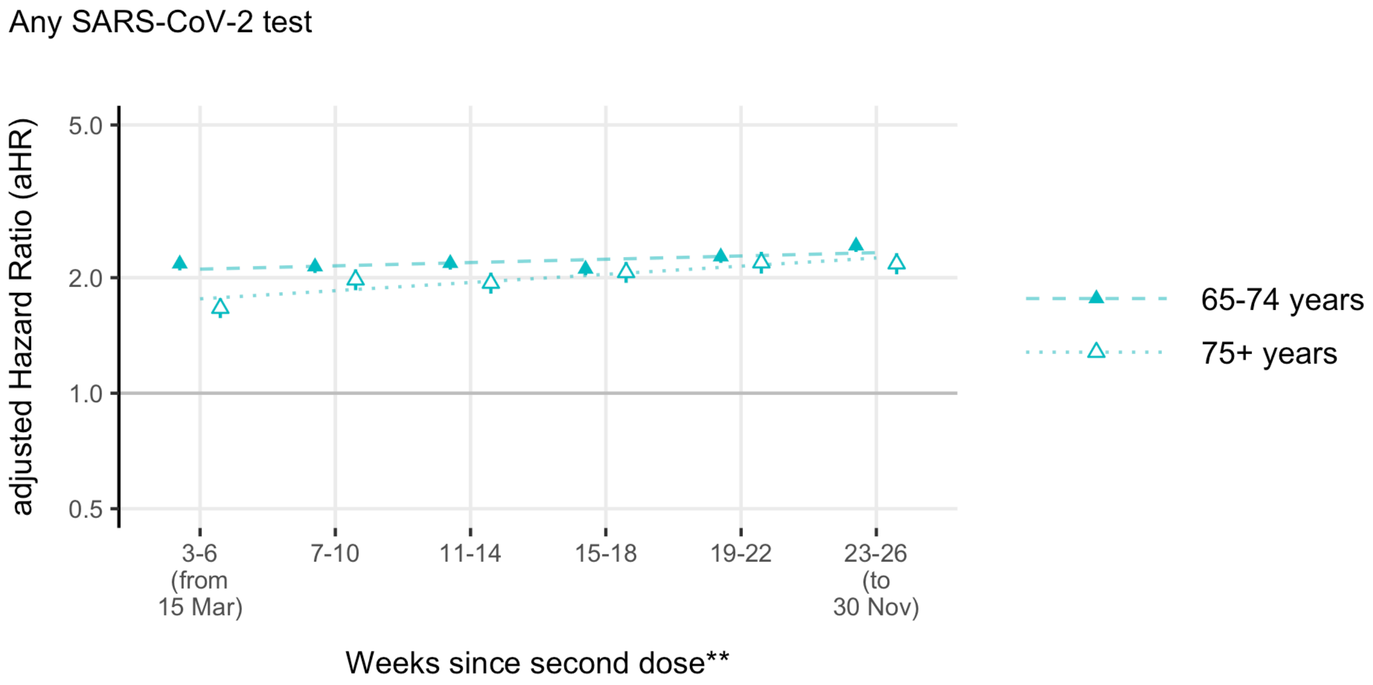

Supplementary Figure 39: Adjusted hazard ratios for any SARS-CoV-2 test for ChAdOx1 vs unvaccinated from the 65-74 years and 75+ years models.
